# Supplementary material for: Association of 25(OH)D serum level with biological aging: A Cross-Sectional Study of 2007–2016 NHANES surveys
Source: PLoS One. 2025 Aug 6;20(8):e0328107. doi: 10.1371/journal.pone.0328107 (PMC12327616; doi:10.1371/journal.pone.0328107)
Supplement: S1 File — (DOCX) [file pone.0328107.s002.docx]

**Statistical analysis plan**

**Research purpose**

This cross-sectional study aims to investigate the relationship between serum 25(OH)D level and biological aging.

**Data source**

Data from this study were obtained from National Health and Nutrition Examination Survey (NHANES), a nationwide survey conducted by the National Center for Health Statistics (NCHS) of the Centers for Disease Control and Prevention (CDC). All data used in current study is publicly available from NHANES website (https://wwwn.cdc.gov/nchs/nhanes/Default.aspx).

**Variables**

a. Exposure variable: Serum 25(OH)D levels

Serum 25(OH)D levels was calculated as the sum of serum 25(OH)D3 and 25(OH)D2 levels, which was reported in nmol/L. Due to skewed distribution, serum 25(OH)D level was log-transformed for regression analyses. Serum 25(OH)D level was further categorized according to clinical sufficiency and quartiles.

b. Outcome variable: Biological aging

Biological aging was indicated as PhenoAge acceleration, which was calculated as the difference between PhenoAge and chronological age. A positive value indicated accelerated aging. PhenoAge acceleration was standardized to z-scores (mean=0, SD=1).

c. Covariates

Covariates were selected according to previous researches considered confounding in vitamin D and aging research. Model adjustment included age (continuous, years), race (Non-Hispanic white/Non-Hispanic black/others), education level (below high school/high school or equivalent/above high school), poverty-income ratio (≤1.0/1.0-3.0/>3.0), body mass index (continuous, kg/m²), smoking (never/previous/current), drinking (never/previous/current), physical activity (inactive/insufficient/moderate/high), sleep duration (<7 h/7-8 h/≥8 h), hypertension (no/yes), diabetes (no/yes) and season of measurement (summer/winter).

For questionnaire acquired data, responses for “Refused/Don’t know.” were treated as missing. This study aplied complete case analysis, hence participants with missing data were excluded.

**Statistical analysis**

a. Descriptive analysis

Continuous variables were reported as mean ± standard deviation, and median (interquartile range) if non-normally distributed. Categorical variables were presented as frequency (percentage). Difference between subgroups (male and female) was assessed using student’s t-test for normally distributed variables, Mann-Whitney test for non-normal variables and Chi-square test for categorical variables.

b. Primary analysis

To investigate the relationships between serum 25(OH)D level and biological aging, two weighted linear regression models were constructed: a crude model and a fully adjusted model adjusted for age, race, education level, poverty-income ratio, body mass index, smoking, drinking, physical activity, sleep duration, hypertension, diabetes and season of measurement. Serum 25(OH)D level was modeled as independent variable after three parameterizations: continuous log-transformed values, clinical categories, and quartiles. Biological aging, quantified as standardized PhenoAge advancement, was put in the models as dependent variable. The relationships between serum 25(OH)D level and biological aging were examined in total population, as well as in male and female subgroups.

c. Secondary analysis

To explore effect modification, the relationships between serum 25(OH)D level and biological aging were further tested across subgroups, stratified by age (<60 y/≥60 y), BMI (<25.00/≥25.00), smoking status (no/yes), drinking status (no/yes), physical activity (inactive or insufficient/moderate or high), hypertension (no/yes), diabetes (no/yes) and sleep duration (<7 h/7-8 h/≥8 h). Interaction term of each stratification factor and serum 25(OH)D status was constructed and put into the regression model to test interaction effect. Non-linear relationship between serum 25(OH)D level and biological aging was further tested by generalized additive models. Segmented regression model was then constructed to determine the threshold effect.

**Statistical software**

All analyses are performed using R version 4.2.1 (R Core Team, Vienna, Austria) with packages including BioAge (for PhenoAge calculation) and mgcv (for GAM anlysis).
