# Supplementary material for: Association of 25(OH)D serum level with biological aging: A Cross-Sectional Study of 2007–2016 NHANES surveys
Source: PLoS One. 2025 Aug 6;20(8):e0328107. doi: 10.1371/journal.pone.0328107 (PMC12327616; doi:10.1371/journal.pone.0328107)
Supplement: Attachment — Submitted filename: PONE-D-24-25180_R1-Academic Editor comments.pdf [file pone.0328107.s006.pdf]

PLOS ONE

Association of serum 25(OH)D level and physiological aging: A Cross-Sectional Study of NHANES 2007-2016

--Manuscript Draft--

**Manuscript Number:**	PONE-D-24-25180R1
**Article Type:**	Research Article
**Full Title:**	Association of serum 25(OH)D level and physiological aging: A Cross-Sectional Study of NHANES 2007-2016
**Short Title:**	aging and serum 25(OH)D level
**Corresponding Author:**	Jue Wang<br>nanhui new town community health service center<br>shanghai, CHINA
**Keywords:**	vitamin D; serum 25(OH)D; Age advancement; PhenoAge; Nonlinear correlation
**Abstract:**	Vitamin D plays a crucial role in various aspects of human body, including aging, but chronological age may not accurately reflect true physiological aging status. Recently, PhenoAge has been developed to estimate an individual's biological age based on different physiological and clinical measures. Therefore, we investigated the relationship between serum 25(OH)D levels and physiological aging calculated by PhenoAge in the National Health and Nutrition Examination Survey (NHANES). Biological age showed a negative correlation with serum 25(OH)D levels (β=-0.04; 95% CI: -0.08-0.00, p=0.039), but this correlation was only found to be dose-dependent for females (β=-0.07; 95% CI: -0.12-0.01, p=0.007), but not for males. Further analysis confirmed that these associations were nonlinear in both sexes, revealing a U-shaped curve for males and an L-shaped curve for females. Specifically, serum 25(OH)D levels were linked to decelerated aging at levels below 38.20 nmol/L in males (β=-0.013; 95% CI: -0.025--0.002, p=0.017) and 62.50 nmol/L in females (β=-0.007; 95% CI: -0.01--0.004, p<0.001). However, high serum 25(OH)D levels (>125 nmol/L) did not correlate with aging in females (β=-0.001; 95% CI: -0.005-0.002), while in males, elevated levels (>91.55 nmol/L) were associated with accelerated aging (β=0.005; 95% CI: 0.002-0.008, p<0.001). Our findings indicate that vitamin D deficiency shows a potential link to accelerated physiological aging, but high levels of vitamin D do not link to decelerated physiological aging, offering valuable insights into the relationship between vitamin D and aging.
**Order of Authors:**	Yi Feng
	Licheng Yu
	Jue Wang
**Opposed Reviewers:**	
**Response to Reviewers:**	Dear editor and reviewer:<br>Thank you very much for your careful review and constructive suggestions with regard to our manuscript. Those comments are helpful for us to revise and improve our paper. We have studied comments carefully and tried our best to revise and improve the manuscript and made great changes in the manuscript according to the good comments. We earnestly appreciate for the warm work from you, and hope that the corrections will meet with approval. Please feel free to contact us with any questions and we are looking forward to your consideration. The main corrections in the paper and the responds to your comments are as following:<br><br>Additional Editor Comments:<br>In addition to the reviewer's comments, I have the following comments:<br>1.In the title, you would better replace "age advancement" with "biological aging" or "physiological aging" given that PhenoAge used as metric of aging in this study is a measure of biological/physiological aging.<br>Response: Thank you for your insightful feedback. We have revised the title to use "biological aging" instead of "age advancement" to more accurately reflect our study's focus on the biological aspects measured by the PhenoAge metric.

2.The authors should revise the "Materials and methods", results and discussion sections based on the STROBE guidelines statement (see von Elm et al. PLoS Med. 2007;4(10):e296) to allow for the reproducibility of their methods. Accordingly, they should start the "Materials and methods section" stating that their report conforms to STROBE guidelines for observational studies, and upload a STROBE checklist in the appendix. The "Statistical analysis" sub-section should follow the SAMPL guideline statement (see Lang TA et al. Medical Writing.2016;25(3):31-36) and the authors should upload a statistical analysis plan for this study as well as raw data (if legal frameworks of NHANES allow that) as supplemental materials to this manuscript. This recommendation aims to improve the readability of their manuscript, allow the credibility and reproducibility of their study methods, and increase the credibility of their findings and trustworthiness of the interpretation of their findings.
Response: Thank you for your constructive suggestions and guidance. We have thoroughly revised the manuscript to ensure alignment with STROBE guidelines for observational studies, as well as the SAMPL recommendations for statistical reporting. Meanwhile, in the Materials and methods section, we supplemented the following statement: “This study was conducted and reported in adherence to STROBE guidelines for observational studies.” (marked in blue, Page 4, Lines 95). The completed STROBE checklist is provided in Supplementary materials. A statistical analysis plan has also been written and provided in Supplementary materials. Regarding data accessibility, all raw data was publicly available through the NHANES website: https://wwwn.cdc.gov/nchs/nhanes/Default.aspx. According to the NHANES data use agreement, redistribution of raw datasets is prohibited. Therefore, we are unable to provide NHANES raw data directly. This has been clarified in the Materials and methods section: “All data included in current study are publicly available through the NHANES website [18].” (marked in blue, Page 5, Lines 99).
The following reference was supplemented in the References List:
19.Centers for Disease Control and Prevention National Health and Nutrition Examination Survey Questionnaires D, and Related Documentation 2020. [cited 2025 March 7]. Available from: https://wwwn.cdc.gov/nchs/nhanes/.

3.The "Materials and methods" section should end with a sub-section titled "Ethical considerations"in which the authors should state whether they abode by the ethical rules for conducting medical/health research (see the 2024 declaration of Helsinki statement published in JAMA), how they accessed NHANES (USA) data whereas all authors are affiliated with China institutes and the study was funded by a Chinese organization). Did they get an IRB approval to use the NHANES dataset?
Response: We thank the editor for your constructive suggestions and guidance. Our research adhered to the ethical principles in the 2024 Declaration of Helsinki regarding secondary analysis. It exempts studies from additional ethical review when utilizing pre-existing de-identified data [1]. Meanwhile the NHANES datasets we used were publicly available and did not contain restricted use datasets. According to the CDC/NCHS Research Ethics Review Board statement, researchers using only the publicly available data files do not need to seek IRB approval [2]. Hence, no additional IRB was required in our study.
In the Materials and methods section, we have supplemented the “Ethical considerations” sub-section in the revised manuscript: “This study utilized data from NHANES study, which was conducted in accordance with the principles in the Declaration of Helsinki. The NHANES protocol received prior ethical approval from the National Center for Health Statistics' Review Board. Since our study is a secondary analysis of publicly accessible, de-identified NHANES data sets, additional institutional review board approval was not required [26, 27].” (marked in blue, Page 7, Lines186).
The following reference was supplemented in the References List:
26.University of Connecticut. Guidance on secondary analysis of existing data sets. [cited 2025 Feb 5]. Available from: https://ovpr.uconn.edu/services/rics/irb/researcher-guide/secondary-analysis-of-data-sets/.
27.World Medical A. World Medical Association Declaration of Helsinki: Ethical Principles for Medical Research Involving Human Participants. JAMA. 2025;333(1):71-4. Epub 2024/10/19 20:42. doi: 10.1001/jama.2024.21972. PubMed PMID: 39425955.

4.In the conflicts of interest statement on the manuscript's agenda, the authors should

Powered by Editorial Manager® and ProduXion Manager® from Aries Systems Corporation

clarify any potential conflict of interest with the funder and/or NHANES database managers.
Response: We thank the editor for your guidance. We have revised the “Conflict of interest” statement in the manuscript as follows: “The authors declare no competing financial or non-financial interests related to this work. NHANES is a publicly available dataset managed by the Centers for Disease Control and Prevention. The authors confirm that there is no affiliation with or involvement from the NHANES program administrators in this study. The funding source had no role in study design, data collection, analysis, or manuscript preparation.”.

5.It is difficult to know in tables and figures what type of effect magnitude estimates (odds ratios? Others?) the authors base their conclusions on and whether those estimates are those that should be used in cross-sectional studies to assess associations: see Thiese MS. Observatiobal and interventional study design types; an overview. Biochemistry Med (Zagreb).2014;24(2):199-210. Did you perform logistic regression analyses and/ or correlation analyses? Why perform both or why perform one of them? These clarifications need to be made in the statistical analysis sub-section of the "Materials and methods" section. Along these lines, I would like to remember that the authors overstated their conclusions (see Schooling MC and Heidi JE. Clarifying questions about "risk factors": predictors versus explanation. Epidemiology in Themes. 2018;15:10), and this should be corrected throughout the manuscript.
Response: We sincerely thank the editor for your guidance. Regarding the choice of effect estimates, our analyses utilized beta coefficients derived from weighted multivariable linear regression models. According to Thiese et al., odds ratios are standard for binary outcomes in logistic regression. However, when measuring continuous response variables, beta coefficients are recommended as they quantify linear association between continuous variables. To improve the clarity of our manuscript, we supplemented the following sentences in the Materials and methods section: “Beta coefficients with 95% confidence intervals (CIs) were reported, representing the change in dependent variable per unit increase in the independent variable. The use of beta coefficients provides interpretable estimates for linear associations when analyzing continuous dependent variables, which is widely recommended in epidemiological studies.” (marked in blue, Page 7). Meanwhile, we made adjustment to all Tables and Figures, as well as description of results to ensure the use of beta coefficient is straightforward.
We also supplemented the following sentences in the Materials and methods section to explain why correlation analysis is omitted in current study: “Correlation analysis was not employed because it does not account for confounding factors, whereas the primary aim of current study is to estimate the confounder-adjusted association between 25(OH)D status and PhenoAge aging.” (marked in blue, Page 7).

The following reference was supplemented in the References List:
24.Bender R. Introduction to the use of regression models in epidemiology. Cancer epidemiology: Springer; 2009. P. 179-95

Regarding the potential overstatement of conclusion, we have revised the manuscript following the guidance of Schooling and Heidi (2018). Adjustments have been made throughout the Abstract, Results, and Discussion and Conclusion sections to avoid formative and causal language.

6.The authors cite res 4 to 6 for the definition of vitamin D deficiency but use ref 18 for the definition of vitamin D deficiency in this study. Why can't they use only reference 6 in the introduction and for the definition in this study given that it is the most recently published article? Can you specify whether you assessed the human form of 25-OH-D (25-OH-D3) throughout the manuscript?
Response: We thank the editor for your careful reading and constructive suggestions. We realize that the previous version of the manuscript cited multiple references regarding the deficiency of vitamin D level, which induced redundancy. We agree that ref 6 is the latest and authoritative source for defining vitamin D status. Therefore, we have unified citation of ref 6 in both introduction and methods sections, meanwhile remove ref 4-5 and 18 from our revised manuscript.
Regarding the detected form of 25-OH-D, we supplemented the following sentences in

Powered by Editorial Manager® and ProduXion Manager® from Aries Systems Corporation

the Methods to improve the clarity of our manuscript: “From cycle 2007-2008 and beyond, liquid chromatography-tandem mass spectrometry (LC-MS/MS) was used to measure serum 25(OH)D concentrations, including 25(OH)D3, epi-25(OH)D3, and 25(OH)D2 in human serum. We calculated the 25(OH)D levels by adding 25(OH)D3 and 25(OH)D2 levels.” (marked in blue, Page 5).

7.After reading your introduction, I still do not well understand the rationale for conducting this study given that as well mentioned in reference 6 of the current manuscript, it is largely known that older individuals generally have lower vitamin D levels than younger populations. Do you want to asses whether that knowledge based on social age could hold even when using biological aging metrics (taking into consideration the discrepancy between social and biological ages)? The aim and rationale for conducting the study should be unambiguous in the last paragraph of the introduction. Still regarding the introduction, it is important to begin to be more cautious and specific when making claims about the involvement of vitamin D in human disease based on epidemiological data (see Vitamin D and human healrh: evidence from Mendelian randomization studies. Eur J Epidemiol. 2024;39: 467-490 and Schooling MC and Jones HE. Clarifying questions about "risk factors": predictors versus explanation. Epidemiology in Themes. 2018; 15:10). From my perspective, in a study whereby you seemingly assess the epidemiological link between vitamin D and physiological aging, the introduction should (in a brief overview form: introduction of no more than 1.5 pages) highlight how the human form of vitamin D evolves throughout the age spectrum based on the most certain available peer-reviewed evidence, as well as what is known and what is unknown about the epidemiological association between the human form of vitamin D and physiological aging, before stating the rationale for conducting the study (gaps in knowledge you would like to fill) and the study's aim.
Response: Thank you for your feedback regarding the introduction section of our manuscript. We have rewritten the introduction section to make it clear and concise in the revised manuscript. Briefly, the aim of current study is to investigate the relationship between vitamin D and physiological aging. We have read the articles mentioned above and rewrite the introduction of vitamin D in human disease based on epidemiological data. The length of introduction has been limited to less than 2 pages, a little longer than 1.5 pages. We have done our best to improve the induction section and hope this improvement could meet your requirement. Thank you very much.

8. Finally, language editing is also required.
Response: Thank you for your valuable feedback and suggestions for improvement. I acknowledge the need for language editing to enhance the clarity, precision, and professionalism of the manuscript. I will thoroughly review and ask help from professional language improvement to ensure that the text is clear and effectively conveys the intended meaning.

Reviewer #1:
Feng et al conduct this research in a rare direction while most of researchers will focus on the association between 25(OH)D and disease. Here are some issues should be concerned.

1.In the abstract, there is no statistical results. The conclusion that VD deficiency promotes aging is not proper since cross sectional study only remind the possible association between exposure and outcome instead of causuality.
Response: Thank you for your thoughtful feedback. We have revised the abstract to include key statistical results to better inform readers about the study's findings. Additionally, we acknowledge your point regarding the limitations of cross-sectional studies in establishing causality. You are correct that such studies can only identify associations, and we will revise the language in the abstract and manuscript to reflect this appropriately. Specifically, we will modify the conclusion to state that vitamin D deficiency "may be associated with" or "shows a potential link to" biological aging, rather than implying causation.

2.The introduction section is a little long, in which 3-4 paragraphes can be better.
Response: Thank you for your suggestion. We acknowledge that the current introduction may be too lengthy and appreciate your suggestion to condense it into 3-4

Powered by Editorial Manager® and ProduXion Manager® from Aries Systems Corporation

	focused paragraphs.<br><br>3.In methods section, data including flow chart should be added, and please learn some from the below articles:<br>a. Possible non-linear relation between prostate specific antigen and vitamin D: a machine learning study based on cross-section data<br>b. Association between urinary metals and prostate-specific antigen in aging population with depression: a cross-sectional study<br>Response: We thank the reviewer for your constructive suggestion. We have supplemented a flowchart that outline the participant selection process as Figure 1 in revised manuscript.<br><br>4.In the discussion section, this study should declare the mechanism in the increase of 25(OH)D acclerate aging.<br>Response: Thank you for your suggestion. We have rewritten the discussion section, providing the mechanism how the increase of 25(OH)D accelerate aging. “These detrimental effects may stem from the toxicity of excessive vitamin D. When vitamin D intake is too high, serum levels of its metabolites (25(OH)D and 24,25$(OH)_2$D) rise and surpass the binding capacity of vitamin D binding protein. This leads to an increase in the free active metabolite, 1,25$(OH)_2$D. This active form reaches the vitamin D receptor (VDR) in the nucleus, promoting gene expression that enhances calcium absorption and bone mobilization. Consequently, this can result in hypercalcemia or hyperphosphatemia, which in turn suppresses initial parathyroid hormone (iPTH) levels. These disrupting balance of calcium and hormone may lead to conditions such as cardiovascular disease, kidney damage, and skin aging. Research reported that the working diagnosis of vitamin D toxicity was determined based on total concentrations of 25(OH)D at 32~80ng/mL (80~200 nmol/L) detected by immunoassay. Our results found the 25(OH)D levels above125 nmol/L in female participants were linked to accelerated physiological aging, approximately consistent with those reports.” (marked in blue, Page 12)<br><br>5.Except the increase of 25(OH)D acclerate aging, if there are some literatures reporting aging can increase 25(OH)D. Maybe it is hard to explain the causality of the interactive association between 25(OH)D and aging. Please make this part clear.<br>Response: The relationship between 25(OH)D levels and aging is complex and bidirectional, making it challenging to establish a clear cause-and-effect relationship in our cross-sectional study. It is widely accepted that the level of vitamin D decrease as people get old, and people grow awareness of vitamin D deficiency and related health problems, vitamin D became a popular supplement, and its use has increased markedly. However, an increased intake of vitamin D supplements by the general population and a growing number of prescriptions of therapeutic doses without medical monitoring might result in a greater risk of excessive vitamin D toxicity. We have added some words to make this part clear in the discussion section. Thank you so much for your suggestion.<br><br>6.Language should be revised. Like in conclusion, "These relationships exhibited nonlinearity in both males and females.". It is not clear between what.<br>Response: Thank you for your valuable feedback and suggestions for improvement. I acknowledge the need for language editing to enhance the clarity, precision, and professionalism of the manuscript. I will thoroughly review and ask help from professional language improvement to ensure that the text is clear.<br><br>Reference<br>1.World Medical A. World Medical Association Declaration of Helsinki: Ethical Principles for Medical Research Involving Human Participants. JAMA. 2025;333(1):71-4. Epub 2024/10/19 20:42. doi: 10.1001/jama.2024.21972. PubMed PMID: 39425955.<br>2.NHANES Data Access Policy [cited 2025 feb 25]. Available from: https://www.cdc.gov/nchs/policy/data-user-agreement.html.
**Additional Information:**	
**Question**	**Response**
**Financial Disclosure**	The author(s) received no specific funding for this work.

Enter a financial disclosure statement that describes the sources of funding for the work included in this submission. Review the submission guidelines for detailed requirements. View published research articles from *PLOS ONE* for specific examples.<br><br>This statement is required for submission and **will appear in the published article** if the submission is accepted. Please make sure it is accurate.<br><br>**Funded studies**<br>Enter a statement with the following details:<br>• Initials of the authors who received each award<br>• Grant numbers awarded to each author<br>• The full name of each funder<br>• URL of each funder website<br>• Did the sponsors or funders play any role in the study design, data collection and analysis, decision to publish, or preparation of the manuscript?<br><br>Did you receive funding for this work?	
**Competing Interests**<br><br>Use the instructions below to enter a competing interest statement for this submission. On behalf of all authors, disclose any competing interests that could be perceived to bias this work—acknowledging all financial support and any other relevant financial or non-financial competing interests.<br><br>This statement is required for submission and **will appear in the published article** if the submission is accepted. Please make sure it is accurate and that any funding sources listed in your Funding Information later in the submission form are also declared in your Financial Disclosure statement.<br><br>View published research articles from	The authors have declared that no competing interests exist.

PLOS ONE for specific examples.<br><br>**NO authors have competing interests**<br><br>Enter: *The authors have declared that no competing interests exist.*<br><br>**Authors with competing interests**<br><br>Enter competing interest details beginning with this statement:<br><br>*I have read the journal's policy and the authors of this manuscript have the following competing interests: [insert competing interests here]*<br><br>* typeset	
**Ethics Statement**<br><br>Enter an ethics statement for this submission. This statement is required if the study involved:<br><br>• Human participants<br>• Human specimens or tissue<br>• Vertebrate animals or cephalopods<br>• Vertebrate embryos or tissues<br>• Field research<br><br>Write "N/A" if the submission does not require an ethics statement.<br><br>General guidance is provided below. Consult the submission guidelines for detailed instructions. **Make sure that all information entered here is included in the Methods section of the manuscript.**	the study involves human participants. all data from NHANES, which is a publicly available dataset managed by the Centers for Disease Control and Prevention. The ethical approval of NHANES has been stated on the website. (https://wwwn.cdc.gov/nchs/nhanes/Default.aspx)

**Format for specific study types**<br><br>**Human Subject Research (involving human participants and/or tissue)**<br>• Give the name of the institutional review board or ethics committee that approved the study<br>• Include the approval number and/or a statement indicating approval of this research<br>• Indicate the form of consent obtained (written/oral) or the reason that consent was not obtained (e.g. the data were analyzed anonymously)<br><br>**Animal Research (involving vertebrate animals, embryos or tissues)**<br>• Provide the name of the Institutional Animal Care and Use Committee (IACUC) or other relevant ethics board that reviewed the study protocol, and indicate whether they approved this research or granted a formal waiver of ethical approval<br>• Include an approval number if one was obtained<br>• If the study involved *non-human primates*, add *additional details* about animal welfare and steps taken to ameliorate suffering<br>• If anesthesia, euthanasia, or any kind of animal sacrifice is part of the study, include briefly which substances and/or methods were applied<br><br>**Field Research**<br><br>Include the following details if this study involves the collection of plant, animal, or other materials from a natural setting:<br>• Field permit number<br>• Name of the institution or relevant body that granted permission	
**Data Availability**<br><br>Authors are required to make all data underlying the findings described fully available, without restriction, and from the time of publication. PLOS allows rare exceptions to address legal and ethical concerns. See the PLOS Data Policy and FAQ for detailed information.	Yes - all data are fully available without restriction

A Data Availability Statement describing where the data can be found is required at submission. Your answers to this question constitute the Data Availability Statement and **will be published in the article**, if accepted.<br><br>**Important:** Stating 'data available on request from the author' is not sufficient. If your data are only available upon request, select 'No' for the first question and explain your exceptional situation in the text box.<br><br>Do the authors confirm that all data underlying the findings described in their manuscript are fully available without restriction?	
**Describe where the data may be found in full sentences. If you are copying our sample text, replace any instances of XXX with the appropriate details.**<br><br>• If the data are **held or will be held in a public repository**, include URLs, accession numbers or DOIs. If this information will only be available after acceptance, indicate this by ticking the box below. For example: *All XXX files are available from the XXX database (accession number(s) XXX, XXX.*).<br>• If the data are all contained **within the manuscript and/or Supporting Information files**, enter the following: *All relevant data are within the manuscript and its Supporting Information files.*<br>• If neither of these applies but you are able to provide **details of access elsewhere**, with or without limitations, please do so. For example:<br><br>*Data cannot be shared publicly because of [XXX]. Data are available from the XXX Institutional Data Access / Ethics Committee (contact via XXX) for researchers who meet the criteria for access to confidential data.*<br><br>*The data underlying the results presented in the study are available from (include the name of the third party*	All files are available from the NHANES database

and contact information or URL).<br>• This text is appropriate if the data are owned by a third party and authors do not have permission to share the data.<br><br>* typeset	
Additional data availability information:	

Powered by Editorial Manager® and ProduXion Manager® from Aries Systems Corporation

Dear Editors:

As we provide funding information in the Acknowledgments section, but this is not currently declared in my Funding Statement. We have removed the funding information in the manuscript, and would like to update our Funding Statement as “This work was supported by Special Clinical Research Project of Shanghai Municipal Health Commission [20224Y0335]”. Though the funding information has been filled, it is still not shown in the final PDF file. Could you help us change the online submission form ? thank you very much.

As my ORCiD iD was not validated in Editorial Manager account, I have registered in the website and got my ORCiD iD (0009-0002-0016-7591), which can be seen in my Editorial Manager account.

We deeply appreciate your advices. If you have any queries, please don’t hesitate to contact me. Thank you and best regards.

Yours sincerely

Corresponding author: Jue Wang

Mar, 2025

Manuscript Click here to access/download;Manuscript;revised manuscript clean.docx

Association of serum 25(OH)D level and physiological aging: A Cross-Sectional Study of NHANES 2007-2016

Yi Feng[a]; Licheng Yu[b]; Jue Wang[a] *

[a] Nanhui new town Community Health Service Center, Pudong New Area, Shanghai, 201308, China.

[b] Prevention and Health Care Department, Heqing Community Health Service Center of Pudong New District, Shanghai, 201201, China.

Corresponding author. Jue Wang

E-mail address: wangjue180130@126.com

Abstract

Vitamin D plays a crucial role in various aspects of human body, including aging, but chronological age may not accurately reflect true physiological aging status. Recently, PhenoAge has been developed to estimate an individual's biological age based on different physiological and clinical measures. Therefore, we investigated the relationship between serum 25(OH)D levels and physiological aging calculated by PhenoAge in the National Health and Nutrition Examination Survey (NHANES). Biological age showed a negative correlation with serum 25(OH)D levels (β=-0.04; 95% CI: -0.08-0.00, p=0.039), but this correlation was only found to be dose-dependent for females (β=-0.07; 95% CI: -0.12-0.01, p=0.007), but not for males. Further analysis confirmed that these associations were nonlinear in both sexes, revealing a U-shaped curve for males and an L-shaped curve for females. Specifically, serum 25(OH)D levels were linked to decelerated aging at levels below 38.20 nmol/L in males (β=-0.013; 95% CI: -0.025--0.002, p=0.017) and 62.50 nmol/L in females (β=-0.007; 95% CI: -0.01--0.004, p<0.001). However, high serum 25(OH)D levels (>125 nmol/L) did not correlate with aging in females (β=-0.001; 95% CI: -0.005-0.002), while in males, elevated levels (>91.55 nmol/L) were associated with accelerated aging (β=0.005; 95% CI: 0.002-0.008, p<0.001). Our findings indicate that vitamin D deficiency shows a potential link to accelerated physiological aging, but high levels of vitamin D do not link to decelerated physiological aging, offering valuable insights into the relationship between vitamin D and aging.

Key words: Vitamin D; Serum 25(OH)D; Physiological aging; PhenoAge; Nonlinear correlation

1. Introduction

Vitamin D is a fat-soluble vitamin that plays a crucial role in various aspects of human health, with its effects extending to bone metabolism, immune function, and overall physiological well-being. It is acquired through two primary sources: endogenous production (mainly vitamin D2) in the skin upon exposure to ultraviolet B (UVB), and exogenous intake (vitamin D2 and D3) through dietary sources, such as fatty fish, egg yolks or supplements. Once Vitamin D is obtained, vitamin D can be converted into 25-hydroxyvitamin D [25(OH)D] in the liver, then transformed into the biologically active form, 1,25-dihydroxyvitamin D [1,25(OH)$_2$D], in the kidneys. This active form binds to the vitamin D recep[illegible] (VDR), regulating calcium and phosphate metabolism [1, 2].

The measurement of serum 25(OH)D levels is critical for evaluating vitamin D status [3], but can be influenced by various factors, such as geographic location, race and food intake [4]. According to established criteria, serum 25(OH)D levels below 20 ng/mL (50 nmol/L) are classified as deficient, while levels between 20-30 ng/mL (50-75 nmol/L) are considered insufficient [5-7]. Vitam[illegible] D deficiency is a global public health concern, associated with increased risk of type 2 diabetes (T2D), hypertension, multiple sclerosis, etc [8-10]. The prevalence of vitamin D deficiency is particularly high among the elderly, with [illegible]dies suggesting that it may accelerate chronological aging and contribute to age-related diseases such as cardiovascular disorders [11]. However, several large trials of vitamin D supplement[illegible]n have failed to demonstrate significant benefits on cardiovascular disease, casting doubt on the observational evidence [12, 13]. This ambiguity highlights the complexity of the association between vitamin D and aging. Previous studies have a limitation of reliance on chronological age, which does not account for individual differences in physiological aging. While chronological age measures the passage of time, physiological aging reflects the rate of physiological decline, which can vary significantly among individuals due to genetic, environmental, and lifestyle factors [14]. Some individuals may age biologically faster or slower than their chronological age underscoring the need for more accurate biomarkers of aging.

To address this challenge, several methods have been created, including clinical

biomarkers-based approaches (PhenoAge, GrimAge etc) and methylation-based algorithms (DNAm PhenoAge acceleration, mortality risk score.etc) [15]. Among these, the PhenoAge method has emerged as a promising tool, employs a machine learning algorithm to estimate an individual's biological age based on various physiological and clinical measure, including inflammation (e.g. C-reactive protein), metabolic health (e.g. glucose, albumin), and organ function (e.g. alkaline phosphatase, lymphocyte percent) [16]. This method was developed by using data from the National Health and Nutrition Examination Survey (NHANES) and over 4,000 adults aged 20-84 years with complete biomarker data were used to train and validate the machine learning model [17]. PhenoAge demonstrates high accuracy, with a mean absolute error of approximately 3.6 years in predicting chronological age [16]. In a three-year longitudinal study, DO-HEALTH trial, involving 777 older adults, vitamin D supplement slows PhenoAge index, but not the other physiological aging index, including GrimAge, GrimAge2 and DunedinPACE [18]. At baseline, the mean 25(OH)D level among participants was 22.69 ng/mL, which falls within the insufficient range (20-30 ng/mL), proving that vitamin D supplement is beneficial for the elderly with vitamin D deficiency. However, the comprehensive picture of the association between vitamin D levels and physiological aging remains to be fully elucidated, particularly in the general population without vitamin D deficiency or insufficiency.

In this study, the association of serum 25(OH)D and physiological aging calculated by PhenoAge method was investigated in the NHANES data from 2007-2016. By exploring this relationship, the study aims to enhance our understanding of the potential link between vitamin D and aging, offering valuable insights into the underlying mechanisms and implications for public health.

2. Method

2.1 Study design and population

This study was conducted and reported in adherence to STROBE guidelines for observational studies. This study utilized data derived from National Health and Nutrition Examination Survey (NHANES), a nationwide survey conducted by the

National Center for Health Statistics (NCHS) of the Centers for Disease Control and Prevention (CDC). All data included in current study are publicly available through the NHANES website [19]. NHANES includes in-home interview, physical measurement and laboratory tests. Written consents were provided by all participants at recruitment. This study obtained 50588 individuals of NHANES data from 2007-2016. We excluded participants with incomplete information and participants aged below 40 years old. A total of 13,010 participants were included for further analysis (Figure 1).

2.2 Measurement of serum 25(OH)D concentrations

Serum 25-hydroxyvitamin D [25(OH)D] concentrations were measured as a reliable index to reflect vitamin D status. From cycle 2007-2008 and beyond, liquid chromatography-tandem mass spectrometry (LC-MS/MS) was used to measure serum 25(OH)D concentrations, including 25(OH)D3, epi-25(OH)D3, and 25(OH)D2 in human serum. We calculated the 25(OH)D levels by adding 25(OH)D3 and 25(OH)D2 levels. According to the American Endocrine Society guidelines, we transformed serum 25(OH)D concentration into categorical variables: deficiency (<50 nmol/L), insufficiency (50–75 nmol/L) and sufficiency (≥75 nmol/L) [20].

2.3 Measurement of physiological aging

In this study, we utilized PhenoAge as the indicator of aging, considering its high robustness and feasibility in NHANES data as a reliable predictor of mortality and age-related disease risks. The PhenoAge algorithm is constructed using NHANES III (1988–1994) by elastic-net regression, encompasses 10 markers: albumin, creatinine, glucose, white blood cell count, lymphocyte percent, red cell distribution width, mean red cell volume, C-reactive protein, alkaline phosphatase and chronological age [21]. The R package BioAge was used to compute PhenoAge [16]. One of the markers, C-reactive protein, was unavailable from 2011 to 2018. According to previous studies, there is a strong correlation (correlation coefficient of 0.99) between PhenoAge measurements with and without C-reactive protein, utilizing an algorithm that excludes

this marker to calculate PhenoAge [22, 23]. Due to the absence of C-reactive protein data, we employed PhenoAge algorithm that excludes this marker in the current study. We further calculated physiological aging as the difference between PhenoAge and chronological age. A positive value indicates accelerated aging, while a negative value indicates slower aging. Subsequently, physiological aging was standardized to have a mean of 0 and a standard deviation (SD) of 1.

2.4 Covariates

NHANES interviews were conducted by trained interviewers, where questionnaires were administered to collect information on demographics [age, gender, race, education level, poverty income ratio (PIR)] and lifestyle (smoking and drinking status, physical activity and comorbidities). Body mass index (BMI) were measured at the Mobile Examination Center.

Smoking status was classified as never smokers (had smoked <100 cigarettes in their lifetime), current smokers (had smoked ≥100 cigarettes in their lifetime and still smoked at the time of the interview) and former smokers (had smoked ≥100 cigarettes in their lifetime but had quit smoking at the time of the survey). Drinking status was classified as never drinkers (had drunk <12 drinks in their lifetime), current drinkers (had drunk ≥12 drinks in their lifetime and at least one drink in the past year) and former drinkers (had drunk ≥12 drinks in their lifetime but no drink in the past year). Metabolic equivalent task (MET)-min per week were calculated to assess physical activity, and levels of physical activity were categorized as: inactive (no regular physical activity), insufficient (<500 MET-min activity/week), moderate (500-1,000 MET-min activity/week) and high (≥1,000 MET-min activity/week). For sleep duration, participants responded to the question: "How much sleep do you usually get at night on weekdays or workdays?" and were categorized into 3 groups: <7 h, 7-8 h and ≥8h). Hypertension was ascertained by self-reporting history of hypertension, use

of medication for hypertension or physical examination (systolic blood pressure ≥140 mmHg or diastolic blood pressure ≥ 90 mmHg). Diabetes was ascertained by self-reporting history of diabetes or results from laboratory examination (fasting glucose level ≥126 mg/dL or glycohemoglobin ≥6.5%).

2.5 Statistical analysis

Continuous data were reported as mean ± standard deviation (SD) or median (interquartile range). Categorical data were reported as numbers (percentage). Weighted multivariable linear regression models were constructed to estimate the association between serum 25(OH)D status and standardized physiological aging. Serum 25(OH)D level were log-transformed due to skewed distribution. To evaluate potential dose-response patterns, 25(OH)D level was analyzed both as a continuous variable (log-transformed) and categorically through quartiles and four clinically defined sufficiency categories. Beta coefficients with 95% confidence intervals (CIs) were reported, representing the change in dependent variable per unit increase in the independent variable. The use of beta coefficients provides interpretable estimates for linear associations when analyzing continuous dependent variables, which is widely recommended in epidemiological studies [24]. Correlation analysis was not employed because it does not account for confounding factors, whereas the primary aim of current study is to estimate the confounder-adjusted association between 25(OH)D status and physiological aging. All models were adjusted for age, race, education level, PIR, BMI, smoking, drinking, physical activity, sleep duration, hypertension, diabetes and season of measurement.

To explore the non-linear relationship between serum 25(OH)D and physiological aging, generalized additive model (GAM) was employed to fit the smooth curve. The threshold effect was further estimated using segmented regression model. A *P*-value of <0.05 was considered statistical significance. All data analyses were performed using R version 4.2.1 (R Core Team, Vienna, Austria). R package ‘BioAge’ was used to calculate PhenoAge [16], while package ‘mgcv’ was used to conduct GAM analysis

[25].

2.6 Ethical considerations

This study utilized data from NHANES study, which was conducted in accordance with the principles in the Declaration of Helsinki. The NHANES protocol received prior ethical approval from the National Center for Health Statistics' Review Board. Since our study is a secondary analysis of publicly accessible, de-identified NHANES data sets, additional institutional review board approval was not required [26, 27].

3. Results

3.1 Characteristics of the study population

The current study included a total of 6836 males and 7074 females, with the characteristics of the study population presented in Table 1. Significant differences between males and females in the distribution of basic information, such as age, race, BMI, and poverty-income ratio ($p < 0.001$), except for education level. Additionally, gender-specific differences were noted in behaviors influencing aging, such as smoking, drinking, physical activity, and sleep duration, were also observed between males and females ($p < 0.001$). Notably, males had a higher prevalence of diabetes than females. The median serum 25(OH)D level in females was higher (66.60 [illegible]ol/L) than in males (63.80 nmol/L), and the PhenoAge calculated from 10 clinical biomarkers was lower in females than in males. Furthermore, the physiological aging was also lower in females compared to males ($p < 0.001$).

3.2 Association of se[illegible]um 25(OH)D level and physiological aging

The associations between serum 25(OH)D levels and physiological aging was analyzed and presented in Table 2. After adjusting for age, race, education level, PIR, BMI, smoking, drinking, physical activity, sleep duration, hypertension, and diabetes, physiological aging was found to be negatively associated with serum 25(OH)D levels (β=-0.04; 95% CI: -0.08-0.0[illegible]). However, this association was statistically significant in females (p=0.007), and not in males (p=0.334). To further explore this relationship,

the population was categorized into four groups based on quartiles for analysis. Higher serum 25(OH)D levels were inversely associated with physiological aging, with no apparent dose-dependent trend as the p-value for trend was not significant (p=0.173). This pattern was consistent in females but absent in males. Furthermore, the population was categorized into four groups according to the endocrine society guidelines, which similarly revealed that higher serum 25(OH)D levels were associated with reduced physiological aging, albeit without dose-dependent relationships (p=0.076) in whole population and females. Interestingly, moderate level of 25(OH)D levels (30-50 and 50-75 nmol/L) were associated with reduced physiological aging in males categorized based on endocrine society guidelines, though these statistical differences were not observed in male based on quartile categories.

Stratified analysis was conducted to further investigate the impact of various factors on the relationship between serum 25(OH)D levels and physiological aging. In males, no overall association was observed; however, in male subpopulations over 60 years of age with hypertension or diabetes, higher serum 25(OH)D levels were associated with slower physiological aging. Additionally, there were statistically significant interactions between serum 25(OH)D levels and age, hypertension, or diabetes (Fig.2A). In contrast, in females, factors such as age, BMI, smoking, drinking, physical activity, and hypertension did not alter the inverse association between them, indicating the robustness of this relationship in females (Fig.2B).

3.3 Non-linear correlation between serum 25(OH)D level and physiological aging

Given the non-significant trend in Table 2, indicating a non-linear correlation, a generalized additive model (GAM) was utilized to investigate the potential link. The analysis revealed a nonlinear relationship between serum 25(OH)D level and standardized physiological aging in both males and females (p<0.001) (Fig 3 A-B). In males, the relationship exhibited a U shape, while in females, it resembled more of like an L shape, as confirmed by segmented regression modeling. Two inflection points were identified in males at 38.2 nmol/L and 91.55 nmol/L. Low serum 25(OH)D levels (<38.2 nmol/L) were negatively correlated with standardized physiological aging (β=-

0.013; 95% CI: -0.025--0.002), whereas high levels (>91.55 nmol/L) showed a positive association (β=0.005; 95% CI: 0.002-0.008). For females, serum 25(OH)D levels below 62.5 nmol/L were negatively linked to physiological aging (β=-0.007; 95% CI: -0.01--0.004), with no significant association observed beyond 125 nmol/L (β=-0.001; 95% CI: -0.005-0.002). Overall, low (<38.2 nmol/L) or high (>91.55 nmol/L) serum 25(OH)D levels in males were associated with accelerating aging, while in females, only low levels (<62.5 nmol/L) pose a risk factor for aging (Table 3).

4. Discussion

In the current study, the association between serum 25(OH)D level and physiological aging were investigated in NHANES. The analysis revealed a negative correlation between physiological age and serum 25(OH)D level, whether categorized by quartiles or accor[illegible] to the endocrine society's criteria. This correlation exhibited a dose-dependent relationship in females but not in the overall population or males. Further analysis confirmed these associations were nonlinear in both genders, b with a U-shaped curve in males and an L-shaped curve in females.

Numerous studies have established that serum 25(OH)D level, representing vitamin D status in our body, are associated with various diseases or disorders, including all-cause mortality, cardiovascular diseases, and aging etc. Multiple large-scale observational studies have consistently shown a negative correlation between serum 25(OH)D levels and all-cause mortality risk, lower serum 25(OH)D levels are associated with an increased risk of all-cause mortality, and a dose-response relationship observed [28, 29]. Individuals with lower vitamin D levels (typically below 30 ng/mL) linked to a higher risk of all-cause mortality compared to those with adequate vitamin D status [30]. Regarding aging, studies have reported that a gradual decline in serum 25(OH)D levels with chronological age [31]. This decline is attributed to several factors, including decreased sun exposure, reduced capacity of the skin to synthesize vitamin D, decreased dietary intake of vitamin D, and impaired renal function, resulting in vitamin D deficiency in older adults [32]. Additionally, we also found that low serum 25(OH)D levels were associated with increased physiological

aging, suggesting vitamin D may also play a crucial role in the regulation of the aging process. Adequate 25(OH)D levels are essential for maintaining calcium absorption and bone health preventing osteoporosis, a common age-related condition, and regulating calcium homeostasis and bone metabolism [33]. Moreover, vitamin D can interact with antioxidant and anti-inflammatory pathways, such as activating Nrf2 (nuclear factor erythroid 2-related factor 2) and SIRT1 (sirtuin 1) signaling cascades, and suppressing the production of pro-inflammatory cytokines, including interleukin-6 (IL-6) and tumor necrosis factor-alpha (TNF-α), through inhibiting nuclear factor-κB (NF-κB). These actions lead to reduction of inflammation and oxidative stress, both of which are known to contribute to the aging process [33, 34]. Therefore, vitamin D and aging have a mutual influence on each other.

The significant association between vitamin D and ~~aging~~ suggests that vitamin D supplementation could be an effective way to reverse or mitigate various diseases and disorders. Vitamin D supplementation may help lower blood pressure and improve endothelial function, leading to reduced risk of cardiovascular events, such as heart attacks and strokes, and improved cardiovascular outcomes [35]. A meta-analysis of 75 randomized controlled trials in 2014 revealed that vitamin D supplementation was associated with a 6% reduction in all-cause mortality [36]. Some studies suggest that the impact of vitamin D supplementation on all-cause mortality may be more pronounced in certain subgroups, such as older adults or individuals with vitamin D deficiency. For example, vitamin D supplementation was associated with a reduced risk of all-cause mortality in trials with a mean baseline 25(OH)D level <30 ng/mL (RR 0.89, 95% CI 0.80-0.99), but not in trials with a mean baseline 25(OH)D level ≥30 ng/mL (RR 1.04, 95% CI 0.97-1.11) [37]. Thus, the growing body of evidence highlights the potential of vitamin D supplementation in promoting overall health and reducing the risk of various chronic diseases, particularly in elderly people, where serum vitamin D levels tend to decline with age.

Despite its potential benefits, the use of vitamin D supplements has increased markedly, and concerns about excessive intake have emerged. the Vitamin D and

Omega-3 Trial (VITAL) study, a randomized trial involving over 25,000 participants, discovered that vitamin D supplementation did not significantly reduce the risk of all-cause mortality or lower risk of fractures among generally healthy midlife and older adults without vitamin D deficiency or osteoporosis.[38]. Furthermore, increased intake of vitamin D supplements by the general population and a growing number of high-dose prescriptions without medical monitoring might result in a greater risk of excessive vitamin D toxicity[39]. Our current results indicated that the correlation of serum 25(OH)D level and physiological aging followed a U-shaped curve in males and an L-shaped curve in females, revealing that high serum level of 25(OH)D is not associated with decelerated aging in either males and females, and even associated with accelerated physiological aging in males. These detrimental effects may stem from the toxicity of excessive vitamin D. When vitamin D intake is too high, serum levels of its metabolites [25(OH)D and 24,25$(OH)_2$D] rise beyond the binding capacity of vitamin D binding protein, leading to an increase in the free active metabolite, 1,25$(OH)_2$D. This active form reaches the VDR in the nucleus, constantly promoting gene expression that enhances calcium absorption and bone mobilization. Consequently, this can result in hypercalcemia or hyperphosphatemia, which in turn suppresses initial parathyroid hormone (iPTH) level [40, 41]. Such disrupting balance of calcium and hormone may contribute to cardiovascular disease, kidney damage, and skin aging. Research reported that the working diagnosis of vitamin D toxicity is diagnosed based on total 25(OH)D concentrations of 32~80ng/mL (80~200 nmol/L) detected by immunoassay [42]. Our results found the 25(OH)D levels over 125 nmol/L in female participants were linked to accelerated physiological aging, approximately consistent with those reports.

Our results reported differences of dose-response curve between in male and in female, which may be attributed to the sex hormones, especially estrogen. As women age, particularly during the menopausal transition and after menopause, their ovaries produce less estrogen, leading to a decline of estrogen levels. This decline contributes to the thinning and wrinkling of the skin, as well as changes in hair texture and growth patterns [43]. Some studies suggest that vitamin D could enhance the activity of estrogen receptors (ER) via interacting with them, potentially amplifying the anti-aging

effects of estrogen in the body [44]. Vitamin D and estrogen also have a synergistic effect on bone metabolism, helping to prevent osteoporosis and fractures, especially in postmenopausal women [45]. Additionally, vitamin D regulate estrogen metabolism, leading to higher circulating estrogen levels and subsequently delay aging [44]. Therefore, high level of serum 25(OH)D may not be associated with physiological aging in females, but the mechanisms underlying the adverse effects of extremely high levels (>100 nmol/L) in males remain unclear. Further studies are needed to clarify the complex interplay between these two important hormonal systems.

The current study has several limitations. First, this cross-sectional study only provide information on the association of serum 25(OH)D level and physiological aging, making it difficult to establish causal relationships. Second, we applied PhenoAge method to calculate physiological aging, but the data of C-reactive protein were not available. Finally, the gender-specific differences in results require further detailed analysis to fully understand the underlying mechanisms.

5. Conclusion

In this study, we investigated the relationship between serum 25(OH)D levels and biological age in NHANES, and found that there was a negative correlation between physiological age and serum 25(OH)D level, and these associations were nonlinear in both genders, revealing a U-shaped curve for males and an L-shaped curve for females. Low serum 25(OH)D levels shows a potential link to accelerated physiological aging in both genders, but high serum 25(OH)D levels do not correlate with decelerated physiological aging, and even associated with accelerated physiological aging in male. Our results suggest that the association between vitamin D levels with physiological aging is complex and whether vitamin D supplementation in individuals with sufficient 25(OH)D levels is beneficial warrants further investigation.

Contributions

Yi Feng: Conceptualization; Methodology; Data curation; Formal analysis;

Writing original draft**; Licheng Yu**: Data curation; Formal analysis; **Jue Wang**: Project administration; Supervision; Writing - review &editing.

Conflict of interest

The authors declare no competing financial or non-financial interests related to this work. NHANES is a publicly available dataset managed by the Centers for Disease Control and Prevention. The authors confirm that there is no affiliation with or involvement from the NHANES program administrators in this study. The funding source had no role in study design, data collection, analysis, or manuscript preparation.

Reference

1. Dominguez LJ, Farruggia M, Veronese N, Barbagallo M. Vitamin D Sources, Metabolism, and Deficiency: Available Compounds and Guidelines for Its Treatment. Metabolites. 2021;11(4). Epub 2021/05/01. doi: 10.3390/metabo11040255. PubMed PMID: 33924215; PubMed Central PMCID: PMCPMC8074587.
2. Bikle D, Christakos S. New aspects of vitamin D metabolism and action - addressing the skin as source and target. Nat Rev Endocrinol. 2020;16(4):234-52. Epub 2020/02/08. doi: 10.1038/s41574-019-0312-5. PubMed PMID: 32029884.
3. Couchman L, Moniz CF. Analytical considerations for the biochemical assessment of vitamin D status. Ther Adv Musculoskelet Dis. 2017;9(4):97-104. Epub 2017/04/07. doi: 10.1177/1759720X17692500. PubMed PMID: 28382113; PubMed Central PMCID: PMCPMC5367642.
4. Rahman A, Elmi A. Air pollutants are negatively associated with vitamin D-synthesizing UVB radiation intensity on the ground. Sci Rep. 2021;11(1):21480. Epub 2021/11/04. doi: 10.1038/s41598-021-00980-6. PubMed PMID: 34728744; PubMed Central PMCID: PMCPMC8563978.
5. Bordelon P, Ghetu MV, Langan RC. Recognition and management of vitamin D deficiency. Am Fam Physician. 2009;80(8):841-6. Epub 2009/10/20. PubMed PMID: 19835345.
6. Cashman KD. Vitamin D Deficiency: Defining, Prevalence, Causes, and Strategies of Addressing. Calcif Tissue Int. 2020;106(1):14-29. Epub 2019/05/10. doi: 10.1007/s00223-019-00559-4. PubMed PMID: 31069443.
7. Demay MB, Pittas AG, Bikle DD, Diab DL, Kiely ME, Lazaretti-Castro M, et al. Vitamin D for the Prevention of Disease: An Endocrine Society Clinical Practice Guideline. J Clin Endocrinol Metab. 2024. Epub 2024/06/03. doi: 10.1210/clinem/dgae290. PubMed PMID: 38828931.
8. Zhao S, Qian F, Wan Z, Chen X, Pan A, Liu G. Vitamin D and major chronic diseases. Trends Endocrinol Metab. 2024. Epub 2024/06/02. doi: 10.1016/j.tem.2024.04.018. PubMed PMID: 38824035.
9. Gaksch M, Jorde R, Grimnes G, Joakimsen R, Schirmer H, Wilsgaard T, et al. Vitamin D and mortality: Individual participant data meta-analysis of standardized 25-hydroxyvitamin D in 26916

individuals from a European consortium. Plos One. 2017;12(2):e0170791. Epub 2017/02/17. doi: 10.1371/journal.pone.0170791. PubMed PMID: 28207791; PubMed Central PMCID: PMCPMC5312926.
10. Fang A, Zhao Y, Yang P, Zhang X, Giovannucci EL. Vitamin D and human health: evidence from Mendelian randomization studies. Eur J Epidemiol. 2024;39(5):467-90. Epub 2024/01/12. doi: 10.1007/s10654-023-01075-4. PubMed PMID: 38214845.
11. Berridge MJ. Vitamin D deficiency accelerates ageing and age-related diseases: a novel hypothesis. The Journal of physiology. 2017;595(22):6825-36. Epub 2017/09/28. doi: 10.1113/JP274887. PubMed PMID: 28949008; PubMed Central PMCID: PMCPMC5685827.
12. Zittermann A, Ernst JB, Prokop S, Fuchs U, Dreier J, Kuhn J, et al. Effect of vitamin D on all-cause mortality in heart failure (EVITA): a 3-year randomized clinical trial with 4000 IU vitamin D daily. Eur Heart J. 2017;38(29):2279-86. Epub 2017/05/13. doi: 10.1093/eurheartj/ehx235. PubMed PMID: 28498942.
13. Scragg R, Stewart AW, Waayer D, Lawes CMM, Toop L, Sluyter J, et al. Effect of Monthly High-Dose Vitamin D Supplementation on Cardiovascular Disease in the Vitamin D Assessment Study : A Randomized Clinical Trial. JAMA Cardiol. 2017;2(6):608-16. Epub 2017/04/07. doi: 10.1001/jamacardio.2017.0175. PubMed PMID: 28384800; PubMed Central PMCID: PMCPMC5815022 ICMJE Form of Disclosure of Potential Conflicts of Interest and none were reported.
14. Xia X, Wang Y, Yu Z, Chen J, Han JJ. Assessing the rate of aging to monitor aging itself. Ageing Res Rev. 2021;69:101350. Epub 2021/05/04. doi: 10.1016/j.arr.2021.101350. PubMed PMID: 33940202.
15. Jylhava J, Pedersen NL, Hagg S. Biological Age Predictors. EBioMedicine. 2017;21:29-36. Epub 2017/04/12. doi: 10.1016/j.ebiom.2017.03.046. PubMed PMID: 28396265; PubMed Central PMCID: PMCPMC5514388.
16. Kwon D, Belsky DW. A toolkit for quantification of biological age from blood chemistry and organ function test data: BioAge. Geroscience. 2021;43(6):2795-808. Epub 2021/11/03. doi: 10.1007/s11357-021-00480-5. PubMed PMID: 34725754; PubMed Central PMCID: PMCPMC8602613.
17. Bortz J, Guariglia A, Klaric L, Tang D, Ward P, Geer M, et al. Biological age estimation using circulating blood biomarkers. Commun Biol. 2023;6(1):1089. Epub 2023/10/27. doi: 10.1038/s42003-023-05456-z. PubMed PMID: 37884697; PubMed Central PMCID: PMCPMC10603148 manuscript, P.K.J. and J.B. were paid consultants to Humanity Inc, a company focussed on measuring and developing interventions for Biological Age. L.K. was an employee of Humanity Inc. A.G. was formerly a paid consultant of Humanity Inc. M.G. and P.W. are founders of Humanity Inc and are employees and hold ordinary shares. P.K.J., M.G. and P.W. are partly remunerated under a Humanity Inc share option scheme. P.K.J. is founder of Geromica, a consultancy providing advice on measurement of health and aging. M.C.-H. holds shares in the O-SMOSE company and has no conflict of interest to disclose. Consulting activities conducted by the company are independent of the present work. All other authors declare no competing interests.
18. Bischoff-Ferrari HA, Gangler S, Wieczorek M, Belsky DW, Ryan J, Kressig RW, et al. Individual and additive effects of vitamin D, omega-3 and exercise on DNA methylation clocks of biological aging in older adults from the DO-HEALTH trial. Nat Aging. 2025;5(3):376-85. Epub 2025/02/04. doi: 10.1038/s43587-024-00793-y. PubMed PMID: 39900648.
19. Centers for Disease Control and Prevention National Health and Nutrition Examination Survey Questionnaires D, and Related Documentation 2020. [cited 2025 March 7]. Available from: https://wwwn.cdc.gov/nchs/nhanes/.

20. Holick MF, Binkley NC, Bischoff-Ferrari HA, Gordon CM, Hanley DA, Heaney RP, et al. Evaluation, treatment, and prevention of vitamin D deficiency: an Endocrine Society clinical practice guideline. J Clin Endocrinol Metab. 2011;96(7):1911-30. Epub 2011/06/08. doi: 10.1210/jc.2011-0385. PubMed PMID: 21646368.
21. Liu Z, Kuo PL, Horvath S, Crimmins E, Ferrucci L, Levine M. A new aging measure captures morbidity and mortality risk across diverse subpopulations from NHANES IV: A cohort study. PLoS Med. 2018;15(12):e1002718. Epub 2019/01/01. doi: 10.1371/journal.pmed.1002718. PubMed PMID: 30596641; PubMed Central PMCID: PMCPMC6312200.
22. He H, Chen X, Ding Y, Chen X, He X. Composite dietary antioxidant index associated with delayed biological aging: a population-based study. Aging (Albany NY). 2024;16(1):15-27. Epub 2024/01/04. doi: 10.18632/aging.205232. PubMed PMID: 38170244; PubMed Central PMCID: PMCPMC10817368.
23. Liu W, Wang J, Wang M, Hou H, Ding X, Ma L, et al. Oxidative Stress Factors Mediate the Association Between Life's Essential 8 and Accelerated Phenotypic Aging: NHANES 2005-2018. J Gerontol A Biol Sci Med Sci. 2024;79(1). Epub 2023/10/10. doi: 10.1093/gerona/glad240. PubMed PMID: 37813096.
24. Bender R. Introduction to the use of regression models in epidemiology. Cancer epidemiology: Springer; 2009. p. 179-95.
25. Wood SN. Stable and efficient multiple smoothing parameter estimation for generalized additive models. Journal of the American Statistical Association. 2004;99(467):673-86.
26. Yucebilgic G, Bilgin R, Tamer L, Tukel S. Effects of lead on Na(+)-K(+) ATPase and Ca(+2) ATPase activities and lipid peroxidation in blood of workers. International journal of toxicology. 2003;22(2):95-7. doi: 10.1080/10915810305096. PubMed PMID: 12745990.
27. World Medical A. World Medical Association Declaration of Helsinki: Ethical Principles for Medical Research Involving Human Participants. JAMA. 2025;333(1):71-4. Epub 2024/10/19 20:42. doi: 10.1001/jama.2024.21972. PubMed PMID: 39425955.
28. Amrein K, Scherkl M, Hoffmann M, Neuwersch-Sommeregger S, Kostenberger M, Tmava Berisha A, et al. Vitamin D deficiency 2.0: an update on the current status worldwide. Eur J Clin Nutr. 2020;74(11):1498-513. Epub 2020/01/22. doi: 10.1038/s41430-020-0558-y. PubMed PMID: 31959942; PubMed Central PMCID: PMCPMC7091696 The other authors declare that they have no conflict of interest.
29. Liu W, Hu J, Fang Y, Wang P, Lu Y, Shen N. Vitamin D status in Mainland of China: A systematic review and meta-analysis. EClinicalMedicine. 2021;38:101017. Epub 2021/07/27. doi: 10.1016/j.eclinm.2021.101017. PubMed PMID: 34308318; PubMed Central PMCID: PMCPMC8283334.
30. Bouillon R, Manousaki D, Rosen C, Trajanoska K, Rivadeneira F, Richards JB. The health effects of vitamin D supplementation: evidence from human studies. Nat Rev Endocrinol. 2022;18(2):96-110. Epub 2021/11/25. doi: 10.1038/s41574-021-00593-z. PubMed PMID: 34815552; PubMed Central PMCID: PMCPMC8609267 Farma, Ceres and Proctor and Gamble. J.B.R. has worked as a consultant to GlaxoSmithKline and Deerfield Capital. The other authors declare no competing interests.
31. Schottker B, Hagen L, Zhang Y, Gao X, Holleczek B, Gao X, et al. Serum 25-Hydroxyvitamin D Levels as an Aging Marker: Strong Associations With Age and All-Cause Mortality Independent From Telomere Length, Epigenetic Age Acceleration, and 8-Isoprostane Levels. J Gerontol A Biol Sci Med Sci. 2019;74(1):121-8. Epub 2018/10/30. doi: 10.1093/gerona/gly253. PubMed PMID: 30371905.
32. Liu L, Chen C, Lo K, Huang J, Yu Y, Huang Y, et al. Serum 25-hydroxyvitamin D, frailty, and

mortality among the Chinese oldest old: Results from the CLHLS study. Nutr Metab Cardiovasc Dis. 2021;31(9):2707-15. Epub 2021/08/06. doi: 10.1016/j.numecd.2021.05.033. PubMed PMID: 34348876.

33. Veldurthy V, Wei R, Oz L, Dhawan P, Jeon YH, Christakos S. Vitamin D, calcium homeostasis and aging. Bone Res. 2016;4:16041. Epub 2016/10/30. doi: 10.1038/boneres.2016.41. PubMed PMID: 27790378; PubMed Central PMCID: PMCPMC5068478.

34. Ge D, Chen Q, Xie X, Li Q, Yang Y. Unveiling the potent effect of vitamin D: harnessing Nrf2/HO-1 signaling pathways as molecular targets to alleviate urban particulate matter-induced asthma inflammation. BMC Pulm Med. 2024;24(1):55. Epub 2024/01/26. doi: 10.1186/s12890-024-02869-2. PubMed PMID: 38273268; PubMed Central PMCID: PMCPMC10809564.

35. Pilz S, Gaksch M, Kienreich K, Grubler M, Verheyen N, Fahrleitner-Pammer A, et al. Effects of vitamin D on blood pressure and cardiovascular risk factors: a randomized controlled trial. Hypertension. 2015;65(6):1195-201. Epub 2015/03/25. doi: 10.1161/HYPERTENSIONAHA.115.05319. PubMed PMID: 25801871.

36. Chowdhury R, Kunutsor S, Vitezova A, Oliver-Williams C, Chowdhury S, Kiefte-de-Jong JC, et al. Vitamin D and risk of cause specific death: systematic review and meta-analysis of observational cohort and randomised intervention studies. BMJ. 2014;348:g1903. Epub 2014/04/03. doi: 10.1136/bmj.g1903. PubMed PMID: 24690623; PubMed Central PMCID: PMCPMC3972416 at www.icmje.org/coi_disclosure.pdf (available on request from the corresponding authors) and declare: no support from any organisation for the submitted work; no financial relationships with any organisations that might have an interest in the submitted work in the previous three years; no other relationships or activities that could appear to have influenced the submitted work.

37. Rejnmark L, Bislev LS, Cashman KD, Eiriksdottir G, Gaksch M, Grubler M, et al. Non-skeletal health effects of vitamin D supplementation: A systematic review on findings from meta-analyses summarizing trial data. Plos One. 2017;12(7):e0180512. Epub 2017/07/08. doi: 10.1371/journal.pone.0180512. PubMed PMID: 28686645; PubMed Central PMCID: PMCPMC5501555.

38. LeBoff MS, Chou SH, Ratliff KA, Cook NR, Khurana B, Kim E, et al. Supplemental Vitamin D and Incident Fractures in Midlife and Older Adults. N Engl J Med. 2022;387(4):299-309. Epub 2022/08/09. doi: 10.1056/NEJMoa2202106. PubMed PMID: 35939577; PubMed Central PMCID: PMCPMC9716639.

39. Pludowski P, Grant WB, Karras SN, Zittermann A, Pilz S. Vitamin D Supplementation: A Review of the Evidence Arguing for a Daily Dose of 2000 International Units (50 microg) of Vitamin D for Adults in the General Population. Nutrients. 2024;16(3). Epub 2024/02/10. doi: 10.3390/nu16030391. PubMed PMID: 38337676; PubMed Central PMCID: PMCPMC10857599.

40. Marcinowska-Suchowierska E, Kupisz-Urbanska M, Lukaszkiewicz J, Pludowski P, Jones G. Vitamin D Toxicity-A Clinical Perspective. Front Endocrinol (Lausanne). 2018;9:550. Epub 2018/10/09. doi: 10.3389/fendo.2018.00550. PubMed PMID: 30294301; PubMed Central PMCID: PMCPMC6158375.

41. Batman A, Saygili ES, Yildiz D, Sen EC, Erol RS, Canat MM, et al. Risk of hypercalcemia in patients with very high serum 25-OH vitamin D levels. Int J Clin Pract. 2021;75(7):e14181. Epub 2021/03/25. doi: 10.1111/ijcp.14181. PubMed PMID: 33759301; PubMed Central PMCID: PMCPMC8250214.

42. Sajid U, Orton D, Kaufmann M, Jones G, Kline GA. Understanding Elevated Vitamin D Measurements to Uncover Hypercalcemia Etiology. Clin Chem. 2024;70(6):798-802. Epub 2024/06/03.

doi: 10.1093/clinchem/hvae044. PubMed PMID: 38825340.

43. Santoro N, Roeca C, Peters BA, Neal-Perry G. The Menopause Transition: Signs, Symptoms, and Management Options. J Clin Endocrinol Metab. 2021;106(1):1-15. Epub 2020/10/24. doi: 10.1210/clinem/dgaa764. PubMed PMID: 33095879.

44. Crescioli C. The Role of Estrogens and Vitamin D in Cardiomyocyte Protection: A Female Perspective. Biomolecules. 2021;11(12). Epub 2021/12/25. doi: 10.3390/biom11121815. PubMed PMID: 34944459; PubMed Central PMCID: PMCPMC8699224.

45. McCurdy R, Jiang X, Clarkson TB, Nudy M, Schnatz PF. Vitamin D and conjugated equine estrogen: the association with coronary artery atherosclerosis in cynomolgus monkeys. Menopause. 2016;23(5):481-7. Epub 2016/02/18. doi: 10.1097/GME.0000000000000582. PubMed PMID: 26886883.

Table 1. Characteristics of the study population

Characteristics	Male (n=6836)	Female (n=7074)	*P*-value
Age (mean±SD)	57.07 (11.34)	58.12 (11.82)	<0.001
Race			0.001[b]
Non-Hispanic white	3249 (47.53)	3288 (46.48)	
Non-Hispanic black	1360 (19.89)	1417 (20.03)	
Others	2227 (32.58)	2369 (33.49)	
Education level			0.394[b]
Below high school	1847 (27.02)	1831 (25.88)	
High school or equivalent	1588 (23.23)	1597 (22.58)	
Above high school	3401 (49.75)	3646 (51.54)	
Poverty-income ratio			<0.001[b]
⩽1.0	1199 (17.54)	1447 (20.46)	
1.0-3.0	2839 (41.53)	3029 (42.82)	
>3.0	2798 (40.93)	2598 (36.73)	
BMI, kg/m^2			<0.001[b]
<25	1585 (23.19)	1841 (26.02)	
25-30	2749 (40.21)	2117 (29.93)	
⩾30	2502 (36.60)	3116 (44.05)	
Smoking			<0.001[b]
Never smoker	2823 (41.30)	4297 (60.74)	
Previous smoker	2570 (37.60)	1666 (23.55)	
Current smoker	1443 (21.11)	1111 (15.71)	
Drinking			<0.001[b]
Never drinker	527 (7.71)	1572 (22.22)	
Previous drinker	637 (9.32)	1466 (20.72)	
Current drinker	5672 (82.97)	4036 (57.05)	
Physical activity			<0.001[b]
Inactive	1775 (25.97)	2533 (35.81)	
Insufficient	802 (11.73)	1086 (15.35)	
Moderate	680 (9.95)	842 (11.90)	
High	3579 (52.36)	2613 (36.94)	
Sleep duration			<0.001[b]
<7 h	2572 (37.62)	2445 (34.56)	
7-8h	1858 (27.18)	1812 (25.61)	
⩾8 h	2406 (35.20)	2817 (39.82)	
Hypertension			0.839[b]

No	3007 (43.99)	3067 (43.36)	
Yes	3829 (56.01)	4007 (56.64)	
Diabetes			<0.001[b]
No	5086 (74.40)	5515 (77.96)	
Yes	1750 (25.60)	1559 (22.04)	
Serum 25(OH)D, nmol/L			
Median (IQR)	63.80 (48.38, 80.20)	66.60 (47.00, 87.30)	<0.001[c]
<30	420 (6.14)	545 (7.70)	<0.001[b]
30-50	1451 (21.23)	1457 (20.60)	
50-75	2766 (40.46)	2293 (32.41)	
⩾75	2199 (32.17)	2779 (39.28)	
Ph[illegible]age (median [IQR])	53.77 (45.07, 64.60)	53.05 (44.23, 64.13)	0.032
Physiological aging (median [IQR])	-2.40 (-5.13, 0.94)	-4.14 (-7.22, -0.46)	<0.001[c]
Standardized physiological aging (mean±SD)	0.11 (0.95)	-0.16 (1.05)	<0.001[a]

BMI: body mass index; IQR: interquartile range; SD: standard deviation

Data were reported as number (percentage) for categorical variables; mean±SD or median (IQR) for continuous variables.

[a] Student's t-test was used to compare means of variables between two groups

[b] Chi-square test was used to compare categorical variables between two groups.

[c] Mann-Whitney test was used to compare non-normal variables between two groups.

Table 2. Association between serum 25(OH)D level and physiological aging

Characteristics	All			Male			Female		
	Beta coefficient (95% CI)		*P*-value	Beta coefficient (95% CI)		*P*-value	Beta coefficient (95% CI)		*P*-value
	Crude model	Adjusted model [a]		Crude model	Adjusted model [a]		Crude model	Adjusted model [a]	
Log(25(OH)D)	-0.27 (-0.32, -0.22)	-0.04 (-0.08, 0.00)	0.039	-0.13 (-0.20, -0.06)	0.03 (-0.02, 0.09)	0.334	-0.32 (-0.38, -0.26)	-0.07 (-0.12, -0.01)	0.007
Quartiles									
Q1	Reference	Reference		Reference	Reference		Reference	Reference	
Q2	-0.23 (-0.29, -0.17)	-0.10 (-0.16, -0.05)	<0.001	-0.16 (-0.24, -0.08)	-0.02 (-0.09, 0.04)	0.448	-0.33 (-0.41, -0.25)	-0.16 (-0.23, -0.08)	<0.001
Q3	-0.29 (-0.36, -0.23)	-0.10 (-0.16, -0.04)	0.001	-0.20 (-0.29, -0.11)	-0.03 (-0.12, 0.04)	0.355	-0.40 (-0.49, -0.32)	-0.15 (-0.22, -0.07)	<0.001
Q4	-0.32 (-0.38, -0.26)	-0.05 (-0.11, -0.00)	0.022	-0.16 (-0.24, -0.07)	0.02 (-0.04, 0.10)	0.553	-0.42 (-0.51, -0.34)	-0.10 (-0.18, -0.02)	0.009
P for trend			0.173			0.429			0.076
Categories based on the Endocrine Society									
<30	Reference	Reference		Reference	Reference		Reference	Reference	
30-50	-0.23 (-0.33, -0.13)	-0.14 (-0.23, -0.05)	0.002	-0.26 (-0.41, -0.11)	-0.15 (-0.29, -0.02)	0.024	-0.22 (-0.34, -0.09)	-0.10 (-0.21, 0.00)	0.057
50-75	-0.42 (-0.52, -0.33)	-0.21 (-0.30, -0.13)	<0.001	-0.39 (-0.53, -0.25)	-0.16 (-0.29, -0.04)	0.009	-0.51 (-0.63, -0.40)	-0.24 (-0.33, -0.15)	<0.001
⩾75	-0.46 (-0.56, -0.36)	-0.15 (-0.24, -0.06)	0.001	-0.36 (-0.50, -0.22)	-0.09 (-0.22, 0.03)	0.131	-0.54 (-0.66, -0.42)	-0.17 (-0.27, -0.07)	0.001
P for trend			0.130			0.366			0.014

[a] Adjusted for age, race, education level, poverty-income ratio, body mass index, smoking, drinking, physical activity, sleep duration,

hypertension, diabetes and season of measurement.

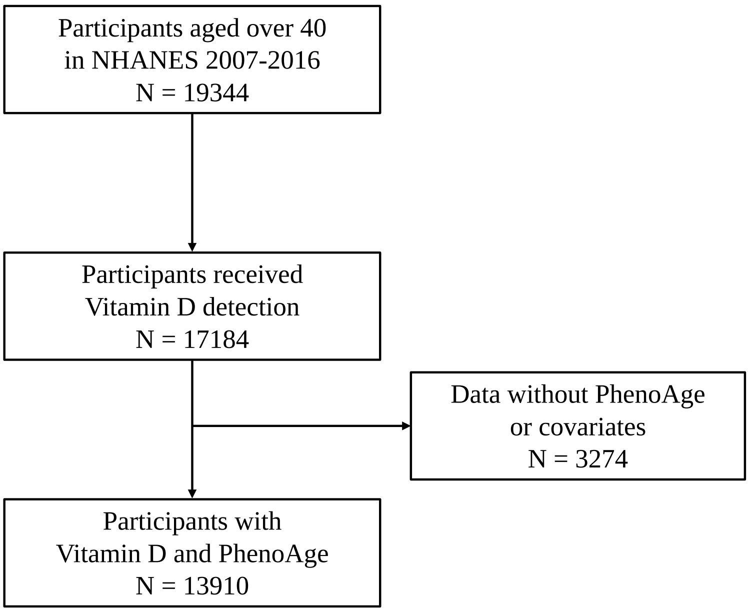

Figure 1 Flowchart of participants selection.

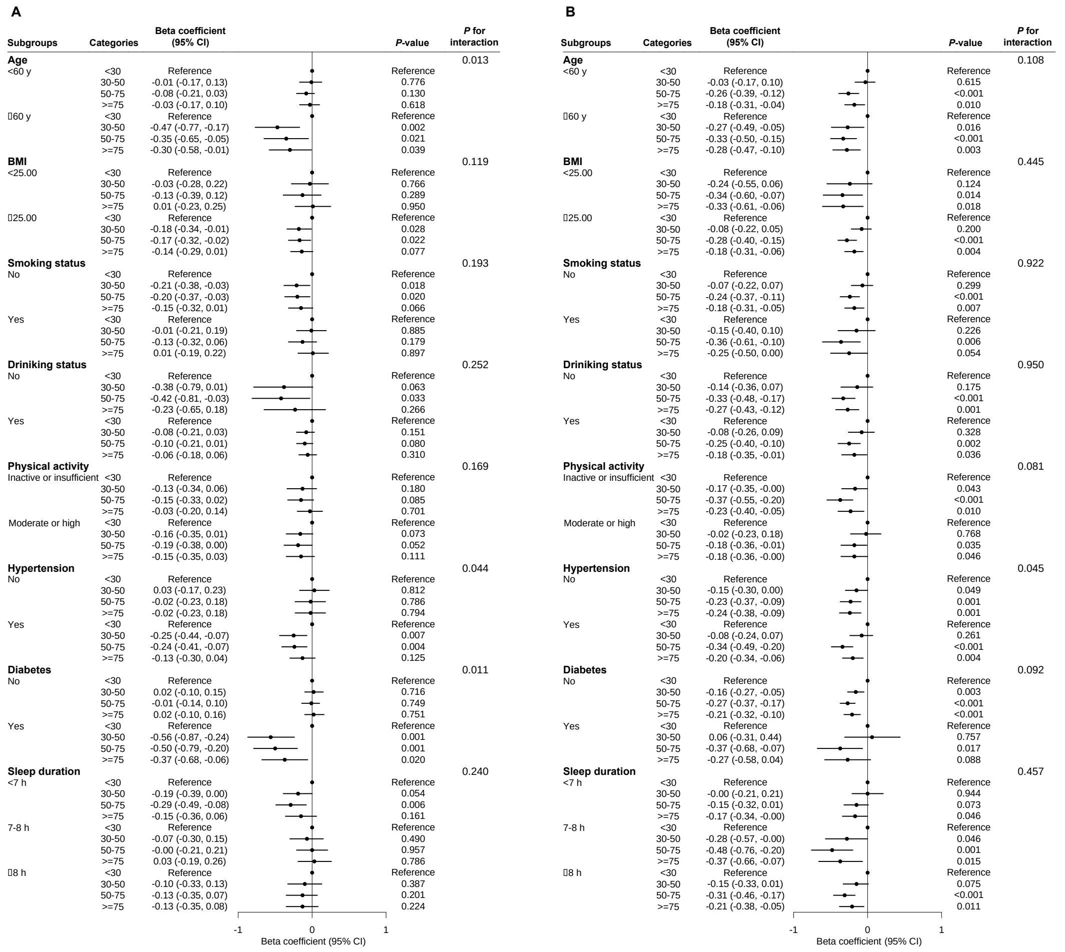

Figure 2 Subgroup analyses of the association between 25(OH)D categories and physiological aging among males (A) and females (B). The filled circles and horizontal lines represent regression coefficients and the 95% confidence intervals. Multiple linear regression models were adjusted for age, race, education level, poverty-income ratio, body mass index, smoking, drinking, physical activity, sleep duration, hypertension, diabetes and season of measurement. BMI, body mass index; CI, confidence intervals.

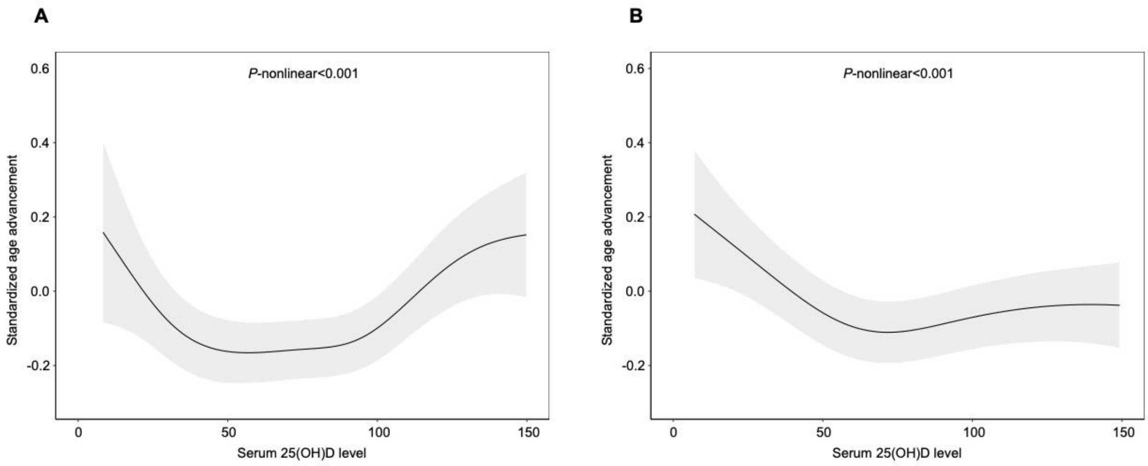

Figure 3 Nonlinear relationships between serum 25(OH)D level and physiological agingphysiological aging among males (A) and females (B). Generalized additive models were adjusted for age, race, education level, poverty-income ratio, body mass index, smoking, drinking, physical activity, sleep duration, hypertension, diabetes and season of measurement. The black solid line represents the smooth curve fit between variables. The grey band represents the 95% confidence interval of the fit.

Table 3. Threshold effect analysis of serum 25(OH)D level and physiological aging

Serum 25(OH)D levels	Beta coefficient (95% CI)	*P*-value
Male		
<38.20	-0.013 (-0.025, -0.002)	0.017
38.20-91.55	0.001 (-0.001, 0.002)	0.487
⩾91.55	0.005 (0.002, 0.008)	<0.001
Female		
<62.50	-0.007 (-0.010, -0.004)	<0.001
62.50-125.00	0.002 (0.000, 0.004)	0.029
⩾125.00	-0.001 (-0.005, 0.002)	0.535

CI: confidence intervals

Segmented regression models were adjusted for age, race, education level, poverty-income ratio, body mass index, smoking, drinking, physical activity, sleep duration, hypertension, diabetes and season of measurement.

Click here to access/download
Supporting Information
Supplemental materials.docx

Click here to access/download
Other
PLOSOne_Human_Subjects_Research_Checklist.docx

Revised Manuscript with Track Changes

Association of serum 25(OH)D level and ~~age advancement~~physiological aging: A Cross-Sectional Study of NHANES 2007-2016

Yi Feng[a]; Licheng Yu[b]; Jue Wang[a] *

[a] Nanhui new town Community Health Service Center, Pudong New Area, Shanghai, 201308, China.

[b] Prevention and Health Care Department, Heqing Community Health Service Center of Pudong New District, Shanghai, 201201, China.

Corresponding author. Jue Wang

E-mail address: wangjue180130@126.com

Abstract

~~Vitamin D deficiency is a global public health concern and is associated with various diseases and disorders, including aging. However, chronological age may not accurately reflect true biological aging status. Recently, PhenoAge has been developed to estimate an individual's biological age based on different physiological and clinical measures. Therefore, we investigated the relationship between serum 25(OH)D levels and age advancement calculated by PhenoAge in the National Health and Nutrition Examination Survey (NHANES). High serum 25(OH)D levels were linked to lower age advancement, but these associations showed nonlinearity in both males and females, with a U-shaped curve in males and an L-shaped curve in females. Interestingly, elevated levels of 25(OH)D did not slow down aging in females (>125 nmol/L) and could even accelerate aging in males (>91.55 nmol/L). Our findings suggest that Vitamin D deficiency promotes aging, but individuals without vitamin D deficiency may not benefit from vitamin D supplementation, which should be used cautiously.~~

Vitamin D plays a crucial role in various aspects of human body, including aging, but chronological age may not accurately reflect true physiological aging status. Recently, PhenoAge has been developed to estimate an individual's biological age based on different physiological and clinical measures. Therefore, we investigated the relationship between serum 25(OH)D levels and physiological aging calculated by PhenoAge in the National Health and Nutrition Examination Survey (NHANES). Biological age showed a negative correlation with serum 25(OH)D levels (β=-0.04; 95% CI: -0.08-0.00, p=0.039), but this correlation was only found to be dose-dependent for females (β=-0.07; 95% CI: -0.12-0.01, p=0.007), but not for males. Further analysis confirmed that these associations were nonlinear in both sexes, revealing a U-shaped curve for males and an L-shaped curve for females. Specifically, serum 25(OH)D levels were linked to decelerated aging at levels below 38.20 nmol/L in males (β=-0.013; 95% CI: -0.025--0.002, p=0.017) and 62.50 nmol/L in females (β=-0.007; 95% CI: -0.01--0.004, p<0.001). However, high serum 25(OH)D levels (>125 nmol/L) did not correlate with aging in females (β=-0.001; 95% CI: -0.005-0.002), while in males, elevated levels (>91.55 nmol/L) were associated with accelerated aging (β=0.005; 95% CI: 0.002-

Formatted: Font: Not Bold

Formatted: Font: Not Bold

Formatted: Font: Not Bold

Formatted: Font: Not Bold

Formatted: Font: Not Bold

Formatted: Font: Not Bold

Formatted: Font: Not Bold

Formatted: Font: Not Bold

Formatted: Font: Not Bold

Formatted: Font: Not Bold

0.008, p<0.001). Our findings indicate that vitamin D deficiency shows a potential link to accelerated physiological aging, but high levels of vitamin D do not link to decelerated physiological aging, offering valuable insights into the relationship between vitamin D and aging.

Key words: Vitamin D; Serum 25(OH)D; ~~Age advancement~~Physiological aging; PhenoAge; Nonlinear correlation

Formatted: Font: Not Bold

Formatted: Font: Not Bold

Formatted: Font: Not Bold

Formatted: Font: Not Bold

Formatted: Font: Not Bold

Formatted: Font: Not Bold

1. Introduction

Vitamin D is a fat-soluble vitamin that plays a crucial role in various aspects of human health, with its effects extending to bone metabolism, immune function, and overall physiological well-being.~~.~~ It is acquired through two primary sources~~There are two sources of vitamin D~~: endogenous production (mainly vitamin D2) in the skin upon exposure to ultraviolet B (UVB), and exogenous intake (vitamin D2 and D3) through dietary sources,~~endogenous production and dietary sources. The skin is the primary source of vitamin D, where it is synthesized when exposed to ultraviolet B (UVB) radiation from sunlight. It can also be obtained from certain foods,~~ such as fatty fish, egg yolks~~, fortified dairy products,~~ or supplements~~. Vitamin D plays a vital role in calcium and phosphorus homeostasis, bone health, and the immune system [1, 2]~~. Once Vitamin D is obtained, vitamin D can be converted into 25-hydroxyvitamin D [25(OH)D] in the liver, then transformed into the biologically active form, 1,25-dihydroxyvitamin D [$1,25(OH)_2D$], in the kidneys. This active form binds to the vitamin D receptor (VDR), regulating calcium and phosphate metabolism [1, 2].

The measurement of serum 25(OH)D levels is critical for evaluating vitamin D status [3], but can be influenced by various factors, such as geographic location, race and food intake [4]. According to established criteria, serum 25(OH)D levels below 20 ng/mL (50 nmol/L) are classified as deficient, while levels between 20-30 ng/mL (50-75 nmol/L) are considered insufficient [5-7]. Vitamin D deficiency is a global public health concern, associated with increased risk of type 2 diabetes (T2D), hypertension, multiple sclerosis, etc [8-10]. The prevalence of vitamin D deficiency is particularly high among the elderly, with studies suggesting that it may accelerate chronological aging and contribute to age-related diseases such as cardiovascular disorders

~~Vitamin D deficiency is a global public health concern, with high prevalence rates observed in many countries. Serum 25 hydroxyvitamin D [25(OH)D] is the best indicator of an individual's vitamin D status, as it reflects both endogenous production and dietary intake [3]. Vitamin D deficiency is generally defined as a serum 25(OH)D level below 20 ng/mL (50 nmol/L), while insufficiency is defined as a level between 20-30 ng/mL (50-75 nmol/L)~~[5-7]~~. Individual serum 25(OH)D level can be influenced~~

by various factors, such as geographic location, race and food intake. Geographic location determines the latitude, season, and air quality, which affect the amount of UVB radiation reaching the skin, influencing endogenous vitamin D production [4]. Individuals with darker skin pigmentation require more sun exposure to produce adequate amounts of vitamin D [11]. Vitamin D synthesis decreases with age, leading to a higher risk of deficiency in the elderly [2]. Obesity or certain medical conditions like malabsorption disorders, liver and kidney diseases, and medications can also affect vitamin D metabolism and status [8]. Vitamin D deficiency has been linked to a higher risk of various diseases, such as osteoporosis, cardiovascular disease, cancer, and autoimmune disorders. It is also suggested that low level of 25(OH)D is strongly associated with all-cause mortality [9].

Considering the importance of Vitamin D for health and the association between serum 25(OH)D with multiple disorders, vitamin D deficiency could also accelerate human aging and age-related diseases [11]. However, several large trials of vitamin D supplementation have failed to demonstrate significant benefits on cardiovascular disease, casting doubt on the observational evidence [12, 13]. This ambiguity highlights the complexity of the association between vitamin D and aging. Previous studies have a limitation of reliance on chronological age, which does not account for individual differences in physiological aging. While chronological age measures the passage of time, physiological aging reflects the rate of physiological decline, which can vary significantly among individuals due to genetic, environmental, and lifestyle factors However, the widely used chronological age does not perfectly reflect true biological aging, as individuals age at different rates due to genetic, environmental, and lifestyle factors [14]. Some individuals may age biologically faster or slower than their chronological age underscoring the need for more accurate biomarkers of aging. Some individuals may age biologically faster or slower than their chronological age indicates, prompting researchers to search for more precise biomarkers of aging beyond chronological age.

–To address this challenge, several methods have been created, including clinical biomarkers-based approaches (PhenoAge, GrimAge etc) and methylation-based

Field Code Changed

Field Code Changed

Field Code Changed

Field Code Changed

Field Code Changed

Field Code Changed

Field Code Changed

Field Code Changed

algorithms (DNAm PhenoAge acceleration, mortality risk score.etc) [15].~~Yet, these DNA methylation-based algorithms have several potential drawbacks, as DNA methylation patterns are intricate and can vary across different tissues, cell types, and even within the same individual over time~~ [17, 18]~~. This variability poses a challenge in establishing robust and reliable predictive models that can be universally applied.~~Among these, the PhenoAge method has emerged as a promising tool, employs a machine learning algorithm to estimate an individual's biological age based on various physiological and clinical measure, including inflammation (e.g. C-reactive protein), metabolic health (e.g. glucose, albumin), and organ function (e.g. alkaline phosphatase, lymphocyte percent) [16]. This method was developed by using data from the National Health and Nutrition Examination Survey (NHANES) and over 4,000 adults aged 20-84 years with complete biomarker data were used to train and validate the machine learning model ~~uses a machine learning algorithm to estimate an individual's biological age based on various physiological and clinical measure, including inflammation (e.g. C-reactive protein), metabolic health (e.g. glucose, albumin), and organ function (e.g. alkaline phosphatase, lymphocyte percent)~~ [17]. PhenoAge demonstrates high accuracy, with a mean absolute error of approximately 3.6 years in predicting chronological age [16]. In a three-year longitudinal study, DO-HEALTH trial, involving 777 older adults, vitamin D supplement slows PhenoAge index, but not the other physiological aging index, including GrimAge, GrimAge2 and DunedinPACE [18]. At baseline, the mean 25(OH)D level among participants was 22.69 ng/mL, which falls within the insufficient range (20-30 ng/mL), proving that vitamin D supplement is beneficial for the elderly with vitamin D deficiency. However, the comprehensive picture of the association between vitamin D levels and physiological aging remains to be fully elucidated, particularly in the general population without vitamin D deficiency or insufficiency.

~~However, there is currently no universally accepted standard for aging biomarkers. One promising approach involves a set of algorithms that analyze DNA methylation data to estimate an individual's biological age or mortality risk~~ [16]~~. Yet, these DNA methylation-based algorithms have several potential drawbacks, as DNA methylation patterns are intricate and can vary across different tissues, cell types, and even within~~

Field Code Changed

Field Code Changed

Field Code Changed

Field Code Changed

Field Code Changed

Field Code Changed

Field Code Changed

the same individual over time [17, 18]. This variability poses a challenge in establishing robust and reliable predictive models that can be universally applied. Factors such as lifestyle, environment, and underlying health conditions can influence DNA methylation patterns, affecting the accuracy of age predictions [17]. Accounting for these confounding factors in the predictive models can be challenging, another promising aging biomarkers consists of several algorithms derived from blood chemistries and clinical data [17]. Compared with DNA methylation method, clinical parameters-based algorithms are equally or more predictive of multiple diseases, disorders, and mortality and sensitive to a range of causes hypothesized to accelerate aging. Furthermore, studies incorporating some, but not all, clinical markers in a specific algorithm could also assess the aging process [20].

We utilized PhenoAge as the indicator of aging. PhenoAge is a biological age predictor that uses a machine learning algorithm to estimate an individual's biological age based on various physiological and clinical measure, including inflammation (e.g. C-reactive protein), metabolic health (e.g. glucose, albumin), and organ function (e.g. alkaline phosphatase, lymphocyte percent) [19]. It was developed by using data from the National Health and Nutrition Examination Survey (NHANES) and over 4,000 adults aged 20-84 years with complete biomarker data were used to train and validate the machine learning model [17]. PhenoAge can predict chronological age with a mean absolute error of around 3.6 years, and it has been demonstrated to outperform other biological age estimators, such as the widely used Horvath clock. PhenoAge has also been validated against mortality risk, showing that higher PhenoAge associated with increased risk of all cause and cause specific mortality. Therefore, it can be utilized to track an individual's biological aging trajectory over time and employed in research to comprehend the factors influencing the pace of biological aging [17]. Overall, PhenoAge provides a more comprehensive and accurate assessment of an individual's biological age compared to simply relying on chronological age. This can have important implications for disease prediction, prevention, and healthy aging interventions.

In this study, the association of serum 25(OH)D and age advancementphysiological

Field Code Changed

Field Code Changed

Field Code Changed

Field Code Changed

Field Code Changed

Field Code Changed

Field Code Changed

aging calculated by PhenoAge method was investigated in the NHANES data from 2007-2016. By exploring this relationship, the study aims to enhance our understanding of the potential link between vitamin D and aging, offering valuable insights into the underlying mechanisms and implications for public health.

This analysis could enhance our understanding of the possible link between vitamin D and aging, and assist in determining the appropriate use of vitamin D supplementation.

2. Method

2.1 Study design and population

This study was conducted and reported in adherence to STROBE guidelines for observational studies. This study utilized data derived from National Health and Nutrition Examination Survey (NHANES), a nationwide survey conducted by the National Center for Health Statistics (NCHS) of the Centers for Disease Control and Prevention (CDC). All data included in current study are publicly available through the NHANES website [19]. NHANES includes in-home interview, physical measurement and laboratory tests. Written consents were provided by all participants at recruitment. This study obtained 50588 individuals of NHANES data from 2007-2016. We excluded participants with incomplete information and participants aged below 40 years old. A total of 13,910 participants were included for further analysis (Figure 1).

2.2 Measurement of serum 25(OH)D concentrations

Serum 25-hydroxyvitamin D [25(OH)D] concentrations were measured as a reliable index to reflect vitamin D status. From cycle 2007-2008 and beyond, liquid chromatography-tandem mass spectrometry (LC-MS/MS) was used to measure serum 25(OH)D concentrations, including 25(OH)D3, epi-25(OH)D3, and 25(OH)D2 in human serum. We calculated the 25(OH)D levels by adding 25(OH)D3 and 25(OH)D2 levels. According to the American Endocrine Society guidelines, we transformed serum 25(OH)D concentration into categorical variables: deficiency (<50 nmol/L),

Formatted: Font color: Custom Color(RGB(3,30,255))
Formatted: Indent: First line: 0.29"
Formatted: Font color: Custom Color(RGB(3,30,255))
Formatted: Font color: Custom Color(RGB(3,30,255))
Formatted: Font color: Custom Color(RGB(3,30,255))
Formatted: Font color: Custom Color(RGB(3,30,255))
Formatted: Font color: Custom Color(RGB(3,30,255))
Field Code Changed
Formatted: Font color: Custom Color(RGB(3,30,255))
Formatted: Font color: Custom Color(RGB(3,30,255))

insufficiency (50–75 nmol/L) and sufficiency (≥75 nmol/L) [20].

2.3 Measurement of ~~biological aging~~physiological aging

In this study, we utilized PhenoAge as the indicator of aging, considering its high robustness and feasibility in NHANES data as a reliable predictor of mortality and age-related disease risks. The PhenoAge algorithm is constructed using NHANES III (1988–1994) by elastic-net regression, encompasses 10 markers: albumin, creatinine, glucose, white blood cell count, lymphocyte percent, red cell distribution width, mean red cell volume, C-reactive protein, alkaline phosphatase and chronological age [21]. The R package BioAge was used to compute PhenoAge [16]. One of the markers, C-reactive protein, was unavailable from 2011 to 2018. According to previous studies, there is a strong correlation (correlation coefficient of 0.99) between PhenoAge measurements with and without C-reactive protein, utilizing an algorithm that excludes this marker to calculate PhenoAge [22, 23]. Due to the absence of C-reactive protein data, we employed PhenoAge algorithm that excludes this marker in the current study. We further calculated physiological aging ~~PhenoAge advancement~~ as the difference between PhenoAge and chronological age. A positive value indicates accelerated aging, while a negative value indicates slower aging. Subsequently, physiological aging ~~PhenoAge advancement~~ was~~ere~~ standardized to have a mean of 0 and a standard deviation (SD) of 1.

2.4 Covariates

NHANES interviews were conducted by trained interviewers, where questionnaires were administered to collect information on demographics [age, gender, race, education level, poverty income ratio (PIR)] and lifestyle (smoking and drinking status, physical activity and comorbidities). Body mass index (BMI) were measured at the Mobile Examination Center.

Smoking status was classified as never smokers (had smoked <100 cigarettes in their lifetime), current smokers (had smoked ≥100 cigarettes in their lifetime and still

Field Code Changed

Field Code Changed

Field Code Changed

Field Code Changed

Formatted: Indent: First line: 0 ch

smoked at the time of the interview) and former smokers (had smoked ⩾100 cigarettes in their lifetime but had quit smoking at the time of the survey). Drinking status was classified as never drinkers (had drunk <12 drinks in their lifetime), current drinkers (had drunk ⩾12 drinks in their lifetime and at least one drink in the past year) and former drinkers (had drunk ⩾12 drinks in their lifetime but no drink in the past year). Metabolic equivalent task (MET)-min per week were calculated to assess physical activity, and levels of physical activity were categorized as: inactive (no regular physical activity), insufficient (<500 MET-min activity/week), moderate (500-1,000 MET-min activity/week) and high (⩾1,000 MET-min activity/week). For sleep duration, participants responded to the question: “How much sleep do you usually get at night on weekdays or workdays?” and were categorized into 3 groups: <7 h, 7-8 h and ⩾8h). Hypertension was ascertained by self-reporting history of hypertension, use of medication for hypertension or physical examination (systolic blood pressure⩾140 mmHg or diastolic blood pressure⩾90 mmHg). Diabetes was ascertained by self-reporting history of diabetes or results from laboratory examination (fasting glucose level ⩾126 mg/dL or glycohemoglobin ⩾6.5%).

2.5 Statistical analysis

Continuous data were reported as mean ± standard deviation (SD) or median (interquartile range). Categorical data were reported as numbers (percentage). Weighted m~~M~~ultivariable linear regression models were constructed to estimate the association between serum 25(OH)D status and standardized ~~age advancement~~physiological aging. Serum 25(OH)D level were log-transformed due to skewed distribution. To evaluate potential dose-response patterns, 25(OH)D level was analyzed both as a continuous variable (log-transformed) and categorically through quartiles and four clinically defined sufficiency categories. Beta coefficients with 95% confidence intervals (CIs) were reported, representing the change in dependent variable per unit increase in the

Formatted: Indent: First line: 0.29"

Formatted: Indent: First line: 0.29"

Formatted: Font color: Blue

independent variable. The use of beta coefficients provides interpretable estimates for linear associations when analyzing continuous dependent variables, which is widely recommended in epidemiological studies [24]. Correlation analysis was not employed because it does not account for confounding factors, whereas the primary aim of current study is to estimate the confounder-adjusted association between 25(OH)D status and ~~PhenoAge advancement~~physiological aging. All m~~M~~odels were adjusted for age, race, education level, PIR, BMI, smoking, drinking, physical activity, sleep duration, hypertension, diabetes and season of measurement.

To explore the non-linear relationship between serum 25(OH)D and ~~age advancement~~physiological aging, generalized additive model (GAM) was employed to fit the smooth curve. The threshold effect was further estimated using segmented regression model. A *P*-value of <0.05 was considered statistical significance. All data analyses were performed using R version 4.2.1 (R Core Team, Vienna, Austria). R package 'BioAge' was used to calculate PhenoAge [16], while package 'mgcv' was used to conduct GAM analysis [25].

2.6 Ethical considerations

This study utilized data from NHANES study, which was conducted in accordance with the principles in the Declaration of Helsinki. The NHANES protocol received prior ethical approval from the National Center for Health Statistics' Review Board. Since our study is a secondary analysis of publicly accessible, de-identified NHANES data sets, additional institutional review board approval was not required [26, 27].

3. Results

3.1 Characteristics of the study population

The current study included a total of 6836 males and 7074 females~~ were included in the current study.~~, with ~~Table 1 showed ~~the characteristics of the study population presented in Table 1~~,~~. ~~revealing s~~Significant differences between males and females in the distribution of basic information, such as age, race, BMI, and poverty-income ratio

Formatted: Font color: Blue

Formatted: Font color: Blue

Formatted: Indent: First line: 2 ch

Field Code Changed

Field Code Changed

Formatted: Font: Bold

Formatted: Font color: Blue

Formatted: Font color: Blue

Formatted: Font: Not Bold

($p < 0.001$), except for education level. Additionally, gender-specific differences were noted in behaviors influencing aging.Differences in human behaviors affecting aging, such as smoking, drinking, physical activity, and sleep duration, were also observed between males and females ($p < 0.001$). Notably,Additionally, males had a higher prevalence of diabetes than females. The median serum 25(OH)D level in females was higher (66.60nmol/L) than in males (63.80 nmol/L), and the PhenoAge calculated from 10 clinical biomarkers was lower in females than in males. Furthermore, the age advancementphysiological aging, which represents the difference between PhenoAge and chronological age, was also lower in females compared to males ($p < 0.001$).

3.2 Association of serum 25(OH)D level and age advancementphysiological aging

The associations between serum 25(OH)D levels and age advancementphysiological aging were was analyzed and presented in Table 2. After adjusting for age, race, education level, PIR, BMI, smoking, drinking, physical activity, sleep duration, hypertension, and diabetes, age advancementphysiological aging was found to be negatively associated with serum 25(OH)D levels (β=-0.04; 95% CI: -0.08-0.00). However, this association was statistically significant only observed in females (p=0.007), and not in males (p=0.334). To further explore this relationship, the population was categorized into four groups After dividing the whole population into four categories based on quartiles for analysis., hHigher serum 25(OH)D levels were inversely negatively associated with age advancementphysiological aging, with no apparent dose-dependent trend as the p-value for trend was not significant (p=0.173). This pattern was consistent in females but absent in males.Findings in females was consistent with those of the overall population, while no significant correlations were observed in males. Furthermore, the population was categorized into four groups according to the endocrine society guidelines., which similarly revealed that higher serum 25(OH)D levels were associated with reduced physiological aging, albeit without dose-dependent relationships (p=0.076) in whole population and females. Consistent with the quartile analysis results, higher serum 25(OH)D levels were linked to reduced age advancement but without dose-dependent relationships (p=0.076) in

~~whole population and females.~~ Interestingly, moderate ~~middle~~ level of 25(OH)D levels (30-50 and 50-75 nmol/L) were associated with ~~could~~ reduced ~~age advancement~~physiological aging in males categorized based on endocrine society guidelines, though these statistical differences were not observed in male based on quartile categories~~while these statistical differences were not observed in male based on quartile categories~~.

Stratified analysis was conducted to further investigate the impact of various factors on the relationship between serum 25(OH)D levels and ~~age advancement~~physiological aging. In males, no overall association was observed~~While no association was found in the male population;~~, however, in male subpopulations over 60 years of age with hypertension or diabetes, higher serum 25(OH)D levels were associated with slower physiological aging ~~stratified analysis revealed that high serum 25(OH)D levels were linked to slower age advancement in male subpopulations over 60 years old with hypertension or diabetes~~. Additionally, there were statistically significant interactions between serum 25(OH)D levels and age, hypertension, or diabetes (Fig.~~1~~2A). In contrast, i~~I~~n females, factors such as age, BMI, smoking, drinking, physical activity, and hypertension did not alter the inverse association between them, indicating the robustness of this relationship in females (Fig.2~~1~~B).

3.3 Non-linear correlation between serum 25(OH)D level and ~~age advancement~~physiological aging

Given the non-significant ~~P for~~ trend in Table 2, indicating a non-linear correlation, a generalized additive model (GAM) was utilized to investigate the potential link. The analysis revealed a nonlinear relationship between serum 25(OH)D level and standardized ~~age advancement~~physiological aging in both males and females (p<0.001) (Fig ~~2~~3 A-B). In males, ~~t~~the relationship ~~he curve~~ exhibited a U shape, while in females, it resembled more of like an L shape, as confirmed by segmented regression modeling. Two inflection points were identified in males at 38.2 nmol/L and 91.55 nmol/L. Low serum 25(OH)D levels (<38.2 nmol/L) were negatively correlated with standardized ~~age advancement~~physiological aging (β=-0.013; 95% CI: -0.025--0.002), whereas high

levels (>91.55 nmol/L) showed a positive association (β=0.005; 95% CI: 0.002-0.008). For females, serum 25(OH)D levels below 62.5 nmol/L were negatively linked to ~~age advancement~~physiological aging (β=-0.007; 95% CI: -0.01--0.004), with no significant association observed beyond 125 nmol/L (β=-0.001; 95% CI: -0.005-0.002). Overall, low (<38.2 nmol/L) or high (>91.55 nmol/L) serum 25(OH)D levels in males were associated with ~~may~~ acceleratinge~~e~~ aging, while in females, only low levels (<62.5 nmol/L) pose a risk factor for aging (Table 3).

4. Discussion

In the current study, the association between serum 25(OH)D level and ~~age advancement~~physiological aging ~~calculated as the difference between PhenoAge and chronological age~~ were investigated in NHANES. The analysis revealed a negative correlation between physiological ~~A~~age ~~progression showed a negative correlation with~~and serum 25(OH)D level, whether categorized by quartiles or according to the endocrine society's criteria. This correlation exhibited a~~was found to be~~ dose-dependent relationship in females but not in the overall population or males. Further analysis confirmed these associations were nonlinear in both genders~~males and females~~, b with a ~~ut the correlation analysis revealed a~~ U-shaped curve in males and an L-shaped curve in females.

Numerous studies have established ~~reported~~ that serum 25(OH)D level, representing vitamin D status in our body, are~~is~~ associated with various ~~many~~ diseases or disorders, including all-cause mortality, cardiovascular diseases, and aging etc. Multiple large-scale observational studies have consistently shown a negative correlation between serum 25(OH)D levels and all-cause mortality risk, lower serum 25(OH)D levels are associated with an increased risk of all-cause mortality, and~~with~~ a dose-response relationship observed [28, 29]. Individuals with lower vitamin D levels (typically below 30 ng/mL) linked to ~~have~~ a higher risk of all-cause mortality compared to those with adequate vitamin D status [30]. Regarding aging, studies have reported that a gradual decline in serum 25(OH)D levels with chronological age [31]. This decline is attributed to several factors, including decreased sun exposure, reduced

Field Code Changed

Field Code Changed

Field Code Changed

capacity of the skin to synthesize vitamin D, decreased dietary intake of vitamin D, and impaired renal function, resulting in vitamin D deficiency in older adults [32]. Additionally, we also found that low serum 25(OH)D levels were associated with increased ~~high age advancement~~physiological aging, suggesting vitamin D may also play a crucial role in the regulation of the aging process. Adequate 25(OH)D levels are essential for maintaining calcium absorption and bone health preventing osteoporosis, a common age-related condition, and regulating calcium homeostasis and bone metabolism ~~necessary for maintaining calcium absorption and deposition in bones, which is essential for bone health and preventing osteoporosis, a common age-related condition, indicating its importance in regulating calcium homeostasis and bone metabolism~~ [33]. Moreover, vitamin D ~~it~~ can ~~also~~ interact with ~~and enhance the activity of~~ antioxidant and anti-inflammatory pathways, such as activating Nrf2 (nuclear factor erythroid 2-related factor 2) and SIRT1 (sirtuin 1) signaling cascades, and suppressing ~~or suppress~~ the production of pro-inflammatory cytokines, including interleukin-6 (IL-6) and tumor necrosis factor-alpha (TNF-α), through inhibiting nuclear factor-κB (NF-κB)~~,~~. These actions lead~~ing~~ to reduction of inflammation and oxidative stress, both of which are known to contribute to the aging process [33, 34]. Therefore, vitamin D and aging have a mutual influence on each other.

The significant association between vitamin D and aging suggests that vitamin D supplementation could be an effective way to reverse or mitigate various diseases and disorders. Vitamin D supplementation may help lower blood pressure and improve endothelial function, leading to reduced risk of cardiovascular events, such as heart attacks and strokes, and improved cardiovascular outcomes [35].~~ In terms of brain health, supplementation of vitamin D has been linked to improved cognitive function, particularly in older adults, and could potentially reduce the risk of dementia and Alzheimer's disease~~ [38]~~.~~ A meta-analysis of 75 randomized controlled trials in 2014 revealed that vitamin D supplementation was associated with a 6% reduction in all-cause mortality [36]. Some studies suggest that the impact of vitamin D supplementation on all-cause mortality may be more pronounced in certain subgroups, such as older adults or individuals with vitamin D deficiency. For example, vitamin D

Field Code Changed

Field Code Changed

Field Code Changed

Field Code Changed

Field Code Changed

Field Code Changed

supplementation was associated with a reduced risk of all-cause mortality in trials with a mean baseline 25(OH)D level <30 ng/mL (RR 0.89, 95% CI 0.80-0.99), but not in trials with a mean baseline 25(OH)D level ≥30 ng/mL (RR 1.04, 95% CI 0.97-1.11) [37]. Thus, the growing body of evidence highlights the potential of vitamin D supplementation in promoting overall health and reducing the risk of various chronic diseases, particularly in elderly people, where serum vitamin D levels tend to decline with age.

~~.~~Despite its potential benefits, the use of vitamin D supplements has increased markedly, and concerns about excessive intake have emerged. ~~However,~~ the Vitamin D and Omega-3 Trial (VITAL) study, a randomized trial involving over 25,000 participants, discovered that vitamin D supplementation did not significantly reduce the risk of all-cause mortality or lower risk of fractures among generally healthy midlife and older adults ~~who did not have~~ without vitamin D deficiency or osteoporosis.[38]. Furthermore,~~Therefore, the results have been mixed, with some studies showing a reduction in mortality and others not. It's important to note that the specific benefits of vitamin D supplementation may depend on factors such as the dose, duration of supplementation, and individual health status~~ [42]~~.~~ increased intake of vitamin D supplements by the general population and a growing number of high-dose prescriptions without medical monitoring might result in a greater risk of excessive vitamin D toxicity[39]~~.~~. ~~Those evidences caution against the widespread use of vitamin D supplementation in general population, and o~~Our current results ~~furtherly~~ indicated that the correlation of serum 25(OH)D level and ~~age advancement~~physiological aging followed a U-shaped curve in males and an L-shaped curve in females, revealing that high serum level of 25(OH)D is not associated with decelerated aging~~not only lack beneficial effects~~ in ~~both~~ either males and females, and even associated with accelerated physiological~~but also promotes~~ aging in males. These detrimental effects may stem from the toxicity of excessive vitamin D. When vitamin D intake is too high, serum levels of its metabolites [25(OH)D and $24,25(OH)_2D$] rise beyond the binding capacity of vitamin D binding protein, leading to an increase in the free active metabolite,

Field Code Changed

Field Code Changed

Field Code Changed

Field Code Changed

$1,25(OH)_2D$. This active form reaches the VDR in the nucleus, constantly promoting gene expression that enhances calcium absorption and bone mobilization. Consequently, this can result in hypercalcemia or hyperphosphatemia, which in turn suppresses initial parathyroid hormone (iPTH) level [40, 41]. Such disrupting balance of calcium and hormone may contribute to cardiovascular disease, kidney damage, and skin aging. Research reported that the working diagnosis of vitamin D toxicity is diagnosed based on total 25(OH)D concentrations of 32~80ng/mL (80~200 nmol/L) detected by immunoassay [42]. Our results found the 25(OH)D levels over 125 nmol/L in female participants were linked to accelerated physiological aging, approximately consistent with those reports.

Our results reported differences of dose-response curve between in male and in female, which may be attributed to the sex hormones, especially estrogen. As women age, particularly during the menopausal transition and after menopause, their ovaries produce less estrogen, leading to a decline of estrogen levels~~,~~. This decline ~~which~~ contributes to the thinning and wrinkling of the skin, as well as changes in hair texture and growth patterns [43]. Some studies suggest that vitamin D could enhance the activity of estrogen receptors (ER) via interacting with them, potentially amplifying the anti-aging effects of estrogen in the body [44]~~(~~. Vitamin D and estrogen also have a synergistic effect on bone metabolism, helping to prevent osteoporosis and fractures, especially in postmenopausal women [45]. Additionally, vitamin D regulate estrogen metabolism, leading to higher circulating estrogen levels and subsequently delay aging [44]. Therefore, ~~a~~ high level of serum 25(OH)D may not be associated with ~~age advancement~~physiological aging in females, but the mechanisms underlying the adverse effects of extremely high levels (>100 nmol/L) in males remain unclear~~but the detail mechanisms of why extreme high level of serum 25(OH)D level (>100 nmol/L) is bad for males but not for females,~~. ~~f~~Further studies are needed to clarify the complex interplay between these two important hormonal systems.

The current study has several limitations~~There are several limitations in the current study~~. First~~ly~~, this cross-sectional ~~this is a cross-sectional~~ study~~,~~ only providi~~ng~~e information ~~about~~on the association of serum 25(OH)D level and ~~age~~

Formatted: Font color: Auto
Field Code Changed
Formatted: Font color: Auto
Formatted: Font color: Auto
Field Code Changed
Formatted: Font color: Auto
Formatted: Font color: Auto
Formatted: Font color: Red
Field Code Changed
Field Code Changed
Field Code Changed
Field Code Changed

advancementphysiological aging, making it difficult to establish infer causal relationships. Secondly, we applied PhenoAge method to calculate age advancementphysiological aging, but the data of C-reactive protein were not availableis absent. Finally, the gender-specific differences in results require further detailed analysis to fully understand the underlying mechanisms.

the different results in male and female were obtained, and detailed analysis should be carried out to furtherly investigate.

Formatted: Indent: First line: 0.29"

5. Conclusion

In this study, we investigated the relationship between serum 25(OH)D levels and biological age in NHANES, and discovered found that there was a negative correlation between physiological age and serum 25(OH)D level, and— these associations were nonlinear in both genders, revealing a U-shaped curve for males and an L-shaped curve for females. Low serum 25(OH)D levels shows a potential link to accelerated physiological aging in both genders, but high serum 25(OH)D levels do not correlate with decelerated physiological aging, and even associated with accelerated physiological aging in male. Our results suggest that the association between vitamin D levels with physiological aging is complex and whether vitamin D supplementation in individuals with sufficient 25(OH)D levels is beneficial warrants further investigation.

Formatted: Indent: First line: 0.29"

serum 25(OH)D levels were inversely associated to age status in NHANES. These relationships exhibited nonlinearity in both males and females. However, the correlation took the form of a U-shaped curve in males and an L-shaped curve in females indicating that a deficiency in vitamin D could accelerate the aging process. Interestingly, high levels of vitamin D did not decelerate aging in females (>125 nmol/L) and could even accelerate aging in males (>91.55 nmol/L). Our findings suggest that individuals without a vitamin D deficiency may not derive benefits from vitamin D supplementation, which should be approached with caution.

Contributions

Yi Feng: Conceptualization; Methodology; Data curation; Formal analysis; Writing original draft**; Licheng Yu**: Data curation; Formal analysis; **Jue Wang**: Project administration; Supervision; Writing - review &editing.

~~**Acknowledgments**~~

~~This work was supported by Special Clinical Research Project of Shanghai Municipal Health Commission [20224Y0335].~~

Formatted: Indent: First line: 0.29"

Conflict of interest

~~We declare that we have no conflicts of interest.~~The authors declare no competing financial or non-financial interests related to this work. NHANES is a publicly available dataset managed by the Centers for Disease Control and Prevention. The authors confirm that there is no affiliation with or involvement from the NHANES program administrators in this study. The funding source had no role in study design, data collection, analysis, or manuscript preparation.

Formatted: Font color: Custom Color(RGB(3,30,255))

Formatted: Indent: First line: 0.29", Line spacing: 1.5 lines

Reference

Field Code Changed

Formatted: Font: (Default) Times New Roman

1. Dominguez LJ, Farruggia M, Veronese N, Barbagallo M. Vitamin D Sources, Metabolism, and Deficiency: Available Compounds and Guidelines for Its Treatment. Metabolites. 2021;11(4). Epub 2021/05/01. doi: 10.3390/metabo11040255. PubMed PMID: 33924215; PubMed Central PMCID: PMCPMC8074587.
2. Bikle D, Christakos S. New aspects of vitamin D metabolism and action - addressing the skin as source and target. Nat Rev Endocrinol. 2020;16(4):234-52. Epub 2020/02/08. doi: 10.1038/s41574-019-0312-5. PubMed PMID: 32029884.
3. Couchman L, Moniz CF. Analytical considerations for the biochemical assessment of vitamin D status. Ther Adv Musculoskelet Dis. 2017;9(4):97-104. Epub 2017/04/07. doi: 10.1177/1759720X17692500. PubMed PMID: 28382113; PubMed Central PMCID: PMCPMC5367642.
4. Rahman A, Elmi A. Air pollutants are negatively associated with vitamin D-synthesizing UVB radiation intensity on the ground. Sci Rep. 2021;11(1):21480. Epub 2021/11/04. doi: 10.1038/s41598-021-00980-6. PubMed PMID: 34728744; PubMed Central PMCID: PMCPMC8563978.
5. Bordelon P, Ghetu MV, Langan RC. Recognition and management of vitamin D deficiency. Am

Fam Physician. 2009;80(8):841-6. Epub 2009/10/20. PubMed PMID: 19835345.

6. Cashman KD. Vitamin D Deficiency: Defining, Prevalence, Causes, and Strategies of Addressing. Calcif Tissue Int. 2020;106(1):14-29. Epub 2019/05/10. doi: 10.1007/s00223-019-00559-4. PubMed PMID: 31069443.

7. Demay MB, Pittas AG, Bikle DD, Diab DL, Kiely ME, Lazaretti-Castro M, et al. Vitamin D for the Prevention of Disease: An Endocrine Society Clinical Practice Guideline. J Clin Endocrinol Metab. 2024. Epub 2024/06/03. doi: 10.1210/clinem/dgae290. PubMed PMID: 38828931.

8. Zhao S, Qian F, Wan Z, Chen X, Pan A, Liu G. Vitamin D and major chronic diseases. Trends Endocrinol Metab. 2024. Epub 2024/06/02. doi: 10.1016/j.tem.2024.04.018. PubMed PMID: 38824035.

9. Gaksch M, Jorde R, Grimnes G, Joakimsen R, Schirmer H, Wilsgaard T, et al. Vitamin D and mortality: Individual participant data meta-analysis of standardized 25-hydroxyvitamin D in 26916 individuals from a European consortium. Plos One. 2017;12(2):e0170791. Epub 2017/02/17. doi: 10.1371/journal.pone.0170791. PubMed PMID: 28207791; PubMed Central PMCID: PMCPMC5312926.

10. Fang A, Zhao Y, Yang P, Zhang X, Giovannucci EL. Vitamin D and human health: evidence from Mendelian randomization studies. Eur J Epidemiol. 2024;39(5):467-90. Epub 2024/01/12. doi: 10.1007/s10654-023-01075-4. PubMed PMID: 38214845.

11. Berridge MJ. Vitamin D deficiency accelerates ageing and age-related diseases: a novel hypothesis. The Journal of physiology. 2017;595(22):6825-36. Epub 2017/09/28. doi: 10.1113/JP274887. PubMed PMID: 28949008; PubMed Central PMCID: PMCPMC5685827.

12. Zittermann A, Ernst JB, Prokop S, Fuchs U, Dreier J, Kuhn J, et al. Effect of vitamin D on all-cause mortality in heart failure (EVITA): a 3-year randomized clinical trial with 4000 IU vitamin D daily. Eur Heart J. 2017;38(29):2279-86. Epub 2017/05/13. doi: 10.1093/eurheartj/ehx235. PubMed PMID: 28498942.

13. Scragg R, Stewart AW, Waayer D, Lawes CMM, Toop L, Sluyter J, et al. Effect of Monthly High-Dose Vitamin D Supplementation on Cardiovascular Disease in the Vitamin D Assessment Study : A Randomized Clinical Trial. JAMA Cardiol. 2017;2(6):608-16. Epub 2017/04/07. doi: 10.1001/jamacardio.2017.0175. PubMed PMID: 28384800; PubMed Central PMCID: PMCPMC5815022 ICMJE Form of Disclosure of Potential Conflicts of Interest and none were reported.

14. Xia X, Wang Y, Yu Z, Chen J, Han JJ. Assessing the rate of aging to monitor aging itself. Ageing Res Rev. 2021;69:101350. Epub 2021/05/04. doi: 10.1016/j.arr.2021.101350. PubMed PMID: 33940202.

15. Jylhava J, Pedersen NL, Hagg S. Biological Age Predictors. EBioMedicine. 2017;21:29-36. Epub 2017/04/12. doi: 10.1016/j.ebiom.2017.03.046. PubMed PMID: 28396265; PubMed Central PMCID: PMCPMC5514388.

16. Kwon D, Belsky DW. A toolkit for quantification of biological age from blood chemistry and organ function test data: BioAge. Geroscience. 2021;43(6):2795-808. Epub 2021/11/03. doi: 10.1007/s11357-021-00480-5. PubMed PMID: 34725754; PubMed Central PMCID: PMCPMC8602613.

17. Bortz J, Guariglia A, Klaric L, Tang D, Ward P, Geer M, et al. Biological age estimation using circulating blood biomarkers. Commun Biol. 2023;6(1):1089. Epub 2023/10/27. doi: 10.1038/s42003-023-05456-z. PubMed PMID: 37884697; PubMed Central PMCID: PMCPMC10603148 manuscript, P.K.J. and J.B. were paid consultants to Humanity Inc, a company focussed on measuring and developing interventions for Biological Age. L.K. was an employee of Humanity Inc. A.G. was formerly a paid consultant of Humanity Inc. M.G. and P.W. are founders of Humanity Inc and are employees and hold ordinary shares. P.K.J., M.G. and P.W. are partly remunerated under a Humanity Inc share option scheme.

P.K.J. is founder of Geromica, a consultancy providing advice on measurement of health and aging. M.C.-H. holds shares in the O-SMOSE company and has no conflict of interest to disclose. Consulting activities conducted by the company are independent of the present work. All other authors declare no competing interests.

18. Bischoff-Ferrari HA, Gangler S, Wieczorek M, Belsky DW, Ryan J, Kressig RW, et al. Individual and additive effects of vitamin D, omega-3 and exercise on DNA methylation clocks of biological aging in older adults from the DO-HEALTH trial. Nat Aging. 2025;5(3):376-85. Epub 2025/02/04. doi: 10.1038/s43587-024-00793-y. PubMed PMID: 39900648.

19. Centers for Disease Control and Prevention National Health and Nutrition Examination Survey Questionnaires D, and Related Documentation 2020. [cited 2025 March 7]. Available from: https://wwwn.cdc.gov/nchs/nhanes/.

20. Holick MF, Binkley NC, Bischoff-Ferrari HA, Gordon CM, Hanley DA, Heaney RP, et al. Evaluation, treatment, and prevention of vitamin D deficiency: an Endocrine Society clinical practice guideline. J Clin Endocrinol Metab. 2011;96(7):1911-30. Epub 2011/06/08. doi: 10.1210/jc.2011-0385. PubMed PMID: 21646368.

21. Liu Z, Kuo PL, Horvath S, Crimmins E, Ferrucci L, Levine M. A new aging measure captures morbidity and mortality risk across diverse subpopulations from NHANES IV: A cohort study. PLoS Med. 2018;15(12):e1002718. Epub 2019/01/01. doi: 10.1371/journal.pmed.1002718. PubMed PMID: 30596641; PubMed Central PMCID: PMCPMC6312200.

22. He H, Chen X, Ding Y, Chen X, He X. Composite dietary antioxidant index associated with delayed biological aging: a population-based study. Aging (Albany NY). 2024;16(1):15-27. Epub 2024/01/04. doi: 10.18632/aging.205232. PubMed PMID: 38170244; PubMed Central PMCID: PMCPMC10817368.

23. Liu W, Wang J, Wang M, Hou H, Ding X, Ma L, et al. Oxidative Stress Factors Mediate the Association Between Life's Essential 8 and Accelerated Phenotypic Aging: NHANES 2005-2018. J Gerontol A Biol Sci Med Sci. 2024;79(1). Epub 2023/10/10. doi: 10.1093/gerona/glad240. PubMed PMID: 37813096.

24. Bender R. Introduction to the use of regression models in epidemiology. Cancer epidemiology: Springer; 2009. p. 179-95.

25. Wood SN. Stable and efficient multiple smoothing parameter estimation for generalized additive models. Journal of the American Statistical Association. 2004;99(467):673-86.

26. Yucebilgic G, Bilgin R, Tamer L, Tukel S. Effects of lead on Na(+)-K(+) ATPase and Ca(+2) ATPase activities and lipid peroxidation in blood of workers. International journal of toxicology. 2003;22(2):95-7. doi: 10.1080/10915810305096. PubMed PMID: 12745990.

27. World Medical A. World Medical Association Declaration of Helsinki: Ethical Principles for Medical Research Involving Human Participants. JAMA. 2025;333(1):71-4. Epub 2024/10/19 20:42. doi: 10.1001/jama.2024.21972. PubMed PMID: 39425955.

28. Amrein K, Scherkl M, Hoffmann M, Neuwersch-Sommeregger S, Kostenberger M, Tmava Berisha A, et al. Vitamin D deficiency 2.0: an update on the current status worldwide. Eur J Clin Nutr. 2020;74(11):1498-513. Epub 2020/01/22. doi: 10.1038/s41430-020-0558-y. PubMed PMID: 31959942; PubMed Central PMCID: PMCPMC7091696 The other authors declare that they have no conflict of interest.

29. Liu W, Hu J, Fang Y, Wang P, Lu Y, Shen N. Vitamin D status in Mainland of China: A systematic review and meta-analysis. EClinicalMedicine. 2021;38:101017. Epub 2021/07/27. doi: 10.1016/j.eclinm.2021.101017. PubMed PMID: 34308318; PubMed Central PMCID:

Formatted: Font: (Default) Times New Roman

Formatted: Font: (Default) Times New Roman

PMCPMC8283334.

30. Bouillon R, Manousaki D, Rosen C, Trajanoska K, Rivadeneira F, Richards JB. The health effects of vitamin D supplementation: evidence from human studies. Nat Rev Endocrinol. 2022;18(2):96-110. Epub 2021/11/25. doi: 10.1038/s41574-021-00593-z. PubMed PMID: 34815552; PubMed Central PMCID: PMCPMC8609267 Farma, Ceres and Proctor and Gamble. J.B.R. has worked as a consultant to GlaxoSmithKline and Deerfield Capital. The other authors declare no competing interests.

31. Schottker B, Hagen L, Zhang Y, Gao X, Holleczek B, Gao X, et al. Serum 25-Hydroxyvitamin D Levels as an Aging Marker: Strong Associations With Age and All-Cause Mortality Independent From Telomere Length, Epigenetic Age Acceleration, and 8-Isoprostane Levels. J Gerontol A Biol Sci Med Sci. 2019;74(1):121-8. Epub 2018/10/30. doi: 10.1093/gerona/gly253. PubMed PMID: 30371905.

32. Liu L, Chen C, Lo K, Huang J, Yu Y, Huang Y, et al. Serum 25-hydroxyvitamin D, frailty, and mortality among the Chinese oldest old: Results from the CLHLS study. Nutr Metab Cardiovasc Dis. 2021;31(9):2707-15. Epub 2021/08/06. doi: 10.1016/j.numecd.2021.05.033. PubMed PMID: 34348876.

33. Veldurthy V, Wei R, Oz L, Dhawan P, Jeon YH, Christakos S. Vitamin D, calcium homeostasis and aging. Bone Res. 2016;4:16041. Epub 2016/10/30. doi: 10.1038/boneres.2016.41. PubMed PMID: 27790378; PubMed Central PMCID: PMCPMC5068478.

34. Ge D, Chen Q, Xie X, Li Q, Yang Y. Unveiling the potent effect of vitamin D: harnessing Nrf2/HO-1 signaling pathways as molecular targets to alleviate urban particulate matter-induced asthma inflammation. BMC Pulm Med. 2024;24(1):55. Epub 2024/01/26. doi: 10.1186/s12890-024-02869-2. PubMed PMID: 38273268; PubMed Central PMCID: PMCPMC10809564.

35. Pilz S, Gaksch M, Kienreich K, Grubler M, Verheyen N, Fahrleitner-Pammer A, et al. Effects of vitamin D on blood pressure and cardiovascular risk factors: a randomized controlled trial. Hypertension. 2015;65(6):1195-201. Epub 2015/03/25. doi: 10.1161/HYPERTENSIONAHA.115.05319. PubMed PMID: 25801871.

36. Chowdhury R, Kunutsor S, Vitezova A, Oliver-Williams C, Chowdhury S, Kiefte-de-Jong JC, et al. Vitamin D and risk of cause specific death: systematic review and meta-analysis of observational cohort and randomised intervention studies. BMJ. 2014;348:g1903. Epub 2014/04/03. doi: 10.1136/bmj.g1903. PubMed PMID: 24690623; PubMed Central PMCID: PMCPMC3972416 at www.icmje.org/coi_disclosure.pdf (available on request from the corresponding authors) and declare: no support from any organisation for the submitted work; no financial relationships with any organisations that might have an interest in the submitted work in the previous three years; no other relationships or activities that could appear to have influenced the submitted work.

37. Rejnmark L, Bislev LS, Cashman KD, Eiriksdottir G, Gaksch M, Grubler M, et al. Non-skeletal health effects of vitamin D supplementation: A systematic review on findings from meta-analyses summarizing trial data. Plos One. 2017;12(7):e0180512. Epub 2017/07/08. doi: 10.1371/journal.pone.0180512. PubMed PMID: 28686645; PubMed Central PMCID: PMCPMC5501555.

38. LeBoff MS, Chou SH, Ratliff KA, Cook NR, Khurana B, Kim E, et al. Supplemental Vitamin D and Incident Fractures in Midlife and Older Adults. N Engl J Med. 2022;387(4):299-309. Epub 2022/08/09. doi: 10.1056/NEJMoa2202106. PubMed PMID: 35939577; PubMed Central PMCID: PMCPMC9716639.

39. Pludowski P, Grant WB, Karras SN, Zittermann A, Pilz S. Vitamin D Supplementation: A Review of the Evidence Arguing for a Daily Dose of 2000 International Units (50 microg) of Vitamin D for Adults in the General Population. Nutrients. 2024;16(3). Epub 2024/02/10. doi: 10.3390/nu16030391.

Formatted: Font: (Default) Times New Roman

Formatted: Font: (Default) Times New Roman

PubMed PMID: 38337676; PubMed Central PMCID: PMCPMC10857599.
40. Marcinowska-Suchowierska E, Kupisz-Urbanska M, Lukaszkiewicz J, Pludowski P, Jones G. Vitamin D Toxicity-A Clinical Perspective. Front Endocrinol (Lausanne). 2018;9:550. Epub 2018/10/09. doi: 10.3389/fendo.2018.00550. PubMed PMID: 30294301; PubMed Central PMCID: PMCPMC6158375.
41. Batman A, Saygili ES, Yildiz D, Sen EC, Erol RS, Canat MM, et al. Risk of hypercalcemia in patients with very high serum 25-OH vitamin D levels. Int J Clin Pract. 2021;75(7):e14181. Epub 2021/03/25. doi: 10.1111/ijcp.14181. PubMed PMID: 33759301; PubMed Central PMCID: PMCPMC8250214.
42. Sajid U, Orton D, Kaufmann M, Jones G, Kline GA. Understanding Elevated Vitamin D Measurements to Uncover Hypercalcemia Etiology. Clin Chem. 2024;70(6):798-802. Epub 2024/06/03. doi: 10.1093/clinchem/hvae044. PubMed PMID: 38825340.
43. Santoro N, Roeca C, Peters BA, Neal-Perry G. The Menopause Transition: Signs, Symptoms, and Management Options. J Clin Endocrinol Metab. 2021;106(1):1-15. Epub 2020/10/24. doi: 10.1210/clinem/dgaa764. PubMed PMID: 33095879.
44. Crescioli C. The Role of Estrogens and Vitamin D in Cardiomyocyte Protection: A Female Perspective. Biomolecules. 2021;11(12). Epub 2021/12/25. doi: 10.3390/biom11121815. PubMed PMID: 34944459; PubMed Central PMCID: PMCPMC8699224.
45. McCurdy R, Jiang X, Clarkson TB, Nudy M, Schnatz PF. Vitamin D and conjugated equine estrogen: the association with coronary artery atherosclerosis in cynomolgus monkeys. Menopause. 2016;23(5):481-7. Epub 2016/02/18. doi: 10.1097/GME.0000000000000582. PubMed PMID: 26886883.

Table 1. Characteristics of the study population

Characteristics	Male (n=6836)	Female (n=7074)	*P*-value
Age (mean±SD)	57.07 (11.34)	58.12 (11.82)	<0.001
Race			0.001[b]
Non-Hispanic white	3249 (47.53)	3288 (46.48)	
Non-Hispanic black	1360 (19.89)	1417 (20.03)	
Others	2227 (32.58)	2369 (33.49)	
Education level			0.394[b]
Below high school	1847 (27.02)	1831 (25.88)	
High school or equivalent	1588 (23.23)	1597 (22.58)	
Above high school	3401 (49.75)	3646 (51.54)	
Poverty-income ratio			<0.001[b]
≤1.0	1199 (17.54)	1447 (20.46)	
1.0-3.0	2839 (41.53)	3029 (42.82)	
>3.0	2798 (40.93)	2598 (36.73)	
BMI, kg/m^2			<0.001[b]
<25	1585 (23.19)	1841 (26.02)	
25-30	2749 (40.21)	2117 (29.93)	
≥30	2502 (36.60)	3116 (44.05)	
Smoking			<0.001[b]
Never smoker	2823 (41.30)	4297 (60.74)	
Previous smoker	2570 (37.60)	1666 (23.55)	
Current smoker	1443 (21.11)	1111 (15.71)	
Drinking			<0.001[b]
Never drinker	527 (7.71)	1572 (22.22)	
Previous drinker	637 (9.32)	1466 (20.72)	
Current drinker	5672 (82.97)	4036 (57.05)	
Physical activity			<0.001[b]
Inactive	1775 (25.97)	2533 (35.81)	
Insufficient	802 (11.73)	1086 (15.35)	
Moderate	680 (9.95)	842 (11.90)	
High	3579 (52.36)	2613 (36.94)	
Sleep duration			<0.001[b]
<7 h	2572 (37.62)	2445 (34.56)	
7-8h	1858 (27.18)	1812 (25.61)	
≥8 h	2406 (35.20)	2817 (39.82)	
Hypertension			0.839[b]

No	3007 (43.99)	3067 (43.36)	
Yes	3829 (56.01)	4007 (56.64)	
Diabetes			<0.001[b]
No	5086 (74.40)	5515 (77.96)	
Yes	1750 (25.60)	1559 (22.04)	
Serum 25(OH)D, nmol/L			
Median (IQR)	63.80 (48.38, 80.20)	66.60 (47.00, 87.30)	<0.001[c]
<30	420 (6.14)	545 (7.70)	<0.001[b]
30-50	1451 (21.23)	1457 (20.60)	
50-75	2766 (40.46)	2293 (32.41)	
⩾75	2199 (32.17)	2779 (39.28)	
Phenoage (median [IQR])	53.77 (45.07, 64.60)	53.05 (44.23, 64.13)	0.032
~~Age advancement~~Physiological aging (median [IQR])	-2.40 (-5.13, 0.94)	-4.14 (-7.22, -0.46)	<0.001[c]
Standardized ~~age advancement~~physiological aging (mean±SD)	0.11 (0.95)	-0.16 (1.05)	<0.001[a]

BMI: body mass index; IQR: interquartile range; SD: standard deviation

Data were reported as number (percentage) for categorical variables; mean±SD or median (IQR) for continuous variables.

[a] Student's t-test was used to compare means of variables between two groups

[b] Chi-square test was used to compare categorical variables between two groups.

[c] Mann-Whitney test was used to compare non-normal variables between two groups.

Table 2. Association between serum 25(OH)D level and age advancementphysiological agingbiological aging

Characteristics	All: Estimated effectBeta coefficient (95% CI)			Male: Beta coefficientEstimated effect (95% CI)			Female: Beta coefficientEstimated effect (95% CI)		
	Crude model	Adjusted model [a]	*P*-value	Crude model	Adjusted model [a]	*P*-value	Crude model	Adjusted model [a]	*P*-value
Log(25(OH)D)	-0.27 (-0.32, -0.22)	-0.04 (-0.08, 0.00)	0.039	-0.13 (-0.20, -0.06)	0.03 (-0.02, 0.09)	0.334	-0.32 (-0.38, -0.26)	-0.07 (-0.12, -0.01)	0.007
Quartiles									
Q1	Reference	Reference		Reference	Reference		Reference	Reference	
Q2	-0.23 (-0.29, -0.17)	-0.10 (-0.16, -0.05)	<0.001	-0.16 (-0.24, -0.08)	-0.02 (-0.09, 0.04)	0.448	-0.33 (-0.41, -0.25)	-0.16 (-0.23, -0.08)	<0.001
Q3	-0.29 (-0.36, -0.23)	-0.10 (-0.16, -0.04)	0.001	-0.20 (-0.29, -0.11)	-0.03 (-0.12, 0.04)	0.355	-0.40 (-0.49, -0.32)	-0.15 (-0.22, -0.07)	<0.001
Q4	-0.32 (-0.38, -0.26)	-0.05 (-0.11, -0.00)	0.022	-0.16 (-0.24, -0.07)	0.02 (-0.04, 0.10)	0.553	-0.42 (-0.51, -0.34)	-0.10 (-0.18, -0.02)	0.009
P for trend			0.173			0.429			0.076
Categories based on the Endocrine Society									
<30	Reference	Reference		Reference	Reference		Reference	Reference	
30-50	-0.23 (-0.33, -0.13)	-0.14 (-0.23, -0.05)	0.002	-0.26 (-0.41, -0.11)	-0.15 (-0.29, -0.02)	0.024	-0.22 (-0.34, -0.09)	-0.10 (-0.21, 0.00)	0.057
50-75	-0.42 (-0.52, -0.33)	-0.21 (-0.30, -0.13)	<0.001	-0.39 (-0.53, -0.25)	-0.16 (-0.29, -0.04)	0.009	-0.51 (-0.63, -0.40)	-0.24 (-0.33, -0.15)	<0.001
⩾75	-0.46 (-0.56, -0.36)	-0.15 (-0.24, -0.06)	0.001	-0.36 (-0.50, -0.22)	-0.09 (-0.22, 0.03)	0.131	-0.54 (-0.66, -0.42)	-0.17 (-0.27, -0.07)	0.001
P for trend			0.130			0.366			0.014

Formatted: Font color: Text 1
Formatted: Font color: Custom Color(RGB(3,30,255))
Formatted: Font color: Custom Color(RGB(3,30,255))
Formatted: Font color: Custom Color(RGB(3,30,255))
Formatted: Font color: Custom Color(RGB(3,30,255))
Formatted: Font color: Custom Color(RGB(3,30,255))

[a] Adjusted for age, race, education level, poverty-income ratio, body mass index, smoking, drinking, physical activity, sleep duration, hypertension, diabetes and season of measurement.

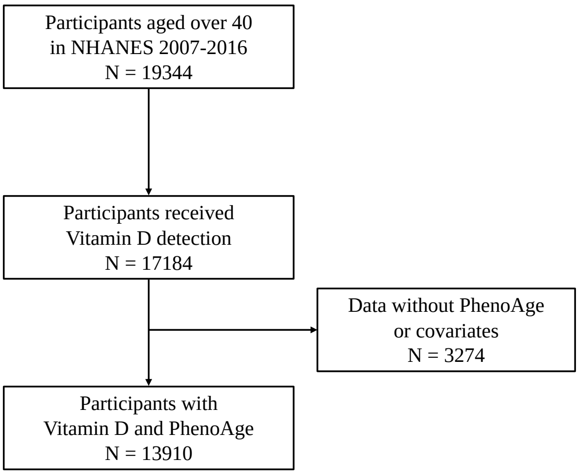

Figure 1 Flowchart of participants selection.

Formatted: Font: Bold

A

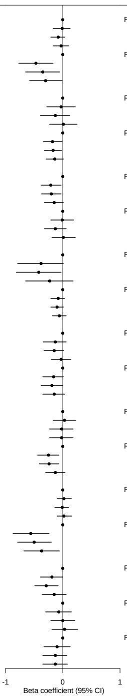

Subgroups	Categories	Beta coefficient (95% CI)	*P*-value	*P* for interaction
**Age**				0.013
<60 y	<30	Reference	Reference	
	30-50	-0.01 (-0.17, 0.13)	0.776	
	50-75	-0.08 (-0.21, 0.03)	0.130	
	>=75	-0.03 (-0.17, 0.10)	0.618	
≥60 y	<30	Reference	Reference	
	30-50	-0.47 (-0.77, -0.17)	0.002	
	50-75	-0.35 (-0.65, -0.05)	0.021	
	>=75	-0.30 (-0.58, -0.01)	0.039	
**BMI**				0.119
<25.00	<30	Reference	Reference	
	30-50	-0.03 (-0.28, 0.22)	0.766	
	50-75	-0.13 (-0.39, 0.12)	0.289	
	>=75	0.01 (-0.23, 0.25)	0.950	
≥25.00	<30	Reference	Reference	
	30-50	-0.18 (-0.34, -0.01)	0.028	
	50-75	-0.17 (-0.32, -0.02)	0.022	
	>=75	-0.14 (-0.29, 0.01)	0.077	
**Smoking status**				0.193
No	<30	Reference	Reference	
	30-50	-0.21 (-0.38, -0.03)	0.018	
	50-75	-0.20 (-0.37, -0.03)	0.020	
	>=75	-0.15 (-0.32, 0.01)	0.066	
Yes	<30	Reference	Reference	
	30-50	-0.01 (-0.21, 0.19)	0.885	
	50-75	-0.13 (-0.32, 0.06)	0.179	
	>=75	0.01 (-0.19, 0.22)	0.897	
**Driniking status**				0.252
No	<30	Reference	Reference	
	30-50	-0.38 (-0.79, 0.01)	0.063	
	50-75	-0.42 (-0.81, -0.03)	0.033	
	>=75	-0.23 (-0.65, 0.18)	0.266	
Yes	<30	Reference	Reference	
	30-50	-0.08 (-0.21, 0.03)	0.151	
	50-75	-0.10 (-0.21, 0.01)	0.080	
	>=75	-0.06 (-0.18, 0.06)	0.310	
**Physical activity**				0.169
Inactive or insufficient	<30	Reference	Reference	
	30-50	-0.13 (-0.34, 0.06)	0.180	
	50-75	-0.15 (-0.33, 0.02)	0.085	
	>=75	-0.03 (-0.20, 0.14)	0.701	
Moderate or high	<30	Reference	Reference	
	30-50	-0.16 (-0.35, 0.01)	0.073	
	50-75	-0.19 (-0.38, 0.00)	0.052	
	>=75	-0.15 (-0.35, 0.03)	0.111	
**Hypertension**				0.044
No	<30	Reference	Reference	
	30-50	0.03 (-0.17, 0.23)	0.812	
	50-75	-0.02 (-0.23, 0.18)	0.786	
	>=75	-0.02 (-0.23, 0.18)	0.794	
Yes	<30	Reference	Reference	
	30-50	-0.25 (-0.44, -0.07)	0.007	
	50-75	-0.24 (-0.41, -0.07)	0.004	
	>=75	-0.13 (-0.30, 0.04)	0.125	
**Diabetes**				0.011
No	<30	Reference	Reference	
	30-50	0.02 (-0.10, 0.15)	0.716	
	50-75	-0.01 (-0.14, 0.10)	0.749	
	>=75	0.02 (-0.10, 0.16)	0.751	
Yes	<30	Reference	Reference	
	30-50	-0.56 (-0.87, -0.24)	0.001	
	50-75	-0.50 (-0.79, -0.20)	0.001	
	>=75	-0.37 (-0.68, -0.06)	0.020	
**Sleep duration**				0.240
<7 h	<30	Reference	Reference	
	30-50	-0.19 (-0.39, 0.00)	0.054	
	50-75	-0.29 (-0.49, -0.08)	0.006	
	>=75	-0.15 (-0.36, 0.06)	0.161	
7-8 h	<30	Reference	Reference	
	30-50	-0.07 (-0.30, 0.15)	0.490	
	50-75	-0.00 (-0.21, 0.21)	0.957	
	>=75	0.03 (-0.19, 0.26)	0.786	
≥8 h	<30	Reference	Reference	
	30-50	-0.10 (-0.33, 0.13)	0.387	
	50-75	-0.13 (-0.35, 0.07)	0.201	
	>=75	-0.13 (-0.35, 0.08)	0.224	

B

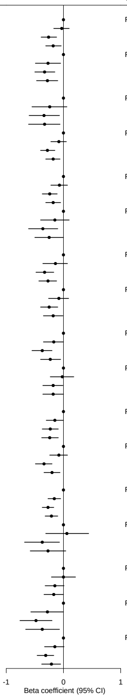

Subgroups	Categories	Beta coefficient (95% CI)	*P*-value	*P* for interaction
**Age**				0.108
<60 y	<30	Reference	Reference	
	30-50	-0.03 (-0.17, 0.10)	0.615	
	50-75	-0.26 (-0.39, -0.12)	<0.001	
	>=75	-0.18 (-0.31, -0.04)	0.010	
≥60 y	<30	Reference	Reference	
	30-50	-0.27 (-0.49, -0.05)	0.016	
	50-75	-0.33 (-0.50, -0.15)	<0.001	
	>=75	-0.28 (-0.47, -0.10)	0.003	
**BMI**				0.445
<25.00	<30	Reference	Reference	
	30-50	-0.24 (-0.55, 0.06)	0.124	
	50-75	-0.34 (-0.60, -0.07)	0.014	
	>=75	-0.33 (-0.61, -0.06)	0.018	
≥25.00	<30	Reference	Reference	
	30-50	-0.08 (-0.22, 0.05)	0.200	
	50-75	-0.28 (-0.40, -0.15)	<0.001	
	>=75	-0.18 (-0.31, -0.06)	0.004	
**Smoking status**				0.922
No	<30	Reference	Reference	
	30-50	-0.07 (-0.22, 0.07)	0.299	
	50-75	-0.24 (-0.37, -0.11)	<0.001	
	>=75	-0.18 (-0.31, -0.05)	0.007	
Yes	<30	Reference	Reference	
	30-50	-0.15 (-0.40, 0.10)	0.226	
	50-75	-0.36 (-0.61, -0.10)	0.006	
	>=75	-0.25 (-0.50, 0.00)	0.054	
**Driniking status**				0.950
No	<30	Reference	Reference	
	30-50	-0.14 (-0.36, 0.07)	0.175	
	50-75	-0.33 (-0.48, -0.17)	<0.001	
	>=75	-0.27 (-0.43, -0.12)	0.001	
Yes	<30	Reference	Reference	
	30-50	-0.08 (-0.26, 0.09)	0.328	
	50-75	-0.25 (-0.40, -0.10)	0.002	
	>=75	-0.18 (-0.35, -0.01)	0.036	
**Physical activity**				0.081
Inactive or insufficient	<30	Reference	Reference	
	30-50	-0.17 (-0.35, -0.00)	0.043	
	50-75	-0.37 (-0.55, -0.20)	<0.001	
	>=75	-0.23 (-0.40, -0.05)	0.010	
Moderate or high	<30	Reference	Reference	
	30-50	-0.02 (-0.23, 0.18)	0.768	
	50-75	-0.18 (-0.36, -0.01)	0.035	
	>=75	-0.18 (-0.36, -0.00)	0.046	
**Hypertension**				0.045
No	<30	Reference	Reference	
	30-50	-0.15 (-0.30, 0.00)	0.049	
	50-75	-0.23 (-0.37, -0.09)	0.001	
	>=75	-0.24 (-0.38, -0.09)	0.001	
Yes	<30	Reference	Reference	
	30-50	-0.08 (-0.24, 0.07)	0.261	
	50-75	-0.34 (-0.49, -0.20)	<0.001	
	>=75	-0.20 (-0.34, -0.06)	0.004	
**Diabetes**				0.092
No	<30	Reference	Reference	
	30-50	-0.16 (-0.27, -0.05)	0.003	
	50-75	-0.27 (-0.37, -0.17)	<0.001	
	>=75	-0.21 (-0.32, -0.10)	<0.001	
Yes	<30	Reference	Reference	
	30-50	0.06 (-0.31, 0.44)	0.757	
	50-75	-0.37 (-0.68, -0.07)	0.017	
	>=75	-0.27 (-0.58, 0.04)	0.088	
**Sleep duration**				0.457
<7 h	<30	Reference	Reference	
	30-50	-0.00 (-0.21, 0.21)	0.944	
	50-75	-0.15 (-0.32, 0.01)	0.073	
	>=75	-0.17 (-0.34, -0.00)	0.046	
7-8 h	<30	Reference	Reference	
	30-50	-0.28 (-0.57, -0.00)	0.046	
	50-75	-0.48 (-0.76, -0.20)	0.001	
	>=75	-0.37 (-0.66, -0.07)	0.015	
≥8 h	<30	Reference	Reference	
	30-50	-0.15 (-0.33, 0.01)	0.075	
	50-75	-0.31 (-0.46, -0.17)	<0.001	
	>=75	-0.21 (-0.38, -0.05)	0.011	

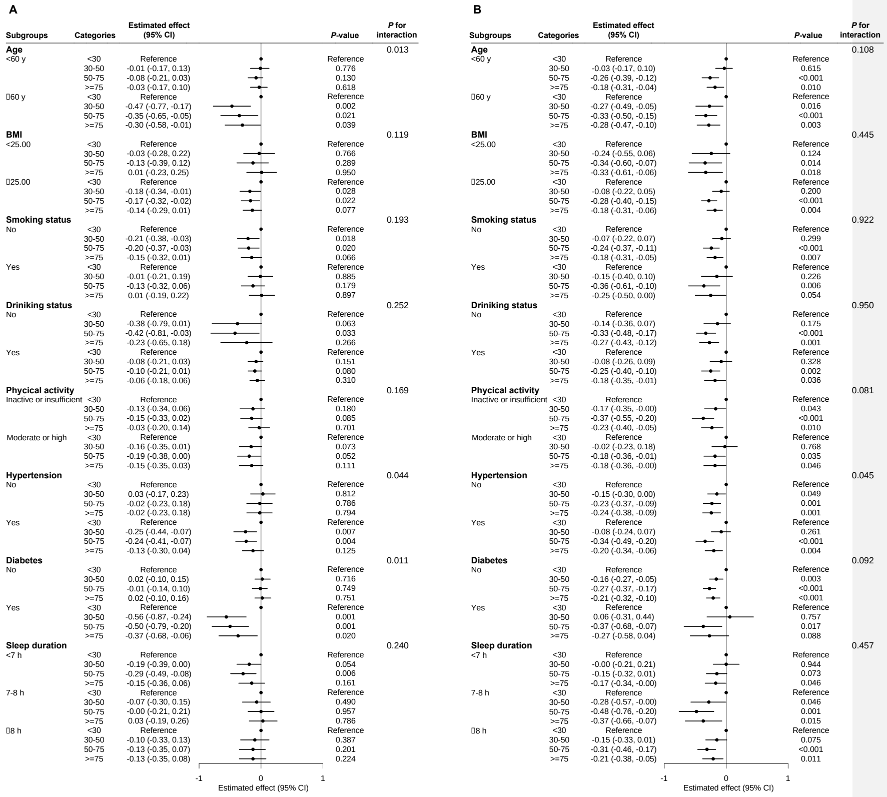

Figure ~~1~~ 2 Subgroup analyses of the association between 25(OH)D categories and ~~age advancement~~physiological aging~~biological aging~~ among males (A) and females (B). The filled circles and horizontal lines represent regression coefficients and the 95% confidence intervals. Multiple linear regression models were adjusted for age, race, education level, poverty-income ratio, body mass index, smoking, drinking, physical activity, sleep duration, hypertension, diabetes and season of measurement. BMI, body mass index; CI, confidence intervals.

Formatted: Font color: Custom Color(RGB(3,30,255))

Formatted: Font color: Custom Color(RGB(3,30,255))

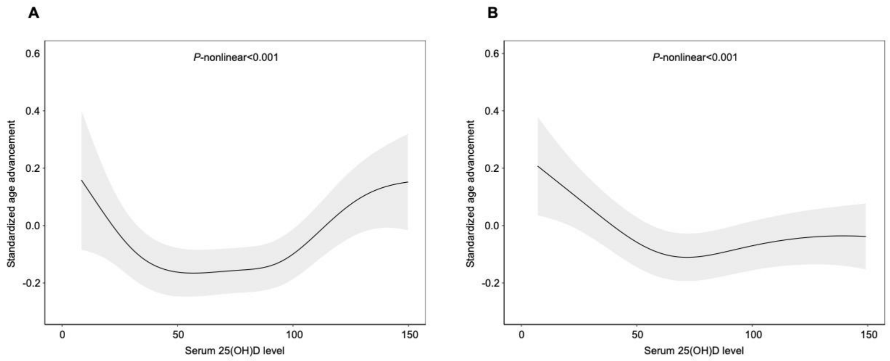

Figure ~~2~~ 3 Nonlinear relationships between serum 25(OH)D level and ~~biological aging~~physiological aging~~age advancement~~physiological aging among males (A) and females (B). Generalized additive models were adjusted for age, race, education level, poverty-income ratio, body mass index, smoking, drinking, physical activity, sleep duration, hypertension, diabetes and season of measurement. The black solid line represents the smooth curve fit between variables. The grey band represents the 95% confidence interval of the fit.

Formatted: Font color: Custom Color(RGB(3,30,255))

Table 3. Threshold effect analysis of serum 25(OH)D level and ~~biological aging~~physiological aging ~~advancement~~

Serum 25(OH)D levels	Beta coefficient~~Estimated effect~~ (95% CI)	*P*-value
Male		
<38.20	-0.013 (-0.025, -0.002)	0.017
38.20-91.55	0.001 (-0.001, 0.002)	0.487
⩾91.55	0.005 (0.002, 0.008)	<0.001
Female		
<62.50	-0.007 (-0.010, -0.004)	<0.001
62.50-125.00	0.002 (0.000, 0.004)	0.029
⩾125.00	-0.001 (-0.005, 0.002)	0.535

CI: confidence intervals

Segmented regression models were adjusted for age, race, education level, poverty-income ratio, body mass index, smoking, drinking, physical activity, sleep duration, hypertension, diabetes and season of measurement.

Formatted: Font color: Custom Color(RGB(3,30,255))

Dear editor and reviewer:

Thank you very much for your careful review and constructive suggestions with regard to our manuscript. Those comments are helpful for us to revise and improve our paper. We have studied comments carefully and tried our best to revise and improve the manuscript and made great changes in the manuscript according to the good comments. We earnestly appreciate for the warm work from you, and hope that the corrections will meet with approval. Please feel free to contact us with any questions and we are looking forward to your consideration. The main corrections in the paper and the responds to your comments are as following:

Additional Editor Comments:

In addition to the reviewer's comments, I have the following comments:

1. **In the title, you would better replace "age advancement" with "biological aging" or "physiological aging" given that PhenoAge used as metric of aging in this study is a measure of biological/physiological aging.**

Response: Thank you for your insightful feedback. We have revised the title to use "biological aging" instead of "age advancement" to more accurately reflect our study's focus on the biological aspects measured by the PhenoAge metric.

2. **The authors should revise the "Materials and methods", results and discussion sections based on the STROBE guidelines statement (see von Elm et al. PLoS Med. 2007;4(10):e296) to allow for the reproducibility of their methods. Accordingly, they should start the "Materials and methods section" stating that their report conforms to STROBE guidelines for observational studies, and upload a STROBE checklist in the appendix. The "Statistical analysis" sub-section should follow the SAMPL guideline statement (see Lang TA et al. Medical Writing.2016;25(3):31-36) and the authors should upload a statistical analysis plan for this study as well as raw data (if legal frameworks of NHANES allow that) as supplemental materials to this manuscript. This recommendation aims to improve the readability of their manuscript, allow the credibility and reproducibility of their study methods, and increase the credibility of their findings and trustworthiness of the interpretation of their findings.**

Response: Thank you for your constructive suggestions and guidance. We have thoroughly revised the manuscript to ensure alignment with STROBE guidelines for observational studies, as well as the SAMPL recommendations for statistical reporting. Meanwhile, in the Materials and methods section, we supplemented the following statement: "This study was conducted and reported in adherence to STROBE guidelines for observational studies." (marked in blue, Page 4, Lines 95). The completed STROBE checklist is provided in Supplementary materials. A statistical analysis plan has also been written and provided in Supplementary materials. Regarding data accessibility, all raw data was publicly available through the NHANES website: https://wwwn.cdc.gov/nchs/nhanes/Default.aspx. According to the NHANES data use agreement, redistribution of raw datasets is prohibited. Therefore,

we are unable to provide NHANES raw data directly. This has been clarified in the Materials and methods section: "All data included in current study are publicly available through the NHANES website [18]." (marked in blue, Page 5, Lines 99).

The following reference was supplemented in the References List:

19. Centers for Disease Control and Prevention National Health and Nutrition Examination Survey Questionnaires D, and Related Documentation 2020. [cited 2025 March 7]. Available from: https://wwwn.cdc.gov/nchs/nhanes/.

3. **The "Materials and methods" section should end with a sub-section titled "Ethical considerations"in which the authors should state whether they abode by the ethical rules for conducting medical/health research (see the 2024 declaration of Helsinki statement published in JAMA), how they accessed NHANES (USA) data whereas all authors are affiliated with China institutes and the study was funded by a Chinese organization). Did they get an IRB approval to use the NHANES dataset?**

Response: We thank the editor for your constructive suggestions and guidance. Our research adhered to the ethical principles in the 2024 Declaration of Helsinki regarding secondary analysis. It exempts studies from additional ethical review when utilizing pre-existing de-identified data [1]. Meanwhile the NHANES datasets we used were publicly available and did not contain restricted use datasets. According to the CDC/NCHS Research Ethics Review Board statement, researchers using only the publicly available data files do not need to seek IRB approval [2]. Hence, no additional IRB was required in our study.

In the Materials and methods section, we have supplemented the "Ethical considerations" sub-section in the revised manuscript: "This study utilized data from NHANES study, which was conducted in accordance with the principles in the Declaration of Helsinki. The NHANES protocol received prior ethical approval from the National Center for Health Statistics' Review Board. Since our study is a secondary analysis of publicly accessible, de-identified NHANES data sets, additional institutional review board approval was not required [26, 27]." (marked in blue, Page 7, Lines186).

The following reference was supplemented in the References List:

26. University of Connecticut. Guidance on secondary analysis of existing data sets. [cited 2025 Feb 5]. Available from: https://ovpr.uconn.edu/services/rics/irb/researcher-guide/secondary-analysis-of-data-sets/.

27. World Medical A. World Medical Association Declaration of Helsinki: Ethical Principles for Medical Research Involving Human Participants. JAMA. 2025;333(1):71-4. Epub 2024/10/19 20:42. doi: 10.1001/jama.2024.21972. PubMed PMID: 39425955.

4. **In the conflicts of interest statement on the manuscript's agenda, the authors should clarify any potential conflict of interest with the funder and/or NHANES database managers.**

Response: We thank the editor for your guidance. We have revised the "**Conflict of interest**" statement in the manuscript as follows: "The authors declare no competing financial or non-

financial interests related to this work. NHANES is a publicly available dataset managed by the Centers for Disease Control and Prevention. The authors confirm that there is no affiliation with or involvement from the NHANES program administrators in this study. The funding source had no role in study design, data collection, analysis, or manuscript preparation.”**.**

5. **It is difficult to know in tables and figures what type of effect magnitude estimates (odds ratios? Others?) the authors base their conclusions on and whether those estimates are those that should be used in cross-sectional studies to assess associations: see Thiese MS. Observatiobal and interventional study design types; an overview. Biochemistry Med (Zagreb).2014;24(2):199-210. Did you perform logistic regression analyses and/ or correlation analyses? Why perform both or why perform one of them? These clarifications need to be made in the statistical analysis sub-section of the "Materials and methods" section. Along these lines, I would like to remember that the authors overstated their conclusions (see Schooling MC and Heidi JE. Clarifying questions about "risk factors": predictors versus explanation. Epidemiology in Themes. 2018;15:10), and this should be corrected throughout the manuscript.**

Response: We sincerely thank the editor for your guidance. Regarding the choice of effect estimates, our analyses utilized beta coefficients derived from weighted multivariable linear regression models. According to Thiese et al., odds ratios are standard for binary outcomes in logistic regression. However, when measuring continuous response variables, beta coefficients are recommended as they quantify linear association between continuous variables. To improve the clarity of our manuscript, we supplemented the following sentences in the Materials and methods section: “Beta coefficients with 95% confidence intervals (CIs) were reported, representing the change in dependent variable per unit increase in the independent variable. The use of beta coefficients provides interpretable estimates for linear associations when analyzing continuous dependent variables, which is widely recommended in epidemiological studies.” (marked in blue, Page 7). Meanwhile, we made adjustment to all Tables and Figures, as well as description of results to ensure the use of beta coefficient is straightforward.

We also supplemented the following sentences in the Materials and methods section to explain why correlation analysis is omitted in current study: “Correlation analysis was not employed because it does not account for confounding factors, whereas the primary aim of current study is to estimate the confounder-adjusted association between 25(OH)D status and PhenoAge aging.” (marked in blue, Page 7).

The following reference was supplemented in the References List:

24. Bender R. Introduction to the use of regression models in epidemiology. Cancer epidemiology: Springer; 2009. P. 179-95

Regarding the potential overstatement of conclusion, we have revised the manuscript

following the guidance of Schooling and Heidi (2018). Adjustments have been made throughout the Abstract, Results, and Discussion and Conclusion sections to avoid formative and causal language.

6. **The authors cite res 4 to 6 for the definition of vitamin D deficiency but use ref 18 for the definition of vitamin D deficiency in this study. Why can't they use only reference 6 in the introduction and for the definition in this study given that it is the most recently published article? Can you specify whether you assessed the human form of 25-OH-D (25-OH-D3) throughout the manuscript?**

Response: We thank the editor for your careful reading and constructive suggestions. We realize that the previous version of the manuscript cited multiple references regarding the deficiency of vitamin D level, which induced redundancy. We agree that ref 6 is the latest and authoritative source for defining vitamin D status. Therefore, we have unified citation of ref 6 in both introduction and methods sections, meanwhile remove ref 4-5 and 18 from our revised manuscript.

Regarding the detected form of 25-OH-D, we supplemented the following sentences in the Methods to improve the clarity of our manuscript: "From cycle 2007-2008 and beyond, liquid chromatography-tandem mass spectrometry (LC-MS/MS) was used to measure serum 25(OH)D concentrations, including 25(OH)D3, epi-25(OH)D3, and 25(OH)D2 in human serum. We calculated the 25(OH)D levels by adding 25(OH)D3 and 25(OH)D2 levels." (marked in blue, Page 5).

7. **After reading your introduction, I still do not well understand the rationale for conducting this study given that as well mentioned in reference 6 of the current manuscript, it is largely known that older individuals generally have lower vitamin D levels than younger populations. Do you want to asses whether that knowledge based on social age could hold even when using biological aging metrics (taking into consideration the discrepancy between social and biological ages)? The aim and rationale for conducting the study should be unambiguous in the last paragraph of the introduction. Still regarding the introduction, it is important to begin to be more cautious and specific when making claims about the involvement of vitamin D in human disease based on epidemiological data (see Vitamin D and human healrh: evidence from Mendelian randomization studies. Eur J Epidemiol. 2024;39: 467-490 and Schooling MC and Jones HE. Clarifying questions about "risk factors": predictors versus explanation. Epidemiology in Themes. 2018; 15:10). From my perspective, in a study whereby you seemingly assess the epidemiological link between vitamin D and physiological aging, the introduction should (in a brief overview form: introduction of no more than 1.5 pages) highlight how the human form of vitamin D evolves throughout the age spectrum based on the most certain available peer-reviewed evidence, as well as what is known and what is unknown about the epidemiological association between the human form of vitamin D and**

physiological aging, before stating the rationale for conducting the study (gaps in knowledge you would like to fill) and the study's aim.

Response: Thank you for your feedback regarding the introduction section of our manuscript. We have rewritten the introduction section to make it clear and concise in the revised manuscript. Briefly, the aim of current study is to investigate the relationship between vitamin D and physiological aging. We have read the articles mentioned above and rewrite the introduction of vitamin D in human disease based on epidemiological data. The length of introduction has been limited to less than 2 pages, a little longer than 1.5 pages. We have done our best to improve the induction section and hope this improvement could meet your requirement. Thank you very much.

8. Finally, language editing is also required.

Response: Thank you for your valuable feedback and suggestions for improvement. I acknowledge the need for language editing to enhance the clarity, precision, and professionalism of the manuscript. I will thoroughly review and ask help from professional language improvement to ensure that the text is clear and effectively conveys the intended meaning.

Reviewer #1:

Feng et al conduct this research in a rare direction while most of researchers will focus on the association between 25(OH)D and disease. Here are some issues should be concerned.

1. **In the abstract, there is no statistical results. The conclusion that VD deficiency promotes aging is not proper since cross sectional study only remind the possible association between exposure and outcome instead of causuality.**

Response: Thank you for your thoughtful feedback. We have revised the abstract to include key statistical results to better inform readers about the study's findings.
Additionally, we acknowledge your point regarding the limitations of cross-sectional studies in establishing causality. You are correct that such studies can only identify associations, and we will revise the language in the abstract and manuscript to reflect this appropriately. Specifically, we will modify the conclusion to state that vitamin D deficiency "may be associated with" or "shows a potential link to" biological aging, rather than implying causation.

2. **The introduction section is a little long, in which 3-4 paragraphes can be better.**

Response: Thank you for your suggestion. We acknowledge that the current introduction may be too lengthy and appreciate your suggestion to condense it into 3-4 focused paragraphs.

3. **In methods section, data including flow chart should be added, and please learn some from the below articles:**
a. Possible non-linear relation between prostate specific antigen and vitamin D: a machine learning study based on cross-section data
b. Association between urinary metals and prostate-specific antigen in aging population with depression: a cross-sectional study

Response: We thank the reviewer for your constructive suggestion. We have supplemented a flowchart that outline the participant selection process as Figure 1 in revised manuscript.

4. **In the discussion section, this study should declare the mechanism in the increase of 25(OH)D acclerate aging.**

Response: Thank you for your suggestion. We have rewritten the discussion section, providing the mechanism how the increase of 25(OH)D accelerate aging. "These detrimental effects may stem from the toxicity of excessive vitamin D. When vitamin D intake is too high, serum levels of its metabolites (25(OH)D and 24,25$(OH)_2$D) rise and surpass the binding capacity of vitamin D binding protein. This leads to an increase in the free active metabolite, 1,25$(OH)_2$D. This active form reaches the vitamin D receptor (VDR) in the nucleus, promoting gene expression that enhances calcium absorption and bone mobilization. Consequently, this can result in hypercalcemia or hyperphosphatemia, which in turn suppresses initial parathyroid hormone (iPTH) levels. These disrupting balance of calcium and hormone may lead to conditions such as cardiovascular disease, kidney damage, and skin aging. Research reported that the working diagnosis of vitamin D toxicity was determined based on total concentrations of 25(OH)D at 32~80ng/mL (80~200 nmol/L) detected by immunoassay. Our results found the 25(OH)D levels above125 nmol/L in female participants were linked to accelerated physiological aging, approximately consistent with those reports." (marked in blue, Page 12)

5. **Except the increase of 25(OH)D acclerate aging, if there are some literatures reporting aging can increase 25(OH)D. Maybe it is hard to explain the causality of the interactive association between 25(OH)D and aging. Please make this part clear.**

Response: The relationship between 25(OH)D levels and aging is complex and bidirectional, making it challenging to establish a clear cause-and-effect relationship in our cross-sectional study. It is widely accepted that the level of vitamin D decrease as people get old, and people grow awareness of vitamin D deficiency and related health problems, vitamin D became a popular supplement, and its use has increased markedly. However, an increased intake of vitamin D supplements by the general population and a growing number of prescriptions of therapeutic doses without medical monitoring might result in a greater risk of excessive vitamin D toxicity. We have added some words to make this part clear in the discussion section. Thank you so much for your suggestion.

6. **Language should be revised. Like in conclusion, "These relationships exhibited nonlinearity in both males and females.". It is not clear between what.**

Response: Thank you for your valuable feedback and suggestions for improvement. I acknowledge the need for language editing to enhance the clarity, precision, and professionalism of the manuscript. I will thoroughly review and ask help from professional language improvement to ensure that the text is clear.

Reference

1. World Medical A. World Medical Association Declaration of Helsinki: Ethical Principles for Medical Research Involving Human Participants. JAMA. 2025;333(1):71-4. Epub 2024/10/19 20:42. doi: 10.1001/jama.2024.21972. PubMed PMID: 39425955.

2. NHANES Data Access Policy [cited 2025 feb 25]. Available from: https://www.cdc.gov/nchs/policy/data-user-agreement.html.
