# Supplementary material for: Association of 25(OH)D serum level with biological aging: A Cross-Sectional Study of 2007–2016 NHANES surveys
Source: PLoS One. 2025 Aug 6;20(8):e0328107. doi: 10.1371/journal.pone.0328107 (PMC12327616; doi:10.1371/journal.pone.0328107)
Supplement: Attachment — Submitted filename: PONE-D-24-25180_R2_Mickael Essouma comments and edits.pdf [file pone.0328107.s008.pdf]

PLOS ONE

Association of 25(OH)D serum level with biological aging: A Cross-Sectional Study of 2007-2016 NHANES surveys

--Manuscript Draft--

**Manuscript Number:**	PONE-D-24-25180R2
**Article Type:**	Research Article
**Full Title:**	Association of 25(OH)D serum level with biological aging: A Cross-Sectional Study of 2007-2016 NHANES surveys
**Short Title:**	aging and 25(OH)D serum level
**Corresponding Author:**	Jue Wang<br>nanhui new town community health service center<br>shanghai, CHINA
**Keywords:**	Vitamin D; Serum 25(OH)D; Biological aging; PhenoAge; Nonlinear association
**Abstract:**	Vitamin D plays a crucial role in various aspects of human body, including aging, but chronological age may not accurately reflect the true biological aging status. Recently, PhenoAge has been developed to estimate an individual's biological age based on different biological and clinical measures. Therefore, we investigated the relationship between 25(OH)D serum levels and biological aging calculated by PhenoAge using data from the 2007-2016 NHANES survey. 25(OH)D serum levels showed a negative association with PhenoAge acceleration (PhenoAgeAccel) (β=-0.04; 95% CI: -0.08 to 0.00, p=0.039). This negative correlation was only found to be dose-dependent for females (β=-0.07; 95% CI: -0.12 to 0.01, p=0.007), but not for males. Generalized additive models further showed nonlinear associations were revealing a U-shaped relationship observed in males but an L-shaped pattern in females. Specifically, 25(OH)D serum levels were linked to reduced PhenoAgeAccel at levels below 38.20 nmol/L (15.28 ng/mL) in males (β=-0.013; 95% CI: -0.025 to -0.002, p=0.017) and 62.50 nmol/L (25ng/mL) in females (β=-0.007; 95% CI: -0.01 to -0.004, p<0.001). However, 25(OH)D serum levels above 125 nmol/L were not associated with PhenoAgeAccel in females (β=-0.001; 95% CI: -0.005-0.002), while in males, elevated levels (>91.55 nmol/L) were associated with increased PhenoAgeAccel (β=0.005 SD; 95% CI: 0.002-0.008, p<0.001). Our findings indicate that vitamin D deficiency shows a potential link to accelerated biological aging, and high levels of vitamin D in males link to accelerated biological aging, offering valuable insights into the relationship between vitamin D and biological aging.
**Order of Authors:**	Yi Feng
	Licheng Yu
	Jue Wang
**Opposed Reviewers:**	
**Response to Reviewers:**	Dear editor and reviewers:<br>Thank you very much for your careful review and constructive suggestions with regard to our manuscript. Those comments are helpful for us to revise and improve our paper. We have studied comments carefully and tried our best to revise and improve the manuscript and made great changes in the manuscript according to the good comments. We earnestly appreciate for the warm work from you, and hope that the corrections will meet with approval. Please feel free to contact us with any questions and we are looking forward to your consideration. The main corrections in the paper and the responds to your comments are as following:<br><br>Additional Editor Comments:<br><br>The authors have improved the manuscript. However, as mentioned by the reviewers, there is a room for further improvement.<br><br>Suggestions about how to address the comments of Reviewer 5:

1. The terminologies "Physiological age" and "Biological age". Although the terminology "Physiological age" is known in the health literature to be based on some bodily functions as described in the article by Nakamura et al. Further evaluation of physical fitness age versus physiological age in women. Eur J Appl Physiol (1998) 78: 195-200 for example, the authors should indeed replace the terminology "Physiological age" with "biological age" where necessary because DNAm PhenoAge, the aging metric used in this study, is a composite of biomarkers, not bodily functions.
Response: we have replaced the "Physiological age" with "biological age". Thank you for this suggestion.

2. The race issue. The authors should stick to the racial stratification used in the 2007, 2008, 2009, 2010, 2011, 2012, 2013, 2014, 2015 and 2016 NHANES surveys. Indeed, this is not the first report on 25(OH)D assessment in a multiracial NHANES population as shown by the article Daraghmeh et al. Evidence for the vitamin D hypothesis: The NHANES III extended mortality follow-up. Atherosclerosis 255 (2016) 96e101. The lack of data segregation by race could be discussed as a common study limitation of NHANES surveys (https://pubmed.ncbi.nlm.nih.gov/?term=Vitamin+D+AND+NHANES) in the absence of race-specific cut-off values for vitamin D levels as for other biological biomarkers such as the creatinine-based estimated glomerular filtration rate, and when it is unclear whether 25-hydroxyvitamin serum levels ≥ 20 ng/mL are optimal across all racial groups in the USA: see The Journal of Clinical Endocrinology & Metabolism, Volume 109, Issue 8, August 2024, Pages 1948–1954, https://doi.org/10.1210/clinem/dgae322IF: 5.0 Q1 .
Response: the discussion of race has been added in the revised manuscript. thanks for your suggestion. (Page14, line 371-381)

3. The cut-off value of 25-hydroxyvitamin D for vitamin D deficiency. Given that the reviewer did not provide a reference to support the value of 25 nmol/L proposed, I recommend the authors stick to 20 ng/mL (50 nmol/L) as the cut-off value to define optimum and low levels of 25-hydroxyvitamin D in this study: see J Clin Endocrinol Metab. 2024 Jul 12;109(8):1907-1947. doi: 10.1210/clinem/dgae290IF: 5.0 Q1 . and The Journal of Clinical Endocrinology & Metabolism, Volume 109, Issue 8, August 2024, Pages 1948–1954, https://doi.org/10.1210/clinem/dgae322IF: 5.0 Q1 . However, the authors need to implement the reviewer's comment in their discussion of study limitations. In the same vein, the lack of genetic data on isoform of Vitamin D binding protein (VBD) should be discussed as a study limitation because VBD is allegedly the main determinant of the unbound 25-hydroxyvitamin D which is postulated to be the active compound of 25(OH)D (Bikle and Schwatrz. Vitamin D Binding Protein, Total and Free Vitamin D Levels in Different Physiological and Pathophysiological Conditions. Front Endocrinol (Lausanne). 2019 May 28:10:317. doi: 10.3389/fendo.2019.00317.).
Response: we have added a paragraph discussing the cut-off value of 25-hydroxyvitamin D for vitamin D deficiency. (Page 13, line 332-352)
“Regarding the threshold of vitamin D deficiency, it remains an ongoing controversy. The Scientific Advisory Committee on Nutrition (2016) recommended threshold at 25 nmol/L among UK population [46], whereas the IOM (2011) suggested thresholds at 30 nmol/L based on evidence in U.S. and Canadian populations [20]. Meanwhile, the European Food Safety Authority 2016 and the Endocrine Society guideline 2011 proposed higher level of 50 nmol/L as deficiency cutoff [47, 48]. The discrepancy in cutoff values stems from multiple factors, for example, different regional ultraviolet B exposure patterns, dietary practice and population-specific biological variability. Furthermore, guideline variations also reflect different focus on health outcomes, with some prioritizing musculoskeletal health [49]. In 2024, the Endocrine Society guideline retracted their previous recommendation of threshold at 50 nmol/L due to limited clinical trial evidence [7]. It also highlighted the impracticality of universal thresholds due to heterogeneity in health outcomes and population characteristics. Hence, the interpretation of 25(OH)D level threshold determination in our study should be with caution, especially when it derived from observational study in an ethnically diverse population. Nevertheless, the trend of benefits attenuation we observed is worth noting, suggesting excessive level of serum 25(OH)D may not be beneficial regarding biological aging process.”

4. Contemporary evidence about vitamin D association with disease. As mentioned in

	the first round of review and further highlighted by Reviewer 5, the authors need to exercise caution when stating that vitamin D serum concentrations are associated with diseases and that vitamin D supplementation is beneficial in disease prevention. For example, see the articles by Fang A, Zhao Y, Yang P, Zhang X, and Giovannucci EL. Vitamin D and human health: evidence from Mendelian randomization studies. Eur J Epidemiol. 2024;39(5):467-90. Epub 2024/01/12. doi: 10.1007/s10654-023-01075-4IF: 7.7 Q1 . PubMed PMID: 38214845IF: 7.7 Q1 , Sun et al. Association between vitamin D level and risk of type 2 diabetes: a systematic review of Mendelian Randomization studies. Crit Rev Food Sci Nutr. 2025 Mar 12:1-10. doi: 10.1080/10408398.2025.2466758IF: 7.3 Q1 , J Clin Endocrinol Metab. 2024 Jul 12;109(8):1907-1947. doi: 10.1210/clinem/dgae290IF: 5.0 Q1 . and The Journal of Clinical Endocrinology & Metabolism, Volume 109, Issue 8, August 2024, Pages 1948–1954, https://doi.org/10.1210/clinem/dgae322IF: 5.0 Q1 .<br>Response: we have checked the expression of our result and conclusion to avoid expressing causal relationships. Our study is only a cross-sectional epidemiological research.<br><br>5. The main source of vitamin D in human beings. I agree with Reviewer 5 that food intake may be the greatest source of vitamin D in White people living in the Transatlantic region where the sunny period is relatively limited. However, textbooks report vitamin D production by the skin as the main source of vitamin D in human beings, with food intake contributing to only up to 10% of the human body's store of vitamin D. The limited epidemiologic data on the source of vitamin D in the other ancestral groups in the transatlantic region and in tropical regions (for example, see the articles by Vearing, R.M., Hart, K.H., Darling, A.L. et al. Global Perspective of the Vitamin D Status of African-Caribbean Populations: A Systematic Review and Meta-analysis. Eur J Clin Nutr 76, 516–526 (2022). https://doi.org/10.1038/s41430-021-00980-9IF: 3.6 Q2 and Lancet Glob Health. 2020 Jan;8(1):e134-e142. doi: 10.1016/S2214-109X(19)30457-7IF: 19.9 Q1 . Epub 2019 Nov 27) does not allow the verification of this textbook knowledge in these other ancestral groups. However, vitamin D production by the skin could be the predominant source of vitamin D in the non-White population (at least those with a melanoderm skin), given their much higher exposure to sun-derived UVB radiations throughout the year with co-existent undernutrition when compared with White populations.I have also appended additional comments in the attached pdf.<br>Response: sun exposure and food intake are main sources of vitamin D. We totally agree this comment from reviewer and editor. The discussion has been added in the section of limitation. We have carefully read the additional comments added from editor. thank you so much for helping us improving our manuscript.<br><br><br>Reviewers' comments:<br>Reviewer #1: All revisions were made and there is no further comment. The revised manuscript meet the journal publish standard.<br>Response: Thank you so much.<br><br>Reviewer #2: There is no comment. In my opinion, the present version of the manuscript is ready for publication. However, please check the plagiarism of the whole text and be sure that the similarity index was less than 30 %.<br>Response: Thank you so much. We have checked the plagiarism of manuscript and it meets the journal standards.<br><br><br>Reviewer #3: Authors addressed most of the previous comments from reviewers. The manuscript reads better in terms of flow and clarity. However further language editing is still required in many sections throughout the manuscript.<br><br>1. In the abstract, authors to reflect the U shape effect observed among the males; improve clarity of sentences<br>Response: we are sorry for this language problem. In the revised abstract, we clearly state the U-shaped effect, provides context for its significance, and ensures each sentence is concise and informative.<br><br>2. In methodology, citing previous publications, authors to briefly elaborate on each

Powered by Editorial Manager® and ProduXion Manager® from Aries Systems Corporation

	biomarkers/model. At present, there's gap of information for these modelling.<br>Response: We thank the reviewer for your constructive comments. To fully elucidate the rationale for selecting analysis models, we have supplemented the following sentences in the Methods section: “To explore the non-linear relationship between serum 25(OH)D and physiological aging, generalized additive model (GAM) was constructed. The GAMs extend traditional linear regression by replacing linear terms with smooth functions, allowing flexible modeling of complex dose-response relationships without assuming a priori parametric forms. The threshold effect was further estimated using segmented regression model. This method identifies inflection points where the slope of the association between 25(OH)D and PhenoAgeAccel changes significantly. The optimal breakpoint was determined via likelihood ratio tests, comparing the goodness-of-fit between the non-segmented linear model and the segmented regression model.” (Page 7, line 180-189).<br>The following references were supplemented in the References List:<br>28 Hastie TJ. Generalized additive models. Statistical models in S. 2017:249-307.<br>29 Muggeo VM. Estimating regression models with unknown break-points. Stat Med. 2003;22(19):3055-71. Epub 2003/09/16. doi: 10.1002/sim.1545. PubMed PMID: 12973787.<br><br>3. Is there methodology established to determine physiological aging? if there is, how does the PhenoAge algorithm fare when compared? This would further establish validity of methodology.<br>Response: We thank the reviewer for your constructive comments. In agree with the editor’s suggestion, the terminology has been revised from "physiological aging" to "biological aging" throughout the manuscript, as this better aligns with the definition of PhenoAge—a composite metric integrating 10 clinical biomarkers that collectively reflect systemic inflammatory, metabolic, and hematopoietic dysfunction. There are several established methods for quantification of biological aging, including molecular clocks (e.g., DNA methylation clocks), functional decline indices (e.g., frailty index) and composite biomarker metrics (e.g., PhenoAge). The PhenoAge algorithm was constructed based on NHANES III (1988–1994), and was validated using data form NHANES IV (1999–2010). Hence, in NHANES dataset, the PhenoAge method showed good data compatibility and epidemiological validity. In the Methods section, we have supplemented the following sentences to elucidate the rationale for choosing PhenoAge as biological aging indicator: “In this study, we utilized PhenoAge as the indicator of biological aging, considering its high robustness and feasibility in NHANES data as a reliable predictor of mortality and age-related disease risks. The PhenoAge algorithm is constructed using NHANES III (1988–1994) by elastic-net regression, encompasses 10 markers: albumin, creatinine, glucose, white blood cell count, lymphocyte percent, red cell distribution width, mean red cell volume, C-reactive protein, alkaline phosphatase and chronological age [21]. PhenoAge was both developed and validated within NHANES populations. Meanwhile, all constituent biomarkers are routinely measured in NHANES with standardized protocols, ensuring reproducibility across study cycles.” (Page 5, line 118-140).<br><br>4. In discussion and results, there's inconsistency in reporting ie ie dose-dependent relationship in females<br>Response: we have improved this description in the section of discussion and results. Thank you for your suggestion.<br><br>5. In discussion, further strengthening of write up required. At present, it is unclear if low vit d level drives/associated with physiological ageing or is an impact of ageing process.<br>Response: Numerous evidences have found that vitamin D may play a role in modulating key biological pathways associated with chronological age. Vitamin D is known to influence cellular senescence, oxidative stress, and inflammation, which are central to the aging process. Furthermore, experimental studies have demonstrated that vitamin D supplementation can mitigate oxidative damage and improve immune function in elder, suggesting its potential role in slowing age-related physiological decline. Thus, we did not provide enough information about this aspect. In the revised manuscript, we have added this in the discussion. Moreover, we have asked professional help to improve our language. Thank you so much for your review.

Powered by Editorial Manager® and ProduXion Manager® from Aries Systems Corporation

	Reviewer #4: Feng et al. investigated the association between vitamin D deficiency and physiological aging. While the authors have made considerable efforts to address previous reviewers' comments, I believe the manuscript still requires further revision. Below are my comments:<br>1. The Introduction remains somewhat lengthy. Additionally, please clarify that endogenous vitamin D production in humans and animals yields vitamin D3, not D2 (see line 43).<br>Response: thank you for your suggestion. The revised introduction is more streamlined, removing unnecessary details while maintaining essential information. Vitamin D exists in two primary forms: vitamin D3 (cholecalciferol) and vitamin D2 (ergocalciferol). D3 is produced from 7-dehydrocholesterol (7-DHC) through a two-step process in which the B ring is broken by UV light (spectrum 280–320 UVB) radiation from the sun, forming pre-D3 that isomerizes to D3 in a thermo-sensitive but noncatalytic process. Both UVB intensity and skin pigmentation level contribute to the rate of D3 formation. Vitamin D3 is synthesized endogenously in the skin of humans and animals upon sunlight exposure, while vitamin D2 is primarily derived from dietary sources.<br><br>2. Appropriate references should be added for the activation steps of vitamin D, including both the 25-hydroxylation and 1α-hydroxylation processes.<br>Response: we have checked the reference and added appropriate one in the section of introduction.<br><br>3. Line 47: Please correct the terminology to "1α,25-dihydroxyvitamin D."<br>Response: Sorry for this mistake and we have corrected it. Thank you so much.<br><br>4. Line 49: Choose either reference 1 or 2, citing both is unnecessary.<br>Response: In the revised manuscript, reference 2 was removed.<br><br>5. The criteria for defining vitamin D status are based solely on the Endocrine Society guidelines. The Institute of Medicine (IOM) guidelines, which differ substantially and are widely used in clinical settings, should also be mentioned. See ref below:<br>A. Catharine Ross, JoAnn E. Manson, Steven A. Abrams, John F. Aloia, Patsy M. Brannon, Steven K. Clinton, Ramon A. Durazo-Arvizu, J. Christopher Gallagher, Richard L. Gallo, Glenville Jones, Christopher S. Kovacs, Susan T. Mayne, Clifford J. Rosen, Sue A. Shapses, The 2011 Report on Dietary Reference Intakes for Calcium and Vitamin D from the Institute of Medicine: What Clinicians Need to Know, The Journal of Clinical Endocrinology & Metabolism, Volume 96, Issue 1, 1 January 2011, Pages 53–58, https://doi.org/10.1210/jc.2010-2704IF: 5.0 Q1<br>Response: We thank the reviewer for your insightful comment. The Institute of Medicine (IOM) guidelines define serum 25(OH)D thresholds based on musculoskeletal health outcomes, including calcium absorption efficiency, fracture risk, and prevention of rickets/osteomalacia [1]. Their criteria, widely adopted in clinical practice for U.S. and Canadian populations, align with the NHANES data characteristics. The IOM classification are as follows: risk of deficiency (<30 nmol/L), risk of inadequacy (30–50 nmol/L), likelihood of sufficiency (50–75 nmol/L) and no increased benefit (≥75 nmol/L). These thresholds are evidence-based and regionally validated, making them appropriate for our exploratory analysis.<br>While the 2011 Endocrine Society guidelines proposed higher thresholds (sufficiency: ≥75 nmol/L and insufficiency: 50-75 nmol/L), their 2024 update withdrew these definitions due to insufficient clinical trial evidence linking specific 25(OH)D levels to net health benefits [2]. Hence, for operational clarity in cross-sectional analysis, we prioritized the IOM criteria. In the Methods, we revised the classification as follows: "According to the Institute of Medicine Committee recommendation, we transformed serum 25(OH)D concentration into categorical variables: risk of deficiency (<30 nmol/L), risk of inadequacy (30–50 nmol/L), likelihood of sufficiency (50–75 nmol/L) and no increased benefit (≥75 nmol/L) [20,21]." (Page 5-6, line 112-115).<br>The following reference was supplemented in the References List:<br>In: Ross AC, Taylor CL, Yaktine AL, Del Valle HB, editors. Dietary Reference Intakes for Calcium and Vitamin D. The National Academies Collection: Reports funded by National Institutes of Health. Washington (DC)2011.<br>Ross AC, Manson JE, Abrams SA, Aloia JF, Brannon PM, Clinton SK, et al. The 2011 report on dietary reference intakes for calcium and vitamin D from the Institute of Medicine: what clinicians need to know. J Clin Endocrinol Metab. 2011;96(1):53-8.

Epub 2010/12/02. doi: 10.1210/jc.2010-2704. PubMed PMID: 21118827; PubMed Central PMCID: PMCPMC3046611.

6. The rationale for the study remains unclear. The authors should clarify their aims more.
Response: we have rewritten the introduction and improved clarification of our aims and background introduction (Page 4, line 82-85). We hope the improvement could make them clear and rational. Thank you very much.

7. The sentence: "However, the comprehensive picture of the association between vitamin D levels and physiological aging remains to be fully elucidated, particularly in the general population without vitamin D deficiency or insufficiency" is vague. Please revise to clarify what is meant by "general population without deficiency or insufficiency."
Response: we want to investigate the relationship between vitamin D and biological aging in the general population, whether they have sufficient or insufficient vitamin D, as most of previous studies focus on the population with insufficient vitamin D, which may contribute to harmful effects on health. The former sentence has been rewritten as "However, the comprehensive picture of the association between vitamin D levels and biological aging remains to be fully elucidated, particularly in the general population with vitamin D levels within the normal range."

8. Although several covariates were adjusted for, the possibility of residual confounding, particularly from factors such as sun exposure and vitamin D supplementation, should be acknowledged in the discussion.
Response: we totally agree with your opinion. sun exposure and vitamin D supplementation greatly affect vitamin D level in body. We have added the information in the section of discussion and limitation. (Page 14, line371-381)

9. Recent research has provided new insights into vitamin D metabolism and its regulation by metabolic state:
Aatsinki and Elkhwanky et al. (2019) demonstrated that fasting and diabetes activate at least two transcriptional regulators i.e., PGC-1α/ERRα and the glucocorticoid receptor (GR), which repress CYP2R1 and thereby reduce bioactivation of vitamin D. These findings question the long-held assumption that vitamin D levels solely reflect intake and sun exposure.
Elkhwanky et al. (2020) showed that obesity suppresses CYP2R1, the enzyme responsible for vitamin D 25-hydroxylation, in both hepatic and extrahepatic tissues.

These two references below need to be cited and discussed in the discussion section:
Aatsinki SM*, Elkhwanky MS*, Kummu O, Karpale M, Buler M, Viitala P, Rinne V, Mutikainen M, Tavi P, Franko A, Wiesner RJ, Chambers KT, Finck BN & Hakkola J (2019) Fasting-Induced Transcription Factors Repress Vitamin D Bioactivation, a Mechanism for Vitamin D Deficiency in Diabetes. Diabetes 68(5): 918-931. https://doi.org/10.2337/db18-1050IF: 6.2 Q1 .
Elkhwanky MS, Kummu O, Piltonen TT, Laru J, Morin-Papunen L, Mutikainen M, Tavi P & Hakkola J (2020) Obesity Represses CYP2R1, the Vitamin D 25-Hydroxylase, in the Liver and Extrahepatic Tissues. JBMR PLUS https://doi.org/10.1002/jbm4.10397IF: 3.4 Q2 .
These findings collectively challenge the traditional view that 25-hydroxylation is a stable, unregulated process, emphasizing instead the influence of metabolic state on vitamin D status. They also raise important questions about the suitability of 25(OH)D as a reliable biomarker in populations with metabolic disorders including diabetes. While 25(OH)D remains the most accepted marker of vitamin D status, these studies should be cited and discussed, particularly in relation to the study's diabetic subgroup
Response: Thank you very much for your insightful suggestion. This is an important issue and we totally agree that 25(OH)D may be not suitable for any conditions as a biomarker of vitamin D, though it is widely used. We have added this part in the limitation of this study (Page 14, line371-381). "Moreover, recent evidence challenge the traditional view that serum 25(OH)D is treated as a reliable biomarker of vitamin D, though it remains the most accepted marker of vitamin D status. For example, in individuals diagnosed with diabetes or obesity, the expression of CYP2R1, the enzyme responsible for vitamin D 25-hydroxylation, are suppressed, resulting in the decrease

Powered by Editorial Manager® and ProduXion Manager® from Aries Systems Corporation

of 25(OH)D while vitamin D level is stable.”

10. Line 261: Please remove either “diseases” or “disorders” to avoid redundancy.
Response: we have removed the “disorders” to avoid redundancy.

11. Lines 313–316: A reference on vitamin D toxicity should be included to support the statement.
Response: a reference on vitamin D toxicity has been added at this paragraph.
43.Marcinowska-Suchowierska E, Kupisz-Urbanska M, Lukaszkiewicz J, Pludowski P, Jones G. Vitamin D Toxicity-A Clinical Perspective. Front Endocrinol (Lausanne). 2018;9:550. Epub 2018/10/09. doi: 10.3389/fendo.2018.00550. PubMed PMID: 30294301; PubMed Central PMCID: PMCPMC6158375.

12. Some phrases still imply causality (e.g., "vitamin D deficiency promotes aging"). Since this is a cross-sectional study, causal language must be avoided.
Response: we are sorry for this mistake, and correct it.

13. The suggestion that high vitamin D levels may accelerate aging in men appears speculative and requires either stronger supporting evidence or a more cautious tone.
Response: we totally agree with your opinion. Our study could not conclude that high vitamin D levels may accelerate aging in men, and just found an interesting relationship between high vitamin D levels and biological aging. Further animal and clinical researches should be carried out to clarify this conclusion.

14. English language revision is recommended.
Response: we have done our best to solve this academic writing problem in the first revision. To further improve our writing, we have asked professional help. Thank you very much for your valuable review.

In summary, although this is a timely and relevant study with notable improvements, several key areas including the clarity of the rationale, the discussion of mechanistic insights, and the interpretation of results, still require revision. Therefore, I recommend a major revision.

Reviewer #5: This is a study on chronological and biological aging and 25(OH-D-vitamin based on data from NHANES.

Major issues:

1. The first problem is how the authors treat age. Chronological age is simple, calculated biological age is also relevant. Phenoage is not used as defined by omitting some of the variables defining biological age.
Response: We appreciate the reviewer’s insightful opinion. The algorithm we used to calculate PhenoAge omitted CRP values. For the current analysis, we used 5 cycles to achieve adequate sample size, 3 of which were absent of CRP data. We conducted additional correlation analyses between 2 PhenoAge estimates with and without CRP values. We found a high correlation of 0.986, suggesting that the exclusion of CRP did not significantly alter the overall estimates of PhenoAge. Additionally, this practice of omitting CRP was referenced form previous researches using the same NHANES datasets [3, 4].
We supplemented the following sentences in the Methods: “In this study, we utilized PhenoAge as the indicator of biological aging, considering its high robustness and feasibility in NHANES data as a reliable predictor of mortality and age-related disease risks. The PhenoAge algorithm is constructed using NHANES III (1988–1994) by elastic-net regression, encompasses 10 markers: albumin, creatinine, glucose, white blood cell count, lymphocyte percent, red cell distribution width, mean red cell volume, C-reactive protein, alkaline phosphatase and chronological age [22]. The R package BioAge was used to compute PhenoAge [16]. PhenoAge was both developed and validated within NHANES populations. Meanwhile, all constituent biomarkers are routinely measured in NHANES with standardized protocols, ensuring reproducibility across study cycles. One of the markers, CRP, was unavailable from 2011 to 2018. Previous studies have applied an algorithm that excluded CRP to calculate PhenoAge, demonstrating good applicability [23, 24]. To assess the validity of using PhenoAge without CRP data, we calculated 2 PhenoAge estimates with and without C-reactive

protein, using data from cycles 2007-2008 and 2009-2010, which contained all 10 markers. We found a strong correlation between 2 PhenoAge estimates with and without CRP (correlation coefficient of 0.986). Based on these findings, we proceeded with the main analysis using the PhenoAge algorithm excluding CRP, employing data from five NHANES cycles.”. (Page 5-6, line 118-140).
Nevertheless, we acknowledge that this is a limitation of the current study and have explicitly acknowledged it in the Discussion: “Secondly, the PhenoAge algorithm we applied did not contain CRP data. This could be a limitation given that CRP is a key marker of inflammation and is potentially closely associated with vitamin D status. The absence of CRP in the PhenoAge calculation might lead to underestimation of the true relationship between vitamin D status and age acceleration. To gain a more accurate understanding of this association, future studies with more comprehensive datasets should be conducted.”. (Page14, line371-381).
The following reference was supplemented in the References List:
23.Liu W, Wang J, Wang M, Hou H, Ding X, Ma L, et al. Oxidative Stress Factors Mediate the Association Between Life's Essential 8 and Accelerated Phenotypic Aging: NHANES 2005-2018. J Gerontol A Biol Sci Med Sci. 2024;79(1). Epub 2023/10/10. doi: 10.1093/gerona/glad240. PubMed PMID: 37813096.
24.He H, Chen X, Ding Y, Chen X, He X. Composite dietary antioxidant index associated with delayed biological aging: a population-based study. Aging (Albany NY). 2024;16(1):15-27. Epub 2024/01/04. doi: 10.18632/aging.205232. PubMed PMID: 38170244; PubMed Central PMCID: PMCPMC10817368.

2. The authors also define a concept called physiological age, which is also referenced in the title. This is a major problem.
Page 16 line “We further calculated physiological aging as the difference between PhenoAge and chronological age.“
This is a confusing terminology, not only for the readers, but the authors also have difficulties with this:
In the abstract line 21 “Biological age showed a negative correlation with serum 25(OH)D levels (β=-0.04; 95% CI: -0.08-0.00, p=0.039)”
In the results page 18 line 211: “physiological aging was found to be negatively associated with serum 25(OH)D levels (β=-0.04; 95% CI: -0.08-0.00).”
Omit the term' physiological age' and name it for what it really is.
Write instead: The difference between Phenoage and age was negatively correlated serum 25(OH)D levels …
Response: We thank the reviewer for your insightful suggestions.
In agree with the editor’s suggestion, the terminology has been revised from "physiological aging" to "biological aging" throughout the manuscript, as this better aligns with the definition of PhenoAge—a composite metric integrating 10 clinical biomarkers that collectively reflect systemic inflammatory, metabolic, and hematopoietic dysfunction. In regression models, the standardized difference between PhenoAge and chronological age was included as the dependent variable. To enhance conciseness, we used the academic term “PhenoAgeAccel” as previously used by other literature [5, 6]. Throughout the Results, we consistently use "PhenoAgeAccel". When discussing accelerated biological aging processes, we used the term "biological aging". These revisions have been made throughout the whole manuscript.
In the Methods section, we have rewritten the following sentences to improve clarity: “We further calculated PhenoAge acceleration (PhenoAgeAccel) as the difference between PhenoAge and chronological age. A positive value of PhenoAgeAccel represents accelerated biological aging process, while a negative value represented slower biological aging process. Subsequently, PhenoAgeAccel was standardized to have a mean of 0 and a standard deviation (SD) of 1.” (Page 6, line 125-140).

Major cohort problem

3. In NHANES, as in the rest of the US, race has been defined in different ways during the years. For example if one of the parents are of different races and one is white, the child is classified according to the race of the other parent. When neither parent is white the child is classified according to the fathers race. Other ways of defining race are in use, such as self-stated, decision by the doctor, or even name analysis. This is a major problem when discussing vitamin D in relation in a mixed cohort like the one used by the author. How big percentage would define race?
As has been thoroughly discussed about the inherent errors caused by race-adjusted

equations for estimating glomerular filtration rate based on creatinine, this also applies to a study on vitamin D.
Response: Thank you for your insightful comment regarding the classification of race in NHANES. Race definition indeed varies based on several criteria, including parental race and self-identification. These different definitions can lead to inconsistencies when analyzing health outcomes, such as vitamin D levels, across diverse populations. Understanding these complexities is essential for accurate interpretation of results and their implications in public health contexts. The lack of data segregation by race is a common study limitation of NHANES surveys in the absence of race-specific cut-off values for vitamin D levels as for other biological biomarkers such as the creatinine-based estimated glomerular filtration rate. Racial stratification has been used in studies of NHANES surveys, this is not the first report on 25(OH)D assessment in a multiracial NHANES population [7]. However, it should be more careful to use this race data, and we have added this in the limitation in the revised manuscript.

4. The main determinant for 25(OH)-D-vitamin in serum/plasma is a genetic variant of vitamin D-binding protein = GC-globulin. There are three isoforms, Gc1, Gc1-2, and Gc2-2 isoforms. The genetic distribution of these isoforms is highly dependent on ethic origin. The three isoforms come with different 25(OH)-D vitamin levels, although this does not affect bioavailable vitamin D not measured in this and many other studies. Thus, there would be a need for different cut-offs for deficiency, depending on the isoforms. For example, vitamin D is associated with colorectal cancer only in one of these isoforms, and the isoforms modify the risk for bladder cancer.
Thus, it can be concluded that the NHANES study is not well suited for this kind of study applying knowledge from the last ten years. Perhaps if using whites only, but it is not biologically accurate to use a composite population like the authors do.
Response: Thank you for your insightful comments regarding the influence of genetic variants of GC-globulin on serum levels of 25(OH)-D and the implications for different ethnic groups. This is an amazing idea applying different cut-offs for vitamin D deficiency depending on the isoforms. We acknowledge that the genetic distribution of these isoforms can significantly impact vitamin D levels and its associated health outcomes. However, we believe that our study's focus on the broader population sampling, including diverse ethnic backgrounds. Current analyses rely on total 25(OH)-D levels for practicality and this is the standard measurement. Several studies have investigated the role of vitamin D in various diseases based on the NHANES data [7-9]. Apparently, it would be better to analyze the isoform levels (Gc1, Gc1-2, Gc2-2) for a more detailed investigation, but NHANES currently lacks this data. With technological advancements and increasing demand, we hope that NHANES could expand and refine its relevant indicators to better accommodate various research needs.

5. The authors state incorrectly that vitamin D is associated with cardiovascular disease and osteoporosis. This has been proven incorrect over the last decade. There remains an association with some inflammatory diseases such as multiple sclerosis and rheumatoid arthritis.
There is no “growing body of evidence” to support a statement of the potential of vitamin D supplementation to promote overall health.
Response: studies found that vitamin D deficiency was associated with cardiovascular disease and osteoporosis. However, several large contemporary trials of vitamin D supplementation have failed to demonstrate significant benefits on cardiovascular disease, casting doubt on the observational evidence, as we described in the section of introduction. We agree with your comment, and rewrite this sentence to make it appropriate. Thank you very much.
As more evidence have been provided, the protective role of vitamin D or vitamin D supplementation has been challenged in some diseases. We need to exercise caution when stating that vitamin D serum concentrations are associated with diseases and that vitamin D supplementation is beneficial in disease prevention [10].
“These evidence highlights the potential of vitamin D supplementation in reducing the risk of several chronic diseases, particularly in elderly people, where serum vitamin D levels tend to decline with age.”

Minor issues:

6. Vitamin D is more common in southern compared to northern Europe (PMID: 34933700IF: 3.0 Q2), as vitamin D is probably more dependent on food intake

compared to sun exposure.
Response: Thank you for your thoughtful comments. We agree with your opinion about the source of Vitamin D. studies report that the main source of vitamin D in human beings is vitamin D production by the skin, while food intake contributing to only up to 10% of the human body's store of vitamin D. however, in White people living in high latitude regions, food intake is the main source of vitamin D, as they are exposed to less sun-derived UVB radiations compared with other non-White population. In the current study, we analyzed the association of serum vitamin D level and biological aging, but did not investigate whether vitamin D is from sun exposure or food intake.

7. The cut-off for deficiency at 50 nmol per liter is not solid, it is a consensus not anchored in facts. A more likely cut-off would be 25 nmol/L.
Response: We appreciate the reviewer’s insightful comment. We fully recognize that vitamin D deficiency definitions remain contentious worldwide. Multiple scientific organizing committees have proposed distinct cutoff values for vitamin D deficiency status. Their suggestions stemmed from evidence in different target population, which means various factors would affect the population vitamin D status, such as UVB exposure, dietary habits, and genetic factors influencing vitamin D metabolism. In the current analysis, we categorize vitamin D level based on the Institute of Medicine (IOM) guidelines [1]. The IOM guidelines specifically addressed U.S. and Canadian populations with considerations for bone health outcomes, directly corresponding to the NHANES cohort's demographic characteristics. Hence, for operational clarity in cross-sectional analysis, we chose to reference the IOM criteria. In the Methods, we have rewritten the following sentences to improve clarity: “According to the Institute of Medicine Committee recommendation, we transformed serum 25(OH)D concentration into categorical variables: risk of deficiency (<30 nmol/L), risk of inadequacy (30–50 nmol/L), likelihood of sufficiency (50–75 nmol/L) and no increased benefit (≥75 nmol/L) [20,21].” (Page 6, line 112-115).
The following reference was supplemented in the References List:
20.In: Ross AC, Taylor CL, Yaktine AL, Del Valle HB, editors. Dietary Reference Intakes for Calcium and Vitamin D. The National Academies Collection: Reports funded by National Institutes of Health. Washington (DC)2011.
21.Ross AC, Manson JE, Abrams SA, Aloia JF, Brannon PM, Clinton SK, et al. The 2011 report on dietary reference intakes for calcium and vitamin D from the Institute of Medicine: what clinicians need to know. J Clin Endocrinol Metab. 2011;96(1):53-8. Epub 2010/12/02. doi: 10.1210/jc.2010-2704. PubMed PMID: 21118827; PubMed Central PMCID: PMCPMC3046611.
Nevertheless, we fully acknowledge that vitamin D deficiency threshold remain an ongoing controversy. We have expanded the Discussion to address this: “Our study identified a transition point in relationship between serum 25(OH)D level and biological aging, with males exhibiting a threshold at 38.20 nmol/L and females at 62.50 nmol/L. Beyond this transition point, the increase in 25(OH)D level no longer associates with biological aging deceleration. Regarding the threshold of vitamin D deficiency, it remains an ongoing controversy. The Scientific Advisory Committee on Nutrition (2016) recommended threshold at 25 nmol/L among UK population [46], whereas the IOM (2011) suggested thresholds at 30 nmol/L based on evidence in U.S. and Canadian populations [20]. Meanwhile, the European Food Safety Authority 2016 and the Endocrine Society guideline 2011 proposed higher level of 50 nmol/L as deficiency cutoff [47,48]. The discrepancy in cutoff values stems from multiple factors, for example, different regional ultraviolet B exposure patterns, dietary practice and population-specific biological variability. Furthermore, guideline variations also reflect different focus on health outcomes, with some prioritizing musculoskeletal health [49]. In 2024, the Endocrine Society guideline retracted their previous recommendation of threshold at 50 nmol/L due to limited clinical trial evidence [7]. It also highlighted the impracticality of universal thresholds due to heterogeneity in health outcomes and population characteristics. Hence, the interpretation of 25(OH)D level threshold determination in our study should be with caution, especially when it derived from observational study in an ethnically diverse population. Nevertheless, the trend of benefits attenuation we observed is worth noting, suggesting excessive level of serum 25(OH)D may not be beneficial regarding biological aging process.” (Page 12, line332-352).
The following reference was supplemented in the References List:
46.England PH. SACN vitamin D and health report 2016 [updated 2025-04-22]. Available from: https://www.gov.uk/government/publications/sacn-vitamin-d-and-

health-report.
47.Turck D, Bresson J, Burlingame B. Scientific opinion on dietary reference values for vitamin D EFSA Panel on Dietetic Products, Nutrition, and Allergies (NDA). EFSA J. 2016;179.
48.Holick MF, Binkley NC, Bischoff-Ferrari HA, Gordon CM, Hanley DA, Heaney RP, et al. Evaluation, treatment, and prevention of vitamin D deficiency: an Endocrine Society clinical practice guideline. J Clin Endocrinol Metab. 2011;96(7):1911-30. Epub 2011/06/08. doi: 10.1210/jc.2011-0385. PubMed PMID: 21646368.
49.Roth DE, Abrams SA, Aloia J, Bergeron G, Bourassa MW, Brown KH, et al. Global prevalence and disease burden of vitamin D deficiency: a roadmap for action in low- and middle-income countries. Ann N Y Acad Sci. 2018;1430(1):44-79. Epub 2018/09/19. doi: 10.1111/nyas.13968. PubMed PMID: 30225965; PubMed Central PMCID: PMCPMC7309365.

8. It is, however, interesting that higher levels are less beneficial in terms of biological age. This is not in line with the recommendations from organisations associated with the industry for supplementing vitamin D, aiming to increase vitamin D levels in the population.
Response: Thank you for your comments. Our findings indicate that high levels of vitamin D are not associated with reduced aging; in fact, they may be linked to accelerated aging in males. While this is an intriguing observation, we cannot conclude that high vitamin D levels promote aging based on this cross-sectional study. Currently, we can only suggest a possible association, and further research is needed to clarify these results.

Reviewer #6: In this manuscript by Feng et al, the authors aimed to explore the link between blood vitamin D levels and physiological aging, using data from over 13,000 U.S. adults in NHANES (2007–2016). The physiological aging was estimated using the PhenoAge model. The results revealed a nonlinear relationship: lower vitamin D levels were generally associated with accelerated aging, especially in females, while excessively high levels were linked to faster aging in males. These patterns suggest that both deficiency and excess of vitamin D may impact aging differently by gender. This revised manuscript has addressed most of the concerns raised by the Editor and Reviewer. However, there are a number of points that still need to be clarified. Specific comments are as follows:
1. The authors mentioned in the methods that CRP numbers were missing in some NHANES data and that PhenoAge (excluding CRP) was used. It would be helpful to briefly comment on how omitting CRP might affect comparability with studies using the standard PhenoAge metric.
Response: We thank the reviewer for your constructive suggestion. For the current analysis, we used 5 cycles to achieve adequate sample size, 3 of which were absent of CRP data. We conducted additional correlation analyses between 2 PhenoAge estimates with and without CRP values. We found a high correlation of 0.986, suggesting that the exclusion of CRP did not significantly alter the overall estimates of PhenoAge. Additionally, this practice of omitting CRP was referenced form previous researches using the same NHANES datasets [3, 4].
We supplemented the following sentences in the Methods: “In this study, we utilized PhenoAge as the indicator of biological aging, considering its high robustness and feasibility in NHANES data as a reliable predictor of mortality and age-related disease risks. The PhenoAge algorithm is constructed using NHANES III (1988–1994) by elastic-net regression, encompasses 10 markers: albumin, creatinine, glucose, white blood cell count, lymphocyte percent, red cell distribution width, mean red cell volume, C-reactive protein, alkaline phosphatase and chronological age [22]. The R package BioAge was used to compute PhenoAge [16]. PhenoAge was both developed and validated within NHANES populations. Meanwhile, all constituent biomarkers are routinely measured in NHANES with standardized protocols, ensuring reproducibility across study cycles. One of the markers, CRP, was unavailable from 2011 to 2018. Previous studies have applied an algorithm that excluded CRP to calculate PhenoAge, demonstrating good applicability [23, 24]. To assess the validity of using PhenoAge without CRP data, we calculated 2 PhenoAge estimates with and without C-reactive protein, using data from cycles 2007-2008 and 2009-2010, which contained all 10 markers. We found a strong correlation between 2 PhenoAge estimates with and without CRP (correlation coefficient of 0.986). Based on these findings, we proceeded

Powered by Editorial Manager® and ProduXion Manager® from Aries Systems Corporation

	with the main analysis using the PhenoAge algorithm excluding CRP, employing data from five NHANES cycles.". (Page5-6, line 118-140).<br>Additionally, we acknowledge that this is a limitation of the current study and have explicitly acknowledged it in the Discussion: "Secondly, the PhenoAge algorithm we applied did not contain CRP data. This could be a limitation given that CRP is a key marker of inflammation and is potentially closely associated with vitamin D status. The absence of CRP in the PhenoAge calculation might lead to underestimation of the true relationship between vitamin D status and age acceleration. To gain a more accurate understanding of this association, future studies with more comprehensive datasets should be conducted.". (Page 14, line371-381).<br>The following reference was supplemented in the References List:<br>23.Liu W, Wang J, Wang M, Hou H, Ding X, Ma L, et al. Oxidative Stress Factors Mediate the Association Between Life's Essential 8 and Accelerated Phenotypic Aging: NHANES 2005-2018. J Gerontol A Biol Sci Med Sci. 2024;79(1). Epub 2023/10/10. doi: 10.1093/gerona/glad240. PubMed PMID: 37813096.<br>24.He H, Chen X, Ding Y, Chen X, He X. Composite dietary antioxidant index associated with delayed biological aging: a population-based study. Aging (Albany NY). 2024;16(1):15-27. Epub 2024/01/04. doi: 10.18632/aging.205232. PubMed PMID: 38170244; PubMed Central PMCID: PMCPMC10817368.<br><br>2. In Line 220, the authors cite a p-value (p = 0.076) to describe the absence of dose-dependent trends in both the whole population and females. It is suggested to clarify this sentence to correctly distinguish between the groups with the right numbers.<br>Response: thank you for your careful reading. We have corrected this mistake to make it clear.<br><br>3. In the Discussion section (line 323), the authors state that serum 25(OH)D levels above 125 nmol/L in female participants were linked to accelerated physiological aging. However, this interpretation does not appear to be supported by the data in Table 3. Revising this statement to more accurately reflect the results is recommended.<br>Response: thank you very much for your comments. We have revised as "Our results found the 25(OH)D levels over 91.5 nmol/L in male participants were not linked to accelerated biological aging, approximately consistent with those reports."<br><br>Reference<br>1.. Ross AC, Taylor CL, Yaktine AL, Del Valle HB, editors. Washington (DC)2011.<br>2.McCartney CR, McDonnell ME, Corrigan MD, Lash RW. Vitamin D Insufficiency and Epistemic Humility: An Endocrine Society Guideline Communication. J Clin Endocrinol Metab. 2024;109(8):1948-54. Epub 2024/06/03. doi: 10.1210/clinem/dgae322. PubMed PMID: 38828961.<br>3.He H, Chen X, Ding Y, Chen X, He X. Composite dietary antioxidant index associated with delayed biological aging: a population-based study. Aging (Albany NY). 2024;16(1):15-27. Epub 2024/01/04. doi: 10.18632/aging.205232. PubMed PMID: 38170244; PubMed Central PMCID: PMCPMC10817368.<br>4.Liu W, Wang J, Wang M, Hou H, Ding X, Ma L, et al. Oxidative Stress Factors Mediate the Association Between Life's Essential 8 and Accelerated Phenotypic Aging: NHANES 2005-2018. J Gerontol A Biol Sci Med Sci. 2024;79(1). Epub 2023/10/10. doi: 10.1093/gerona/glad240. PubMed PMID: 37813096.<br>5.Dan YL, Yang YQ, Zhu DC, Bo L, Lei SF. Accelerated biological aging as a potential risk factor for rheumatoid arthritis. Int J Rheum Dis. 2024;27(4):e15156. Epub 2024/04/26. doi: 10.1111/1756-185X.15156. PubMed PMID: 38665050.<br>6.Jain P, Binder A, Chen B, Parada H, Jr., Gallo LC, Alcaraz J, et al. The Association of Epigenetic Age Acceleration and Multimorbidity at Age 90 in the Women's Health Initiative. J Gerontol A Biol Sci Med Sci. 2023;78(12):2274-81. Epub 2022/09/16. doi: 10.1093/gerona/glac190. PubMed PMID: 36107798; PubMed Central PMCID: PMCPMC10692424.<br>7.Daraghmeh AH, Bertoia ML, Al-Qadi MO, Abdulbaki AM, Roberts MB, Eaton CB. Evidence for the vitamin D hypothesis: The NHANES III extended mortality follow-up. Atherosclerosis. 2016;255:96-101. Epub 2016/11/18. doi: 10.1016/j.atherosclerosis.2016.04.007. PubMed PMID: 27855294.<br>8.Ma J, Li K. Negative Association ...
**Additional Information:**	
**Question**	**Response**

**Financial Disclosure**<br><br>Enter a financial disclosure statement that describes the sources of funding for the work included in this submission. Review the submission guidelines for detailed requirements. View published research articles from *PLOS ONE* for specific examples.<br><br>This statement is required for submission and **will appear in the published article** if the submission is accepted. Please make sure it is accurate.<br><br>**Funded studies**<br>Enter a statement with the following details:<br>• Initials of the authors who received each award<br>• Grant numbers awarded to each author<br>• The full name of each funder<br>• URL of each funder website<br>• Did the sponsors or funders play any role in the study design, data collection and analysis, decision to publish, or preparation of the manuscript?<br><br>Did you receive funding for this work?	The author(s) received no specific funding for this work.
**Competing Interests**<br><br>Use the instructions below to enter a competing interest statement for this submission. On behalf of all authors, disclose any competing interests that could be perceived to bias this work—acknowledging all financial support and any other relevant financial or non-financial competing interests.<br><br>This statement is required for submission and **will appear in the published article** if the submission is accepted. Please make sure it is accurate and that any funding sources listed in your Funding Information later in the submission form are also declared in your Financial Disclosure statement.	The authors have declared that no competing interests exist.

View published research articles from *PLOS ONE* for specific examples.<br><br>**NO authors have competing interests**<br><br>Enter: *The authors have declared that no competing interests exist.*<br><br>**Authors with competing interests**<br><br>Enter competing interest details beginning with this statement:<br><br>*I have read the journal's policy and the authors of this manuscript have the following competing interests: [insert competing interests here]*<br><br>* typeset	
**Ethics Statement**<br><br>Enter an ethics statement for this submission. This statement is required if the study involved:<br><br>• Human participants<br>• Human specimens or tissue<br>• Vertebrate animals or cephalopods<br>• Vertebrate embryos or tissues<br>• Field research<br><br>Write "N/A" if the submission does not require an ethics statement.<br><br>General guidance is provided below. Consult the submission guidelines for detailed instructions. **Make sure that all information entered here is included in the Methods section of the manuscript.**	the study involves human participants. all data from NHANES, which is a publicly available dataset managed by the Centers for Disease Control and Prevention. The ethical approval of NHANES has been stated on the website. (https://wwwn.cdc.gov/nchs/nhanes/Default.aspx)

**Format for specific study types**<br><br>**Human Subject Research (involving human participants and/or tissue)**<br>• Give the name of the institutional review board or ethics committee that approved the study<br>• Include the approval number and/or a statement indicating approval of this research<br>• Indicate the form of consent obtained (written/oral) or the reason that consent was not obtained (e.g. the data were analyzed anonymously)<br><br>**Animal Research (involving vertebrate animals, embryos or tissues)**<br>• Provide the name of the Institutional Animal Care and Use Committee (IACUC) or other relevant ethics board that reviewed the study protocol, and indicate whether they approved this research or granted a formal waiver of ethical approval<br>• Include an approval number if one was obtained<br>• If the study involved *non-human primates*, add *additional details* about animal welfare and steps taken to ameliorate suffering<br>• If anesthesia, euthanasia, or any kind of animal sacrifice is part of the study, include briefly which substances and/or methods were applied<br><br>**Field Research**<br><br>Include the following details if this study involves the collection of plant, animal, or other materials from a natural setting:<br>• Field permit number<br>• Name of the institution or relevant body that granted permission	
**Data Availability**<br><br>Authors are required to make all data underlying the findings described fully available, without restriction, and from the time of publication. PLOS allows rare exceptions to address legal and ethical concerns. See the PLOS Data Policy and FAQ for detailed information.	Yes - all data are fully available without restriction

A Data Availability Statement describing where the data can be found is required at submission. Your answers to this question constitute the Data Availability Statement and **will be published in the article**, if accepted.<br><br>**Important:** Stating 'data available on request from the author' is not sufficient. If your data are only available upon request, select 'No' for the first question and explain your exceptional situation in the text box.<br><br>Do the authors confirm that all data underlying the findings described in their manuscript are fully available without restriction?	
**Describe where the data may be found in full sentences. If you are copying our sample text, replace any instances of XXX with the appropriate details.**<br><br>• If the data are **held or will be held in a public repository**, include URLs, accession numbers or DOIs. If this information will only be available after acceptance, indicate this by ticking the box below. For example: *All XXX files are available from the XXX database (accession number(s) XXX, XXX.*).<br>• If the data are all contained **within the manuscript and/or Supporting Information files**, enter the following: *All relevant data are within the manuscript and its Supporting Information files.*<br>• If neither of these applies but you are able to provide **details of access elsewhere**, with or without limitations, please do so. For example:<br><br>*Data cannot be shared publicly because of [XXX]. Data are available from the XXX Institutional Data Access / Ethics Committee (contact via XXX) for researchers who meet the criteria for access to confidential data.*<br><br>*The data underlying the results presented in the study are available from (include the name of the third party*	All files are available from the NHANES database

and contact information or URL).<br>• This text is appropriate if the data are owned by a third party and authors do not have permission to share the data.<br><br>* typeset	
Additional data availability information:	

Dear Editors:

The revised manuscript without any tracked changes or highlighting has been uploaded as manuscript. Thank you so much. If you have any queries, please don't hesitate to contact me. Thank you and best regards.

Yours sincerely

Corresponding author: Jue Wang

May, 2025

Manuscript
Click here to access/download;Manuscript;revised manuscript clean_20250514.docx

Association of 25(OH)D serum level with biological aging: A Cross-Sectional Study of 2007-2016 NHANES surveys

Yi Feng[a]; Licheng Yu[b]; Jue Wang[a] *

[a] Nanhui new town Community Health Service Center, Pudong New Area, Shanghai, 201308, China.

[b] Prevention and Health Care Department, Heqing Community Health Service Center of Pudong New District, Shanghai, 201201, China.

Corresponding author. Jue Wang

E-mail address: wangjue180130@126.com

Abstract

Vitamin D plays a crucial role in various aspects of human body, including aging, but chronological age may not accurately reflect the true biological aging status. Recently, PhenoAge has been developed to estimate an individual's biological age based on different biological and clinical measures. Therefore, we investigated the relationship between 25(OH)D serum levels and biological aging calculated by PhenoAge using data from the 2007-2016 NHANES survey. 25(OH)D serum levels showed a negative association with PhenoAge acceleration (PhenoAgeAccel) (β=-0.04; 95% ~~CI~~: -0.08 to 0.00~~,~~ ~~p=0.039~~). This negative ~~corre~~lation was ~~only~~ ~~found to be~~ dose-dependent ~~for~~ females (β=-0.07; 95% CI: -0.12 to 0.01~~,~~ ~~p=0.007~~), but not ~~for~~ males. Generalized additive models further showed nonlinear associations ~~were revealing~~ a U-shaped relationship observed in males but an L-shaped pattern in females. Specifically, 25(OH)D serum levels were linked to reduced PhenoAgeAccel at levels below 38.20 nmol/L (15.28 ng/mL) in males (β=-0.013; 95% CI: -0.025 to -0.002, p=0.017) and ~~62.50~~ nmol/L (25ng/mL) in females (β=-0.007; 95% CI: -0.01 to -0.004~~,~~ ~~p<0.001~~). However, 25(OH)D serum levels above 125 nmol/L were not associated with PhenoAgeAccel in females (β=-0.001; 95% CI: -0.005-0.002)~~,~~ while in males~~,~~ elevated levels (>91.55 nmol/L) were associated with increased PhenoAgeAccel (β=0.005 SD; 95% CI: 0.002-0.008~~,~~ ~~p<0.001~~). Our findings indicate that vitamin D deficiency ~~shows~~ ~~a~~ potential ~~link to~~ accelerated biological aging, and high levels of vitamin D in males ~~link to~~ accelerated biological ~~aging~~, offering valuable insights into the relationship between vitamin D and biological aging.

Key words: Vitamin D; Serum 25(OH)D; Biological aging; PhenoAge; Nonlinear association

1. Introduction

Vitamin D is a fat-soluble vitamin that plays a crucial role in various aspects of human health, with its effects extending to bone metabolism, immune function, etc. It is acquired through two primary sources: endogenous production (mainly vitamin D3) in the skin upon exposure to ultraviolet B (UVB) radiations, and exogenous intake (vitamin D2 and D3) through dietary sources, such as fatty fish, egg yolks or supplements. Once absorbed in the body, vitamin D is converted into 25-hydroxyvitamin D (25(OH)D) in the liver, then transformed into the biologically active form, 1α,25-dihydroxyvitamin D (1α,25$(OH)_2$D), in the kidneys [1]. This active form binds to the vitamin D receptor (VDR), regulating calcium and phosphate metabolism [2]. Serum 25(OH)D is the most commonly measured form of vitamin D in humans because it is the major circulating form and provides an accurate indicator of the body's overall vitamin D status [3], and it can be influenced by various factors, including geographic location, race and food intake [4].

Vitamin D deficiency is a global public health concern, associated with increased risk of type 2 diabetes (T2D), hypertension, multiple sclerosis [5-7]. The prevalence of vitamin D deficiency is particularly high among the elderly [8], with some studies suggesting that it may accelerate chronological aging and contribute to age-related diseases and inflammatory diseases, such as cardiovascular disease, multiple sclerosis and rheumatoid arthritis [9]. However, several large randomized clinical trials of vitamin D supplementation failed to demonstrate significant benefits on cardiovascular disease, casting doubt on the evidence from observational studies linking vitamin D deficiency with cardiovascular disease [10, 11]. This ambiguity highlights challenges in assessing the association between vitamin D and aging. A major limitation of previous studies is that they relied on chronological age which is quite discrepant with biological age. Indeed, unlike the chronological age which is a precise reflection of an individual's lifetime, the biomarkers that make up the biological age are mainly determined by a combination of genetic, environmental and lifestyle factors [12]. Accordingly, some individuals may age biologically faster or slower than indicated by

their chronological age.

To better characterize physiological aging in epidemiological studies, several aging metrics based on the combination of clinical (e.g., PhenoAge and GrimAge) and biological (DNAmPhenoAge acceleration) markers have been developed [13]. In a post hoc analysis of the European DO-HEALTH randomized clinical trial involving 777 participants with a mean 25(OH)D level of 22.69 ng/mL, DNAm PhenoAge was the only biological age metric shown to be slowed by daily vitamin D (2000 UI) supplementation among the four biological age metrics that were assessed: DNAm PhenoAge, GrimAge, GrimAge2 and DunedinPACE [14]. PhenoAge was developed and validated using machine learning methods on a National Health and Nutrition Examination Survey (NHANES) dataset of 4000 adults aged 20 to 84 years and it showed a mean absolute error of approximately 3.6 years when predicting the chronological age [15]. The calculation of PhenoAge is based on the addition of serum levels of the following nine biomarkers on top of the chronological age. Although vitamin D supplementation was shown to slow the PhenoAge value in older adults with low 25(OH)D serum concentrations, observational studies linking 25(OH)D serum concentrations with PhenoAge are lacking. Therefore, in this study, we assessed whether there is a linear and/or non-linear relationship between 25(OH)D serum concentration and PhenoAge using datasets of 2007-2016 NHANES surveys. By exploring this relationship, the study aims to enhance our understanding of the link between serum vitamin D levels and biological age in human beings for the benefit of public health.

2. Method

2.1 Study design and population

This study was conducted and reported in agreement to STROBE guidelines for reporting observational studies. This study utilized data derived from the 2007-2016 NHANES, surveys conducted in the United States of America by the National Center for Health Statistics of the Centers for Disease Control and Prevention. All data

included in the current study are publicly available on the NHANES website [16]. NHANES includes in-home interview, physical measurement and laboratory tests. This study is based on data from 50558 participants of the 2007-2016 NHANES datasets. We excluded participants with incomplete information and participants aged less than 40 years old. A total of 13,910 participants were finally included in this analysis (Figure 1).

2.2 Measurement of serum 25(OH)D concentrations

Serum vitamin D levels were reflected by 25(OH)D levels. Because the forms of 25(OH)D measured in the 2007-2016 NHANES sample included both 25(OH)D2 and 25(OH)D3 (classic 25(OH)D3 and C3 epimer of 25(OH)D3 [C3-epi-25(OH)D3]), serum 25(OH)D included 25(OH)D2, 25(OH)D3 and C3-epi-25(OH)D3 in this study. During the study period (2007-2016), 25(OH)D levels were measured in the NHANES population using the liquid chromatography-tandem mass spectrometry (LC-MS/MS). According to the Institute of Medicine Committee recommendation, we transformed serum 25(OH)D concentration into categorical variables: risk of deficiency (<30 nmol/L), risk of inadequacy (30–50 nmol/L), likelihood of sufficiency (50–75 nmol/L) and no increased benefit (≥75 nmol/L) [17, 18].

2.3 Measurement of biological aging

In this study, we utilized PhenoAge as the indicator of biological aging, considering its high robustness and feasibility in NHANES data as a reliable predictor of mortality and age-related disease risks. The PhenoAge algorithm is constructed using NHA[illegible]S III (1988–1994) by elastic-net regression, encompasses 10 markers: albumin, creatinine, glucose, white blood cell count, lymphocyte percent, red cell distribution width, mean red cell volume, C-reactive protein, alkaline phosphatase and chronological age [19]. The R package BioAge was used to compute PhenoAge [15]. PhenoAge was both developed and validated within NHANES populations. Meanwhile, all constituent biomarkers are routinely measured in NHANES with standardized protocols, ensuring

reproducibility across study cycles. One of the markers, CRP, was unavailable from 2011 to ~~2018~~. Previous studies have applied an algorithm that excluded CRP to calculate PhenoAge, demonstrating good applicability [20, 21]. To assess the validity of ~~using~~ PhenoAge without CRP data, we calculated 2 PhenoAge estimates with and without C-reactive protein, using data from cycles 2007-2008 and 2009-2010, which contained all 10 markers. We found a strong correlation between 2 PhenoAge estimates with and without CRP (correlation coefficient of 0.986). Based on th~~ese~~ findings, we proceeded with the main ~~analysis~~ using the PhenoAge algorithm excluding CRP~~, employing data from five NHANES cycles~~. We further calculated PhenoAge acceleration (PhenoAgeAccel) as the difference between PhenoAge and chronological age. A positive value of PhenoAgeAccel represents accelerated biological aging process, while a negative value represented slower biological aging process. Subsequently, PhenoAgeAccel was standardized to have a mean of 0 and a standard deviation (SD) of 1.

~~2.4~~ Covariates

NHANES interviews were conducted by trained interviewers~~, where~~ questionnaires were administered to ~~collect~~ information on demographics (chronological age, gender, race, education level, poverty income ratio (PIR) and ~~lifestyle~~ (tobacco smoking ~~and~~ alcohol consumption ~~variable~~, physical activity and comorbidities). Body mass index (BMI) were measured at the Mobile Examination Center.

The ~~active~~ tobacco smoking was classified into three categories: never smokers (previous smoking of a total of less than 100 cigarettes), current smokers (lifetime smoking of over 100 cigarettes) and former smokers (had smoked ⩾100 cigarettes in their lifetime but had quit smoking at the time of the survey). The ~~alcohol~~ consumption ~~variable~~ was classified as never drinkers (~~had~~ drunk <12 drinks in their lifetime), current drinkers (~~had~~ drunk ⩾12 drinks in their lifetime and at least one drink in the past year) and former drinkers (~~had~~ drunk ⩾12 drinks in their lifetime but no drink in

the past year). Metabolic equiva[illegible] task (MET)~~-min per week~~ were calculated to assess physical activity~~, and levels of physical activity~~ were categorized as: inactive (no regular physical activity), insufficient (<500 MET-min activity/week), moderate (500-1,000 MET-min activity/week) and high (≥1,000 MET-min activity/week) [22]. For sleep duration, participants responded to the question: "How much sleep do you usually get at night on weekdays or ~~workdays~~?" ~~and were~~ categorized into 3 groups: <7 h, 7-8 h and ≥8h~~)~~. The definition of hypertension was based on self-reports, the use of antihypertensive drugs or on the record of a systolic blood pressure≥140 mmHg with or without a diastolic blood pressure≥90 mmHg on physical exam. The definition of diabetes mellitus was based on self-reports or on laboratory test results showing fasting blood glucose levels ≥126 mg/dL or HbA1c values ≥6.5%.

~~2.5~~ Statistical analysis

Weighted multivariable linear regression models were constructed to estimate the association between serum 25(OH)D status and PhenoAge. 25(OH)D serum levels were log-transformed due to their skewed distribution. To evaluate whether the association between 25(OH)D serum levels and PhenoAge was not just linear but also dose-dependent ~~potential dose-response patterns~~, 25(OH)D serum level was analyzed as both a continuous (log-transformed) and a categorical variable. In agreement with standards for linear regression analyses involving continuous dependent variables, the association between 25(OH)D serum levels as continuous variables and PhenoAge was estimated based on beta coefficients with their 95% confidence intervals [23]. All models were adjusted for chronological age, gender, race, education level, PIR, BMI, tobacco smoking and alcohol consumption variable, physical activity, sleep duration, hypertension, diabetes and season of measurement.

To explore whether there is also a non-linear relationship between 25(OH)D serum levels and PhenoAge, a Generalized Additive Model (GAM) was employed to fit the smooth curve . The GAMs extend traditional linear regression by replacing linear terms

with smooth functions, allowing flexible modeling of complex dose-response relationships without assuming a priori parametric forms [24]. The threshold effect was further estimated using segmented regression model. This method identifies inflection points where the slope of the association between 25~~(OH)D~~ and PhenoAgeAccel changes significantly. The optimal breakpoint was determined via likelihood ratio tests, comparing the goodness-of-fit between the non-segmented linear model and the segmented regression model [25]. ~~A *P*-value of <0.05 was~~ considered ~~statistical~~ significance. All data analyses were performed using ~~R~~ software version 4.2.1 (R Core Team, Vienna, Austria). R package 'BioAge' was used to calculate PhenoAge [15], while ~~package~~ 'mgcv' was used to conduct GAM analysis [26]. Continuous data were reported as mean ± standard deviation (SD) ~~or~~ median (interquartile ~~range)~~. Categorical data were reported as numbers (percentage).

~~2.6~~ Ethical considerations

This study utilized data from NHANES study, which was conducted in accordance with the principles in the Declaration of Helsinki. The NHANES protocol received prior ethical approval from the National Center for Health Statistics' Review Board. Since our study is a secondary analysis of publicly accessible, de-identified NHANES data sets, additional institutional review board approval was not required [27, 28].

~~3.~~ Results

~~3.1~~ Characteristics of the study population

The current study included a total of 6836 males and 7074 females, with the characteristics of the study population presented in Table 1. There are significant differences between males and females ~~in~~ the distributions of age, race, BMI, and poverty-income ratio (p < 0.001). Additionally, gender-specific differences were noted ~~in~~ behaviors influencing biological aging, such as smoking, drinking, physical activity, and sleep duration~~, were also observed between males and females~~ (p < 0.001). Notably, males had a higher prevalence of ~~diabetes~~ than females. The median 25(OH)D serum

level in females was 66.60 nmol/L compared with 63.80 nmol/L in males ($p<0.001$), and the PhenoAge was lower in females than in males.

3.2 Association between 25(OH)D serum level with biological aging

The associations between 25(OH)D serum levels and PhenoAgeAccel were presented in Table 2. After adjusting for age, race, education level, PIR, BMI, smoking, alcohol consumption, level of physical, sleep duration and presence of hypertension and/or diabetes mellitus. Serum 25(OH)D levels was found to be negatively associated with PhenoAgeAccel (β=-0.04 SD; 95% CI: -0.08 to 0.00). Segregating data by sex, this association was statistically significant only in females ($p=0.007$). To further explore this relationship, the population was categorized into four groups based on quartiles of 25(OH)D serum level. 25(OH)D serum levels were inversely associated with PhenoAgeAccel, with no apparent dose-dependent trend (p for trend =0.173). When segregating data by sex, we found the population was categorized into four groups according to the IMO guideline, which similarly revealed that higher 25(OH)D serum levels were associated with reduced PhenoAgeAccel, especially in females (p for trend =0.014). Additionally, 25(OH)D levels at 30-75 nmol/L were associated with reduced PhenoAgeAccel in males categorized based on IMO guidelines, though these statistical differences were not observed in males based on quartile categories.

Stratified analysis furtherly revealed that no overall association was observed in males; however, in male subpopulations over 60 years of age with hypertension or diabetes, higher 25(OH)D serum levels were associated with reduced PhenoAgeAccel. Additionally, there were statistically significant interactions between 25(OH)D serum levels and age, hypertension, or diabetes (Fig.2A). In females, mediators such as age, BMI, smoking, drinking, physical activity, and hypertension did not alter the inverse association between 25(OH)D serum levels and PhenoAgeAccel, indicating the robustness of this relationship (Fig.2B).

3.3 Association of 25(OH)D serum levels with PhenoAge on GAM

Given the non-significant trend in Table 2 indicating a non-linear correlation, GAM was utilized to investigate the potential linkage between serum 25(OH)D and PhenoAgeAccel. There was a nonlinear relationship between 25(OH)D serum level and PhenoAgeAccel in both males and females ($p<0.001$) (Fig 3 A-B). In men, the relationship was U-shaped with the two inflection points at 15.8 ng/mL (38.2 nmol/L) and 36.6 ng/mL (91.5 nmol/L) and in women, the segmented regression modelling confirmed a L-shaped relationship. Low 25(OH)D serum levels (<38.2 nmol/L) were negatively correlated with PhenoAgeAccel (β=-0.013 SD; 95% CI: -0.025 to -0.002), whereas high levels (>91.55 nmol/L) showed a positive association (β=0.005 SD; 95% CI: 0.002 to 0.008). For females, 25(OH)D serum levels below 62.5 nmol/L were negatively linked to PhenoAgeAccel (β=-0.007 SD; 95% CI: -0.01 to -0.004), with no significant association observed beyond 125 nmol/L (β=-0.001 SD; 95% CI: -0.005 to -0.002). Overall, low (<38.2 nmol/L) or high (>91.55 nmol/L) 25(OH)D serum levels in males were associated with PhenoAgeAccel, while in females, only low levels (<62.5 nmol/L) pose a risk factor for biological aging (Table 3).

4. Discussion

In the current study, the association between 25(OH)D serum level and biological aging was investigated in the 2007-2016 NHANES surveys. The analysis revealed a negative association between biological age with 25(OH)D serum level, whether categorized by quartiles or according to IOM guideline. Additionally, the trends and association can be observed in females but not in males. Non-linear associations were also found between 25(OH)D serum levels and PhenoAge in both male (U-shaped) and female (L-shaped).

Numerous studies have established that 25(OH)D serum level, representing vitamin D status in our body, are associated with various diseases, including all-cause mortality, cardiovascular diseases, and aging etc. Multiple large-scale observational studies have consistently shown a negative correlation between 25(OH)D serum levels and all-cause mortality risk, lower 25(OH)D serum levels are associated with an

increased risk of all-cause mortality, and a dose-response relationship observed [29, 30]. Individuals with lower vitamin D levels (typically below 30 ng/mL) linked to a higher risk of all-cause mortality compared to those with adequate vitamin D status [31]. Regarding aging, studies have reported that a gradual decline in 25(OH)D serum levels with chronological age, and vitamin D supplementation can mitigate oxidative damage and improve immune function in elder people, suggesting its potential role in slowing age-related physiological decline [32]. This decline is attributed to several factors, including decreased sun exposure, reduced capacity of the skin to synthesize vitamin D, and decreased dietary intake of vitamin D, resulting in vitamin D deficiency in older adults [33]. The synthesis of vitamin D from sun exposure vary in different races, but our study lacks this data of sun exposure. Additionally, we also found that low 25(OH)D serum levels were associated with increased biological aging, suggesting vitamin D may also play a crucial role in the regulation of the aging process. Adequate 25(OH)D levels are essential for maintaining calcium absorption and bone health preventing osteoporosis, a common age-related condition, and regulating calcium homeostasis and bone metabolism [34]. Moreover, vitamin D can interact with antioxidant and anti-inflammatory pathways, such as activating Nrf2 (nuclear factor erythroid 2-related factor 2) and SIRT1 (sirtuin 1) signaling cascades, and suppressing the production of pro-inflammatory cytokines, including interleukin-6 (IL-6) and tumor necrosis factor-alpha (TNF-α), through inhibiting nuclear factor-κB (NF-κB). These actions lead to reduction of inflammation and oxidative stress, both of which are known to contribute to the aging process [34, 35]. Therefore, vitamin D and aging have a mutual influence on each other.

Studies report that vitamin D supplementation could be an effective way to reverse or mitigate diseases, including lower blood pressure and improve endothelial function, leading to reduced risk of cardiovascular events, such as heart attacks and strokes, and improved cardiovascular outcomes [36]. A meta-analysis of 75 randomized controlled trials in 2014 revealed that vitamin D supplementation was associated with a 6% reduction in all-cause mortality [37]. Some studies suggest that the impact of vitamin

D supplementation on all-cause mortality may be more pronounced in certain subgroups, such as older adults or individuals with vitamin D deficiency. For example, vitamin D supplementation was associated with a reduced risk of all-cause mortality in trials with a mean baseline 25(OH)D level <30 ng/mL (RR 0.89, 95% CI 0.80-0.99), but not in trials with a mean baseline 25(OH)D level ⩾30 ng/mL (RR 1.04, 95% CI 0.97-1.11) [38]. Thus, these evidence highlights the potential of vitamin D supplementation in reducing the risk of several chronic diseases, particularly in elderly people, where serum vitamin D levels tend to decline with age.

Despite its potential benefits, the use of vitamin D supplements has increased markedly, and concerns about excessive intake have emerged. the Vitamin D and Omega-3 Trial (VITAL) study, a randomized trial involving over 25,000 participants, discovered that vitamin D supplementation did not significantly reduce the risk of all-cause mortality or lower risk of fractures among generally healthy midlife and older adults without vitamin D deficiency or osteoporosis [39]. Furthermore, increased intake of vitamin D supplements by the general population and a growing number of high-dose prescriptions without medical monitoring might result in a greater risk of excessive vitamin D toxicity[40]. Our current results indicated that the correlation of 25(OH)D serum level and biological aging followed a U-shaped curve in males and an L-shaped curve in females, revealing that high serum level of 25(OH)D is not associated with decelerated aging in either males and females, and even associated with accelerated biological aging in males. These detrimental effects may stem from the toxicity of excessive vitamin D. When vitamin D intake is too high, serum levels of its metabolites [25(OH)D and 24,25(OH)$_2$D] rise beyond the binding capacity of vitamin D binding protein, leading to an increase in the free active metabolite, 1,25(OH)$_2$D. This active form reaches the VDR in the nucleus, constantly promoting gene expression that enhances calcium absorption and bone mobilization [41]. Consequently, this can result in hypercalcemia or hyperphosphatemia, which in turn suppresses initial parathyroid hormone (iPTH) level [41, 42]. Such disrupting balance of calcium and hormone may contribute to cardiovascular disease, kidney damage, and skin aging.

Research reported that the working diagnosis of vitamin D toxicity is diagnosed based on total 25(OH)D concentrations of 32~80ng/mL (80~200 nmol/L) detected by immunoassay [43]. Our results found the 25(OH)D levels over 91.5 nmol/L in male participants were not linked to accelerated biological aging, approximately consistent with those reports.

Our study identified a transition point in relationship between 25(OH)D serum level and biological aging, with males exhibiting a threshold at 38.20 nmol/L and females at 62.50 nmol/L. Beyond this transition point, the increase in 25(OH)D level no longer associates with biological aging deceleration. Regarding the threshold of vitamin D deficiency, it remains an ongoing controversy. The Scientific Advisory Committee on Nutrition (2016) recommended threshold at 25 nmol/L among UK population [44], whereas the IOM (2011) suggested thresholds at 30 nmol/L based on evidence in U.S. and Canadian populations [17]. Meanwhile, the European Food Safety Authority 2016 and the Endocrine Society guideline 2011 proposed higher level of 50 nmol/L as deficiency cutoff [45, 46]. The discrepancy in cutoff values stems from multiple factors, for example, different regional ultraviolet B exposure patterns, dietary practice and population-specific biological variability. Furthermore, guideline variations also reflect different focus on health outcomes, with some prioritizing musculoskeletal health [47]. In 2024, the Endocrine Society guideline retracted their previous recommendation of threshold at 50 nmol/L due to limited clinical trial evidence [48]. It also highlighted the impracticality of universal thresholds due to heterogeneity in health outcomes and population characteristics. Hence, the interpretation of 25(OH)D level threshold determination in our study should be with caution, especially when it derived from observational study in an ethnically diverse population. Nevertheless, the trend of benefits attenuation we observed is worth noting, suggesting excessive level of serum 25(OH)D may not be beneficial regarding biological aging process.

Our results reported differences of dose-response curve between in male and in female, which may be attributed to the sex hormones, especially estrogen. As women age, particularly during the menopausal transition and after menopause, their ovaries produce less estrogen, leading to a decline of estrogen levels. This decline contributes

to the thinning and wrinkling of the skin, as well as changes in hair texture and growth patterns [49]. Some studies suggest that vitamin D could enhance the activity of estrogen receptors (ER) via interacting with them, potentially amplifying the anti-aging effects of estrogen in the body [50]. Vitamin D and estrogen also have a synergistic effect on bone metabolism, helping to prevent osteoporosis and fractures, especially in postmenopausal women [51]. Additionally, vitamin D regulate estrogen metabolism, leading to higher circulating estrogen levels and subsequently delay aging [50]. Therefore, high level of serum 25(OH)D may not be associated with biological aging in females, but the mechanisms underlying the adverse effects of extremely high levels (>100 nmol/L) in males remain unclear. Further studies are needed to clarify the complex interplay between these two important hormonal systems.

The current study has several limitations. First, this cross-sectional study only provides information on the association of 25(OH)D serum level and biological aging, making it difficult to establish causal relationships. Secondly, the PhenoAge algorithm we applied did not contain CRP data. This could be a limitation given that CRP is a key marker of inflammation and is potentially closely associated with vitamin D status. The absence of CRP in the PhenoAge calculation might lead to underestimation of the true relationship between vitamin D status and age acceleration. To gain a more accurate understanding of this association, future studies with more comprehensive datasets should be conducted. Moreover, recent evidence challenge the traditional view that serum 25(OH)D is treated as a reliable biomarker of vitamin D, though it remains the most accepted marker of vitamin D status. For example, in individuals diagnosed with diabetes or obesity, the expression of CYP2R1, the enzyme responsible for vitamin D 25-hydroxylation, are suppressed, resulting in the decrease of 25(OH)D while vitamin D level is stable [52, 53]. Finally, the gender-specific differences in ~~results require further detailed~~ analysis to fully understand the underlying mechanisms.

~~5.~~ Conclusion

In this study, we investigated the relationship between 25(OH)D serum levels and biological age in NHANES, and found that there was a negative association between biological age and 25(OH)D serum level, and these associations were nonlinear in both genders, revealing a U-shaped curve for males and an L-shaped curve for females. Low 25(OH)D serum levels shows a potential link to accelerated biological aging in both genders, but high 25(OH)D serum levels do not correlate with decelerated biological aging, and even associated with accelerated biological aging in male. Our results suggest that the association between vitamin D levels with biological aging is different in men and women.

Authors contributions

Yi Feng: Conceptualization; Methodology; Data curation; Formal analysis; Writing original draft**; Licheng Yu**: Data curation; Formal analysis; **Jue Wang**: Project administration; Supervision; Writing - review &editing.

Conflicts of interest

The authors declare no competing financial or non-financial interests related to this work. NHANES is a publicly available dataset managed by the Centers for Disease Control and Prevention. The authors confirm that there is no affiliation with or involvement from the NHANES program administrators in this study. The funding source had no role in study design, data collection, analysis, or manuscript preparation.

References

1. Bikle DD. Vitamin D metabolism, mechanism of action, and clinical applications. Chem Biol. 2014;21(3):319-29. Epub 2014/02/18. doi: 10.1016/j.chembiol.2013.12.016. PubMed PMID: 24529992; PubMed Central PMCID: PMCPMC3968073.

2. Dominguez LJ, Farruggia M, Veronese N, Barbagallo M. Vitamin D Sources, Metabolism, and Deficiency: Available Compounds and Guidelines for Its Treatment. Metabolites. 2021;11(4). Epub 2021/05/01. doi: 10.3390/metabo11040255. PubMed PMID: 33924215; PubMed Central PMCID:

PMCPMC8074587.
3. Couchman L, Moniz CF. Analytical considerations for the biochemical assessment of vitamin D status. Ther Adv Musculoskelet Dis. 2017;9(4):97-104. Epub 2017/04/07. doi: 10.1177/1759720X17692500. PubMed PMID: 28382113; PubMed Central PMCID: PMCPMC5367642.
4. Rahman A, Elmi A. Air pollutants are negatively associated with vitamin D-synthesizing UVB radiation intensity on the ground. Sci Rep. 2021;11(1):21480. Epub 2021/11/04. doi: 10.1038/s41598-021-00980-6. PubMed PMID: 34728744; PubMed Central PMCID: PMCPMC8563978.
5. Zhao S, Qian F, Wan Z, Chen X, Pan A, Liu G. Vitamin D and major chronic diseases. Trends Endocrinol Metab. 2024. Epub 2024/06/02. doi: 10.1016/j.tem.2024.04.018. PubMed PMID: 38824035.
6. Gaksch M, Jorde R, Grimnes G, Joakimsen R, Schirmer H, Wilsgaard T, et al. Vitamin D and mortality: Individual participant data meta-analysis of standardized 25-hydroxyvitamin D in 26916 individuals from a European consortium. Plos One. 2017;12(2):e0170791. Epub 2017/02/17. doi: 10.1371/journal.pone.0170791. PubMed PMID: 28207791; PubMed Central PMCID: PMCPMC5312926.
7. Fang A, Zhao Y, Yang P, Zhang X, Giovannucci EL. Vitamin D and human health: evidence from Mendelian randomization studies. Eur J Epidemiol. 2024;39(5):467-90. Epub 2024/01/12. doi: 10.1007/s10654-023-01075-4. PubMed PMID: 38214845.
8. Cui A, Xiao P, Ma Y, Fan Z, Zhou F, Zheng J, et al. Prevalence, trend, and predictor analyses of vitamin D deficiency in the US population, 2001-2018. Front Nutr. 2022;9:965376. Epub 2022/10/21. doi: 10.3389/fnut.2022.965376. PubMed PMID: 36263304; PubMed Central PMCID: PMCPMC9573946.
9. Berridge MJ. Vitamin D deficiency accelerates ageing and age-related diseases: a novel hypothesis. The Journal of physiology. 2017;595(22):6825-36. Epub 2017/09/28. doi: 10.1113/JP274887. PubMed PMID: 28949008; PubMed Central PMCID: PMCPMC5685827.
10. Zittermann A, Ernst JB, Prokop S, Fuchs U, Dreier J, Kuhn J, et al. Effect of vitamin D on all-cause mortality in heart failure (EVITA): a 3-year randomized clinical trial with 4000 IU vitamin D daily. Eur Heart J. 2017;38(29):2279-86. Epub 2017/05/13. doi: 10.1093/eurheartj/ehx235. PubMed PMID: 28498942.
11. Scragg R, Stewart AW, Waayer D, Lawes CMM, Toop L, Sluyter J, et al. Effect of Monthly High-Dose Vitamin D Supplementation on Cardiovascular Disease in the Vitamin D Assessment Study : A Randomized Clinical Trial. JAMA Cardiol. 2017;2(6):608-16. Epub 2017/04/07. doi: 10.1001/jamacardio.2017.0175. PubMed PMID: 28384800; PubMed Central PMCID: PMCPMC5815022 ICMJE Form of Disclosure of Potential Conflicts of Interest and none were reported.
12. Xia X, Wang Y, Yu Z, Chen J, Han JJ. Assessing the rate of aging to monitor aging itself. Ageing Res Rev. 2021;69:101350. Epub 2021/05/04. doi: 10.1016/j.arr.2021.101350. PubMed PMID: 33940202.
13. Jylhava J, Pedersen NL, Hagg S. Biological Age Predictors. EBioMedicine. 2017;21:29-36. Epub 2017/04/12. doi: 10.1016/j.ebiom.2017.03.046. PubMed PMID: 28396265; PubMed Central PMCID: PMCPMC5514388.
14. Bischoff-Ferrari HA, Gangler S, Wieczorek M, Belsky DW, Ryan J, Kressig RW, et al. Individual and additive effects of vitamin D, omega-3 and exercise on DNA methylation clocks of biological aging in older adults from the DO-HEALTH trial. Nat Aging. 2025;5(3):376-85. Epub 2025/02/04. doi: 10.1038/s43587-024-00793-y. PubMed PMID: 39900648.
15. Kwon D, Belsky DW. A toolkit for quantification of biological age from blood chemistry and organ function test data: BioAge. Geroscience. 2021;43(6):2795-808. Epub 2021/11/03. doi: 10.1007/s11357-

021-00480-5. PubMed PMID: 34725754; PubMed Central PMCID: PMCPMC8602613.

16. Centers for Disease Control and Prevention National Health and Nutrition Examination Survey Questionnaires D, and Related Documentation 2020. [cited 2025 March 7]. Available from: https://wwwn.cdc.gov/nchs/nhanes/.

17. . Ross AC, Taylor CL, Yaktine AL, Del Valle HB, editors. Washington (DC)2011.

18. Ross AC, Manson JE, Abrams SA, Aloia JF, Brannon PM, Clinton SK, et al. The 2011 report on dietary reference intakes for calcium and vitamin D from the Institute of Medicine: what clinicians need to know. J Clin Endocrinol Metab. 2011;96(1):53-8. Epub 2010/12/02. doi: 10.1210/jc.2010-2704. PubMed PMID: 21118827; PubMed Central PMCID: PMCPMC3046611.

19. Liu Z, Kuo PL, Horvath S, Crimmins E, Ferrucci L, Levine M. A new aging measure captures morbidity and mortality risk across diverse subpopulations from NHANES IV: A cohort study. PLoS Med. 2018;15(12):e1002718. Epub 2019/01/01. doi: 10.1371/journal.pmed.1002718. PubMed PMID: 30596641; PubMed Central PMCID: PMCPMC6312200.

20. Liu W, Wang J, Wang M, Hou H, Ding X, Ma L, et al. Oxidative Stress Factors Mediate the Association Between Life's Essential 8 and Accelerated Phenotypic Aging: NHANES 2005-2018. J Gerontol A Biol Sci Med Sci. 2024;79(1). Epub 2023/10/10. doi: 10.1093/gerona/glad240. PubMed PMID: 37813096.

21. He H, Chen X, Ding Y, Chen X, He X. Composite dietary antioxidant index associated with delayed biological aging: a population-based study. Aging (Albany NY). 2024;16(1):15-27. Epub 2024/01/04. doi: 10.18632/aging.205232. PubMed PMID: 38170244; PubMed Central PMCID: PMCPMC10817368.

22. Haskell WL, Lee IM, Pate RR, Powell KE, Blair SN, Franklin BA, et al. Physical activity and public health: updated recommendation for adults from the American College of Sports Medicine and the American Heart Association. Circulation. 2007;116(9):1081-93. Epub 2007/08/03. doi: 10.1161/CIRCULATIONAHA.107.185649. PubMed PMID: 17671237.

23. Bender R. Introduction to the use of regression models in epidemiology. Cancer epidemiology: Springer; 2009. p. 179-95.

24. Hastie TJ. Generalized additive models. Statistical models in S. 2017:249-307.

25. Muggeo VM. Estimating regression models with unknown break-points. Stat Med. 2003;22(19):3055-71. Epub 2003/09/16. doi: 10.1002/sim.1545. PubMed PMID: 12973787.

26. Wood SN. Stable and efficient multiple smoothing parameter estimation for generalized additive models. Journal of the American Statistical Association. 2004;99(467):673-86.

27. Yucebilgic G, Bilgin R, Tamer L, Tukel S. Effects of lead on Na(+)-K(+) ATPase and Ca(+2) ATPase activities and lipid peroxidation in blood of workers. International journal of toxicology. 2003;22(2):95-7. doi: 10.1080/10915810305096. PubMed PMID: 12745990.

28. World Medical A. World Medical Association Declaration of Helsinki: Ethical Principles for Medical Research Involving Human Participants. JAMA. 2025;333(1):71-4. Epub 2024/10/19 20:42. doi: 10.1001/jama.2024.21972. PubMed PMID: 39425955.

29. Amrein K, Scherkl M, Hoffmann M, Neuwersch-Sommeregger S, Kostenberger M, Tmava Berisha A, et al. Vitamin D deficiency 2.0: an update on the current status worldwide. Eur J Clin Nutr. 2020;74(11):1498-513. Epub 2020/01/22. doi: 10.1038/s41430-020-0558-y. PubMed PMID: 31959942; PubMed Central PMCID: PMCPMC7091696 The other authors declare that they have no conflict of interest.

30. Liu W, Hu J, Fang Y, Wang P, Lu Y, Shen N. Vitamin D status in Mainland of China: A systematic review and meta-analysis. EClinicalMedicine. 2021;38:101017. Epub 2021/07/27. doi:

10.1016/j.eclinm.2021.101017. PubMed PMID: 34308318; PubMed Central PMCID: PMCPMC8283334.
31. Bouillon R, Manousaki D, Rosen C, Trajanoska K, Rivadeneira F, Richards JB. The health effects of vitamin D supplementation: evidence from human studies. Nat Rev Endocrinol. 2022;18(2):96-110. Epub 2021/11/25. doi: 10.1038/s41574-021-00593-z. PubMed PMID: 34815552; PubMed Central PMCID: PMCPMC8609267 Farma, Ceres and Proctor and Gamble. J.B.R. has worked as a consultant to GlaxoSmithKline and Deerfield Capital. The other authors declare no competing interests.
32. Schottker B, Hagen L, Zhang Y, Gao X, Holleczek B, Gao X, et al. Serum 25-Hydroxyvitamin D Levels as an Aging Marker: Strong Associations With Age and All-Cause Mortality Independent From Telomere Length, Epigenetic Age Acceleration, and 8-Isoprostane Levels. J Gerontol A Biol Sci Med Sci. 2019;74(1):121-8. Epub 2018/10/30. doi: 10.1093/gerona/gly253. PubMed PMID: 30371905.
33. Liu L, Chen C, Lo K, Huang J, Yu Y, Huang Y, et al. Serum 25-hydroxyvitamin D, frailty, and mortality among the Chinese oldest old: Results from the CLHLS study. Nutr Metab Cardiovasc Dis. 2021;31(9):2707-15. Epub 2021/08/06. doi: 10.1016/j.numecd.2021.05.033. PubMed PMID: 34348876.
34. Veldurthy V, Wei R, Oz L, Dhawan P, Jeon YH, Christakos S. Vitamin D, calcium homeostasis and aging. Bone Res. 2016;4:16041. Epub 2016/10/30. doi: 10.1038/boneres.2016.41. PubMed PMID: 27790378; PubMed Central PMCID: PMCPMC5068478.
35. Ge D, Chen Q, Xie X, Li Q, Yang Y. Unveiling the potent effect of vitamin D: harnessing Nrf2/HO-1 signaling pathways as molecular targets to alleviate urban particulate matter-induced asthma inflammation. BMC Pulm Med. 2024;24(1):55. Epub 2024/01/26. doi: 10.1186/s12890-024-02869-2. PubMed PMID: 38273268; PubMed Central PMCID: PMCPMC10809564.
36. Pilz S, Gaksch M, Kienreich K, Grubler M, Verheyen N, Fahrleitner-Pammer A, et al. Effects of vitamin D on blood pressure and cardiovascular risk factors: a randomized controlled trial. Hypertension. 2015;65(6):1195-201. Epub 2015/03/25. doi: 10.1161/HYPERTENSIONAHA.115.05319. PubMed PMID: 25801871.
37. Chowdhury R, Kunutsor S, Vitezova A, Oliver-Williams C, Chowdhury S, Kiefte-de-Jong JC, et al. Vitamin D and risk of cause specific death: systematic review and meta-analysis of observational cohort and randomised intervention studies. BMJ. 2014;348:g1903. Epub 2014/04/03. doi: 10.1136/bmj.g1903. PubMed PMID: 24690623; PubMed Central PMCID: PMCPMC3972416 at www.icmje.org/coi_disclosure.pdf (available on request from the corresponding authors) and declare: no support from any organisation for the submitted work; no financial relationships with any organisations that might have an interest in the submitted work in the previous three years; no other relationships or activities that could appear to have influenced the submitted work.
38. Rejnmark L, Bislev LS, Cashman KD, Eiriksdottir G, Gaksch M, Grubler M, et al. Non-skeletal health effects of vitamin D supplementation: A systematic review on findings from meta-analyses summarizing trial data. Plos One. 2017;12(7):e0180512. Epub 2017/07/08. doi: 10.1371/journal.pone.0180512. PubMed PMID: 28686645; PubMed Central PMCID: PMCPMC5501555.
39. LeBoff MS, Chou SH, Ratliff KA, Cook NR, Khurana B, Kim E, et al. Supplemental Vitamin D and Incident Fractures in Midlife and Older Adults. N Engl J Med. 2022;387(4):299-309. Epub 2022/08/09. doi: 10.1056/NEJMoa2202106. PubMed PMID: 35939577; PubMed Central PMCID: PMCPMC9716639.
40. Pludowski P, Grant WB, Karras SN, Zittermann A, Pilz S. Vitamin D Supplementation: A Review of the Evidence Arguing for a Daily Dose of 2000 International Units (50 microg) of Vitamin D for

Adults in the General Population. Nutrients. 2024;16(3). Epub 2024/02/10. doi: 10.3390/nu16030391. PubMed PMID: 38337676; PubMed Central PMCID: PMCPMC10857599.

41. Marcinowska-Suchowierska E, Kupisz-Urbanska M, Lukaszkiewicz J, Pludowski P, Jones G. Vitamin D Toxicity-A Clinical Perspective. Front Endocrinol (Lausanne). 2018;9:550. Epub 2018/10/09. doi: 10.3389/fendo.2018.00550. PubMed PMID: 30294301; PubMed Central PMCID: PMCPMC6158375.

42. Batman A, Saygili ES, Yildiz D, Sen EC, Erol RS, Canat MM, et al. Risk of hypercalcemia in patients with very high serum 25-OH vitamin D levels. Int J Clin Pract. 2021;75(7):e14181. Epub 2021/03/25. doi: 10.1111/ijcp.14181. PubMed PMID: 33759301; PubMed Central PMCID: PMCPMC8250214.

43. Sajid U, Orton D, Kaufmann M, Jones G, Kline GA. Understanding Elevated Vitamin D Measurements to Uncover Hypercalcemia Etiology. Clin Chem. 2024;70(6):798-802. Epub 2024/06/03. doi: 10.1093/clinchem/hvae044. PubMed PMID: 38825340.

44. England PH. SACN vitamin D and health report 2016 [updated 2025-04-22]. Available from: https://www.gov.uk/government/publications/sacn-vitamin-d-and-health-report.

45. Turck D, Bresson J, Burlingame B. Scientific opinion on dietary reference values for vitamin D EFSA Panel on Dietetic Products, Nutrition, and Allergies (NDA). EFSA J. 2016;179.

46. Holick MF, Binkley NC, Bischoff-Ferrari HA, Gordon CM, Hanley DA, Heaney RP, et al. Evaluation, treatment, and prevention of vitamin D deficiency: an Endocrine Society clinical practice guideline. J Clin Endocrinol Metab. 2011;96(7):1911-30. Epub 2011/06/08. doi: 10.1210/jc.2011-0385. PubMed PMID: 21646368.

47. Roth DE, Abrams SA, Aloia J, Bergeron G, Bourassa MW, Brown KH, et al. Global prevalence and disease burden of vitamin D deficiency: a roadmap for action in low- and middle-income countries. Ann N Y Acad Sci. 2018;1430(1):44-79. Epub 2018/09/19. doi: 10.1111/nyas.13968. PubMed PMID: 30225965; PubMed Central PMCID: PMCPMC7309365.

48. Demay MB, Pittas AG, Bikle DD, Diab DL, Kiely ME, Lazaretti-Castro M, et al. Vitamin D for the Prevention of Disease: An Endocrine Society Clinical Practice Guideline. J Clin Endocrinol Metab. 2024. Epub 2024/06/03. doi: 10.1210/clinem/dgae290. PubMed PMID: 38828931.

49. Santoro N, Roeca C, Peters BA, Neal-Perry G. The Menopause Transition: Signs, Symptoms, and Management Options. J Clin Endocrinol Metab. 2021;106(1):1-15. Epub 2020/10/24. doi: 10.1210/clinem/dgaa764. PubMed PMID: 33095879.

50. Crescioli C. The Role of Estrogens and Vitamin D in Cardiomyocyte Protection: A Female Perspective. Biomolecules. 2021;11(12). Epub 2021/12/25. doi: 10.3390/biom11121815. PubMed PMID: 34944459; PubMed Central PMCID: PMCPMC8699224.

51. McCurdy R, Jiang X, Clarkson TB, Nudy M, Schnatz PF. Vitamin D and conjugated equine estrogen: the association with coronary artery atherosclerosis in cynomolgus monkeys. Menopause. 2016;23(5):481-7. Epub 2016/02/18. doi: 10.1097/GME.0000000000000582. PubMed PMID: 26886883.

52. Elkhwanky MS, Kummu O, Piltonen TT, Laru J, Morin-Papunen L, Mutikainen M, et al. Obesity Represses CYP2R1, the Vitamin D 25-Hydroxylase, in the Liver and Extrahepatic Tissues. JBMR Plus. 2020;4(11):e10397. Epub 2020/11/20. doi: 10.1002/jbm4.10397. PubMed PMID: 33210060; PubMed Central PMCID: PMCPMC7657391.

53. Aatsinki SM, Elkhwanky MS, Kummu O, Karpale M, Buler M, Viitala P, et al. Fasting-Induced Transcription Factors Repress Vitamin D Bioactivation, a Mechanism for Vitamin D Deficiency in Diabetes. Diabetes. 2019;68(5):918-31. Epub 2019/03/06. doi: 10.2337/db18-1050. PubMed PMID:

30833469; PubMed Central PMCID: PMCPMC6477896.

Table 1. Characteristics of the study population

Characteristics	Male (n=6836)	Female (n=7074)	*P*-value
Age (mean±SD)	57.07 (11.34)	58.12 (11.82)	<0.001
Race			0.001[b]
Non-Hispanic white	3249 (47.53)	3288 (46.48)	
Non-Hispanic black	1360 (19.89)	1417 (20.03)	
Others	2227 (32.58)	2369 (33.49)	
Education level			0.394[b]
Below high school	1847 (27.02)	1831 (25.88)	
High school or equivalent	1588 (23.23)	1597 (22.58)	
Above high school	3401 (49.75)	3646 (51.54)	
Poverty-income ratio			<0.001[b]
≤1.0	1199 (17.54)	1447 (20.46)	
1.0-3.0	2839 (41.53)	3029 (42.82)	
>3.0	2798 (40.93)	2598 (36.73)	
BMI, kg/m^2			<0.001[b]
<25	1585 (23.19)	1841 (26.02)	
25-30	2749 (40.21)	2117 (29.93)	
≥30	2502 (36.60)	3116 (44.05)	
Smoking			<0.001[b]
Never smoker	2823 (41.30)	4297 (60.74)	
Previous smoker	2570 (37.60)	1666 (23.55)	
Current smoker	1443 (21.11)	1111 (15.71)	
Drinking (alcohol consumption)			<0.001[b]
Never drinker	527 (7.71)	1572 (22.22)	
Previous drinker	637 (9.32)	1466 (20.72)	
Current drinker	5672 (82.97)	4036 (57.05)	
Level of physical activity			<0.001[b]
Inactive	1775 (25.97)	2533 (35.81)	
Insufficient	802 (11.73)	1086 (15.35)	
Moderate	680 (9.95)	842 (11.90)	
High	3579 (52.36)	2613 (36.94)	
Sleep duration			<0.001[b]
<7 h	2572 (37.62)	2445 (34.56)	
7-8h	1858 (27.18)	1812 (25.61)	
≥8 h	2406 (35.20)	2817 (39.82)	
Hypertension			0.839[b]

No	3007 (43.99)	3067 (43.36)	
Yes	3829 (56.01)	4007 (56.64)	
~~Diabetes~~			<0.001[b]
No	5086 (74.40)	5515 (77.96)	
Yes	1750 (25.60)	1559 (22.04)	
Serum 25(OH)D, nmol/L			
Median (IQR)	63.80 (48.38, 80.20)	66.60 (47.00, 87.30)	<0.001[c]
<30	420 (6.14)	545 (7.70)	<0.001[b]
30-50	1451 (21.23)	1457 (20.60)	
50-75	2766 (40.46)	2293 (32.41)	
⩾75	2199 (32.17)	2779 (39.28)	
PhenoAge (median [IQR])	53.77 (45.07, 64.60)	53.05 (44.23, 64.13)	0.032
PhenoAgeAccel (median [IQR])	-2.40 (-5.13, 0.94)	-4.14 (-7.22, -0.46)	<0.001[c]
Standardized PhenoAgeAccel (mean±SD)	0.11 (0.95)	-0.16 (1.05)	<0.001[a]

[a] Student's t-test was used to compare means of variables between two groups

[b] Chi-square test was used to compare categorical variables between two groups.

[c] Mann-Whitney test was used to compare non-normal variables between two groups.

Table 2. Association between 25(OH)D serum level and biological aging

Characteristics	All			Male			Female		
	Beta coefficient (95% CI)		*P*-value	Beta coefficient (95% CI)		*P*-value	Beta coefficient (95% CI)		*P*-value
	Crude model	Adjusted model [a]		Crude model	Adjusted model [a]		Crude model	Adjusted model [a]	
Log(25(OH)D)	-0.27 (-0.32, -0.22)	-0.04 (-0.08, 0.00)	0.039	-0.13 (-0.20, -0.06)	0.03 (-0.02, 0.09)	0.334	-0.32 (-0.38, -0.26)	-0.07 (-0.12, -0.01)	0.007
Quartiles									
Q1	Reference	Reference		Reference	Reference		Reference	Reference	
Q2	-0.23 (-0.29, -0.17)	-0.10 (-0.16, -0.05)	<0.001	-0.16 (-0.24, -0.08)	-0.02 (-0.09, 0.04)	0.448	-0.33 (-0.41, -0.25)	-0.16 (-0.23, -0.08)	<0.001
Q3	-0.29 (-0.36, -0.23)	-0.10 (-0.16, -0.04)	0.001	-0.20 (-0.29, -0.11)	-0.03 (-0.12, 0.04)	0.355	-0.40 (-0.49, -0.32)	-0.15 (-0.22, -0.07)	<0.001
Q4	-0.32 (-0.38, -0.26)	-0.05 (-0.11, -0.00)	0.022	-0.16 (-0.24, -0.07)	0.02 (-0.04, 0.10)	0.553	-0.42 (-0.51, -0.34)	-0.10 (-0.18, -0.02)	0.009
P for trend			0.173			0.429			0.076
Categories based on the Institute of Medicine guideline									
<30	Reference	Reference		Reference	Reference		Reference	Reference	
30-50	-0.23 (-0.33, -0.13)	-0.14 (-0.23, -0.05)	0.002	-0.26 (-0.41, -0.11)	-0.15 (-0.29, -0.02)	0.024	-0.22 (-0.34, -0.09)	-0.10 (-0.21, 0.00)	0.057
50-75	-0.42 (-0.52, -0.33)	-0.21 (-0.30, -0.13)	<0.001	-0.39 (-0.53, -0.25)	-0.16 (-0.29, -0.04)	0.009	-0.51 (-0.63, -0.40)	-0.24 (-0.33, -0.15)	<0.001
⩾75	-0.46 (-0.56, -0.36)	-0.15 (-0.24, -0.06)	0.001	-0.36 (-0.50, -0.22)	-0.09 (-0.22, 0.03)	0.131	-0.54 (-0.66, -0.42)	-0.17 (-0.27, -0.07)	0.001
P for trend			0.130			0.366			0.014

[a] Adjusted for age, race, education level, poverty-income ratio, body mass index, smoking, drinking, physical activity, sleep duration,

hypertension, diabetes and season of measurement.

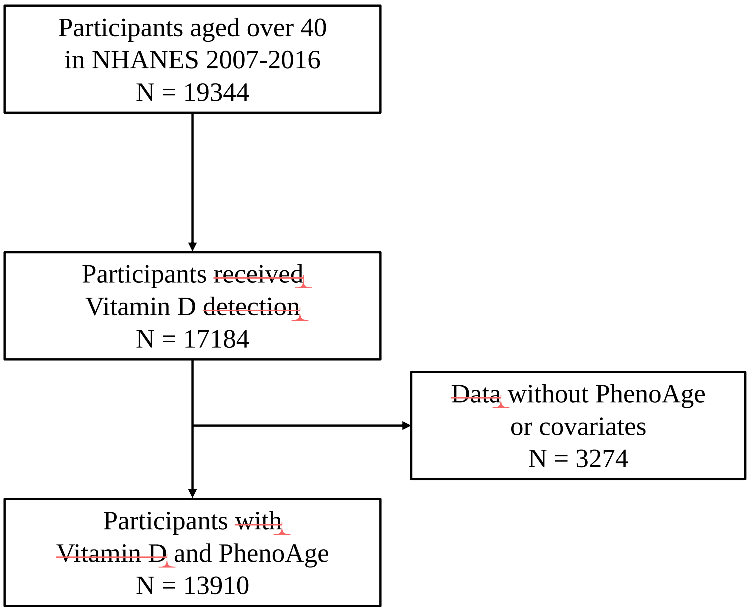

Figure 1 Flowchart of participants selection.

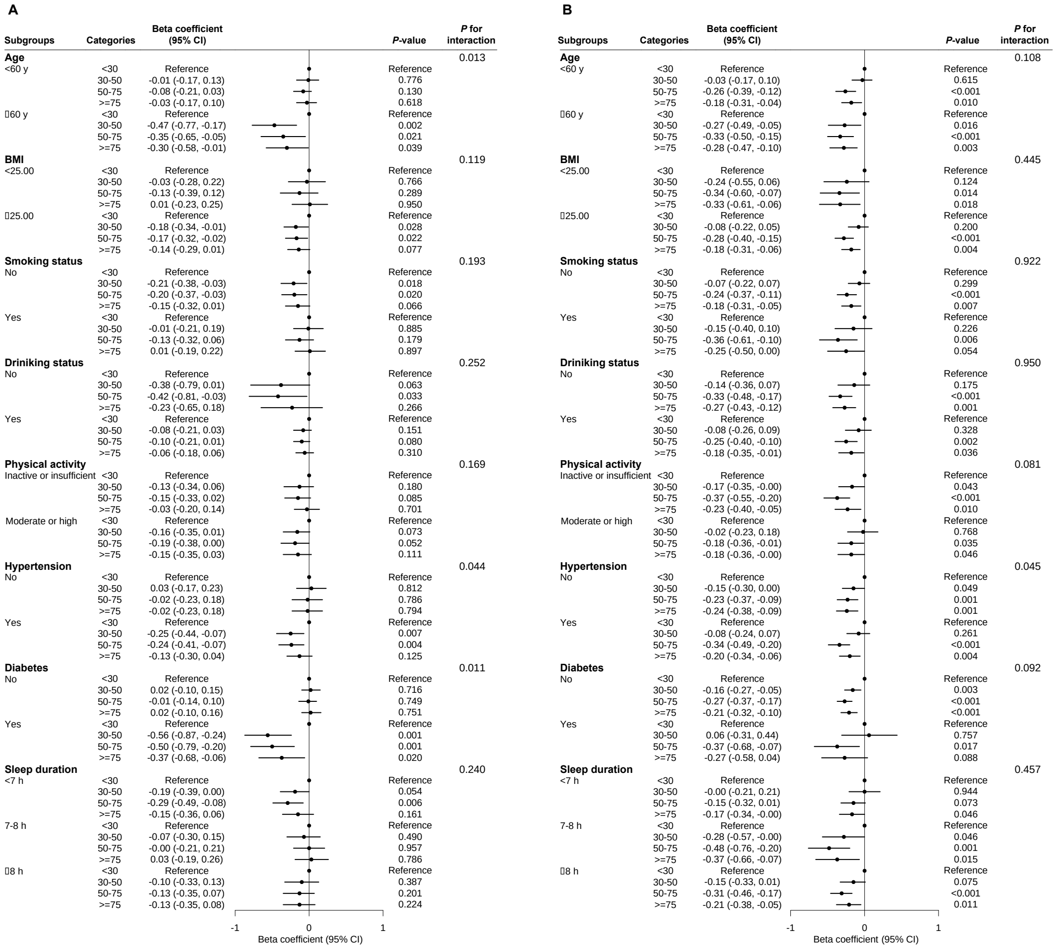

Figure 2 Subgroup analyses of the association between 25(OH)D categories and biological aging among males (A) and females (B). The filled circles and horizontal lines represent regression coefficients and the 95% confidence intervals. Multiple linear regression models were adjusted for age, race, education level, poverty-income ratio, body mass index, smoking, drinking, physical activity, sleep duration, hypertension, diabetes and season of measurement. BMI, body mass index; CI, confidence intervals.

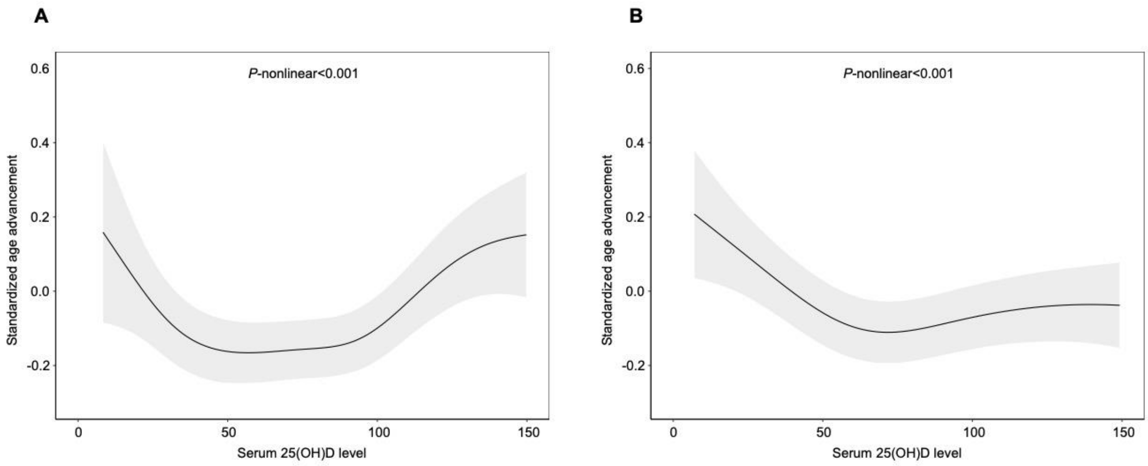

Figure 3 Nonlinear relationships between 25(OH)D serum level and biological aging among males (A) and females (B). Generalized additive models were adjusted for age, race, education level, poverty-income ratio, body mass index, smoking, drinking, physical activity, sleep duration, hypertension, diabetes and season of measurement. The black solid line represents the smooth curve fit between variables. The grey band represents the 95% confidence interval of the fit.

Table 3. Results of the analysis of the relationship between 25(OH)D serum levels and PhenoAge

25(OH)D serum levels (nmol/L)	Beta coefficient (95% CI)	*P*-value
Male		
<38.20	-0.013 (-0.025, -0.002)	0.017
38.20-91.55	0.001 (-0.001, 0.002)	0.487
⩾91.55	0.005 (0.002, 0.008)	<0.001
Female		
<62.50	-0.007 (-0.010, -0.004)	<0.001
62.50-125.00	0.002 (0.000, 0.004)	0.029
⩾125.00	-0.001 (-0.005, 0.002)	0.535

CI: confidence intervals

Segmented regression models were adjusted for age, race, education level, poverty-income ratio, body mass index, smoking, drinking, physical activity, sleep duration, hypertension, diabetes and season of measurement.

Click here to access/download
Supporting Information
Supplemental materials.docx

Click here to access/download
Other
PLOSOne_Human_Subjects_Research_Checklist.docx

Association of ~~serum~~ 25(OH)D serum level with ~~level and physiological aging~~biological aging: A Cross-Sectional Study of 2007-2016 NHANES surveys~~2007-2016~~

Yi Feng[a]; Licheng Yu[b]; Jue Wang[a] *

[a] Nanhui new town Community Health Service Center, Pudong New Area, Shanghai, 201308, China.

[b] Prevention and Health Care Department, Heqing Community Health Service Center of Pudong New District, Shanghai, 201201, China.

Corresponding author. Jue Wang

E-mail address: wangjue180130@126.com

Abstract

Vitamin D plays a crucial role in various aspects of human body, including aging, but chronological age may not accurately reflect the true ~~physiological~~ biological aging status. Recently, PhenoAge has been developed to estimate an individual's biological age based on different ~~physiological~~ biological and clinical measures. Therefore, we investigated the relationship between ~~serum 25(OH)D level~~25(OH)D serum levels and ~~physiological~~ biological aging calculated by PhenoAge ~~in~~ using data from the 2007-2016~~the National Health and Nutrition Examination Survey (~~ NHANES survey~~)~~. ~~Serum 25(OH)D level~~25(OH)D serum levels ~~Biological age~~ showed a negative ~~correlation~~ association with PhenoAge acceleration (PhenoAgeAccel) ~~serum 25(OH)D levels~~ (β=-0.04~~ standard deviation [SD]~~; 95% ~~confidence interval [~~CI~~]CI~~: -0.08~~-~~ to 0.00, p=0.039).~~,~~ ~~but t~~This negative correlation was only found to be dose-dependent for females (β=-0.07~~ SD~~; 95% CI: -0.12 to -0.01, p=0.007), but not for males. Generalized additive models further showed nonlinear associations were revealing a U-shaped relationship observed in males but an L-shaped pattern in females~~Further analysis confirmed that these associations were nonlinear in both sexes, revealing a U-shaped curve for males and an L-shaped curve for females~~. Specifically, ~~serum 25(OH)D level~~25(OH)D serum levels were linked to ~~decelerated~~ reduced PhenoAgeAccel~~aging~~ at levels below 38.20 nmol/L (15.28 ng/mL) in males (β=-0.013~~ SD~~; 95% CI: -0.025 to -0.002, p=0.017) and 62.50 nmol/L (25ng/mL) in females (β=-0.007~~ SD~~; 95% CI: -0.01 to -0.004, p<0.001). However, ~~high serum 25(OH)D level~~25(OH)D serum levels above ~~(>~~ 125 nmol/L~~)~~ were not associated~~did not correlate~~ with PhenoAgeAccel ~~aging~~ in females (β=-0.001~~ SD~~; 95% CI: -0.005-0.002), while in males, elevated levels (>91.55 nmol/L) were associated with ~~accelerated aging~~increased PhenoAgeAccel (β=0.005 SD; 95% CI: 0.002-0.008, p<0.001). Our findings indicate that vitamin D deficiency shows a potential link to accelerated ~~physiological~~ biological aging, ~~but~~ and ~~high~~ high levels of vitamin D in males link to ~~-do not link to de~~accelerated ~~physiological~~ biological aging, offering valuable insights into the relationship between vitamin D and biological aging.

Key words: Vitamin D; Serum 25(OH)D; ~~Physiological aging~~Biological aging; PhenoAge; Nonlinear ~~correlation~~association

1. Introduction

Vitamin D is a fat-soluble vitamin that plays a crucial role in various aspects of human health, with its effects extending to bone metabolism, immune function, etc, and overall physiological well being. It is acquired through two primary sources: endogenous production (mainly vitamin D23) in the skin upon exposure to ultraviolet B (UVB) radiations, and exogenous intake (vitamin D2 and D3) through dietary sources, such as fatty fish, egg yolks or supplements. Once absorbed in the bodyVitamin D is obtained, vitamin D can beis converted into 25-hydroxyvitamin D ([25(OH)D]) in the liver, then transformed into the biologically active form, 1α,25-dihydroxyvitamin D ([1α,25(OH)$_2$D)], in the kidneys [1]. This active form binds to the vitamin D receptor (VDR), regulating calcium and phosphate metabolism [2] [2, 3].

Serum 25(OH)D is the most commonly measured form of vitamin D in humans because it is the major circulating form and provides an accurate indicator of the body's overall vitamin D status The measurement of serum 25(OH)D levels is critical for evaluating vitamin D status [3], and it but can be influenced by various factors, such asincluding geographic location, race and food intake [4]. According to established criteria, serum 25(OH)D levels below 20 ng/mL (50 nmol/L) are classified as deficient, while levels between 20-30 ng/mL (50-75 nmol/L) are considered insufficient [5-7].

Vitamin D deficiency is a global public health concern, associated with increased risk of type 2 diabetes (T2D), hypertension, multiple sclerosis, etc [5-7]. The prevalence of vitamin D deficiency is particularly high among the elderly [8], with some studies suggesting that it may accelerate chronological aging and contribute to age-related diseases and– inflammatory diseases, such as cardiovascular disease, multiple sclerosis and rheumatoid arthritissuch as cardiovascular disorders – [9]. However, several large randomized clinical trials trials of vitamin D supplementation have failed to demonstrate significant benefits on cardiovascular disease, casting doubt on the evidence from observational studies linking vitamin D deficiency with cardiovascular disease observational evidence [10, 11]. This ambiguity highlights challenges in assessing the complexity of the association between vitamin D and aging. A major

Formatted: Indent: First line: 1.5 ch

Field Code Changed

Field Code Changed

Field Code Changed

Field Code Changed

Field Code Changed

Formatted: Font color: Purple

Formatted: Font color: Purple

Field Code Changed

Formatted: First line: 1.75 ch, Adjust space between Latin and Asian text, Adjust space between Asian text and numbers

Field Code Changed

Field Code Changed

Field Code Changed

Field Code Changed

Formatted: Font: (Default) Times New Roman

limitation of previous studies is that they relied on chronological age which is quite discrepant with biological age. ~~Previous studies have a limitation of reliance on chronological age, which does not account for individual differences in physiological aging.~~ Indeed, unlike the chronological age which is a precise reflection of an individual's lifetime, the biomarkers that make up the biological age are mainly determined by a combination of genetic, environmental and lifestyle factors~~While chronological age measures the passage of time, physiological aging reflects the rate of physiological decline, which can vary significantly among individuals due to genetic, environmental, and lifestyle factors~~ [12]. Accordingly, some ~~Some~~ individuals may age biologically faster or slower than indicated by their ~~their~~ chronological age ~~underscoring the need for more accurate biomarkers of aging~~.

To better characterize physiological aging in epidemiological studies~~address this challenge~~, several aging metrics based on the combination of clinical (e.g., PhenoAge and GrimAge) and biological (DNAmPhenoAge acceleration) markers have been developed ~~methods have been created, including clinical biomarkers-based approaches (PhenoAge, GrimAge etc) and methylation-based algorithms (DNAm PhenoAge acceleration, mortality risk score.etc)~~ [13]. In a post hoc analysis of the European DO-HEALTH randomized clinical trial involving 777 participants with a mean 25(OH)D level of 22.69 ng/mL, DNAm PhenoAge was the only biological age metric shown to be slowed by daily vitamin D (2000 UI) supplementation among the four biological age metrics that were assessed: DNAm PhenoAge, GrimAge, GrimAge2 and DunedinPACE [14]. PhenoAge was developed and validated using machine learning methods on a National Health and Nutrition Examination Survey (NHANES) dataset of 4000 adults aged 20 to 84 years and it showed a mean absolute error of approximately 3.6 years when predicting the chronological age [15]. The calculation of PhenoAge is based on the addition of serum levels of the following nine biomarkers on top of the chronological age. Although vitamin D supplementation was shown to slow the PhenoAge value in older adults with low 25(OH)D serum concentrations, observational studies linking 25(OH)D serum concentrations with PhenoAge are lacking. Therefore, in this study, we assessed whether there is a linear and/or non-linear relationship

Field Code Changed

Field Code Changed

Field Code Changed

Field Code Changed

between 25(OH)D serum concentration and PhenoAge using datasets of 2007-2016 NHANES surveys. By exploring this relationship, the study aims to enhance our understanding of the link between serum vitamin D levels and biological age in human beings for the benefit of public health.

~~Among these, the PhenoAge method has emerged as a promising tool, employs a machine learning algorithm to estimate an individual's biological age based on various physiological and clinical measure, including inflammation (e.g. C-reactive protein), metabolic health (e.g. glucose, albumin), and organ function (e.g. alkaline phosphatase, lymphocyte percent)~~ [19]~~. This method was developed by using data from the National Health and Nutrition Examination Survey (NHANES) and over 4,000 adults aged 20-84 years with complete biomarker data were used to train and validate the machine learning model~~ [20]~~. PhenoAge demonstrates high accuracy, with a mean absolute error of approximately 3.6 years in predicting chronological age~~ [19]~~. In a three-year longitudinal study, DO-HEALTH trial, involving 777 older adults, vitamin D supplement slows PhenoAge index, but not the other physiological aging index, including GrimAge, GrimAge2 and DunedinPACE [18]. At baseline, the mean 25(OH)D level among participants was 22.69 ng/mL, which falls within the insufficient range (20-30 ng/mL), proving that vitamin D supplement is beneficial for the elderly with vitamin D deficiency. However, the comprehensive picture of the association between vitamin D levels and physiological aging remains to be fully elucidated, particularly in the general population without vitamin D deficiency or insufficiency.~~

~~In this study, the association of serum 25(OH)D and physiological aging calculated by PhenoAge method was investigated in the NHANES data from 2007-2016. By exploring this relationship, the study aims to enhance our understanding of the potential link between vitamin D and aging, offering valuable insights into the underlying mechanisms and implications for public health.~~

2. Method

2.1 Study design and population

This study was conducted and reported in agreement ~~adherence~~ to STROBE

Field Code Changed

Field Code Changed

Field Code Changed

Field Code Changed

Formatted: Font color: Blue

guidelines for reporting observational studies. This study utilized data derived from the 2007-2016 NHANES, from National Health and Nutrition Examination Survey (NHANES), a nationwide surveys conducted in the United States of America by by the National Center for Health Statistics (NCHS) of the Centers for Disease Control and Prevention (CDC). All data included in the current study are publicly available through on the NHANES website [16]. NHANES includes in-home interview, physical measurement and laboratory tests. Written consents were provided by all participants at recruitment. This study is based on data from 50558 participants of the 2007-2016 NHANES datasets.obtained 50588 individuals of NHANES data from 2007-2016. We excluded participants with incomplete information and participants aged less than below 40 years old. A total of 13,910 participants were finally included in this analysis included for further analysis (Figure 1).

2.2 Measurement of serum 25(OH)D concentrations

Serum vitamin D levels were reflected by 25(OH)D levels. Because the forms of 25(OH)D measured in the 2007-2016 NHANES sample included both 25(OH)D2 and 25(OH)D3 (classic 25(OH)D3 and C3 epimer of 25(OH)D3 [C3-epi-25(OH)D3]), serum 25(OH)D included 25(OH)D2, 25(OH)D3 and C3-epi-25(OH)D3 in this study. During the study period (2007-2016), 25(OH)D levels were measured in the NHANES population using the liquid chromatography-tandem mass spectrometry (LC-MS/MS) Serum 25-hydroxyvitamin D [25(OH)D] concentrations were measured as a reliable index to reflect vitamin D status. From cycle 2007-2008 and beyond, liquid chromatography-tandem mass spectrometry (LC-MS/MS) was used to measure serum 25(OH)D concentrations, including 25(OH)D3, epi-25(OH)D3, and 25(OH)D2 in human serum. We calculated the 25(OH)D levels by adding 25(OH)D3 and 25(OH)D2 levels. According to the Institute of Medicine Committee recommendation, we transformed serum 25(OH)D concentration into categorical variables: risk of deficiency (<30 nmol/L), risk of inadequacy (30-50 nmol/L), likelihood of sufficiency (50-75 nmol/L) and no increased benefit (≥75 nmol/L)According to the American Endocrine

Formatted: Font: (Default) Times New Roman

Field Code Changed

Formatted: Font color: Custom Color(RGB(3,30,255))

~~Society guidelines, we transformed serum 25(OH)D concentration into categorical variables: deficiency (<50 nmol/L), insufficiency (50–75 nmol/L) and sufficiency (≥ 75 nmol/L)~~ [17, 18].

2.3 Measurement of ~~physiological aging~~biological aging

In this study, we utilized PhenoAge as the indicator of biological aging, considering its high robustness and feasibility in NHANES data as a reliable predictor of mortality and age-related disease risks. The PhenoAge algorithm is constructed using NHANES III (1988–1994) by elastic-net regression, encompasses 10 markers: albumin, creatinine, glucose, white blood cell count, lymphocyte percent, red cell distribution width, mean red cell volume, C-reactive protein, alkaline phosphatase and chronological age~~In this study, we utilized PhenoAge as the indicator of aging, considering its high robustness and feasibility in NHANES data as a reliable predictor of mortality and age-related disease risks. The PhenoAge algorithm is constructed using NHANES III (1988–1994) by elastic-net regression, encompasses 10 markers: albumin, creatinine, glucose, white blood cell count, lymphocyte percent, red cell distribution width, mean red cell volume, C-reactive protein, alkaline phosphatase and chronological age~~ [19]. The R package BioAge was used to compute PhenoAge [15]. PhenoAge was both developed and validated within NHANES populations. Meanwhile, all constituent biomarkers are routinely measured in NHANES with standardized protocols, ensuring reproducibility across study cycles. One of the markers, CRP, was unavailable from 2011 to 2018. Previous studies have applied an algorithm that excluded CRP to calculate PhenoAge, demonstrating good applicability [20, 21]. To assess the validity of using PhenoAge without CRP data, we calculated 2 PhenoAge estimates with and without C-reactive protein, using data from cycles 2007-2008 and 2009-2010, which contained all 10 markers. We found a strong correlation between 2 PhenoAge estimates with and without CRP (correlation coefficient of 0.986). Based on these findings, we proceeded with the main analysis using the PhenoAge algorithm excluding CRP, employing data from five NHANES cycles.~~One of the markers, C-reactive protein, was unavailable~~

Field Code Changed

Formatted: Font color: Custom Color(RGB(3,30,255))

Formatted: Font color: Custom Color(RGB(3,30,255))

Formatted: Font color: Custom Color(RGB(3,30,255))

Formatted: Font color: Custom Color(RGB(3,30,255))

Formatted: Font color: Custom Color(RGB(3,30,255))

Field Code Changed

~~from 2011 to 2018. According to previous studies, there is a strong correlation (correlation coefficient of 0.99) between PhenoAge measurements with and without C-reactive protein, utilizing an algorithm that excludes this marker to calculate PhenoAge~~ [25, 26]~~. Due to the absence of C-reactive protein data, we employed PhenoAge algorithm that excludes this marker in the current study.~~ We further calculated PhenoAge acceleration (PhenoAgeAccel) as the difference between PhenoAge and chronological age. A positive value of PhenoAgeAccel represents accelerated biological aging process, while a negative value represented slower biological aging process. Subsequently, PhenoAgeAccel was standardized to have a mean of 0 and a standard deviation (SD) of 1.~~We further calculated physiological aging as the difference between PhenoAge and chronological age. A positive value indicates accelerated aging, while a negative value indicates slower aging. Subsequently, physiological aging was standardized to have a mean of 0 and a standard deviation (SD) of 1.~~

2.4 Covariates

NHANES interviews were conducted by trained interviewers, where questionnaires were administered to collect information on demographics (~~[~~chronological age~~age~~, gender, race, education level, poverty income ratio (PIR)~~]~~) and lifestyle (tobacco smoking ~~smoking~~ and alcohol consumption variable~~drinking status~~, physical activity and comorbidities). Body mass index (BMI) were measured at the Mobile Examination Center.

The active tobacco smoking ~~Smoking status~~ was classified into three categories: ~~as~~ never smokers (previous smoking of a total of less than 100 cigarettes ~~had smoked <100 cigarettes in their lifetime~~), current smokers (lifetime smoking of over 100 cigarettes ~~had smoked ≥100 cigarettes in their lifetime and still smoked at the time of the interview~~) and former smokers (had smoked ≥100 cigarettes in their lifetime but had quit smoking at the time of the survey). The alcohol consumption variable ~~Drinking status~~ was classified as never drinkers (had drunk <12 drinks in their lifetime), current drinkers (had drunk ≥12 drinks in their lifetime and at least one drink in the past year)

Field Code Changed

Formatted: Indent: First line: 0.5 ch

and former drinkers (had drunk ≥12 drinks in their lifetime but no drink in the past year). Metabolic equivalent task (MET)-min per week were calculated to assess physical activity, and levels of physical activity were categorized as: inactive (no regular physical activity), insufficient (<500 MET-min activity/week), moderate (500-1,000 MET-min activity/week) and high (≥1,000 MET-min activity/week) [22]. For sleep duration, participants responded to the question: "How much sleep do you usually get at night on weekdays or workdays?" and were categorized into 3 groups:~~–~~ <7 h, 7-8 h and ≥8h). The definition of hypertension was based on self-reports~~Hypertension was ascertained by self-reporting history of hypertension~~, the use of antihypertensive drugs ~~medication for hypertension~~ or on the record of a systolic ~~physical examination (systolic~~ blood pressure≥140 mmHg with or without a ~~or~~ diastolic blood pressure≥90 mmHg~~),~~ on physical exam. The definition of diabetes mellitus was based on self-reports ~~Diabetes was ascertained by self-reporting history of diabetes~~ or on laboratory test results showing~~results from laboratory examination (~~ fasting blood glucose levels ≥126 mg/dL or HbA1c values ~~glycohemoglobin~~ ≥6.5%~~)~~.

2.5 Statistical analysis

~~Continuous data were reported as mean ± standard deviation (SD) or median (interquartile range). Categorical data were reported as numbers (percentage).~~ Weighted multivariable linear regression models were constructed to estimate the association between serum 25(OH)D status and PhenoAge~~standardized physiological aging~~. ~~Serum 25(OH)D level~~25(OH)D serum levels were log-transformed due to their skewed distribution. To evaluate whether the association between 25(OH)D serum levels and PhenoAge was not just linear but also dose-dependent potential dose-response patterns, ~~25(OH)D level~~25(OH)D serum level was analyzed ~~both~~ as both a continuous ~~variable~~ (log-transformed) and a categorical variable~~categorically through quartiles and four clinically defined sufficiency categories~~. In agreement with standards for linear regression analyses involving continuous dependent variables, the association between

Field Code Changed

Formatted: Font: (Default) Times New Roman

Formatted: Font: (Default) Times New Roman

Formatted: Font color: Blue

25(OH)D serum levels as continuous variables and PhenoAge was estimated based on beta coefficients with their 95% confidence intervals Beta coefficients with 95% confidence intervals (CIs) were reported, representing the change in dependent variable per unit increase in the independent variable. The use of beta coefficients provides interpretable estimates for linear associations when analyzing continuous dependent variables, which is widely recommended in epidemiological studies [23]. Correlation analysis was not employed because it does not account for confounding factors, whereas the primary aim of current study is to estimate the confounder-adjusted association between 25(OH)D status and physiological aging. All models were adjusted for chronological age, gender, race, education level, PIRage, race, education level, PIR, BMI, tobacco smoking and alcohol consumption variable, physical activity, smoking, drinking, physical activity, sleep duration, hypertension, diabetes and season of measurement.

To explore whether there is also a the non-linear relationship between serum 25(OH)D 25(OH)D serum levels and PhenoAgeand physiological aging, a gGeneralized aAdditive mModel (GAM) was employed to fit the smooth curve constructed. The GAMs extend traditional linear regression by replacing linear terms with smooth functions, allowing flexible modeling of complex dose-response relationships without assuming a priori parametric forms [24]. The threshold effect was further estimated using segmented regression model. This method identifies inflection points where the slope of the association between 25(OH)D and PhenoAgeAccel changes significantly. The optimal breakpoint was determined via likelihood ratio tests, comparing the goodness-of-fit between the non-segmented linear model and the segmented regression model [25].To explore the non-linear relationship between serum 25(OH)D and physiological aging, generalized additive model (GAM) was employed to fit the smooth curve. The threshold effect was further estimated using segmented regression model. A *P*-value of <0.05 was considered statistical significance. All data analyses were performed using R software version 4.2.1 (R Core Team, Vienna, Austria). R package 'BioAge' was used to calculate PhenoAge [15], while package 'mgcv' was used to conduct GAM analysis [26]. Continuous data were reported as mean

Formatted: Font color: Blue

Field Code Changed

Formatted: Font color: Custom Color(RGB(3,30,255))

Formatted: Font color: Custom Color(RGB(3,30,255))

Formatted: Font color: Custom Color(RGB(3,30,255))

Formatted: Font color: Custom Color(RGB(3,30,255))

Formatted: Font color: Custom Color(RGB(3,30,255))

Formatted: Font color: Custom Color(RGB(3,30,255))

Formatted: Font color: Custom Color(RGB(3,30,255))

Formatted: Font color: Custom Color(RGB(3,30,255))

Field Code Changed

Formatted: Font color: Custom Color(RGB(3,30,255))

Formatted: Font color: Custom Color(RGB(3,30,255))

Field Code Changed

Formatted: Font color: Custom Color(RGB(3,30,255))

Formatted: Font: (Default) Times New Roman

Field Code Changed

Field Code Changed

± standard deviation (SD) or median (interquartile range). Categorical data were reported as numbers (percentage).

2.6 Ethical considerations

This study utilized data from NHANES study, which was conducted in accordance with the principles in the Declaration of Helsinki. The NHANES protocol received prior ethical approval from the National Center for Health Statistics' Review Board. Since our study is a secondary analysis of publicly accessible, de-identified NHANES data sets, additional institutional review board approval was not required [27, 28].

Field Code Changed

3. Results

3.1 Characteristics of the study population

The current study included a total of 6836 males and 7074 females, with the characteristics of the study population presented in Table 1. There are ~~Significant~~ significant differences between males and females in the distributions of ~~basic information, such as~~ age, race, BMI, and poverty-income ratio (p < 0.001)~~, except for education level~~. Additionally, gender-specific differences were noted in behaviors influencing biological aging, such as smoking, drinking, physical activity, and sleep duration, were also observed between males and females (p < 0.001). Notably, males had a higher prevalence of diabetes than females. The median ~~serum 25(OH)D level~~25(OH)D serum level in females was ~~higher (~~66.60 nmol/L~~)~~ compared with 63.80 nmol/L in ~~than in~~ males (p<0.001)~~ (63.80 nmol/L)~~, and the PhenoAge ~~calculated from 10 clinical biomarkers~~ was lower in females than in males.~~ Furthermore, PhenoAgeAccelthe physiological aging was also lower in females compared to~~than in ~~males (p < 0.001).~~

3.2 Association ~~of~~ between ~~serum 25(OH)D level~~25(OH)D serum level with~~and~~ ~~physiological aging~~biological aging

The associations between ~~serum 25(OH)D level~~25(OH)D serum levels and PhenoAgeAccel~~physiological aging~~ ~~was analyzed and~~were presented in Table 2. After

adjusting for age, race, education level, PIR, BMI, smoking, alcohol consumptiondrinking, level of physicalphysical activity, sleep duration and presence of hypertension and/or diabetes mellitus, hypertension, and diabetes,. Sserum 25(OH)D levels physiological aging was found to be negatively associated with PhenoAgeAccel serum 25(OH)D levels (β=-0.04 SD; 95% CI: -0.08 -to 0.00). Segregating data by sexHowever, this association was statistically significant only in females (p=0.007), and but not in males (p=0.334). To further explore this relationship, the population was categorized into four groups based on quartiles of serum 25(OH)D level25(OH)D serum levelfor analysis. Higher serum 25(OH)D level25(OH)D serum levels were inversely associated with PhenoAgeAccelphysiological aging, with no apparent dose-dependent trend as the p-value for trend was not significant (p for trend =0.173). When segregating data by sex, we foundThis pattern was consistent in females but absent in males. Furthermore, the population was categorized into four groups according to the endocrine society guidelinesIMO guideline, which similarly revealed that higher serum 25(OH)D level25(OH)D serum levels were associated with reduced PhenoAgeAccelphysiological aging, especially albeit withoutwith a significant dose-dependent relationships (p=0.076) in whole population and females (p for trend =0.014). Interestingly Additionally, moderate level of 25(OH)D levels at (30-50 and 50-75 nmol/L) were associated with reduced PhenoAgeAccelphysiological aging in males categorized based on IMOendocrine society guidelines, though these statistical differences were not observed in males based on quartile categories.

Stratified analysis was conducted to furtherly revealed that investigate the impact of various factors on the relationship between serum 25(OH)D levels and PhenoAgeAccelphysiological aging. In males, no overall association was observed in males; however, in male subpopulations over 60 years of age with hypertension or diabetes, higher serum 25(OH)D level25(OH)D serum levels were associated with reduced PhenoAgeAccelslower physiological aging. Additionally, there were statistically significant interactions between serum 25(OH)D level25(OH)D serum levels and age, hypertension, or diabetes (Fig.2A). In contrast, in females, mediatorsfactors such as age, BMI, smoking, drinking, physical activity, and

Formatted: Font: (Default) Times New Roman

Formatted: Not Highlight

Formatted: Font: (Default) Times New Roman

hypertension did not alter the inverse association between ~~serum 25(OH)D level~~25(OH)D serum levels and PhenoAgeAccel~~them~~, indicating the robustness of this relationship ~~in females~~ (Fig.2B).

3.3 Association of 25(OH)D serum levels with PhenoAge on GAM~~Non-linear correlation between serum 25(OH)D level and physiological~~ ~~biological~~ ~~aging~~

Given the non-significant trend in Table 2~~,~~ indicating a non-linear correlation, ~~a generalized additive model (~~GAM~~)~~ was utilized to investigate the potential linkage between serum 25(OH)D and PhenoAgeAccel. ~~The analysis revealed~~There was a nonlinear relationship between ~~serum 25(OH)D level~~25(OH)D serum level and ~~standardized~~ PhenoAgeAccel~~physiological aging~~ in both males and females (p<0.001) (Fig 3 A-B). In men, the relationship was U-shaped with the two inflection points at 15.8 ng/mL (38.2 nmol/L) and 36.6 ng/mL (91.5 nmol/L) and in women, the segmented regression modelling confirmed a L-shaped relationship.~~In males, the relationship exhibited a U shape, while in females, it resembled more of like an L shape, as confirmed by segmented regression modeling. Two inflection points were identified in males at 38.2 nmol/L and 91.55 nmol/L.~~ Low ~~serum 25(OH)D level~~25(OH)D serum levels (<38.2 nmol/L) were negatively correlated with ~~standardized~~ PhenoAgeAccel ~~physiological aging~~ (β=-0.013 SD; 95% CI: -0.025 to ~~-~~-0.002), whereas high levels (>91.55 nmol/L) showed a positive association (β=0.005 SD; 95% CI: 0.002 to ~~-~~0.008). For females, ~~serum 25(OH)D level~~25(OH)D serum levels below 62.5 nmol/L were negatively linked to PhenoAgeAccel~~physiological aging~~ (β=-0.007 SD; 95% CI: -0.01~~-~~ to -0.004), with no significant association observed beyond 125 nmol/L (β=-0.001 SD; 95% CI: -0.005 to -0.002). Overall, low (<38.2 nmol/L) or high (>91.55 nmol/L) ~~serum 25(OH)D level~~25(OH)D serum levels in males were associated with PhenoAgeAccel~~accelerating aging~~, while in females, only low levels (<62.5 nmol/L) pose a risk factor for biological aging (Table 3).

4. Discussion

In the current study, the association between ~~serum~~ 25(OH)D serum level and

Formatted: Font color: Text 1

physiological agingbiological aging were was investigated in the 2007-2016 NHANES surveysNHANES. The analysis revealed a negative correlation association between physiological agebiological age and with serum 25(OH)D serum level, whether categorized by quartiles or according to the endocrine society's criteriaIOM guideline. Additionally, Tthe trends and association can be his correlation observed exhibited a dose-dependent relationship in females but not in the overall population or males. Non-linear associations were also found between 25(OH)D serum levels and PhenoAge in both male (U-shaped) and female (L-shaped)Further analysis confirmed these associations were nonlinear in both genders, b with a U-shaped curve in males and an L-shaped curve in females.

Numerous studies have established that serum 25(OH)D level25(OH)D serum level, representing vitamin D status in our body, are associated with various diseases or disorders, including all-cause mortality, cardiovascular diseases, and aging etc. Multiple large-scale observational studies have consistently shown a negative correlation between serum 25(OH)D level25(OH)D serum levels and all-cause mortality risk, lower serum 25(OH)D level25(OH)D serum levels are associated with an increased risk of all-cause mortality, and a dose-response relationship observed [29, 30]. Individuals with lower vitamin D levels (typically below 30 ng/mL) linked to a higher risk of all-cause mortality compared to those with adequate vitamin D status [31]. Regarding aging, studies have reported that a gradual decline in serum 25(OH)D level25(OH)D serum levels with chronological age, and vitamin D supplementation can mitigate oxidative damage and improve immune function in elder people, suggesting its potential role in slowing age-related physiological decline [32]. This decline is attributed to several factors, including decreased sun exposure, reduced capacity of the skin to synthesize vitamin D, and decreased dietary intake of vitamin D, and impaired renal function, resulting in vitamin D deficiency in older adults [33]. The synthesis of vitamin D from sun exposure vary in different races, but our study lacks this data of sun exposure. –Additionally, we also found that low serum 25(OH)D level25(OH)D serum levels were associated with increased physiological agingbiological aging, suggesting vitamin D may also play a crucial role in the regulation of the aging process. –Adequate

Field Code Changed

Field Code Changed

Formatted: Font color: Blue

Field Code Changed

Formatted: Font color: Blue

Formatted: Font color: Blue

25(OH)D levels are essential for maintaining calcium absorption and bone health preventing osteoporosis, a common age-related condition, and regulating calcium homeostasis and bone metabolism [34]. Moreover, vitamin D can interact with antioxidant and anti-inflammatory pathways, such as activating Nrf2 (nuclear factor erythroid 2-related factor 2) and SIRT1 (sirtuin 1) signaling cascades, and suppressing the production of pro-inflammatory cytokines, including interleukin-6 (IL-6) and tumor necrosis factor-alpha (TNF-α), through inhibiting nuclear factor-κB (NF-κB). These actions lead to reduction of inflammation and oxidative stress, both of which are known to contribute to the aging process [34, 35]. Therefore, vitamin D and aging have a mutual influence on each other.

Studies report that ~~The significant association between vitamin D and aging suggests that~~ vitamin D supplementation could be an effective way to reverse or mitigate ~~various~~ diseases~~ and disorders. Vitamin D supplementation may help~~, including lower blood pressure and improve endothelial function, leading to reduced risk of cardiovascular events, such as heart attacks and strokes, and improved cardiovascular outcomes [36]. A meta-analysis of 75 randomized controlled trials in 2014 revealed that vitamin D supplementation was associated with a 6% reduction in all-cause mortality [37]. Some studies suggest that the impact of vitamin D supplementation on all-cause mortality may be more pronounced in certain subgroups, such as older adults or individuals with vitamin D deficiency. For example, vitamin D supplementation was associated with a reduced risk of all-cause mortality in trials with a mean baseline 25(OH)D level <30 ng/mL (RR 0.89, 95% CI 0.80-0.99), but not in trials with a mean baseline 25(OH)D level ⩾30 ng/mL (RR 1.04, 95% CI 0.97-1.11) [38]. Thus, the~~ growing body of~~se evidence highlights the potential of vitamin D supplementation in ~~promoting overall health and~~ reducing the risk of ~~various~~ several chronic diseases, particularly in elderly people, where serum vitamin D levels tend to decline with age.

Despite its potential benefits, the use of vitamin D supplements has increased markedly, and concerns about excessive intake have emerged. the Vitamin D and

Field Code Changed

Field Code Changed

Field Code Changed

Field Code Changed

Field Code Changed

Omega-3 Trial (VITAL) study, a randomized trial involving over 25,000 participants, discovered that vitamin D supplementation did not significantly reduce the risk of all-cause mortality or lower risk of fractures among generally healthy midlife and older adults without vitamin D deficiency or osteoporosis ~~,~~ [39]. Furthermore, increased intake of vitamin D supplements by the general population and a growing number of high-dose prescriptions without medical monitoring might result in a greater risk of excessive vitamin D toxicity[40]. Our current results indicated that the correlation of ~~serum 25(OH)D level~~25(OH)D serum level and ~~physiological aging~~biological aging followed a U-shaped curve in males and an L-shaped curve in females, revealing that high serum level of 25(OH)D is not associated with decelerated aging in either males and females, and even associated with accelerated ~~physiological aging~~biological aging in males. These detrimental effects may stem from the toxicity of excessive vitamin D. When vitamin D intake is too high, serum levels of its metabolites [25(OH)D and $24,25(OH)_2D$] rise beyond the binding capacity of vitamin D binding protein, leading to an increase in the free active metabolite, $1,25(OH)_2D$. This active form reaches the VDR in the nucleus, constantly promoting gene expression that enhances calcium absorption and bone mobilization [41]. Consequently, this can result in hypercalcemia or hyperphosphatemia, which in turn suppresses initial parathyroid hormone (iPTH) level [41, 42]. Such disrupting balance of calcium and hormone may contribute to cardiovascular disease, kidney damage, and skin aging. Research reported that the working diagnosis of vitamin D toxicity is diagnosed based on total 25(OH)D concentrations of 32~80ng/mL (80~200 nmol/L) detected by immunoassay [43]. Our results found the 25(OH)D levels over ~~125~~ 91.5 nmol/L in ~~fe~~male participants were not linked to accelerated ~~physiological aging~~biological aging, approximately consistent with those reports.

Our study identified a transition point in relationship between ~~serum 25(OH)D level~~25(OH)D serum level and biological aging, with males exhibiting a threshold at 38.20 nmol/L and females at 62.50 nmol/L. Beyond this transition point, the increase in 25(OH)D level no longer associates with biological aging deceleration. Regarding the threshold of vitamin D deficiency, it remains an ongoing controversy. The Scientific Advisory

Field Code Changed
Field Code Changed
Field Code Changed
Field Code Changed
Field Code Changed
Formatted: Not Highlight
Formatted: Font color: Custom Color(RGB(3,30,255))
Formatted: Font color: Custom Color(RGB(3,30,255))

Committee on Nutrition (2016) recommended threshold at 25 nmol/L among UK population [44], whereas the IOM (2011) suggested thresholds at 30 nmol/L based on evidence in U.S. and Canadian populations [17]. Meanwhile, the European Food Safety Authority 2016 and the Endocrine Society guideline 2011 proposed higher level of 50 nmol/L as deficiency cutoff [45, 46]. The discrepancy in cutoff values stems from multiple factors, for example, different regional ultraviolet B exposure patterns, dietary practice and population-specific biological variability. Furthermore, guideline variations also reflect different focus on health outcomes, with some prioritizing musculoskeletal health [47]. In 2024, the Endocrine Society guideline retracted their previous recommendation of threshold at 50 nmol/L due to limited clinical trial evidence [48]. It also highlighted the impracticality of universal thresholds due to heterogeneity in health outcomes and population characteristics. Hence, the interpretation of 25(OH)D level threshold determination in our study should be with caution, especially when it derived from observational study in an ethnically diverse population. Nevertheless, the trend of benefits attenuation we observed is worth noting, suggesting excessive level of serum 25(OH)D may not be beneficial regarding biological aging process.

Our results reported differences of dose-response curve between in male and in female, which may be attributed to the sex hormones, especially estrogen. As women age, particularly during the menopausal transition and after menopause, their ovaries produce less estrogen, leading to a decline of estrogen levels. This decline contributes to the thinning and wrinkling of the skin, as well as changes in hair texture and growth patterns [49]. Some studies suggest that vitamin D could enhance the activity of estrogen receptors (ER) via interacting with them, potentially amplifying the anti-aging effects of estrogen in the body [50]. Vitamin D and estrogen also have a synergistic effect on bone metabolism, helping to prevent osteoporosis and fractures, especially in postmenopausal women [51]. Additionally, vitamin D regulate estrogen metabolism, leading to higher circulating estrogen levels and subsequently delay aging [50]. Therefore, high level of serum 25(OH)D may not be associated with ~~physiological aging~~biological aging in females, but the mechanisms underlying the adverse effects of

Formatted: Font color: Custom Color(RGB(3,30,255))

Field Code Changed

Formatted: Font color: Custom Color(RGB(3,30,255))

Field Code Changed

Formatted: Font color: Custom Color(RGB(3,30,255))

Field Code Changed

Formatted: Font color: Custom Color(RGB(3,30,255))

Field Code Changed

Formatted: Font color: Custom Color(RGB(3,30,255))

Field Code Changed

Formatted: Font color: Custom Color(RGB(3,30,255))

Field Code Changed

Field Code Changed

Field Code Changed

Field Code Changed

extremely high levels (>100 nmol/L) in males remain unclear. Further studies are needed to clarify the complex interplay between these two important hormonal systems.

The current study has several limitations. First, this cross-sectional study only provides information on the association of ~~serum 25(OH)D level~~25(OH)D serum level and ~~physiological aging~~biological aging, making it difficult to establish causal relationships. Secondly, the PhenoAge algorithm we applied did not contain CRP data. This could be a limitation given that CRP is a key marker of inflammation and is potentially closely associated with vitamin D status. The absence of CRP in the PhenoAge calculation might lead to underestimation of the true relationship between vitamin D status and age acceleration. To gain a more accurate understanding of this association, future studies with more comprehensive datasets should be conducted.~~Second, we applied PhenoAge method to calculate physiological aging, but the data of C-reactive protein were not available.~~ Moreover, recent evidence challenge the traditional view that serum 25(OH)D is treated as a reliable biomarker of vitamin D, though it remains the most accepted marker of vitamin D status. For example, in individuals diagnosed with diabetes or obesity, the expression of CYP2R1, the enzyme responsible for vitamin D 25-hydroxylation, are suppressed, resulting in the decrease of 25(OH)D while vitamin D level is stable [52, 53]. Finally, the gender-specific differences in results require further detailed analysis to fully understand the underlying mechanisms.

5. Conclusion

In this study, we investigated the relationship between ~~serum 25(OH)D level~~25(OH)D serum levels and biological age in NHANES, and found that there was a negative ~~correlation~~ association between ~~physiological age~~biological age and ~~serum~~ 25(OH)D serum level, and these associations were nonlinear in both genders, revealing a U-shaped curve for males and an L-shaped curve for females. Low ~~serum 25(OH)D level~~25(OH)D serum levels shows a potential link to accelerated ~~physiological aging~~biological aging in both genders, but high ~~serum~~ 25(OH)D serum levels do not correlate with decelerated ~~physiological aging~~biological aging, and even associated

Formatted: Font color: Custom Color(RGB(3,30,255))

Formatted: Font color: Blue

Formatted: Font color: Blue

Field Code Changed

with accelerated ~~physiological aging~~biological aging in male. Our results suggest that the association between vitamin D levels with ~~physiological aging~~biological aging is ~~complex~~ different in men and women~~and whether vitamin D supplementation in individuals with sufficient 25(OH)D levels is beneficial warrants further investigation~~.

Authors contributions~~Contributions~~

Yi Feng: Conceptualization; Methodology; Data curation; Formal analysis; Writing original draft**; Licheng Yu**: Data curation; Formal analysis; **Jue Wang**: Project administration; Supervision; Writing - review &editing.

Conflicts of interest

The authors declare no competing financial or non-financial interests related to this work. NHANES is a publicly available dataset managed by the Centers for Disease Control and Prevention. The authors confirm that there is no affiliation with or involvement from the NHANES program administrators in this study. The funding source had no role in study design, data collection, analysis, or manuscript preparation.

References

1. Bikle DD. Vitamin D metabolism, mechanism of action, and clinical applications. Chem Biol. 2014;21(3):319-29. Epub 2014/02/18. doi: 10.1016/j.chembiol.2013.12.016. PubMed PMID: 24529992; PubMed Central PMCID: PMCPMC3968073.
2. Dominguez LJ, Farruggia M, Veronese N, Barbagallo M. Vitamin D Sources, Metabolism, and Deficiency: Available Compounds and Guidelines for Its Treatment. Metabolites. 2021;11(4). Epub 2021/05/01. doi: 10.3390/metabo11040255. PubMed PMID: 33924215; PubMed Central PMCID: PMCPMC8074587.
3. Couchman L, Moniz CF. Analytical considerations for the biochemical assessment of vitamin D status. Ther Adv Musculoskelet Dis. 2017;9(4):97-104. Epub 2017/04/07. doi: 10.1177/1759720X17692500. PubMed PMID: 28382113; PubMed Central PMCID: PMCPMC5367642.
4. Rahman A, Elmi A. Air pollutants are negatively associated with vitamin D-synthesizing UVB radiation intensity on the ground. Sci Rep. 2021;11(1):21480. Epub 2021/11/04. doi: 10.1038/s41598-

Field Code Changed

Formatted: Font: (Default) Times New Roman

021-00980-6. PubMed PMID: 34728744; PubMed Central PMCID: PMCPMC8563978.

5. Zhao S, Qian F, Wan Z, Chen X, Pan A, Liu G. Vitamin D and major chronic diseases. Trends Endocrinol Metab. 2024. Epub 2024/06/02. doi: 10.1016/j.tem.2024.04.018. PubMed PMID: 38824035.

6. Gaksch M, Jorde R, Grimnes G, Joakimsen R, Schirmer H, Wilsgaard T, et al. Vitamin D and mortality: Individual participant data meta-analysis of standardized 25-hydroxyvitamin D in 26916 individuals from a European consortium. Plos One. 2017;12(2):e0170791. Epub 2017/02/17. doi: 10.1371/journal.pone.0170791. PubMed PMID: 28207791; PubMed Central PMCID: PMCPMC5312926.

7. Fang A, Zhao Y, Yang P, Zhang X, Giovannucci EL. Vitamin D and human health: evidence from Mendelian randomization studies. Eur J Epidemiol. 2024;39(5):467-90. Epub 2024/01/12. doi: 10.1007/s10654-023-01075-4. PubMed PMID: 38214845.

8. Cui A, Xiao P, Ma Y, Fan Z, Zhou F, Zheng J, et al. Prevalence, trend, and predictor analyses of vitamin D deficiency in the US population, 2001-2018. Front Nutr. 2022;9:965376. Epub 2022/10/21. doi: 10.3389/fnut.2022.965376. PubMed PMID: 36263304; PubMed Central PMCID: PMCPMC9573946.

9. Berridge MJ. Vitamin D deficiency accelerates ageing and age-related diseases: a novel hypothesis. The Journal of physiology. 2017;595(22):6825-36. Epub 2017/09/28. doi: 10.1113/JP274887. PubMed PMID: 28949008; PubMed Central PMCID: PMCPMC5685827.

10. Zittermann A, Ernst JB, Prokop S, Fuchs U, Dreier J, Kuhn J, et al. Effect of vitamin D on all-cause mortality in heart failure (EVITA): a 3-year randomized clinical trial with 4000 IU vitamin D daily. Eur Heart J. 2017;38(29):2279-86. Epub 2017/05/13. doi: 10.1093/eurheartj/ehx235. PubMed PMID: 28498942.

11. Scragg R, Stewart AW, Waayer D, Lawes CMM, Toop L, Sluyter J, et al. Effect of Monthly High-Dose Vitamin D Supplementation on Cardiovascular Disease in the Vitamin D Assessment Study : A Randomized Clinical Trial. JAMA Cardiol. 2017;2(6):608-16. Epub 2017/04/07. doi: 10.1001/jamacardio.2017.0175. PubMed PMID: 28384800; PubMed Central PMCID: PMCPMC5815022 ICMJE Form of Disclosure of Potential Conflicts of Interest and none were reported.

12. Xia X, Wang Y, Yu Z, Chen J, Han JJ. Assessing the rate of aging to monitor aging itself. Ageing Res Rev. 2021;69:101350. Epub 2021/05/04. doi: 10.1016/j.arr.2021.101350. PubMed PMID: 33940202.

13. Jylhava J, Pedersen NL, Hagg S. Biological Age Predictors. EBioMedicine. 2017;21:29-36. Epub 2017/04/12. doi: 10.1016/j.ebiom.2017.03.046. PubMed PMID: 28396265; PubMed Central PMCID: PMCPMC5514388.

14. Bischoff-Ferrari HA, Gangler S, Wieczorek M, Belsky DW, Ryan J, Kressig RW, et al. Individual and additive effects of vitamin D, omega-3 and exercise on DNA methylation clocks of biological aging in older adults from the DO-HEALTH trial. Nat Aging. 2025;5(3):376-85. Epub 2025/02/04. doi: 10.1038/s43587-024-00793-y. PubMed PMID: 39900648.

15. Kwon D, Belsky DW. A toolkit for quantification of biological age from blood chemistry and organ function test data: BioAge. Geroscience. 2021;43(6):2795-808. Epub 2021/11/03. doi: 10.1007/s11357-021-00480-5. PubMed PMID: 34725754; PubMed Central PMCID: PMCPMC8602613.

16. Centers for Disease Control and Prevention National Health and Nutrition Examination Survey Questionnaires D, and Related Documentation 2020. [cited 2025 March 7]. Available from: https://wwwn.cdc.gov/nchs/nhanes/.

17. . Ross AC, Taylor CL, Yaktine AL, Del Valle HB, editors. Washington (DC)2011.

18. Ross AC, Manson JE, Abrams SA, Aloia JF, Brannon PM, Clinton SK, et al. The 2011 report on

Formatted: Font: (Default) Times New Roman

Formatted: Font: (Default) Times New Roman

dietary reference intakes for calcium and vitamin D from the Institute of Medicine: what clinicians need to know. J Clin Endocrinol Metab. 2011;96(1):53-8. Epub 2010/12/02. doi: 10.1210/jc.2010-2704. PubMed PMID: 21118827; PubMed Central PMCID: PMCPMC3046611.

19. Liu Z, Kuo PL, Horvath S, Crimmins E, Ferrucci L, Levine M. A new aging measure captures morbidity and mortality risk across diverse subpopulations from NHANES IV: A cohort study. PLoS Med. 2018;15(12):e1002718. Epub 2019/01/01. doi: 10.1371/journal.pmed.1002718. PubMed PMID: 30596641; PubMed Central PMCID: PMCPMC6312200.

20. Liu W, Wang J, Wang M, Hou H, Ding X, Ma L, et al. Oxidative Stress Factors Mediate the Association Between Life's Essential 8 and Accelerated Phenotypic Aging: NHANES 2005-2018. J Gerontol A Biol Sci Med Sci. 2024;79(1). Epub 2023/10/10. doi: 10.1093/gerona/glad240. PubMed PMID: 37813096.

21. He H, Chen X, Ding Y, Chen X, He X. Composite dietary antioxidant index associated with delayed biological aging: a population-based study. Aging (Albany NY). 2024;16(1):15-27. Epub 2024/01/04. doi: 10.18632/aging.205232. PubMed PMID: 38170244; PubMed Central PMCID: PMCPMC10817368.

22. Haskell WL, Lee IM, Pate RR, Powell KE, Blair SN, Franklin BA, et al. Physical activity and public health: updated recommendation for adults from the American College of Sports Medicine and the American Heart Association. Circulation. 2007;116(9):1081-93. Epub 2007/08/03. doi: 10.1161/CIRCULATIONAHA.107.185649. PubMed PMID: 17671237.

23. Bender R. Introduction to the use of regression models in epidemiology. Cancer epidemiology: Springer; 2009. p. 179-95.

24. Hastie TJ. Generalized additive models. Statistical models in S. 2017:249-307.

25. Muggeo VM. Estimating regression models with unknown break-points. Stat Med. 2003;22(19):3055-71. Epub 2003/09/16. doi: 10.1002/sim.1545. PubMed PMID: 12973787.

26. Wood SN. Stable and efficient multiple smoothing parameter estimation for generalized additive models. Journal of the American Statistical Association. 2004;99(467):673-86.

27. Yucebilgic G, Bilgin R, Tamer L, Tukel S. Effects of lead on Na(+)-K(+) ATPase and Ca(+2) ATPase activities and lipid peroxidation in blood of workers. International journal of toxicology. 2003;22(2):95-7. doi: 10.1080/10915810305096. PubMed PMID: 12745990.

28. World Medical A. World Medical Association Declaration of Helsinki: Ethical Principles for Medical Research Involving Human Participants. JAMA. 2025;333(1):71-4. Epub 2024/10/19 20:42. doi: 10.1001/jama.2024.21972. PubMed PMID: 39425955.

29. Amrein K, Scherkl M, Hoffmann M, Neuwersch-Sommeregger S, Kostenberger M, Tmava Berisha A, et al. Vitamin D deficiency 2.0: an update on the current status worldwide. Eur J Clin Nutr. 2020;74(11):1498-513. Epub 2020/01/22. doi: 10.1038/s41430-020-0558-y. PubMed PMID: 31959942; PubMed Central PMCID: PMCPMC7091696 The other authors declare that they have no conflict of interest.

30. Liu W, Hu J, Fang Y, Wang P, Lu Y, Shen N. Vitamin D status in Mainland of China: A systematic review and meta-analysis. EClinicalMedicine. 2021;38:101017. Epub 2021/07/27. doi: 10.1016/j.eclinm.2021.101017. PubMed PMID: 34308318; PubMed Central PMCID: PMCPMC8283334.

31. Bouillon R, Manousaki D, Rosen C, Trajanoska K, Rivadeneira F, Richards JB. The health effects of vitamin D supplementation: evidence from human studies. Nat Rev Endocrinol. 2022;18(2):96-110. Epub 2021/11/25. doi: 10.1038/s41574-021-00593-z. PubMed PMID: 34815552; PubMed Central PMCID: PMCPMC8609267 Farma, Ceres and Proctor and Gamble. J.B.R. has worked as a consultant

Formatted: Font: (Default) Times New Roman, Font color: Blue

Formatted: Font: (Default) Times New Roman

to GlaxoSmithKline and Deerfield Capital. The other authors declare no competing interests.

32. Schottker B, Hagen L, Zhang Y, Gao X, Holleczek B, Gao X, et al. Serum 25-Hydroxyvitamin D Levels as an Aging Marker: Strong Associations With Age and All-Cause Mortality Independent From Telomere Length, Epigenetic Age Acceleration, and 8-Isoprostane Levels. J Gerontol A Biol Sci Med Sci. 2019;74(1):121-8. Epub 2018/10/30. doi: 10.1093/gerona/gly253. PubMed PMID: 30371905.

33. Liu L, Chen C, Lo K, Huang J, Yu Y, Huang Y, et al. Serum 25-hydroxyvitamin D, frailty, and mortality among the Chinese oldest old: Results from the CLHLS study. Nutr Metab Cardiovasc Dis. 2021;31(9):2707-15. Epub 2021/08/06. doi: 10.1016/j.numecd.2021.05.033. PubMed PMID: 34348876.

34. Veldurthy V, Wei R, Oz L, Dhawan P, Jeon YH, Christakos S. Vitamin D, calcium homeostasis and aging. Bone Res. 2016;4:16041. Epub 2016/10/30. doi: 10.1038/boneres.2016.41. PubMed PMID: 27790378; PubMed Central PMCID: PMCPMC5068478.

35. Ge D, Chen Q, Xie X, Li Q, Yang Y. Unveiling the potent effect of vitamin D: harnessing Nrf2/HO-1 signaling pathways as molecular targets to alleviate urban particulate matter-induced asthma inflammation. BMC Pulm Med. 2024;24(1):55. Epub 2024/01/26. doi: 10.1186/s12890-024-02869-2. PubMed PMID: 38273268; PubMed Central PMCID: PMCPMC10809564.

36. Pilz S, Gaksch M, Kienreich K, Grubler M, Verheyen N, Fahrleitner-Pammer A, et al. Effects of vitamin D on blood pressure and cardiovascular risk factors: a randomized controlled trial. Hypertension. 2015;65(6):1195-201. Epub 2015/03/25. doi: 10.1161/HYPERTENSIONAHA.115.05319. PubMed PMID: 25801871.

37. Chowdhury R, Kunutsor S, Vitezova A, Oliver-Williams C, Chowdhury S, Kiefte-de-Jong JC, et al. Vitamin D and risk of cause specific death: systematic review and meta-analysis of observational cohort and randomised intervention studies. BMJ. 2014;348:g1903. Epub 2014/04/03. doi: 10.1136/bmj.g1903. PubMed PMID: 24690623; PubMed Central PMCID: PMCPMC3972416 at www.icmje.org/coi_disclosure.pdf (available on request from the corresponding authors) and declare: no support from any organisation for the submitted work; no financial relationships with any organisations that might have an interest in the submitted work in the previous three years; no other relationships or activities that could appear to have influenced the submitted work.

38. Rejnmark L, Bislev LS, Cashman KD, Eiriksdottir G, Gaksch M, Grubler M, et al. Non-skeletal health effects of vitamin D supplementation: A systematic review on findings from meta-analyses summarizing trial data. Plos One. 2017;12(7):e0180512. Epub 2017/07/08. doi: 10.1371/journal.pone.0180512. PubMed PMID: 28686645; PubMed Central PMCID: PMCPMC5501555.

39. LeBoff MS, Chou SH, Ratliff KA, Cook NR, Khurana B, Kim E, et al. Supplemental Vitamin D and Incident Fractures in Midlife and Older Adults. N Engl J Med. 2022;387(4):299-309. Epub 2022/08/09. doi: 10.1056/NEJMoa2202106. PubMed PMID: 35939577; PubMed Central PMCID: PMCPMC9716639.

40. Pludowski P, Grant WB, Karras SN, Zittermann A, Pilz S. Vitamin D Supplementation: A Review of the Evidence Arguing for a Daily Dose of 2000 International Units (50 microg) of Vitamin D for Adults in the General Population. Nutrients. 2024;16(3). Epub 2024/02/10. doi: 10.3390/nu16030391. PubMed PMID: 38337676; PubMed Central PMCID: PMCPMC10857599.

41. Marcinowska-Suchowierska E, Kupisz-Urbanska M, Lukaszkiewicz J, Pludowski P, Jones G. Vitamin D Toxicity-A Clinical Perspective. Front Endocrinol (Lausanne). 2018;9:550. Epub 2018/10/09. doi: 10.3389/fendo.2018.00550. PubMed PMID: 30294301; PubMed Central PMCID: PMCPMC6158375.

Formatted: Font: (Default) Times New Roman

Formatted: Font: (Default) Times New Roman

Formatted: Font: (Default) Times New Roman, Font color: Blue

42. Batman A, Saygili ES, Yildiz D, Sen EC, Erol RS, Canat MM, et al. Risk of hypercalcemia in patients with very high serum 25-OH vitamin D levels. Int J Clin Pract. 2021;75(7):e14181. Epub 2021/03/25. doi: 10.1111/ijcp.14181. PubMed PMID: 33759301; PubMed Central PMCID: PMCPMC8250214.

43. Sajid U, Orton D, Kaufmann M, Jones G, Kline GA. Understanding Elevated Vitamin D Measurements to Uncover Hypercalcemia Etiology. Clin Chem. 2024;70(6):798-802. Epub 2024/06/03. doi: 10.1093/clinchem/hvae044. PubMed PMID: 38825340.

44. England PH. SACN vitamin D and health report 2016 [updated 2025-04-22]. Available from: https://www.gov.uk/government/publications/sacn-vitamin-d-and-health-report.

45. Turck D, Bresson J, Burlingame B. Scientific opinion on dietary reference values for vitamin D EFSA Panel on Dietetic Products, Nutrition, and Allergies (NDA). EFSA J. 2016;179.

46. Holick MF, Binkley NC, Bischoff-Ferrari HA, Gordon CM, Hanley DA, Heaney RP, et al. Evaluation, treatment, and prevention of vitamin D deficiency: an Endocrine Society clinical practice guideline. J Clin Endocrinol Metab. 2011;96(7):1911-30. Epub 2011/06/08. doi: 10.1210/jc.2011-0385. PubMed PMID: 21646368.

47. Roth DE, Abrams SA, Aloia J, Bergeron G, Bourassa MW, Brown KH, et al. Global prevalence and disease burden of vitamin D deficiency: a roadmap for action in low- and middle-income countries. Ann N Y Acad Sci. 2018;1430(1):44-79. Epub 2018/09/19. doi: 10.1111/nyas.13968. PubMed PMID: 30225965; PubMed Central PMCID: PMCPMC7309365.

48. Demay MB, Pittas AG, Bikle DD, Diab DL, Kiely ME, Lazaretti-Castro M, et al. Vitamin D for the Prevention of Disease: An Endocrine Society Clinical Practice Guideline. J Clin Endocrinol Metab. 2024. Epub 2024/06/03. doi: 10.1210/clinem/dgae290. PubMed PMID: 38828931.

49. Santoro N, Roeca C, Peters BA, Neal-Perry G. The Menopause Transition: Signs, Symptoms, and Management Options. J Clin Endocrinol Metab. 2021;106(1):1-15. Epub 2020/10/24. doi: 10.1210/clinem/dgaa764. PubMed PMID: 33095879.

50. Crescioli C. The Role of Estrogens and Vitamin D in Cardiomyocyte Protection: A Female Perspective. Biomolecules. 2021;11(12). Epub 2021/12/25. doi: 10.3390/biom11121815. PubMed PMID: 34944459; PubMed Central PMCID: PMCPMC8699224.

51. McCurdy R, Jiang X, Clarkson TB, Nudy M, Schnatz PF. Vitamin D and conjugated equine estrogen: the association with coronary artery atherosclerosis in cynomolgus monkeys. Menopause. 2016;23(5):481-7. Epub 2016/02/18. doi: 10.1097/GME.0000000000000582. PubMed PMID: 26886883.

52. Elkhwanky MS, Kummu O, Piltonen TT, Laru J, Morin-Papunen L, Mutikainen M, et al. Obesity Represses CYP2R1, the Vitamin D 25-Hydroxylase, in the Liver and Extrahepatic Tissues. JBMR Plus. 2020;4(11):e10397. Epub 2020/11/20. doi: 10.1002/jbm4.10397. PubMed PMID: 33210060; PubMed Central PMCID: PMCPMC7657391.

53. Aatsinki SM, Elkhwanky MS, Kummu O, Karpale M, Buler M, Viitala P, et al. Fasting-Induced Transcription Factors Repress Vitamin D Bioactivation, a Mechanism for Vitamin D Deficiency in Diabetes. Diabetes. 2019;68(5):918-31. Epub 2019/03/06. doi: 10.2337/db18-1050. PubMed PMID: 30833469; PubMed Central PMCID: PMCPMC6477896.

Formatted: Font: (Default) Times New Roman

Formatted: Font: (Default) Times New Roman

Formatted: Font: (Default) Times New Roman

Table 1. Characteristics of the study population

Characteristics	Male (n=6836)	Female (n=7074)	*P*-value
Age (mean±SD)	57.07 (11.34)	58.12 (11.82)	<0.001
Race			0.001[b]
Non-Hispanic white	3249 (47.53)	3288 (46.48)	
Non-Hispanic black	1360 (19.89)	1417 (20.03)	
Others	2227 (32.58)	2369 (33.49)	
Education level			0.394[b]
Below high school	1847 (27.02)	1831 (25.88)	
High school or equivalent	1588 (23.23)	1597 (22.58)	
Above high school	3401 (49.75)	3646 (51.54)	
Poverty-income ratio			<0.001[b]
≤1.0	1199 (17.54)	1447 (20.46)	
1.0-3.0	2839 (41.53)	3029 (42.82)	
>3.0	2798 (40.93)	2598 (36.73)	
BMI, kg/m^2			<0.001[b]
<25	1585 (23.19)	1841 (26.02)	
25-30	2749 (40.21)	2117 (29.93)	
≥30	2502 (36.60)	3116 (44.05)	
Smoking			<0.001[b]
Never smoker	2823 (41.30)	4297 (60.74)	
Previous smoker	2570 (37.60)	1666 (23.55)	
Current smoker	1443 (21.11)	1111 (15.71)	
Drinking (alcohol consumption)			<0.001[b]
Never drinker	527 (7.71)	1572 (22.22)	
Previous drinker	637 (9.32)	1466 (20.72)	
Current drinker	5672 (82.97)	4036 (57.05)	
Level of pPhysical activity			<0.001[b]
Inactive	1775 (25.97)	2533 (35.81)	
Insufficient	802 (11.73)	1086 (15.35)	
Moderate	680 (9.95)	842 (11.90)	
High	3579 (52.36)	2613 (36.94)	
Sleep duration			<0.001[b]
<7 h	2572 (37.62)	2445 (34.56)	
7-8h	1858 (27.18)	1812 (25.61)	
≥8 h	2406 (35.20)	2817 (39.82)	
Hypertension			0.839[b]

No	3007 (43.99)	3067 (43.36)	
Yes	3829 (56.01)	4007 (56.64)	
Diabetes			<0.001[b]
No	5086 (74.40)	5515 (77.96)	
Yes	1750 (25.60)	1559 (22.04)	
Serum 25(OH)D, nmol/L			
Median (IQR)	63.80 (48.38, 80.20)	66.60 (47.00, 87.30)	<0.001[c]
<30	420 (6.14)	545 (7.70)	<0.001[b]
30-50	1451 (21.23)	1457 (20.60)	
50-75	2766 (40.46)	2293 (32.41)	
⩾75	2199 (32.17)	2779 (39.28)	
~~Phenoage~~ PhenoAge (median [IQR])	53.77 (45.07, 64.60)	53.05 (44.23, 64.13)	0.032
PhenoAgeAccel~~Physiological aging~~ (median [IQR])	-2.40 (-5.13, 0.94)	-4.14 (-7.22, -0.46)	<0.001[c]
Standardized PhenoAgeAccel~~physiological aging~~ (mean±SD)	0.11 (0.95)	-0.16 (1.05)	<0.001[a]

~~BMI: body mass index; IQR: interquartile range; SD: standard deviation~~

~~Data were reported as number (percentage) for categorical variables; mean±SD or median (IQR) for continuous variables.~~

[a] Student's t-test was used to compare means of variables between two groups

[b] Chi-square test was used to compare categorical variables between two groups.

[c] Mann-Whitney test was used to compare non-normal variables between two groups.

Table 2. Association between ~~serum 25(OH)D level~~25(OH)D serum level and ~~physiological~~ biological aging

Characteristics	All			Male			Female		
	Beta coefficient (95% CI)		*P*-value	Beta coefficient (95% CI)		*P*-value	Beta coefficient (95% CI)		*P*-value
	Crude model	Adjusted model [a]		Crude model	Adjusted model [a]		Crude model	Adjusted model [a]	
Log(25(OH)D)	-0.27 (-0.32, -0.22)	-0.04 (-0.08, 0.00)	0.039	-0.13 (-0.20, -0.06)	0.03 (-0.02, 0.09)	0.334	-0.32 (-0.38, -0.26)	-0.07 (-0.12, -0.01)	0.007
Quartiles									
Q1	Reference	Reference		Reference	Reference		Reference	Reference	
Q2	-0.23 (-0.29, -0.17)	-0.10 (-0.16, -0.05)	<0.001	-0.16 (-0.24, -0.08)	-0.02 (-0.09, 0.04)	0.448	-0.33 (-0.41, -0.25)	-0.16 (-0.23, -0.08)	<0.001
Q3	-0.29 (-0.36, -0.23)	-0.10 (-0.16, -0.04)	0.001	-0.20 (-0.29, -0.11)	-0.03 (-0.12, 0.04)	0.355	-0.40 (-0.49, -0.32)	-0.15 (-0.22, -0.07)	<0.001
Q4	-0.32 (-0.38, -0.26)	-0.05 (-0.11, -0.00)	0.022	-0.16 (-0.24, -0.07)	0.02 (-0.04, 0.10)	0.553	-0.42 (-0.51, -0.34)	-0.10 (-0.18, -0.02)	0.009
P for trend			0.173			0.429			0.076
Categories based on the Institute of Medicine~~Endocrine Society~~ guideline									
<30	Reference	Reference		Reference	Reference		Reference	Reference	
30-50	-0.23 (-0.33, -0.13)	-0.14 (-0.23, -0.05)	0.002	-0.26 (-0.41, -0.11)	-0.15 (-0.29, -0.02)	0.024	-0.22 (-0.34, -0.09)	-0.10 (-0.21, 0.00)	0.057
50-75	-0.42 (-0.52, -0.33)	-0.21 (-0.30, -0.13)	<0.001	-0.39 (-0.53, -0.25)	-0.16 (-0.29, -0.04)	0.009	-0.51 (-0.63, -0.40)	-0.24 (-0.33, -0.15)	<0.001
⩾75	-0.46 (-0.56, -0.36)	-0.15 (-0.24, -0.06)	0.001	-0.36 (-0.50, -0.22)	-0.09 (-0.22, 0.03)	0.131	-0.54 (-0.66, -0.42)	-0.17 (-0.27, -0.07)	0.001
P for trend			0.130			0.366			0.014

[a] Adjusted for age, race, education level, poverty-income ratio, body mass index, smoking, drinking, physical activity, sleep duration,

hypertension, diabetes and season of measurement.

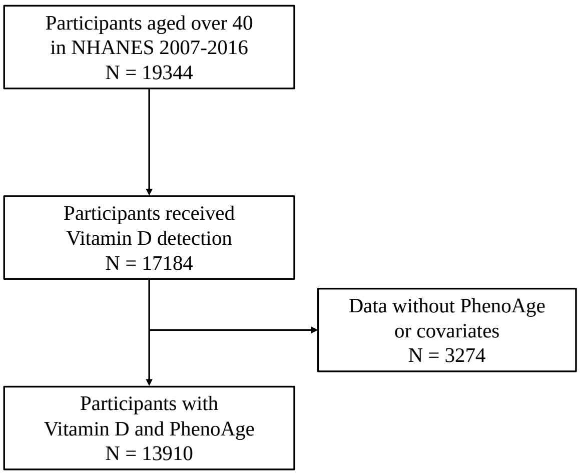

Figure 1 Flowchart of participants selection.

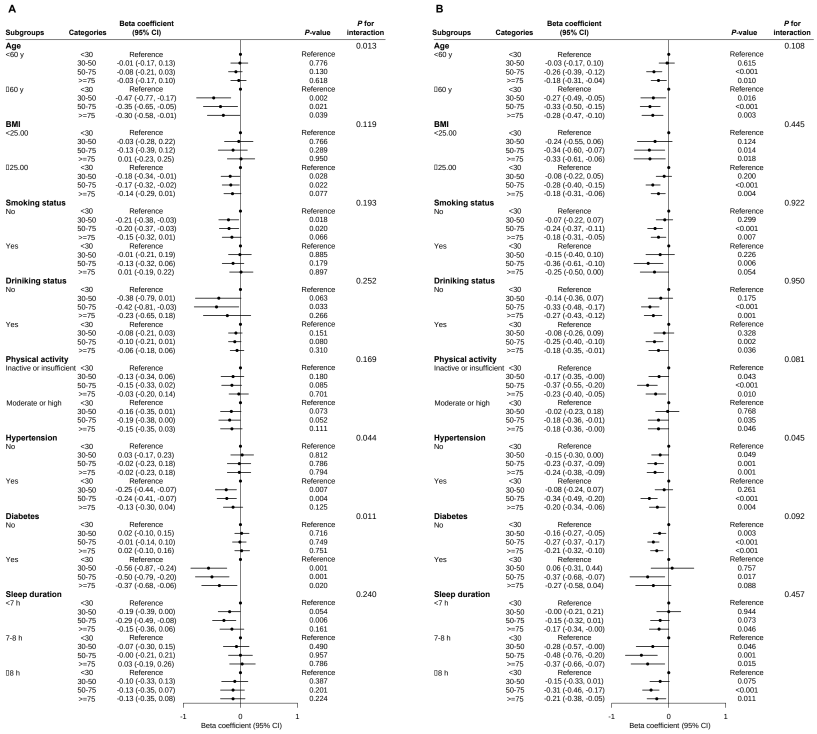

Figure 2 Subgroup analyses of the association between 25(OH)D categories and ~~physiological~~ biological aging among males (A) and females (B). The filled circles and horizontal lines represent regression coefficients and the 95% confidence intervals. Multiple linear regression models were adjusted for age, race, education level, poverty-income ratio, body mass index, smoking, drinking, physical activity, sleep duration, hypertension, diabetes and season of measurement. BMI, body mass index; CI, confidence intervals.

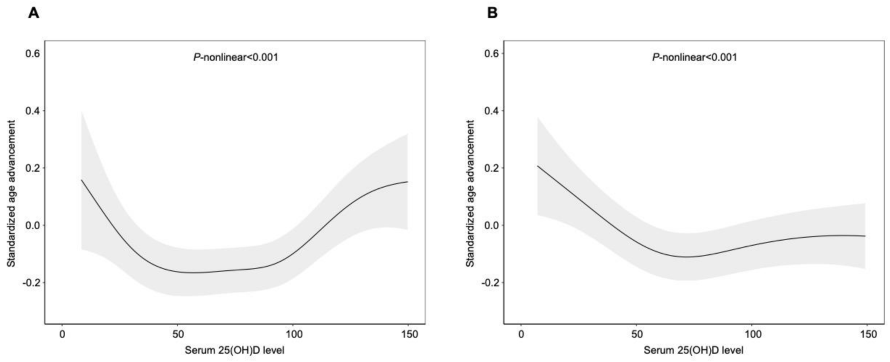

Figure 3 Nonlinear relationships between ~~serum 25(OH)D level~~25(OH)D serum level and ~~physiological~~ biological aging ~~physiological aging~~ among males (A) and females (B). Generalized additive models were adjusted for age, race, education level, poverty-income ratio, body mass index, smoking, drinking, physical activity, sleep duration, hypertension, diabetes and season of measurement. The black solid line represents the smooth curve fit between variables. The grey band represents the 95% confidence interval of the fit.

Table 3. Results of the analysis of the relationship between 25(OH)D serum levels and PhenoAge~~Threshold effect analysis of serum 25(OH)D level and physiological~~ ~~biological~~ ~~aging~~

~~Serum 25(OH)D level~~25(OH)D serum levels (nmol/L)	Beta coefficient (95% CI)	*P*-value
Male		
<38.20	-0.013 (-0.025, -0.002)	0.017
38.20-91.55	0.001 (-0.001, 0.002)	0.487
⩾91.55	0.005 (0.002, 0.008)	<0.001
Female		
<62.50	-0.007 (-0.010, -0.004)	<0.001
62.50-125.00	0.002 (0.000, 0.004)	0.029
⩾125.00	-0.001 (-0.005, 0.002)	0.535

CI: confidence intervals

Segmented regression models were adjusted for age, race, education level, poverty-income ratio, body mass index, smoking, drinking, physical activity, sleep duration, hypertension, diabetes and season of measurement.

Formatted: Font color: Custom Color(RGB(3,30,255))

Dear editor and reviewer:

Thank you very much for your careful review and constructive suggestions with regard to our manuscript. Those comments are helpful for us to revise and improve our paper. We have studied comments carefully and tried our best to revise and improve the manuscript and made great changes in the manuscript according to the good comments. We earnestly appreciate for the warm work from you, and hope that the corrections will meet with approval. Please feel free to contact us with any questions and we are looking forward to your consideration. The main corrections in the paper and the responds to your comments are as following:

Additional Editor Comments:

The authors have improved the manuscript. However, as mentioned by the reviewers, there is a room for further improvement.

Suggestions about how to address the comments of Reviewer 5:

1. The terminologies "Physiological age" and "Biological age". Although the terminology "Physiological age" is known in the health literature to be based on some bodily functions as described in the article by Nakamura et al. Further evaluation of physical fitness age versus physiological age in women. Eur J Appl Physiol (1998) 78: 195-200 for example, the authors should indeed replace the terminology "Physiological age" with "biological age" where necessary because DNAm PhenoAge, the aging metric used in this study, is a composite of biomarkers, not bodily functions.

Response: we have replaced the "Physiological age" with "biological age". Thank you for this suggestion.

2. The race issue. The authors should stick to the racial stratification used in the 2007, 2008, 2009, 2010, 2011, 2012, 2013, 2014, 2015 and 2016 NHANES surveys. Indeed, this is not the first report on 25(OH)D assessment in a multiracial NHANES population as shown by the article Daraghmeh et al. Evidence for the vitamin D hypothesis: The NHANES III extended mortality follow-up. Atherosclerosis 255 (2016) 96e101. The lack of data segregation by race could be discussed as a common study limitation of NHANES surveys (https://pubmed.ncbi.nlm.nih.gov/?term=Vitamin+D+AND+NHANES) in the absence of race-specific cut-off values for vitamin D levels as for other biological biomarkers such as the creatinine-based estimated glomerular filtration rate, and when it is unclear whether 25-hydroxyvitamin serum levels ≥ 20 ng/mL are optimal across all racial groups in the USA: see The Journal of Clinical Endocrinology & Metabolism, Volume 109, Issue 8, August 2024, Pages 1948–1954, https://doi.org/10.1210/clinem/dgae322IF: 5.0 Q1 .

Response: the discussion of race has been added in the revised manuscript. thanks for

your suggestion. **(Page14, line 371-381)**

3. The cut-off value of 25-hydroxyvitamin D for vitamin D deficiency. Given that the reviewer did not provide a reference to support the value of 25 nmol/L proposed, I recommend the authors stick to 20 ng/mL (50 nmol/L) as the cut-off value to define optimum and low levels of 25-hydroxyvitamin D in this study: see J Clin Endocrinol Metab. 2024 Jul 12;109(8):1907-1947. doi: 10.1210/clinem/dgae290IF: 5.0 Q1 . and The Journal of Clinical Endocrinology & Metabolism, Volume 109, Issue 8, August 2024, Pages 1948–1954, https://doi.org/10.1210/clinem/dgae322IF: 5.0 Q1 . However, the authors need to implement the reviewer's comment in their discussion of study limitations. In the same vein, the lack of genetic data on isoform of Vitamin D binding protein (VBD) should be discussed as a study limitation because VBD is allegedly the main determinant of the unbound 25-hydroxyvitamin D which is postulated to be the active compound of 25(OH)D (Bikle and Schwatrz. Vitamin D Binding Protein, Total and Free Vitamin D Levels in Different Physiological and Pathophysiological Conditions. Front Endocrinol (Lausanne). 2019 May 28:10:317. doi: 10.3389/fendo.2019.00317.).

Response: we have added a paragraph discussing the cut-off value of 25-hydroxyvitamin D for vitamin D deficiency. (Page 13, line 332-352)

"Regarding the threshold of vitamin D deficiency, it remains an ongoing controversy. The Scientific Advisory Committee on Nutrition (2016) recommended threshold at 25 nmol/L among UK population [46], whereas the IOM (2011) suggested thresholds at 30 nmol/L based on evidence in U.S. and Canadian populations [20]. Meanwhile, the European Food Safety Authority 2016 and the Endocrine Society guideline 2011 proposed higher level of 50 nmol/L as deficiency cutoff [47, 48]. The discrepancy in cutoff values stems from multiple factors, for example, different regional ultraviolet B exposure patterns, dietary practice and population-specific biological variability. Furthermore, guideline variations also reflect different focus on health outcomes, with some prioritizing musculoskeletal health [49]. In 2024, the Endocrine Society guideline retracted their previous recommendation of threshold at 50 nmol/L due to limited clinical trial evidence [7]. It also highlighted the impracticality of universal thresholds due to heterogeneity in health outcomes and population characteristics. Hence, the interpretation of 25(OH)D level threshold determination in our study should be with caution, especially when it derived from observational study in an ethnically diverse population. Nevertheless, the trend of benefits attenuation we observed is worth noting, suggesting excessive level of serum 25(OH)D may not be beneficial regarding biological aging process."

4. Contemporary evidence about vitamin D association with disease. As mentioned in the first round of review and further highlighted by Reviewer 5, the authors need to exercise caution when stating that vitamin D serum concentrations are associated with diseases and that vitamin D supplementation is beneficial in disease prevention. For example, see the articles by Fang A, Zhao Y, Yang P, Zhang

X, and Giovannucci EL. Vitamin D and human health: evidence from Mendelian randomization studies. Eur J Epidemiol. 2024;39(5):467-90. Epub 2024/01/12. doi: 10.1007/s10654-023-01075-4IF: 7.7 Q1 . PubMed PMID: 38214845IF: 7.7 Q1 , Sun et al. Association between vitamin D level and risk of type 2 diabetes: a systematic review of Mendelian Randomization studies. Crit Rev Food Sci Nutr. 2025 Mar 12:1-10. doi: 10.1080/10408398.2025.2466758IF: 7.3 Q1 , J Clin Endocrinol Metab. 2024 Jul 12;109(8):1907-1947. doi: 10.1210/clinem/dgae290IF: 5.0 Q1 . and The Journal of Clinical Endocrinology & Metabolism, Volume 109, Issue 8, August 2024, Pages 1948–1954, https://doi.org/10.1210/clinem/dgae322IF: 5.0 Q1 .

Response: we have checked the expression of our result and conclusion to avoid expressing causal relationships. Our study is only a cross-sectional epidemiological research.

5. The main source of vitamin D in human beings. I agree with Reviewer 5 that food intake may be the greatest source of vitamin D in White people living in the Transatlantic region where the sunny period is relatively limited. However, textbooks report vitamin D production by the skin as the main source of vitamin D in human beings, with food intake contributing to only up to 10% of the human body's store of vitamin D. The limited epidemiologic data on the source of vitamin D in the other ancestral groups in the transatlantic region and in tropical regions (for example, see the articles by Vearing, R.M., Hart, K.H., Darling, A.L. et al. Global Perspective of the Vitamin D Status of African-Caribbean Populations: A Systematic Review and Meta-analysis. Eur J Clin Nutr 76, 516–526 (2022). https://doi.org/10.1038/s41430-021-00980-9IF: 3.6 Q2 and Lancet Glob Health. 2020 Jan;8(1):e134-e142. doi: 10.1016/S2214-109X(19)30457-7IF: 19.9 Q1 . Epub 2019 Nov 27) does not allow the verification of this textbook knowledge in these other ancestral groups. However, vitamin D production by the skin could be the predominant source of vitamin D in the non-White population (at least those with a melanoderm skin), given their much higher exposure to sun-derived UVB radiations throughout the year with co-existent undernutrition when compared with White populations.I have also appended additional comments in the attached pdf.

Response: sun exposure and food intake are main sources of vitamin D. We totally agree this comment from reviewer and editor. The discussion has been added in the section of limitation. We have carefully read the additional comments added from editor. thank you so much for helping us improving our manuscript.

Reviewers' comments:

Reviewer #1: All revisions were made and there is no further comment. The revised manuscript meet the journal publish standard.

Response: Thank you so much.

Reviewer #2: There is no comment. In my opinion, the present version of the

manuscript is ready for publication. However, please check the plagiarism of the whole text and be sure that the similarity index was less than 30 %.

Response: Thank you so much. We have checked the plagiarism of manuscript and it meets the journal standards.

Reviewer #3: Authors addressed most of the previous comments from reviewers. The manuscript reads better in terms of flow and clarity. However further language editing is still required in many sections throughout the manuscript.

1. In the abstract, authors to reflect the U shape effect observed among the males; improve clarity of sentences

Response: we are sorry for this language problem. In the revised abstract, we clearly state the U-shaped effect, provides context for its significance, and ensures each sentence is concise and informative.

2. In methodology, citing previous publications, authors to briefly elaborate on each biomarkers/model. At present, there's gap of information for these modelling.

Response: We thank the reviewer for your constructive comments. To fully elucidate the rationale for selecting analysis models, we have supplemented the following sentences in the **Methods** section: "To explore the non-linear relationship between serum 25(OH)D and physiological aging, generalized additive model (GAM) was constructed. The GAMs extend traditional linear regression by replacing linear terms with smooth functions, allowing flexible modeling of complex dose-response relationships without assuming a priori parametric forms. The threshold effect was further estimated using segmented regression model. This method identifies inflection points where the slope of the association between 25(OH)D and PhenoAgeAccel changes significantly. The optimal breakpoint was determined via likelihood ratio tests, comparing the goodness-of-fit between the non-segmented linear model and the segmented regression model." **(Page 7, line 180-189).**

The following references were supplemented in the References List:

28 Hastie TJ. Generalized additive models. Statistical models in S. 2017:249-307.

29 Muggeo VM. Estimating regression models with unknown break-points. Stat Med. 2003;22(19):3055-71. Epub 2003/09/16. doi: 10.1002/sim.1545. PubMed PMID: 12973787.

3. Is there methodology established to determine physiological aging? if there is, how does the PhenoAge algorithm fare when compared? This would further establish validity of methodology.

Response: We thank the reviewer for your constructive comments. In agree with the editor's suggestion, the terminology has been revised from "physiological aging" to "biological aging" throughout the manuscript, as this better aligns with the definition of PhenoAge—a composite metric integrating 10 clinical biomarkers that collectively reflect systemic inflammatory, metabolic, and hematopoietic dysfunction. There are

several established methods for quantification of biological aging, including molecular clocks (e.g., DNA methylation clocks), functional decline indices (e.g., frailty index) and composite biomarker metrics (e.g., PhenoAge). The PhenoAge algorithm was constructed based on NHANES III (1988–1994), and was validated using data form NHANES IV (1999–2010). Hence, in NHANES dataset, the PhenoAge method showed good data compatibility and epidemiological validity. In the **Methods** section, we have supplemented the following sentences to elucidate the rationale for choosing PhenoAge as biological aging indicator: "In this study, we utilized PhenoAge as the indicator of biological aging, considering its high robustness and feasibility in NHANES data as a reliable predictor of mortality and age-related disease risks. The PhenoAge algorithm is constructed using NHANES III (1988–1994) by elastic-net regression, encompasses 10 markers: albumin, creatinine, glucose, white blood cell count, lymphocyte percent, red cell distribution width, mean red cell volume, C-reactive protein, alkaline phosphatase and chronological age [21]. PhenoAge was both developed and validated within NHANES populations. Meanwhile, all constituent biomarkers are routinely measured in NHANES with standardized protocols, ensuring reproducibility across study cycles." **(Page 5, line 118-140).**

4. In discussion and results, there's inconsistency in reporting ie ie dose-dependent relationship in females

Response: we have improved this description in the section of discussion and results. Thank you for your suggestion.

5. In discussion, further strengthening of write up required. At present, it is unclear if low vit d level drives/associated with physiological ageing or is an impact of ageing process.

Response: Numerous evidences have found that vitamin D may play a role in modulating key biological pathways associated with chronological age. Vitamin D is known to influence cellular senescence, oxidative stress, and inflammation, which are central to the aging process. Furthermore, experimental studies have demonstrated that vitamin D supplementation can mitigate oxidative damage and improve immune function in elder, suggesting its potential role in slowing age-related physiological decline. Thus, we did not provide enough information about this aspect. In the revised manuscript, we have added this in the discussion. Moreover, we have asked professional help to improve our language. Thank you so much for your review.

Reviewer #4: Feng et al. investigated the association between vitamin D deficiency and physiological aging. While the authors have made considerable efforts to address previous reviewers' comments, I believe the manuscript still requires further revision. Below are my comments:

1. The Introduction remains somewhat lengthy. Additionally, please clarify that endogenous vitamin D production in humans and animals yields vitamin D3, not D2 (see line 43).

Response: thank you for your suggestion. The revised introduction is more streamlined, removing unnecessary details while maintaining essential information.

Vitamin D exists in two primary forms: vitamin D3 (cholecalciferol) and vitamin D2 (ergocalciferol). D3 is produced from 7-dehydrocholesterol (7-DHC) through a two-step process in which the B ring is broken by UV light (spectrum 280–320 UVB) radiation from the sun, forming pre-D3 that isomerizes to D3 in a thermo-sensitive but noncatalytic process. Both UVB intensity and skin pigmentation level contribute to the rate of D3 formation. Vitamin D3 is synthesized endogenously in the skin of humans and animals upon sunlight exposure, while vitamin D2 is primarily derived from dietary sources.

2. Appropriate references should be added for the activation steps of vitamin D, including both the 25-hydroxylation and 1α-hydroxylation processes.

Response: we have checked the reference and added appropriate one in the section of introduction.

3. Line 47: Please correct the terminology to "1α,25-dihydroxyvitamin D."

Response: Sorry for this mistake and we have corrected it. Thank you so much.

4. Line 49: Choose either reference 1 or 2, citing both is unnecessary.

Response: In the revised manuscript, reference 2 was removed.

5. The criteria for defining vitamin D status are based solely on the Endocrine Society guidelines. The Institute of Medicine (IOM) guidelines, which differ substantially and are widely used in clinical settings, should also be mentioned. See ref below:

A. Catharine Ross, JoAnn E. Manson, Steven A. Abrams, John F. Aloia, Patsy M. Brannon, Steven K. Clinton, Ramon A. Durazo-Arvizu, J. Christopher Gallagher, Richard L. Gallo, Glenville Jones, Christopher S. Kovacs, Susan T. Mayne, Clifford J. Rosen, Sue A. Shapses, The 2011 Report on Dietary Reference Intakes for Calcium and Vitamin D from the Institute of Medicine: What Clinicians Need to Know, The Journal of Clinical Endocrinology & Metabolism, Volume 96, Issue 1, 1 January 2011, Pages 53–58, https://doi.org/10.1210/jc.2010-2704IF: 5.0 Q1

Response: We thank the reviewer for your insightful comment. The Institute of Medicine (IOM) guidelines define serum 25(OH)D thresholds based on musculoskeletal health outcomes, including calcium absorption efficiency, fracture risk, and prevention of rickets/osteomalacia [1]. Their criteria, widely adopted in clinical practice for U.S. and Canadian populations, align with the NHANES data characteristics. The IOM classification are as follows: risk of deficiency (<30 nmol/L), risk of inadequacy (30–50 nmol/L), likelihood of sufficiency (50–75 nmol/L) and no increased benefit (≥75 nmol/L). These thresholds are evidence-based and regionally validated, making them appropriate for our exploratory analysis.

While the 2011 Endocrine Society guidelines proposed higher thresholds (sufficiency: ≥75 nmol/L and insufficiency: 50-75 nmol/L), their 2024 update withdrew these

definitions due to insufficient clinical trial evidence linking specific 25(OH)D levels to net health benefits [2]. Hence, for operational clarity in cross-sectional analysis, we prioritized the IOM criteria. In the **Methods**, we revised the classification as follows: "According to the Institute of Medicine Committee recommendation, we transformed serum 25(OH)D concentration into categorical variables: risk of deficiency (<30 nmol/L), risk of inadequacy (30–50 nmol/L), likelihood of sufficiency (50–75 nmol/L) and no increased benefit (≥75 nmol/L) [20,21]." **(Page 5-6, line 112-115).**

The following reference was supplemented in the References List:

In: Ross AC, Taylor CL, Yaktine AL, Del Valle HB, editors. Dietary Reference Intakes for Calcium and Vitamin D. The National Academies Collection: Reports funded by National Institutes of Health. Washington (DC)2011.

Ross AC, Manson JE, Abrams SA, Aloia JF, Brannon PM, Clinton SK, et al. The 2011 report on dietary reference intakes for calcium and vitamin D from the Institute of Medicine: what clinicians need to know. J Clin Endocrinol Metab. 2011;96(1):53-8. Epub 2010/12/02. doi: 10.1210/jc.2010-2704. PubMed PMID: 21118827; PubMed Central PMCID: PMCPMC3046611.

6. The rationale for the study remains unclear. The authors should clarify their aims more.

Response: we have rewritten the introduction and improved clarification of our aims and background introduction (**Page 4, line 82-85**). We hope the improvement could make them clear and rational. Thank you very much.

7. The sentence: "However, the comprehensive picture of the association between vitamin D levels and physiological aging remains to be fully elucidated, particularly in the general population without vitamin D deficiency or insufficiency" is vague. Please revise to clarify what is meant by "general population without deficiency or insufficiency."

Response: we want to investigate the relationship between vitamin D and biological aging in the general population, whether they have sufficient or insufficient vitamin D, as most of previous studies focus on the population with insufficient vitamin D, which may contribute to harmful effects on health. The former sentence has been rewritten as "However, the comprehensive picture of the association between vitamin D levels and biological aging remains to be fully elucidated, particularly in the general population with vitamin D levels within the normal range."

8. Although several covariates were adjusted for, the possibility of residual confounding, particularly from factors such as sun exposure and vitamin D supplementation, should be acknowledged in the discussion.

Response: we totally agree with your opinion. sun exposure and vitamin D supplementation greatly affect vitamin D level in body. We have added the information in the section of discussion and limitation. (**Page 14, line371-381**)

9. Recent research has provided new insights into vitamin D metabolism and its regulation by metabolic state:
Aatsinki and Elkhwanky et al. (2019) demonstrated that fasting and diabetes activate at least two transcriptional regulators i.e., PGC-1α/ERRα and the glucocorticoid receptor (GR), which repress CYP2R1 and thereby reduce bioactivation of vitamin D. These findings question the long-held assumption that vitamin D levels solely reflect intake and sun exposure.
Elkhwanky et al. (2020) showed that obesity suppresses CYP2R1, the enzyme responsible for vitamin D 25-hydroxylation, in both hepatic and extrahepatic tissues.

These two references below need to be cited and discussed in the discussion section:
Aatsinki SM*, Elkhwanky MS*, Kummu O, Karpale M, Buler M, Viitala P, Rinne V, Mutikainen M, Tavi P, Franko A, Wiesner RJ, Chambers KT, Finck BN & Hakkola J
(2019) Fasting-Induced Transcription Factors Repress Vitamin D Bioactivation, a Mechanism for Vitamin D Deficiency in Diabetes. Diabetes 68(5): 918-931. https://doi.org/10.2337/db18-1050IF: 6.2 Q1 .
Elkhwanky MS, Kummu O, Piltonen TT, Laru J, Morin-Papunen L, Mutikainen M,
Tavi P & Hakkola J (2020) Obesity Represses CYP2R1, the Vitamin D 25-Hydroxylase, in the Liver and Extrahepatic Tissues. JBMR PLUS https://doi.org/10.1002/jbm4.10397IF: 3.4 Q2 .
These findings collectively challenge the traditional view that 25-hydroxylation is a stable, unregulated process, emphasizing instead the influence of metabolic state on vitamin D status. They also raise important questions about the suitability of 25(OH)D as a reliable biomarker in populations with metabolic disorders including diabetes. While 25(OH)D remains the most accepted marker of vitamin D status, these studies should be cited and discussed, particularly in relation to the study's diabetic subgroup
Response: Thank you very much for your insightful suggestion. This is an important issue and we totally agree that 25(OH)D may be not suitable for any conditions as a biomarker of vitamin D, though it is widely used. We have added this part in the limitation of this study (**Page 14, line371-381**). "Moreover, recent evidence challenge the traditional view that serum 25(OH)D is treated as a reliable biomarker of vitamin D, though it remains the most accepted marker of vitamin D status. For example, in individuals diagnosed with diabetes or obesity, the expression of CYP2R1, the enzyme responsible for vitamin D 25-hydroxylation, are suppressed, resulting in the decrease of 25(OH)D while vitamin D level is stable."

10. Line 261: Please remove either "diseases" or "disorders" to avoid redundancy.
Response: we have removed the "disorders" to avoid redundancy.

11. Lines 313–316: A reference on vitamin D toxicity should be included to support

the statement.

Response: a reference on vitamin D toxicity has been added at this paragraph.

43. Marcinowska-Suchowierska E, Kupisz-Urbanska M, Lukaszkiewicz J, Pludowski P, Jones G. Vitamin D Toxicity-A Clinical Perspective. Front Endocrinol (Lausanne). 2018;9:550. Epub 2018/10/09. doi: 10.3389/fendo.2018.00550. PubMed PMID: 30294301; PubMed Central PMCID: PMCPMC6158375.

12. Some phrases still imply causality (e.g., "vitamin D deficiency promotes aging"). Since this is a cross-sectional study, causal language must be avoided.

Response: we are sorry for this mistake, and correct it.

13. The suggestion that high vitamin D levels may accelerate aging in men appears speculative and requires either stronger supporting evidence or a more cautious tone.

Response: we totally agree with your opinion. Our study could not conclude that high vitamin D levels may accelerate aging in men, and just found an interesting relationship between high vitamin D levels and biological aging. Further animal and clinical researches should be carried out to clarify this conclusion.

14. English language revision is recommended.

Response: we have done our best to solve this academic writing problem in the first revision. To further improve our writing, we have asked professional help. Thank you very much for your valuable review.

In summary, although this is a timely and relevant study with notable improvements, several key areas including the clarity of the rationale, the discussion of mechanistic insights, and the interpretation of results, still require revision. Therefore, I recommend a major revision.

<u>Reviewer #5</u>: This is a study on chronological and biological aging and 25(OH-D-vitamin based on data from NHANES.

Major issues:

1. The first problem is how the authors treat age. Chronological age is simple, calculated biological age is also relevant. Phenoage is not used as defined by omitting some of the variables defining biological age.

Response: We appreciate the reviewer’s insightful opinion. The algorithm we used to calculate PhenoAge omitted CRP values. For the current analysis, we used 5 cycles to achieve adequate sample size, 3 of which were absent of CRP data. We conducted additional correlation analyses between 2 PhenoAge estimates with and without CRP values. We found a high correlation of 0.986, suggesting that the exclusion of CRP did not significantly alter the overall estimates of PhenoAge. Additionally, this practice of omitting CRP was referenced form previous researches using the same NHANES

datasets [3, 4].

We supplemented the following sentences in the **Methods**: "In this study, we utilized PhenoAge as the indicator of biological aging, considering its high robustness and feasibility in NHANES data as a reliable predictor of mortality and age-related disease risks. The PhenoAge algorithm is constructed using NHANES III (1988–1994) by elastic-net regression, encompasses 10 markers: albumin, creatinine, glucose, white blood cell count, lymphocyte percent, red cell distribution width, mean red cell volume, C-reactive protein, alkaline phosphatase and chronological age [22]. The R package BioAge was used to compute PhenoAge [16]. PhenoAge was both developed and validated within NHANES populations. Meanwhile, all constituent biomarkers are routinely measured in NHANES with standardized protocols, ensuring reproducibility across study cycles. One of the markers, CRP, was unavailable from 2011 to 2018. Previous studies have applied an algorithm that excluded CRP to calculate PhenoAge, demonstrating good applicability [23, 24]. To assess the validity of using PhenoAge without CRP data, we calculated 2 PhenoAge estimates with and without C-reactive protein, using data from cycles 2007-2008 and 2009-2010, which contained all 10 markers. We found a strong correlation between 2 PhenoAge estimates with and without CRP (correlation coefficient of 0.986). Based on these findings, we proceeded with the main analysis using the PhenoAge algorithm excluding CRP, employing data from five NHANES cycles.". **(Page 5-6, line 118-140).**

Nevertheless, we acknowledge that this is a limitation of the current study and have explicitly acknowledged it in the **Discussion**: "Secondly, the PhenoAge algorithm we applied did not contain CRP data. This could be a limitation given that CRP is a key marker of inflammation and is potentially closely associated with vitamin D status. The absence of CRP in the PhenoAge calculation might lead to underestimation of the true relationship between vitamin D status and age acceleration. To gain a more accurate understanding of this association, future studies with more comprehensive datasets should be conducted.". **(Page14, line371-381).**

The following reference was supplemented in the References List:

23. Liu W, Wang J, Wang M, Hou H, Ding X, Ma L, et al. Oxidative Stress Factors Mediate the Association Between Life's Essential 8 and Accelerated Phenotypic Aging: NHANES 2005-2018. J Gerontol A Biol Sci Med Sci. 2024;79(1). Epub 2023/10/10. doi: 10.1093/gerona/glad240. PubMed PMID: 37813096.

24. He H, Chen X, Ding Y, Chen X, He X. Composite dietary antioxidant index associated with delayed biological aging: a population-based study. Aging (Albany NY). 2024;16(1):15-27. Epub 2024/01/04. doi: 10.18632/aging.205232. PubMed PMID: 38170244; PubMed Central PMCID: PMCPMC10817368.

2. The authors also define a concept called physiological age, which is also referenced in the title. This is a major problem.

Page 16 line "We further calculated physiological aging as the difference between PhenoAge and chronological age."

This is a confusing terminology, not only for the readers, but the authors also have difficulties with this:

In the abstract line 21 "Biological age showed a negative correlation with serum 25(OH)D levels (β=-0.04; 95% CI: -0.08-0.00, p=0.039)"
In the results page 18 line 211: "physiological aging was found to be negatively associated with serum 25(OH)D levels (β=-0.04; 95% CI: -0.08-0.00)."
Omit the term' physiological age' and name it for what it really is.
Write instead: The difference between Phenoage and age was negatively correlated serum 25(OH)D levels …

Response: We thank the reviewer for your insightful suggestions.

In agree with the editor's suggestion, the terminology has been revised from "physiological aging" to "biological aging" throughout the manuscript, as this better aligns with the definition of PhenoAge—a composite metric integrating 10 clinical biomarkers that collectively reflect systemic inflammatory, metabolic, and hematopoietic dysfunction. In regression models, the standardized difference between PhenoAge and chronological age was included as the dependent variable. To enhance conciseness, we used the academic term "PhenoAgeAccel" as previously used by other literature [5, 6]. Throughout the Results, we consistently use "PhenoAgeAccel". When discussing accelerated biological aging processes, we used the term "biological aging". These revisions have been made throughout the whole manuscript.

In the **Methods** section, we have rewritten the following sentences to improve clarity: "We further calculated PhenoAge acceleration (PhenoAgeAccel) as the difference between PhenoAge and chronological age. A positive value of PhenoAgeAccel represents accelerated biological aging process, while a negative value represented slower biological aging process. Subsequently, PhenoAgeAccel was standardized to have a mean of 0 and a standard deviation (SD) of 1." **(Page 6, line 125-140).**

Major cohort problem

3. In NHANES, as in the rest of the US, race has been defined in different ways during the years. For example if one of the parents are of different races and one is white, the child is classified according to the race of the other parent. When neither parent is white the child is classified according to the fathers race. Other ways of defining race are in use, such as self-stated, decision by the doctor, or even name analysis. This is a major problem when discussing vitamin D in relation in a mixed cohort like the one used by the author. How big percentage would define race?
As has been thoroughly discussed about the inherent errors caused by race-adjusted equations for estimating glomerular filtration rate based on creatinine, this also applies to a study on vitamin D.

Response: Thank you for your insightful comment regarding the classification of race in NHANES. Race definition indeed varies based on several criteria, including parental race and self-identification. These different definitions can lead to inconsistencies when analyzing health outcomes, such as vitamin D levels, across diverse populations. Understanding these complexities is essential for accurate interpretation of results and

their implications in public health contexts. The lack of data segregation by race is a common study limitation of NHANES surveys in the absence of race-specific cut-off values for vitamin D levels as for other biological biomarkers such as the creatinine-based estimated glomerular filtration rate. Racial stratification has been used in studies of NHANES surveys, this is not the first report on 25(OH)D assessment in a multiracial NHANES population [7]. However, it should be more careful to use this race data, and we have added this in the limitation in the revised manuscript.

4. The main determinant for 25(OH)-D-vitamin in serum/plasma is a genetic variant of vitamin D-binding protein = GC-globulin. There are three isoforms, Gc1, Gc1-2, and Gc2-2 isoforms. The genetic distribution of these isoforms is highly dependent on ethic origin. The three isoforms come with different 25(OH)-D vitamin levels, although this does not affect bioavailable vitamin D not measured in this and many other studies. Thus, there would be a need for different cut-offs for deficiency, depending on the isoforms. For example, vitamin D is associated with colorectal cancer only in one of these isoforms, and the isoforms modify the risk for bladder cancer.

Thus, it can be concluded that the NHANES study is not well suited for this kind of study applying knowledge from the last ten years. Perhaps if using whites only, but it is not biologically accurate to use a composite population like the authors do.

Response: Thank you for your insightful comments regarding the influence of genetic variants of GC-globulin on serum levels of 25(OH)-D and the implications for different ethnic groups. This is an amazing idea applying different cut-offs for vitamin D deficiency depending on the isoforms. We acknowledge that the genetic distribution of these isoforms can significantly impact vitamin D levels and its associated health outcomes. However, we believe that our study's focus on the broader population sampling, including diverse ethnic backgrounds. Current analyses rely on total 25(OH)-D levels for practicality and this is the standard measurement. Several studies have investigated the role of vitamin D in various diseases based on the NHANES data [7-9]. Apparently, it would be better to analyze the isoform levels (Gc1, Gc1-2, Gc2-2) for a more detailed investigation, but NHANES currently lacks this data. With technological advancements and increasing demand, we hope that NHANES could expand and refine its relevant indicators to better accommodate various research needs.

5. The authors state incorrectly that vitamin D is associated with cardiovascular disease and osteoporosis. This has been proven incorrect over the last decade. There remains an association with some inflammatory diseases such as multiple sclerosis and rheumatoid arthritis.

There is no "growing body of evidence" to support a statement of the potential of vitamin D supplementation to promote overall health.

Response: studies found that vitamin D deficiency was associated with cardiovascular disease and osteoporosis. However, several large contemporary trials of vitamin D supplementation have failed to demonstrate significant benefits on cardiovascular

disease, casting doubt on the observational evidence, as we described in the section of introduction. We agree with your comment, and rewrite this sentence to make it appropriate. Thank you very much.
As more evidence have been provided, the protective role of vitamin D or vitamin D supplementation has been challenged in some diseases. We need to exercise caution when stating that vitamin D serum concentrations are associated with diseases and that vitamin D supplementation is beneficial in disease prevention [10].
"These evidence highlights the potential of vitamin D supplementation in reducing the risk of several chronic diseases, particularly in elderly people, where serum vitamin D levels tend to decline with age."

Minor issues:

6. Vitamin D is more common in southern compared to northern Europe (PMID: 34933700IF: 3.0 Q2), as vitamin D is probably more dependent on food intake compared to sun exposure.
Response: Thank you for your thoughtful comments. We agree with your opinion about the source of Vitamin D. studies report that the main source of vitamin D in human beings is vitamin D production by the skin, while food intake contributing to only up to 10% of the human body's store of vitamin D. however, in White people living in high latitude regions, food intake is the main source of vitamin D, as they are exposed to less sun-derived UVB radiations compared with other non-White population. In the current study, we analyzed the association of serum vitamin D level and biological aging, but did not investigate whether vitamin D is from sun exposure or food intake.

7. The cut-off for deficiency at 50 nmol per liter is not solid, it is a consensus not anchored in facts. A more likely cut-off would be 25 nmol/L.
Response: We appreciate the reviewer's insightful comment. We fully recognize that vitamin D deficiency definitions remain contentious worldwide. Multiple scientific organizing committees have proposed distinct cutoff values for vitamin D deficiency status. Their suggestions stemmed from evidence in different target population, which means various factors would affect the population vitamin D status, such as UVB exposure, dietary habits, and genetic factors influencing vitamin D metabolism. In the current analysis, we categorize vitamin D level based on the Institute of Medicine (IOM) guidelines [1]. The IOM guidelines specifically addressed U.S. and Canadian populations with considerations for bone health outcomes, directly corresponding to the NHANES cohort's demographic characteristics. Hence, for operational clarity in cross-sectional analysis, we chose to reference the IOM criteria. In the **Methods**, we have rewritten the following sentences to improve clarity: "According to the Institute of Medicine Committee recommendation, we transformed serum 25(OH)D concentration into categorical variables: risk of deficiency (<30 nmol/L), risk of inadequacy (30–50 nmol/L), likelihood of sufficiency (50–75 nmol/L) and no increased benefit (≥75 nmol/L) [20,21]." **(Page 6, line 112-115).**
The following reference was supplemented in the References List:

20. In: Ross AC, Taylor CL, Yaktine AL, Del Valle HB, editors. Dietary Reference Intakes for Calcium and Vitamin D. The National Academies Collection: Reports funded by National Institutes of Health. Washington (DC)2011.
21. Ross AC, Manson JE, Abrams SA, Aloia JF, Brannon PM, Clinton SK, et al. The 2011 report on dietary reference intakes for calcium and vitamin D from the Institute of Medicine: what clinicians need to know. J Clin Endocrinol Metab. 2011;96(1):53-8. Epub 2010/12/02. doi: 10.1210/jc.2010-2704. PubMed PMID: 21118827; PubMed Central PMCID: PMCPMC3046611.

Nevertheless, we fully acknowledge that vitamin D deficiency threshold remain an ongoing controversy. We have expanded the Discussion to address this: "Our study identified a transition point in relationship between serum 25(OH)D level and biological aging, with males exhibiting a threshold at 38.20 nmol/L and females at 62.50 nmol/L. Beyond this transition point, the increase in 25(OH)D level no longer associates with biological aging deceleration. Regarding the threshold of vitamin D deficiency, it remains an ongoing controversy. The Scientific Advisory Committee on Nutrition (2016) recommended threshold at 25 nmol/L among UK population [46], whereas the IOM (2011) suggested thresholds at 30 nmol/L based on evidence in U.S. and Canadian populations [20]. Meanwhile, the European Food Safety Authority 2016 and the Endocrine Society guideline 2011 proposed higher level of 50 nmol/L as deficiency cutoff [47,48]. The discrepancy in cutoff values stems from multiple factors, for example, different regional ultraviolet B exposure patterns, dietary practice and population-specific biological variability. Furthermore, guideline variations also reflect different focus on health outcomes, with some prioritizing musculoskeletal health [49]. In 2024, the Endocrine Society guideline retracted their previous recommendation of threshold at 50 nmol/L due to limited clinical trial evidence [7]. It also highlighted the impracticality of universal thresholds due to heterogeneity in health outcomes and population characteristics. Hence, the interpretation of 25(OH)D level threshold determination in our study should be with caution, especially when it derived from observational study in an ethnically diverse population. Nevertheless, the trend of benefits attenuation we observed is worth noting, suggesting excessive level of serum 25(OH)D may not be beneficial regarding biological aging process." **(Page 12, line332-352).**

The following reference was supplemented in the References List:

46. England PH. SACN vitamin D and health report 2016 [updated 2025-04-22]. Available from: https://www.gov.uk/government/publications/sacn-vitamin-d-and-health-report.
47. Turck D, Bresson J, Burlingame B. Scientific opinion on dietary reference values for vitamin D EFSA Panel on Dietetic Products, Nutrition, and Allergies (NDA). EFSA J. 2016;179.
48. Holick MF, Binkley NC, Bischoff-Ferrari HA, Gordon CM, Hanley DA, Heaney RP, et al. Evaluation, treatment, and prevention of vitamin D deficiency: an Endocrine Society clinical practice guideline. J Clin Endocrinol Metab. 2011;96(7):1911-30. Epub 2011/06/08. doi: 10.1210/jc.2011-0385. PubMed PMID: 21646368.
49. Roth DE, Abrams SA, Aloia J, Bergeron G, Bourassa MW, Brown KH, et al. Global

prevalence and disease burden of vitamin D deficiency: a roadmap for action in low- and middle-income countries. Ann N Y Acad Sci. 2018;1430(1):44-79. Epub 2018/09/19. doi: 10.1111/nyas.13968. PubMed PMID: 30225965; PubMed Central PMCID: PMCPMC7309365.

8. It is, however, interesting that higher levels are less beneficial in terms of biological age. This is not in line with the recommendations from organisations associated with the industry for supplementing vitamin D, aiming to increase vitamin D levels in the population.

Response: Thank you for your comments. Our findings indicate that high levels of vitamin D are not associated with reduced aging; in fact, they may be linked to accelerated aging in males. While this is an intriguing observation, we cannot conclude that high vitamin D levels promote aging based on this cross-sectional study. Currently, we can only suggest a possible association, and further research is needed to clarify these results.

<u>Reviewer #6:</u> In this manuscript by Feng et al, the authors aimed to explore the link between blood vitamin D levels and physiological aging, using data from over 13,000 U.S. adults in NHANES (2007–2016). The physiological aging was estimated using the PhenoAge model. The results revealed a nonlinear relationship: lower vitamin D levels were generally associated with accelerated aging, especially in females, while excessively high levels were linked to faster aging in males. These patterns suggest that both deficiency and excess of vitamin D may impact aging differently by gender. This revised manuscript has addressed most of the concerns raised by the Editor and Reviewer. However, there are a number of points that still need to be clarified. Specific comments are as follows:

1. The authors mentioned in the methods that CRP numbers were missing in some NHANES data and that PhenoAge (excluding CRP) was used. It would be helpful to briefly comment on how omitting CRP might affect comparability with studies using the standard PhenoAge metric.

Response: We thank the reviewer for your constructive suggestion. For the current analysis, we used 5 cycles to achieve adequate sample size, 3 of which were absent of CRP data. We conducted additional correlation analyses between 2 PhenoAge estimates with and without CRP values. We found a high correlation of 0.986, suggesting that the exclusion of CRP did not significantly alter the overall estimates of PhenoAge. Additionally, this practice of omitting CRP was referenced form previous researches using the same NHANES datasets [3, 4].

We supplemented the following sentences in the **Methods**: "In this study, we utilized PhenoAge as the indicator of biological aging, considering its high robustness and feasibility in NHANES data as a reliable predictor of mortality and age-related disease risks. The PhenoAge algorithm is constructed using NHANES III (1988–1994) by elastic-net regression, encompasses 10 markers: albumin, creatinine, glucose, white blood cell count, lymphocyte percent, red cell distribution width, mean red cell volume,

C-reactive protein, alkaline phosphatase and chronological age [22]. The R package BioAge was used to compute PhenoAge [16]. PhenoAge was both developed and validated within NHANES populations. Meanwhile, all constituent biomarkers are routinely measured in NHANES with standardized protocols, ensuring reproducibility across study cycles. One of the markers, CRP, was unavailable from 2011 to 2018. Previous studies have applied an algorithm that excluded CRP to calculate PhenoAge, demonstrating good applicability [23, 24]. To assess the validity of using PhenoAge without CRP data, we calculated 2 PhenoAge estimates with and without C-reactive protein, using data from cycles 2007-2008 and 2009-2010, which contained all 10 markers. We found a strong correlation between 2 PhenoAge estimates with and without CRP (correlation coefficient of 0.986). Based on these findings, we proceeded with the main analysis using the PhenoAge algorithm excluding CRP, employing data from five NHANES cycles.”. **(Page5-6, line 118-140).**

Additionally, we acknowledge that this is a limitation of the current study and have explicitly acknowledged it in the **Discussion**: “Secondly, the PhenoAge algorithm we applied did not contain CRP data. This could be a limitation given that CRP is a key marker of inflammation and is potentially closely associated with vitamin D status. The absence of CRP in the PhenoAge calculation might lead to underestimation of the true relationship between vitamin D status and age acceleration. To gain a more accurate understanding of this association, future studies with more comprehensive datasets should be conducted.”. **(Page 14, line371-381).**

The following reference was supplemented in the References List:

23. Liu W, Wang J, Wang M, Hou H, Ding X, Ma L, et al. Oxidative Stress Factors Mediate the Association Between Life's Essential 8 and Accelerated Phenotypic Aging: NHANES 2005-2018. J Gerontol A Biol Sci Med Sci. 2024;79(1). Epub 2023/10/10. doi: 10.1093/gerona/glad240. PubMed PMID: 37813096.

24. He H, Chen X, Ding Y, Chen X, He X. Composite dietary antioxidant index associated with delayed biological aging: a population-based study. Aging (Albany NY). 2024;16(1):15-27. Epub 2024/01/04. doi: 10.18632/aging.205232. PubMed PMID: 38170244; PubMed Central PMCID: PMCPMC10817368.

2. In Line 220, the authors cite a p-value (p = 0.076) to describe the absence of dose-dependent trends in both the whole population and females. It is suggested to clarify this sentence to correctly distinguish between the groups with the right numbers.

Response: thank you for your careful reading. We have corrected this mistake to make it clear.

3. In the Discussion section (line 323), the authors state that serum 25(OH)D levels above 125 nmol/L in female participants were linked to accelerated physiological aging. However, this interpretation does not appear to be supported by the data in Table 3. Revising this statement to more accurately reflect the results is recommended.

Response: thank you very much for your comments. We have revised as “Our results

found the 25(OH)D levels over 91.5 nmol/L in male participants were not linked to accelerated biological aging, approximately consistent with those reports."

Reference

1. . Ross AC, Taylor CL, Yaktine AL, Del Valle HB, editors. Washington (DC)2011.
2. McCartney CR, McDonnell ME, Corrigan MD, Lash RW. Vitamin D Insufficiency and Epistemic Humility: An Endocrine Society Guideline Communication. J Clin Endocrinol Metab. 2024;109(8):1948-54. Epub 2024/06/03. doi: 10.1210/clinem/dgae322. PubMed PMID: 38828961.
3. He H, Chen X, Ding Y, Chen X, He X. Composite dietary antioxidant index associated with delayed biological aging: a population-based study. Aging (Albany NY). 2024;16(1):15-27. Epub 2024/01/04. doi: 10.18632/aging.205232. PubMed PMID: 38170244; PubMed Central PMCID: PMCPMC10817368.
4. Liu W, Wang J, Wang M, Hou H, Ding X, Ma L, et al. Oxidative Stress Factors Mediate the Association Between Life's Essential 8 and Accelerated Phenotypic Aging: NHANES 2005-2018. J Gerontol A Biol Sci Med Sci. 2024;79(1). Epub 2023/10/10. doi: 10.1093/gerona/glad240. PubMed PMID: 37813096.
5. Dan YL, Yang YQ, Zhu DC, Bo L, Lei SF. Accelerated biological aging as a potential risk factor for rheumatoid arthritis. Int J Rheum Dis. 2024;27(4):e15156. Epub 2024/04/26. doi: 10.1111/1756-185X.15156. PubMed PMID: 38665050.
6. Jain P, Binder A, Chen B, Parada H, Jr., Gallo LC, Alcaraz J, et al. The Association of Epigenetic Age Acceleration and Multimorbidity at Age 90 in the Women's Health Initiative. J Gerontol A Biol Sci Med Sci. 2023;78(12):2274-81. Epub 2022/09/16. doi: 10.1093/gerona/glac190. PubMed PMID: 36107798; PubMed Central PMCID: PMCPMC10692424.
7. Daraghmeh AH, Bertoia ML, Al-Qadi MO, Abdulbaki AM, Roberts MB, Eaton CB. Evidence for the vitamin D hypothesis: The NHANES III extended mortality follow-up. Atherosclerosis. 2016;255:96-101. Epub 2016/11/18. doi: 10.1016/j.atherosclerosis.2016.04.007. PubMed PMID: 27855294.
8. Ma J, Li K. Negative Association between Serum Vitamin D Levels and Depression in a Young Adult US Population: A Cross-Sectional Study of NHANES 2007-2018. Nutrients. 2023;15(13). Epub 2023/07/14. doi: 10.3390/nu15132947. PubMed PMID: 37447273; PubMed Central PMCID: PMCPMC10346331.
9. Liu C, Hua L, Xin Z. Synergistic impact of 25-hydroxyvitamin D concentrations and physical activity on delaying aging. Redox biology. 2024;73:103188. Epub 2024/05/14. doi: 10.1016/j.redox.2024.103188. PubMed PMID: 38740004; PubMed Central PMCID: PMCPMC11103937.
10. Fang A, Zhao Y, Yang P, Zhang X, Giovannucci EL. Vitamin D and human health: evidence from Mendelian randomization studies. Eur J Epidemiol. 2024;39(5):467-90. Epub 2024/01/12. doi: 10.1007/s10654-023-01075-4. PubMed PMID: 38214845.
