# Supplementary material for: Association of 25(OH)D serum level with biological aging: A Cross-Sectional Study of 2007–2016 NHANES surveys
Source: PLoS One. 2025 Aug 6;20(8):e0328107. doi: 10.1371/journal.pone.0328107 (PMC12327616; doi:10.1371/journal.pone.0328107)
Supplement: Attachment — Submitted filename: PONE-D-24-25180_R3_Mickael Essouma comments and edits.pdf [file pone.0328107.s010.pdf]

PLOS ONE

Association of 25(OH)D serum level with biological aging: A Cross-Sectional Study of 2007-2016 NHANES surveys

--Manuscript Draft--

**Manuscript Number:**	PONE-D-24-25180R3
**Article Type:**	Research Article
**Full Title:**	Association of 25(OH)D serum level with biological aging: A Cross-Sectional Study of 2007-2016 NHANES surveys
**Short Title:**	aging and 25(OH)D serum level
**Corresponding Author:**	Jue Wang<br>nanhui new town community health service center<br>shanghai, CHINA
**Keywords:**	vitamin D; Serum 25(OH)D; Biological Aging; PhenoAge; nonlinear association
**Abstract:**	Vitamin D plays a crucial role in various aspects of human body, including aging, but chronological age may not accurately reflect the true biological aging status. Recently, PhenoAge has been developed to estimate an individual's biological age based on different biological and clinical measures. Therefore, we investigated the relationship between 25(OH)D serum levels and biological aging calculated by PhenoAge Acceleration (PhenoAgeAccel) using data from the 2007-2016 National Health and Nutrition Examination Survey (NHANES). 25(OH)D serum levels showed a negative association with PhenoAgeAccel (β=-0.04; 95% confidence interval [CI]: -0.08 to 0.00). This negative association was dose-dependent in females (β=-0.07; 95% CI: -0.12 to 0.01), but not in males. Generalized additive models further found 25(OH)D serum levels were linked to negative PhenoAgeAccel at levels below 38.2 nmol/L (15.3 ng/mL) in males (β=-0.013; 95% CI: -0.025 to -0.002) and 62.5 nmol/L (25.0 ng/mL) in females (β=-0.007; 95% CI: -0.01 to -0.004,), revealing a U-shaped relationship observed in males but an L-shaped pattern in females. . However, 25(OH)D serum levels above 125 nmol/L were not associated with PhenoAgeAccel in females (β=-0.001; 95% CI: -0.005-0.002), while in males, elevated levels (>91.55 nmol/L) were associated with positive PhenoAgeAccel (β=0.005; 95% CI: 0.002-0.008). Our findings indicate that vitamin D insufficiency has an inverse link with accelerated biological aging, and high levels of vitamin D in males accelerated biological aging as well, offering valuable insights into the relationship between vitamin D and biological aging.
**Order of Authors:**	Yi Feng
	Licheng Yu
	Jue Wang
**Opposed Reviewers:**	
**Response to Reviewers:**	Dear editor Mickael Essouma and reviewers<br>We sincerely thank the reviewers for their approval of our manuscript and the editor for the valuable and constructive suggestions, especially the meticulous word-for-word revisions. We have carefully studied all comments and incorporated them thoroughly into our revisions. We are very grateful for the editor's dedicated support and hope the revised manuscript is now satisfactory. Should you have any questions, please feel free to contact us. We appreciate your continued consideration.<br><br>1. Consider addressing the points 2, 3, 4 and 5 highlighted in the section "Additional Editor comments" of the Decision letter about manuscript R1 when discussing the study limitations in the discussion section.<br>Response：The limitations have been revised in the manuscript, and the concerns raised in the "Additional Editor comments," including the vitamin D classification and race definition, have been addressed. Thank you so much.<br>2. The first major reporting issue is the lack of clarity about the metrics of biological aging studied. Was it PhenoAge alone, PhenoAge and PhenoAgeAccel, or PhenoAgeAccel alone? This issue stems from the fact that the term PhenoAge is inconsistently used throughout the manuscript. In the same vein, it seems to me that

developers of PhenoAge termed it "DNAm PhenoAge". So, even if many authors keep using the term "PhenoAge" rather than "DNAm PhenoAge", I would use the term "DNAm PhenoAge" and consequently also the term "DNAm PhenoAgeAccel" when talking about PHenoAge Acceleration throughout the manuscript. The choice of the term to use between "DNAm PhenoAge" and "PhenoAge" (and consequently also between "DNAm PhenoAgeAccel" and "PhenoAgeAccel") is up to you, the only requeirement in this regard is consistency with the term chosen.
Response： We have reviewed the entire manuscript and made corrections to use either "PhenoAge" or "PhenoAgeAccel" accordingly.

3. A second major issue is that the authors do not use the PhenoAge classification (positive versus negative) when reporting on the association between 25(OH)D serum levels and PhenoAgeAccel throughout the manuscript, although they clearly mentioned that classification in the Materials and Methods section. Rather, they kept using the expression "reduced PhenoAgeAccel" which would need to be replaced by "negative PhenoAgeAccel" or perhaps "Decreasing PhenoAGeAccel". Therefore, consider implementing the recognised PhenoAgeAccel classification in the results and discussion sections of the manuscript.
Response：A positive value of PhenoAgeAccel indicates an accelerated biological aging process, while a negative value indicates a slower biological aging process. We have revised "reduced/increased PhenoAgeAccel" to "negative/positive PhenoAgeAccel".
4. A third major issue (linked to the other major study variable: 25(OH)D serum level) is that the authors sticked to the 2011 IOM classification of 25(OH)D serum levels which includes four vitamin D serum level categories (sufficiency, insufficiency, deficiency and equal to or above 75 nmol/L) meanwhile they were advised to drop that classification given that the Endocrine Society no longer endorses that vitamin D classification status. I get it that the authors have the right to choose the classification system that best suits their needs. However, when doing so, they need to acknowledge in the limitations statement of the discussion section that the classification system used is now outdated and few evidence if any, currently to supports that classification system.
Response： We have added this information in the section of limitation. “the classification of 25(OH)D serum levels used in our study is based on the 2011 IOM guidelines; however, more recent classifications, such as those from the Scientific Advisory Committee on Nutrition (2016), are considered more accurate. It would be preferable to analyze the association between vitamin D status and biological aging using these updated classifications”. Thank you very much.

5. In addition, 25(OH)D serum levels need to be expressed using either or both of the two units ng/mL and/or nmol/L, but the unit chosen should be consistently used throughout the manuscript. I advise against using the term "correlation" throughout the manuscript, given the lack of correlation analyses in the manuscript. More comments and suggested edits are available in the document PONE-D-24-25180_R2_Mickael Essouma comments and suggested edits.pdf attached to this decision letter.
Response：25(OH)D serum levels were expressed using nmol/L and we correct these through the whole manuscript.
In the revised manuscript, the term “correlation” has been replaced with “association”. We have carefully reviewed all the comments in the document and accepted the suggested revisions. These suggestions have been very helpful in improving our manuscript. We are very grateful for the editor's support and revision. Thank you very much.

Additional Information:	
**Question**	**Response**
**Financial Disclosure**<br><br>Enter a financial disclosure statement that describes the sources of funding for the work included in this submission. Review the submission guidelines for detailed	The author(s) received no specific funding for this work.

requirements. View published research articles from *PLOS ONE* for specific examples.<br><br>This statement is required for submission and **will appear in the published article** if the submission is accepted. Please make sure it is accurate.<br><br>**Funded studies**<br>Enter a statement with the following details:<br>• Initials of the authors who received each award<br>• Grant numbers awarded to each author<br>• The full name of each funder<br>• URL of each funder website<br>• Did the sponsors or funders play any role in the study design, data collection and analysis, decision to publish, or preparation of the manuscript?<br><br>Did you receive funding for this work?	
**Competing Interests**<br><br>Use the instructions below to enter a competing interest statement for this submission. On behalf of all authors, disclose any competing interests that could be perceived to bias this work—acknowledging all financial support and any other relevant financial or non-financial competing interests.<br><br>This statement is required for submission and **will appear in the published article** if the submission is accepted. Please make sure it is accurate and that any funding sources listed in your Funding Information later in the submission form are also declared in your Financial Disclosure statement.<br><br>View published research articles from *PLOS ONE* for specific examples.	The authors have declared that no competing interests exist.

**NO authors have competing interests**<br><br>Enter: *The authors have declared that no competing interests exist.*<br><br>**Authors with competing interests**<br><br>Enter competing interest details beginning with this statement:<br><br>*I have read the journal's policy and the authors of this manuscript have the following competing interests: [insert competing interests here]*<br><br>* typeset	
**Ethics Statement**<br><br>Enter an ethics statement for this submission. This statement is required if the study involved:<br><br>• Human participants<br>• Human specimens or tissue<br>• Vertebrate animals or cephalopods<br>• Vertebrate embryos or tissues<br>• Field research<br><br>Write "N/A" if the submission does not require an ethics statement.<br><br>General guidance is provided below. Consult the submission guidelines for detailed instructions. **Make sure that all information entered here is included in the Methods section of the manuscript.**	the study involves human participants. all data from NHANES, which is a publicly available dataset managed by the Centers for Disease Control and Prevention. The ethical approval of NHANES has been stated on the website. (https://wwwn.cdc.gov/nchs/nhanes/Default.aspx)

**Format for specific study types**<br><br>**Human Subject Research (involving human participants and/or tissue)**<br>• Give the name of the institutional review board or ethics committee that approved the study<br>• Include the approval number and/or a statement indicating approval of this research<br>• Indicate the form of consent obtained (written/oral) or the reason that consent was not obtained (e.g. the data were analyzed anonymously)<br><br>**Animal Research (involving vertebrate animals, embryos or tissues)**<br>• Provide the name of the Institutional Animal Care and Use Committee (IACUC) or other relevant ethics board that reviewed the study protocol, and indicate whether they approved this research or granted a formal waiver of ethical approval<br>• Include an approval number if one was obtained<br>• If the study involved *non-human primates*, add *additional details* about animal welfare and steps taken to ameliorate suffering<br>• If anesthesia, euthanasia, or any kind of animal sacrifice is part of the study, include briefly which substances and/or methods were applied<br><br>**Field Research**<br><br>Include the following details if this study involves the collection of plant, animal, or other materials from a natural setting:<br>• Field permit number<br>• Name of the institution or relevant body that granted permission	
**Data Availability**<br><br>Authors are required to make all data underlying the findings described fully available, without restriction, and from the time of publication. PLOS allows rare exceptions to address legal and ethical concerns. See the PLOS Data Policy and FAQ for detailed information.	Yes - all data are fully available without restriction

Powered by Editorial Manager® and ProduXion Manager® from Aries Systems Corporation

A Data Availability Statement describing where the data can be found is required at submission. Your answers to this question constitute the Data Availability Statement and **will be published in the article**, if accepted.<br><br>**Important:** Stating 'data available on request from the author' is not sufficient. If your data are only available upon request, select 'No' for the first question and explain your exceptional situation in the text box.<br><br>Do the authors confirm that all data underlying the findings described in their manuscript are fully available without restriction?	
**Describe where the data may be found in full sentences. If you are copying our sample text, replace any instances of XXX with the appropriate details.**<br><br>• If the data are **held or will be held in a public repository**, include URLs, accession numbers or DOIs. If this information will only be available after acceptance, indicate this by ticking the box below. For example: *All XXX files are available from the XXX database (accession number(s) XXX, XXX.*).<br>• If the data are all contained **within the manuscript and/or Supporting Information files**, enter the following: *All relevant data are within the manuscript and its Supporting Information files.*<br>• If neither of these applies but you are able to provide **details of access elsewhere**, with or without limitations, please do so. For example:<br><br>*Data cannot be shared publicly because of [XXX]. Data are available from the XXX Institutional Data Access / Ethics Committee (contact via XXX) for researchers who meet the criteria for access to confidential data.*<br><br>*The data underlying the results presented in the study are available from (include the name of the third party*	All files are available from the NHANES database

and contact information or URL).<br>• This text is appropriate if the data are owned by a third party and authors do not have permission to share the data.<br><br>* typeset	
Additional data availability information:	

Dear editor Mickael Essouma and reviewers

We sincerely thank the reviewers for their approval of our manuscript and the editor for the valuable and constructive suggestions, especially the meticulous word-for-word revisions. We have carefully studied all comments and incorporated them thoroughly into our revisions. We are very grateful for the editor's dedicated support and hope the revised manuscript is now satisfactory. Should you have any questions, please feel free to contact us. We appreciate your continued consideration.

Yours sincerely

Corresponding author: Jue Wang

JUN, 2025

Manuscript

Click here to access/download;Manuscript;revised manuscript 20250612 clean.docx

Association of 25(OH)D serum level with biological aging: A Cross-Sectional Study of 2007-2016 NHANES surveys

Yi Feng[a]; Licheng Yu[b]; Jue Wang[a] *

[a] Nanhui new town Community Health Service Center, Pudong New Area, Shanghai, 201308, China.

[b] Prevention and Health Care Department, Heqing Community Health Service Center of Pudong New District, Shanghai, 201201, China.

Corresponding author. Jue Wang

E-mail address: wangjue180130@126.com

Abstract

Vitamin D plays a crucial role in various aspects of human body, including aging, but chronological age may not accurately reflect the true biological aging status. Recently, PhenoAge has been developed to estimate an individual's biological age based on different biological and clinical measures. Therefore, we investigated the relationship between 25(OH)D serum levels and biological aging calculated by PhenoAge Acceleration (PhenoAgeAccel) using data from the 2007-2016 National Health and Nutrition Examination Survey (NHANES). 25(OH)D serum levels showed a negative association with PhenoAgeAccel (β=-0.04; 95% confidence interval [CI]: -0.08 to 0.00). This negative association was dose-dependent in females (β=-0.07; 95% CI: -0.12 to 0.01), but not in males. Generalized additive models further found 25(OH)D serum levels were linked to negative PhenoAgeAccel at levels below 38.2 nmol/L (15.3 ng/mL) in males (β=-0.013; 95% CI: -0.025 to -0.002) and 62.5 nmol/L (25.0 ng/mL) in females (β=-0.007; 95% CI: -0.01 to -0.004,), revealing a U-shaped relationship observed in males but an L-shaped pattern in females. However, 25(OH)D serum levels above 125 nmol/L were not associated with PhenoAgeAccel in females (β=-0.001; 95% CI: -0.005-0.002), while in males, elevated levels (>91.55 nmol/L) were associated with positive PhenoAgeAccel (β=0.005; 95% CI: 0.002-0.008). Our findings indicate that vitamin D insufficiency has an inverse link with accelerated biological aging, and high levels of vitamin D in males accelerated biological aging as well, offering valuable insights into the relationship between vitamin D and biological aging.

Key words: Vitamin D; Serum 25(OH)D; Biological aging; PhenoAge; Nonlinear association

1. Introduction

Vitamin D is a fat-soluble vitamin that plays a crucial role in various aspects of human health, with its effects extending to bone metabolism, immune function, etc. It is acquired through two primary sources: endogenous production (mainly vitamin D3) in the skin upon exposure to ultraviolet B (UVB) radiations, and exogenous intake (vitamin D2 and D3) through dietary sources, such as fatty fish, egg yolks or supplements. Once absorbed in the body, vitamin D is converted into 25-hydroxyvitamin D (25(OH)D) in the liver, then transformed into the biologically active form, 1α,25-dihydroxyvitamin D (1α,25$(OH)_2$D), in the kidneys [1]. This active form binds to the vitamin D receptor (VDR), regulating calcium and phosphate metabolism [2]. Serum 25(OH)D is the most commonly measured form of vitamin D in humans because it is the major circulating form and provides an accurate indication of the body's overall vitamin D status [3]; and it can be influenced by various factors including the geographic location, race and food intake [4].

Vitamin D deficiency is a global public health concern, associated with increased risk of type 2 diabetes (T2D), hypertension, multiple sclerosis [5-7]. The prevalence of vitamin D deficiency is particularly high among the elderly [8], with some studies suggesting that it may accelerate chronological aging and contribute to age-related diseases and inflammatory diseases, such as cardiovascular disease, multiple sclerosis and rheumatoid arthritis [9]. However, several large randomized clinical trials of vitamin D supplementation failed to demonstrate significant benefits on cardiovascular disease, casting doubt on the evidence from observational studies linking vitamin D deficiency with cardiovascular disease [10, 11]. This ambiguity highlights challenges in assessing the association between vitamin D and aging. A major limitation of previous studies is that they relied on chronological age which is quite discrepant with biological age. Indeed, unlike chronological age which is a precise reflection of an individual's lifetime, biomarkers making up the biological age are mainly determined by a combination of genetic, environmental and lifestyle factors [12]. Accordingly, some individuals may age biologically faster or slower than indicated by their

chronological age.

To better characterize physiological aging in epidemiological studies, several aging metrics based on the combination of clinical (e.g., PhenoAge and GrimAge) and biological (PhenoAge acceleration) markers have been developed [13]. In a post hoc analysis of the European DO-HEALTH randomized clinical trial involving 777 participants with a mean 25(OH)D serum level of 56.75 nmol/L, PhenoAge was the only biological age metric shown to be slowed by daily vitamin D (2000 UI) supplementation among the four biological age metrics that were assessed: PhenoAge, GrimAge, GrimAge2 and DunedinPACE [14]. PhenoAgeAccel was developed and validated using machine learning methods on a National Health and Nutrition Examination Survey (NHANES) dataset of 4000 adults aged 20 to 84 years and it showed a mean absolute error of approximately 3.6 years when predicting the chronological age [15]. Although vitamin D supplementation was shown to slow the PhenoAgeAccel value in older adults with low 25(OH)D serum concentrations, observational studies linking 25(OH)D serum concentrations with PhenoAgeAccel are lacking. Therefore, in this study, we assessed whether there is a linear and/or non-linear relationship between 25(OH)D serum concentration and PhenoAgeAccel using datasets of 2007-2016 NHANES surveys. By exploring this relationship, the study aims to enhance our understanding of the link between serum vitamin D levels and biological aging in human beings for the benefit of public health.

~~2.~~ Materials and Methods

~~2.1~~ Study design and population

This study was conducted and reported in agreement with STROBE guidelines for reporting observational studies. This study utilized data derived from the 2007-2016 NHANES conducted in the United States of America by the National Center for Health Statistics of the Centers for Disease Control and Prevention. All data included in the current study are publicly available on the NHANES website [16]. NHANES includes in-home interview, physical measurement and laboratory tests. The 2007-2016

NHANES datasets involved 50558 adult participants. participants with incomplete information and participants aged less than 40 years old were excluded. After excluding, a total of 13,910 participants were finally included in this analysis (Figure 1).

2.2 Measurement of 25(OH)D serum level

Vitamin D serum levels were reflected by 25(OH)D. Because the forms of 25(OH)D measured in the 2007-2016 NHANES samples included both 25(OH)D2 and 25(OH)D3 (classic 25(OH)D3 and C3 epimer of 25(OH)D3 [C3-epi-25(OH)D3]), serum 25(OH)D included 25(OH)D2, 25(OH)D3 and C3-epi-25(OH)D3 in this study. During the study period (2007-2016), 25(OH)D levels were measured in the NHANES population using the liquid chromatography-tandem mass spectrometry (LC-MS/MS). According to the 2011 Institute of Medicine's Committee recommendations, we transformed serum 25(OH)D concentrations into a categorical variable with the following modalities: deficiency (<30 nmol/L), insufficiency (30–50 nmol/L), sufficiency (50–75 nmol/L) and abnormally high (≥75 nmol/L)[17].

2.3 Measurement of PhenoAge

In this study, we utilized PhenoAge as the indicator of biological aging, considering its high robustness and feasibility in NHANES data as a reliable predictor of mortality and age-related disease risks. The PhenoAgeAccel algorithm was constructed by applying elastic-net regression methods to NHANES III (1988-1994) datasets, encompasses 10 markers: serum albumin, serum creatinine, plasma glucose, white blood cell count, blood lymphocyte percent, red cell distribution width, mean red cell volume, serum C-reactive protein, serum alkaline phosphatase and chronological age [18]. All constituent biomarkers of PhenoAge were routinely measured during NHANES using standardized protocols ensuring reproducibility across study cycles. One of the markers, CRP, was unavailable from 2011 to 2018 datasets. Previous studies have applied an algorithm that excluded CRP to calculate PhenoAge, demonstrating good applicability [19, 20]. To assess the validity of using PhenoAge without CRP data,

we calculated 2 PhenoAge estimates with and without C-reactive protein, using data from cycles 2007-2008 and 2009-2010, which contained all 10 markers. We found a strong association between 2 PhenoAge estimates with and without CRP (Pearson's correlation coefficient of 0.986). Based on this finding, we proceeded with the main analysis of data from the five NHANES cycles using the PhenoAge algorithm excluding CRP. We further calculated PhenoAgeAccel as the difference between PhenoAge and chronological age. A positive value of PhenoAgeAccel represents accelerated biological aging process, while a negative value represented slower biological aging process. Subsequently, PhenoAgeAccel was standardized to have a mean of 0 and a standard deviation (SD) of 1.

~~2.4~~ Covariates

NHANES interviews were conducted by trained interviewers. Questionnaires were administered to participants to collect information on demographics (chronological age, gender, race, education level, poverty income ratio (PIR) and lifestyle-related factors (tobacco smoking, alcohol consumption, physical activity, and comorbidities)~~. The~~ body mass index (BMI) ~~were~~ measured at the Mobile Examination Center.

Covariates in regression analyses testing the association between 25(OH)D serum levels and biological aging include age, race, education level, PIR, BMI, tobacco smoking, alcohol consumption, physical activity. The variable active tobacco smoking was classified into three categories: never smokers (previous smoking of a total of less than 100 cigarettes), current smokers (lifetime smoking of over 100 cigarettes) and former smokers (had smoked ⩾100 cigarettes in their lifetime but had quit smoking at the time of the survey). The variable alcohol consumption was classified as never drinkers (participants who had drunk <12 drinks in their lifetime), current drinkers (participants who had drunk ⩾12 drinks in their lifetime and at least one drink in the past year), and former drinkers (participants who had drunk ⩾12 drinks in their lifetime but no drink in the past year). A Metabolic Equivalent task (MET)-min per

week were calculated to assess physical activity, levels (in min per week) that were categorized as: inactive (no regular physical activity), insufficient (<500 MET-min activity/week), moderate (500-1,000 MET-min activity/week) and high (⩾1,000 MET-min activity/week) [21]. For sleep duration, participants responded to the question: "How much sleep do you usually get at night on weekdays or on workdays?" Sleep duration was categorized into 3 groups: <7 h, 7-8 h and ⩾8h). The definition of hypertension was based on self-reports, the use of antihypertensive drugs or on the record of a systolic blood pressure⩾140 mmHg with or without a diastolic blood pressure⩾90 mmHg on physical exam. The definition of diabetes mellitus was based on self-reports or on laboratory test results showing fasting blood glucose levels ⩾126 mg/dL or HbA1c values ⩾6.5%.

2.5 Statistical analysis

Weighted multivariable linear regression models were constructed to estimate the association between serum 25(OH)D status and PhenoAgeAccel. 25(OH)D serum levels were log-transformed due to their skewed distribution. To evaluate whether the association between 25(OH)D serum levels and PhenoAgeAccel was not just linear but also dose-dependent, 25(OH)D serum level was analyzed as both a continuous (log-transformed) and a categorical variable. In agreement with standards for linear regression analyses involving continuous dependent variables, the association between 25(OH)D serum levels as continuous variables and PhenoAgeAccel was estimated based on beta coefficients with their 95% confidence intervals [22]. All models were adjusted for chronological age, gender, race, education level, PIR, BMI, tobacco smoking and alcohol consumption variable, physical activity, sleep duration, hypertension, diabetes and season of measurement.

To explore whether there is also a non-linear relationship between 25(OH)D serum levels and PhenoAgeAccel, a Generalized Additive Model (GAM) was employed to fit

the smooth curve. The GAMs extend traditional linear regression by replacing linear terms with smooth functions, allowing flexible modeling of complex dose-response relationships without assuming a priori parametric forms [23]. The threshold effect was further estimated using segmented regression model. This method identifies inflection points where the slope of the association between 25(OH)D serum levels and PhenoAgeAccel changes significantly. The optimal breakpoint was determined via likelihood ratio tests, comparing the goodness-of-fit between the non-segmented linear model and the segmented regression model [24]. Results ~~of~~ *P*-value ~~of~~ <0.05 were considered to be statistically significance . All data analyses were performed using R software version 4.2.1 (R Core Team, Vienna, Austria). R package ‘BioAge’ was used to calculate PhenoAge [15], while the package ‘mgcv’ was used to conduct GAM analysis [25]. Continuous data were reported as means ± standard deviations (SDs) for normal distributions or as medians (interquartile ranges) for skewed distributions. Categorical data were reported as numbers (percentages).

~~2.6~~ Ethical considerations

This study utilized data from NHANES study, which was conducted in accordance with the principles in the Declaration of Helsinki. The NHANES protocol received prior ethical approval from the National Center for Health Statistics' Review Board. Since our study is a secondary analysis of publicly accessible, de-identified NHANES data sets, additional institutional review board approval was not required [26].

~~3.~~ Results

~~3.1~~ Characteristics of the study population

The current study included a total of 6836 males and 7074 females, with the characteristics of the study population presented in Table 1. There are significant differences between males and females regarding the distributions of age, race, BMI, and poverty-income ratio ($p < 0.001$). Additionally, gender-specific differences were noted in terms of behaviors influencing biological aging, such as smoking, drinking,

physical activity, and sleep duration ($p < 0.001$). Notably, males had a higher prevalence of diabetes mellitus than females. The median 25(OH)D serum level in females was 66.6 nmol/L compared with 63.8 nmol/L in males ($p<0.001$). The PhenoAgeAccel of males and females were -2.40 (-5.13, 0.94) and -4.14 (-7.22, -0.46) respectively, suggesting that PhenoAgeAccel was lower in females than in males.

3.2 Association between 25(OH)D serum level and biological aging

The associations between 25(OH)D serum levels and PhenoAgeAccel were presented in Table 2. After adjusting for age, race, education level, PIR, BMI, smoking, alcohol consumption, level of physical, sleep duration and presence of hypertension and/or diabetes mellitus, 25(OH)D serum levels were negatively associated with PhenoAgeAccel (β=-0.04 SD; 95% CI: -0.08 to 0.00). Segregating this data by sex, the association was statistically significant only in females ($p=0.007$). When 25(OH)D serum levels were subdivided into quartiles, 25(OH)D serum levels were inversely associated with PhenoAgeAccel, with no apparent dose-dependent trend (p for trend =0.173). When segregating the data by sex, 25(OH)D serum levels were inversely associated with PhenoAgeAccel only in women, with no apparent dose-response relationship (p for trend = 0.076). When categorizing 25(OH)D serum levels as recommended in the 2011 USA institute of Medicine guidelines, 25(OH)D serum levels were inversely associated with PhenoAgeAccel for 25(OH)D serum levels between 30-50 nmol/L and 50-75 nmol/L in males (with no apparent dose-response relationship), and between 50-75 nmol/L and ⩾75 nmol/L in females, with an apparent dose-response relationship ($p=0.014$).

Results of subgroup/effect modification analysis showed that in males high 25(OH)D serum levels were associated with negative PhenoAgeAccel only in people aged over 60 years, those with hypertension and those with diabetes mellitus, suggesting that there were statistically significant interactions between 25(OH)D serum levels and age, hypertension, and diabetes (Fig.2A). In females, we did not find any effect modifier for the association between 25(OH)D serum levels and PhenoAge Accel

(Fig.2B).

3.3 Association of 25(OH)D serum levels with PhenoAgeAccel on GAM

Given the lack of linear associations between 25(OH)D serum levels and PhenoAgeAccel when subdividing 25(OH)D serum levels into quartiles (Table 2), GAM was utilized to investigate the potential linkage between serum 25(OH)D and PhenoAgeAccel. There was a nonlinear relationship between 25(OH)D serum level and PhenoAgeAccel in both males and females (p<0.001) (Fig 3 A-B). In men, the relationship was U-shaped with the two inflection points at 38.2 nmol/L and 91.5 nmol/L and in women, the segmented regression modelling confirmed a L-shaped relationship. Low 25(OH)D serum levels (<38.2 nmol/L) were negatively related to PhenoAgeAccel (β=-0.013 SD; 95% CI: -0.025 to -0.002), whereas high levels (>91.55 nmol/L) showed a positive association (β=0.005 SD; 95% CI: 0.002 to 0.008). For females, 25(OH)D serum levels below 62.5 nmol/L were negatively linked to PhenoAgeAccel (β=-0.007 SD; 95% CI: -0.01 to -0.004), with no significant association observed beyond 125 nmol/L (β=-0.001 SD; 95% CI: -0.005 to -0.002). Overall, low (<38.2 nmol/L) or high (>91.55 nmol/L) 25(OH)D serum levels in males were associated with PhenoAgeAccel, while in females, only low levels (<62.5 nmol/L) was associated with for PhenoAgeAccel (Table 3).

4. Discussion

In the current study, the association between 25(OH)D serum level and biological aging was investigated in the 2007-2016 NHANES surveys. The analysis revealed a negative association between 25(OH)D serum levels and PhenoAge. The effect modifier analysis showed that age above 60 years, diabetes mellitus and hypertension modified the association between 25(OH)D serum levels and PhenoAge in men, but no modifier was found for that association in females. When subdividing 25(OH)D serum levels into quartiles and stratifying data by sex, this association was still observed only in females, but in a non-linear fashion. When further subdividing 25(OH)D serum

levels as recommended by the Institute of Medicine in USA in 2011, the association persisted for 25(OH)D serum levels between 30-50 nmol/L and between 50-75 nmol/L in males and between 50-75 nmol/L and ≥75 nmol/L, with a linear pattern of relationship observed only in females. Non-linear associations were also found between 25(OH)D serum levels and PhenoAgeAccel in both males (U-shaped) and females (L-shaped).

Previous studies have reported that a gradual decline in 25(OH)D serum levels with chronological age, and vitamin D supplementation can mitigate oxidative damage and improve immune function in elder people, suggesting its potential role in slowing age-related physiological decline [27]. This decline is attributed to several factors, including decreased sun exposure, reduced capacity of the skin to synthesize vitamin D, and decreased dietary intake of vitamin D, resulting in vitamin D deficiency in older adults [28]. The synthesis of vitamin D from sun exposure vary in different races, but our study lacks this data of sun exposure. Additionally, we also found that low 25(OH)D serum levels were associated with positive PhenoAgeAccel, suggesting vitamin D may also play a crucial role in the regulation of the aging process. Adequate 25(OH)D levels are essential for maintaining calcium absorption and bone health preventing osteoporosis, a common age-related condition, and regulating calcium homeostasis and bone metabolism [29]. Moreover, vitamin D can interact with antioxidant and anti-inflammatory pathways, such as activating Nrf2 (nuclear factor erythroid 2-related factor 2) and SIRT1 (sirtuin 1) signaling cascades, and suppressing the production of pro-inflammatory cytokines, including interleukin-6 (IL-6) and tumor necrosis factor-alpha (TNF-α), through inhibiting nuclear factor-κB (NF-κB). These actions lead to reduction of inflammation and oxidative stress, both of which are known to contribute to the aging process [29, 30]. Therefore, vitamin D and aging have a mutual influence on each other.

Vitamin D supplementation could be an effective way to reverse or mitigate diseases, including lower blood pressure and improve endothelial function, leading to reduced risk of cardiovascular events, such as heart attacks and strokes, and improved

cardiovascular outcomes [31]. A meta-analysis of 75 randomized controlled trials in 2014 revealed that vitamin D supplementation was associated with a 6% reduction in all-cause mortality [32]. Some studies suggest that the impact of vitamin D supplementation on all-cause mortality may be more pronounced in certain subgroups, such as older adults or individuals with vitamin D deficiency. For example, vitamin D supplementation was associated with a reduced risk of all-cause mortality in trials with a mean baseline 25(OH)D level <75 nmol/L (RR 0.89, 95% CI 0.80-0.99), but not in trials with a mean baseline 25(OH)D level ≥75 nmol/L (RR 1.04, 95% CI 0.97-1.11) [33]. Thus, these evidence highlights the potential of vitamin D supplementation in reducing the risk of several chronic diseases, particularly in elderly people, where serum vitamin D levels tend to decline with age.

Despite its potential benefits, the use of vitamin D supplements has increased markedly, and concerns about excessive intake have emerged. the Vitamin D and Omega-3 Trial (VITAL) study, a randomized trial involving over 25,000 participants, discovered that vitamin D supplementation did not significantly reduce the risk of all-cause mortality or lower risk of fractures among generally healthy midlife and older adults without vitamin D deficiency or osteoporosis [34]. Furthermore, increased intake of vitamin D supplements by the general population and a growing number of high-dose prescriptions without medical monitoring might result in a greater risk of excessive vitamin D toxicity [35]. Our current results indicated that the association of 25(OH)D serum level and biological aging followed a U-shaped curve in males and an L-shaped curve in females, revealing that high serum level of 25(OH)D is not associated with decelerated aging in either males and females, and even associated with accelerated biological aging in males. These detrimental effects may stem from the toxicity of excessive vitamin D. When vitamin D intake is too high, serum levels of its metabolites [25(OH)D and $24,25(OH)_2D$] rise beyond the binding capacity of vitamin D binding protein, leading to an increase in the free active metabolite, $1,25(OH)_2D$. This active form reaches the VDR in the nucleus, constantly promoting gene expression that enhances calcium absorption and bone mobilization [36]. Consequently, this can

result in hypercalcemia or hyperphosphatemia, which in turn suppresses initial parathyroid hormone (iPTH) level [36, 37]. Such disrupting balance of calcium and hormone may contribute to cardiovascular disease, kidney damage, and skin aging. Research reported that the working diagnosis of vitamin D toxicity is diagnosed based on total 25(OH)D concentrations of 80~200nmol/L detected by immunoassay [38]. Our results found the 25(OH)D levels over 91.5 nmol/L in male participants were not linked to accelerated biological aging, approximately consistent with those reports.

Our study identified a transition point in relationship between 25(OH)D serum level and biological aging, with males exhibiting a threshold at 38.20 nmol/L and females at 62.50 nmol/L. Beyond this transition point, the increase in 25(OH)D level no longer associates with biological aging deceleration. Regarding the threshold of vitamin D deficiency, it remains an ongoing controversy. The Scientific Advisory Committee on Nutrition (2016) recommended threshold at 25 nmol/L among UK population [39], whereas the IOM (2011) suggested thresholds at 30 nmol/L based on evidence in U.S. and Canadian populations [17]. Meanwhile, the European Food Safety Authority 2016 and the Endocrine Society guideline 2011 proposed higher level of 50 nmol/L as deficiency cutoff [40, 41]. The discrepancy in cutoff values stems from multiple factors, for example, different regional ultraviolet B exposure patterns, dietary practice and population-specific biological variability. Furthermore, guideline variations also reflect different focus on health outcomes, with some prioritizing musculoskeletal health [42]. In 2024, the Endocrine Society guideline retracted their previous recommendation of threshold at 50 nmol/L due to limited clinical trial evidence [43]. It also highlighted the impracticality of universal thresholds due to heterogeneity in health outcomes and population characteristics. Hence, the interpretation of 25(OH)D level threshold determination in our study should be with caution, especially when it derived from observational study in an ethnically diverse population. Nevertheless, the trend of benefits attenuation we observed is worth noting, suggesting excessive level of serum 25(OH)D may not be beneficial regarding biological aging process.

Our results reported differences of dose-response curve between in male and in

female, which may be attributed to the sex hormones, especially estrogen. As women age, particularly during the menopausal transition and after menopause, their ovaries produce less estrogen, leading to a decline of estrogen levels. This decline contributes to the thinning and wrinkling of the skin, as well as changes in hair texture and growth patterns [44]. Some studies suggest that vitamin D could enhance the activity of estrogen receptors (ER) via interacting with them, potentially amplifying the anti-aging effects of estrogen in the body [45]. Vitamin D and estrogen also have a synergistic effect on bone metabolism, helping to prevent osteoporosis and fractures, especially in postmenopausal women [46]. Additionally, vitamin D regulate estrogen metabolism, leading to higher circulating estrogen levels and subsequently delay aging [45]. Therefore, high level of serum 25(OH)D may not be associated with biological aging in females, but the mechanisms underlying the adverse effects of extremely high levels (>100 nmol/L) in males remain unclear. Further studies are needed to clarify the complex interplay between these two important hormonal systems.

The current study has several limitations. First, as a cross-sectional investigation, it only provides information on the association between serum 25(OH)D levels and biological aging, making it challenging to establish causal relationships. Second, the classification of 25(OH)D serum levels used in our study is based on the 2011 IOM guidelines; however, more recent classifications, such as those from the Scientific Advisory Committee on Nutrition (2016), are considered more accurate. It would be preferable to analyze the association between vitamin D status and biological aging using these updated classifications. While sun exposure and dietary intake are primary sources of vitamin D, this information was not included in our analysis due to limitations in the NHANES dataset. Additionally, race significantly influences serum vitamin D levels, but the definition of race varies across NHANES data collected over different years, which posed a challenge in our study. Finally, emerging evidence questions the traditional view of serum 25(OH)D as a definitive biomarker of vitamin D status, although it remains the most widely accepted marker. For instance, in individuals with diabetes or obesity, the expression of CYP2R1—the enzyme

responsible for vitamin D 25-hydroxylation—is suppressed, leading to decreased 25(OH)D levels despite stable overall vitamin D levels [47, 48].

5. Conclusion

In this study, we investigated the relationship between 25(OH)D serum levels and PhenoAge in NHANES, and found that there was a negative association between PhenoAgeAccel and 25(OH)D serum level, and these associations were nonlinear in both genders, revealing a U-shaped curve for males and an L-shaped curve for females. Low 25(OH)D serum levels shows a potential positively link to PhenoAgeAccel in both genders, but high 25(OH)D serum levels do not associate with decelerated PhenoAge, and even associated with accelerated PhenoAge in male. Our results suggest that the association between vitamin D levels with biological aging is different in men and women.

Authors contributions

Yi Feng: Conceptualization; Methodology; Data curation; Formal analysis; Writing original draft**; Licheng Yu**: Data curation; Formal analysis; **Jue Wang**: Project administration; Supervision; Writing - review &editing.

Conflicts of interest

The authors declare no competing financial or non-financial interests related to this work. NHANES is a publicly available dataset managed by the Centers for Disease Control and Prevention. The authors confirm that there is no affiliation with or involvement from the NHANES program administrators in this study. The funding source had no role in study design, data collection, analysis, or manuscript preparation.

References

1. Bikle DD. Vitamin D metabolism, mechanism of action, and clinical applications. Chem Biol. 2014;21(3):319-29. Epub 2014/02/18. doi: 10.1016/j.chembiol.2013.12.016. PubMed PMID: 24529992; PubMed Central PMCID: PMCPMC3968073.
2. Dominguez LJ, Farruggia M, Veronese N, Barbagallo M. Vitamin D Sources, Metabolism, and Deficiency: Available Compounds and Guidelines for Its Treatment. Metabolites. 2021;11(4). Epub 2021/05/01. doi: 10.3390/metabo11040255. PubMed PMID: 33924215; PubMed Central PMCID: PMCPMC8074587.
3. Couchman L, Moniz CF. Analytical considerations for the biochemical assessment of vitamin D status. Ther Adv Musculoskelet Dis. 2017;9(4):97-104. Epub 2017/04/07. doi: 10.1177/1759720X17692500. PubMed PMID: 28382113; PubMed Central PMCID: PMCPMC5367642.
4. Rahman A, Elmi A. Air pollutants are negatively associated with vitamin D-synthesizing UVB radiation intensity on the ground. Sci Rep. 2021;11(1):21480. Epub 2021/11/04. doi: 10.1038/s41598-021-00980-6. PubMed PMID: 34728744; PubMed Central PMCID: PMCPMC8563978.
5. Zhao S, Qian F, Wan Z, Chen X, Pan A, Liu G. Vitamin D and major chronic diseases. Trends Endocrinol Metab. 2024. Epub 2024/06/02. doi: 10.1016/j.tem.2024.04.018. PubMed PMID: 38824035.
6. Gaksch M, Jorde R, Grimnes G, Joakimsen R, Schirmer H, Wilsgaard T, et al. Vitamin D and mortality: Individual participant data meta-analysis of standardized 25-hydroxyvitamin D in 26916 individuals from a European consortium. Plos One. 2017;12(2):e0170791. Epub 2017/02/17. doi: 10.1371/journal.pone.0170791. PubMed PMID: 28207791; PubMed Central PMCID: PMCPMC5312926.
7. Fang A, Zhao Y, Yang P, Zhang X, Giovannucci EL. Vitamin D and human health: evidence from Mendelian randomization studies. Eur J Epidemiol. 2024;39(5):467-90. Epub 2024/01/12. doi: 10.1007/s10654-023-01075-4. PubMed PMID: 38214845.
8. Cui A, Xiao P, Ma Y, Fan Z, Zhou F, Zheng J, et al. Prevalence, trend, and predictor analyses of vitamin D deficiency in the US population, 2001-2018. Front Nutr. 2022;9:965376. Epub 2022/10/21. doi: 10.3389/fnut.2022.965376. PubMed PMID: 36263304; PubMed Central PMCID: PMCPMC9573946.
9. Berridge MJ. Vitamin D deficiency accelerates ageing and age-related diseases: a novel hypothesis. The Journal of physiology. 2017;595(22):6825-36. Epub 2017/09/28. doi: 10.1113/JP274887. PubMed PMID: 28949008; PubMed Central PMCID: PMCPMC5685827.
10. Zittermann A, Ernst JB, Prokop S, Fuchs U, Dreier J, Kuhn J, et al. Effect of vitamin D on all-cause mortality in heart failure (EVITA): a 3-year randomized clinical trial with 4000 IU vitamin D daily. Eur Heart J. 2017;38(29):2279-86. Epub 2017/05/13. doi: 10.1093/eurheartj/ehx235. PubMed PMID: 28498942.
11. Scragg R, Stewart AW, Waayer D, Lawes CMM, Toop L, Sluyter J, et al. Effect of Monthly High-Dose Vitamin D Supplementation on Cardiovascular Disease in the Vitamin D Assessment Study : A Randomized Clinical Trial. JAMA Cardiol. 2017;2(6):608-16. Epub 2017/04/07. doi: 10.1001/jamacardio.2017.0175. PubMed PMID: 28384800; PubMed Central PMCID: PMCPMC5815022 ICMJE Form of Disclosure of Potential Conflicts of Interest and none were reported.
12. Xia X, Wang Y, Yu Z, Chen J, Han JJ. Assessing the rate of aging to monitor aging itself. Ageing Res Rev. 2021;69:101350. Epub 2021/05/04. doi: 10.1016/j.arr.2021.101350. PubMed PMID: 33940202.
13. Jylhava J, Pedersen NL, Hagg S. Biological Age Predictors. EBioMedicine. 2017;21:29-36. Epub 2017/04/12. doi: 10.1016/j.ebiom.2017.03.046. PubMed PMID: 28396265; PubMed Central PMCID: PMCPMC5514388.

14. Bischoff-Ferrari HA, Gangler S, Wieczorek M, Belsky DW, Ryan J, Kressig RW, et al. Individual and additive effects of vitamin D, omega-3 and exercise on DNA methylation clocks of biological aging in older adults from the DO-HEALTH trial. Nat Aging. 2025;5(3):376-85. Epub 2025/02/04. doi: 10.1038/s43587-024-00793-y. PubMed PMID: 39900648.
15. Kwon D, Belsky DW. A toolkit for quantification of biological age from blood chemistry and organ function test data: BioAge. Geroscience. 2021;43(6):2795-808. Epub 2021/11/03. doi: 10.1007/s11357-021-00480-5. PubMed PMID: 34725754; PubMed Central PMCID: PMCPMC8602613.
16. Centers for Disease Control and Prevention National Health and Nutrition Examination Survey Questionnaires D, and Related Documentation 2020. [cited 2025 March 7]. Available from: https://wwwn.cdc.gov/nchs/nhanes/.
17. Ross AC, Taylor CL, Yaktine AL, Del Valle HB. Dietary Reference Intakes for Calcium and Vitamin D. The National Academies Collection: Reports funded by National Institutes of Health. Washington (DC)2011.
18. Liu Z, Kuo PL, Horvath S, Crimmins E, Ferrucci L, Levine M. A new aging measure captures morbidity and mortality risk across diverse subpopulations from NHANES IV: A cohort study. PLoS Med. 2018;15(12):e1002718. Epub 2019/01/01. doi: 10.1371/journal.pmed.1002718. PubMed PMID: 30596641; PubMed Central PMCID: PMCPMC6312200.
19. Liu W, Wang J, Wang M, Hou H, Ding X, Ma L, et al. Oxidative Stress Factors Mediate the Association Between Life's Essential 8 and Accelerated Phenotypic Aging: NHANES 2005-2018. J Gerontol A Biol Sci Med Sci. 2024;79(1). Epub 2023/10/10. doi: 10.1093/gerona/glad240. PubMed PMID: 37813096.
20. He H, Chen X, Ding Y, Chen X, He X. Composite dietary antioxidant index associated with delayed biological aging: a population-based study. Aging (Albany NY). 2024;16(1):15-27. Epub 2024/01/04. doi: 10.18632/aging.205232. PubMed PMID: 38170244; PubMed Central PMCID: PMCPMC10817368.
21. Haskell WL, Lee IM, Pate RR, Powell KE, Blair SN, Franklin BA, et al. Physical activity and public health: updated recommendation for adults from the American College of Sports Medicine and the American Heart Association. Circulation. 2007;116(9):1081-93. Epub 2007/08/03. doi: 10.1161/CIRCULATIONAHA.107.185649. PubMed PMID: 17671237.
22. Bender R. Introduction to the use of regression models in epidemiology. Cancer epidemiology: Springer; 2009. p. 179-95.
23. Hastie TJ. Generalized additive models. Statistical models in S. 2017:249-307.
24. Muggeo VM. Estimating regression models with unknown break-points. Stat Med. 2003;22(19):3055-71. Epub 2003/09/16. doi: 10.1002/sim.1545. PubMed PMID: 12973787.
25. Wood SN. Stable and efficient multiple smoothing parameter estimation for generalized additive models. Journal of the American Statistical Association. 2004;99(467):673-86.
26. World Medical A. World Medical Association Declaration of Helsinki: Ethical Principles for Medical Research Involving Human Participants. JAMA. 2025;333(1):71-4. Epub 2024/10/19 20:42. doi: 10.1001/jama.2024.21972. PubMed PMID: 39425955.
27. Schottker B, Hagen L, Zhang Y, Gao X, Holleczek B, Gao X, et al. Serum 25-Hydroxyvitamin D Levels as an Aging Marker: Strong Associations With Age and All-Cause Mortality Independent From Telomere Length, Epigenetic Age Acceleration, and 8-Isoprostane Levels. J Gerontol A Biol Sci Med Sci. 2019;74(1):121-8. Epub 2018/10/30. doi: 10.1093/gerona/gly253. PubMed PMID: 30371905.
28. Liu L, Chen C, Lo K, Huang J, Yu Y, Huang Y, et al. Serum 25-hydroxyvitamin D, frailty, and mortality among the Chinese oldest old: Results from the CLHLS study. Nutr Metab Cardiovasc Dis.

2021;31(9):2707-15. Epub 2021/08/06. doi: 10.1016/j.numecd.2021.05.033. PubMed PMID: 34348876.

29. Veldurthy V, Wei R, Oz L, Dhawan P, Jeon YH, Christakos S. Vitamin D, calcium homeostasis and aging. Bone Res. 2016;4:16041. Epub 2016/10/30. doi: 10.1038/boneres.2016.41. PubMed PMID: 27790378; PubMed Central PMCID: PMCPMC5068478.

30. Ge D, Chen Q, Xie X, Li Q, Yang Y. Unveiling the potent effect of vitamin D: harnessing Nrf2/HO-1 signaling pathways as molecular targets to alleviate urban particulate matter-induced asthma inflammation. BMC Pulm Med. 2024;24(1):55. Epub 2024/01/26. doi: 10.1186/s12890-024-02869-2. PubMed PMID: 38273268; PubMed Central PMCID: PMCPMC10809564.

31. Pilz S, Gaksch M, Kienreich K, Grubler M, Verheyen N, Fahrleitner-Pammer A, et al. Effects of vitamin D on blood pressure and cardiovascular risk factors: a randomized controlled trial. Hypertension. 2015;65(6):1195-201. Epub 2015/03/25. doi: 10.1161/HYPERTENSIONAHA.115.05319. PubMed PMID: 25801871.

32. Chowdhury R, Kunutsor S, Vitezova A, Oliver-Williams C, Chowdhury S, Kiefte-de-Jong JC, et al. Vitamin D and risk of cause specific death: systematic review and meta-analysis of observational cohort and randomised intervention studies. BMJ. 2014;348:g1903. Epub 2014/04/03. doi: 10.1136/bmj.g1903. PubMed PMID: 24690623; PubMed Central PMCID: PMCPMC3972416 at www.icmje.org/coi_disclosure.pdf.

33. Rejnmark L, Bislev LS, Cashman KD, Eiriksdottir G, Gaksch M, Grubler M, et al. Non-skeletal health effects of vitamin D supplementation: A systematic review on findings from meta-analyses summarizing trial data. Plos One. 2017;12(7):e0180512. Epub 2017/07/08. doi: 10.1371/journal.pone.0180512. PubMed PMID: 28686645; PubMed Central PMCID: PMCPMC5501555.

34. LeBoff MS, Chou SH, Ratliff KA, Cook NR, Khurana B, Kim E, et al. Supplemental Vitamin D and Incident Fractures in Midlife and Older Adults. N Engl J Med. 2022;387(4):299-309. Epub 2022/08/09. doi: 10.1056/NEJMoa2202106. PubMed PMID: 35939577; PubMed Central PMCID: PMCPMC9716639.

35. Pludowski P, Grant WB, Karras SN, Zittermann A, Pilz S. Vitamin D Supplementation: A Review of the Evidence Arguing for a Daily Dose of 2000 International Units (50 microg) of Vitamin D for Adults in the General Population. Nutrients. 2024;16(3). Epub 2024/02/10. doi: 10.3390/nu16030391. PubMed PMID: 38337676; PubMed Central PMCID: PMCPMC10857599.

36. Marcinowska-Suchowierska E, Kupisz-Urbanska M, Lukaszkiewicz J, Pludowski P, Jones G. Vitamin D Toxicity-A Clinical Perspective. Front Endocrinol (Lausanne). 2018;9:550. Epub 2018/10/09. doi: 10.3389/fendo.2018.00550. PubMed PMID: 30294301; PubMed Central PMCID: PMCPMC6158375.

37. Batman A, Saygili ES, Yildiz D, Sen EC, Erol RS, Canat MM, et al. Risk of hypercalcemia in patients with very high serum 25-OH vitamin D levels. Int J Clin Pract. 2021;75(7):e14181. Epub 2021/03/25. doi: 10.1111/ijcp.14181. PubMed PMID: 33759301; PubMed Central PMCID: PMCPMC8250214.

38. Sajid U, Orton D, Kaufmann M, Jones G, Kline GA. Understanding Elevated Vitamin D Measurements to Uncover Hypercalcemia Etiology. Clin Chem. 2024;70(6):798-802. Epub 2024/06/03. doi: 10.1093/clinchem/hvae044. PubMed PMID: 38825340.

39. England PH. SACN vitamin D and health report 2016 [updated 2025-04-22]. Available from: https://www.gov.uk/government/publications/sacn-vitamin-d-and-health-report.

40. Turck D, Bresson J, Burlingame B. Scientific opinion on dietary reference values for vitamin D

EFSA Panel on Dietetic Products, Nutrition, and Allergies (NDA). EFSA J. 2016;179.

41. Holick MF, Binkley NC, Bischoff-Ferrari HA, Gordon CM, Hanley DA, Heaney RP, et al. Evaluation, treatment, and prevention of vitamin D deficiency: an Endocrine Society clinical practice guideline. J Clin Endocrinol Metab. 2011;96(7):1911-30. Epub 2011/06/08. doi: 10.1210/jc.2011-0385. PubMed PMID: 21646368.

42. Roth DE, Abrams SA, Aloia J, Bergeron G, Bourassa MW, Brown KH, et al. Global prevalence and disease burden of vitamin D deficiency: a roadmap for action in low- and middle-income countries. Ann N Y Acad Sci. 2018;1430(1):44-79. Epub 2018/09/19. doi: 10.1111/nyas.13968. PubMed PMID: 30225965; PubMed Central PMCID: PMCPMC7309365.

43. Demay MB, Pittas AG, Bikle DD, Diab DL, Kiely ME, Lazaretti-Castro M, et al. Vitamin D for the Prevention of Disease: An Endocrine Society Clinical Practice Guideline. J Clin Endocrinol Metab. 2024. Epub 2024/06/03. doi: 10.1210/clinem/dgae290. PubMed PMID: 38828931.

44. Santoro N, Roeca C, Peters BA, Neal-Perry G. The Menopause Transition: Signs, Symptoms, and Management Options. J Clin Endocrinol Metab. 2021;106(1):1-15. Epub 2020/10/24. doi: 10.1210/clinem/dgaa764. PubMed PMID: 33095879.

45. Crescioli C. The Role of Estrogens and Vitamin D in Cardiomyocyte Protection: A Female Perspective. Biomolecules. 2021;11(12). Epub 2021/12/25. doi: 10.3390/biom11121815. PubMed PMID: 34944459; PubMed Central PMCID: PMCPMC8699224.

46. McCurdy R, Jiang X, Clarkson TB, Nudy M, Schnatz PF. Vitamin D and conjugated equine estrogen: the association with coronary artery atherosclerosis in cynomolgus monkeys. Menopause. 2016;23(5):481-7. Epub 2016/02/18. doi: 10.1097/GME.0000000000000582. PubMed PMID: 26886883.

47. Elkhwanky MS, Kummu O, Piltonen TT, Laru J, Morin-Papunen L, Mutikainen M, et al. Obesity Represses CYP2R1, the Vitamin D 25-Hydroxylase, in the Liver and Extrahepatic Tissues. JBMR Plus. 2020;4(11):e10397. Epub 2020/11/20. doi: 10.1002/jbm4.10397. PubMed PMID: 33210060; PubMed Central PMCID: PMCPMC7657391.

48. Aatsinki SM, Elkhwanky MS, Kummu O, Karpale M, Buler M, Viitala P, et al. Fasting-Induced Transcription Factors Repress Vitamin D Bioactivation, a Mechanism for Vitamin D Deficiency in Diabetes. Diabetes. 2019;68(5):918-31. Epub 2019/03/06. doi: 10.2337/db18-1050. PubMed PMID: 30833469; PubMed Central PMCID: PMCPMC6477896.

Table 1. Characteristics of the study population

Characteristics	Male (n=6836)	Female (n=7074)	*P*-value
Age (mean±SD)	57.07 (11.34)	58.12 (11.82)	<0.001
Race			0.001[b]
Non-Hispanic white	3249 (47.53)	3288 (46.48)	
Non-Hispanic black	1360 (19.89)	1417 (20.03)	
Others ()	2227 (32.58)	2369 (33.49)	
Education level			0.394[b]
Below high school	1847 (27.02)	1831 (25.88)	
High school or equivalent	1588 (23.23)	1597 (22.58)	
Above high school	3401 (49.75)	3646 (51.54)	
Poverty-income ratio			<0.001[b]
≤1.0	1199 (17.54)	1447 (20.46)	
1.0-3.0	2839 (41.53)	3029 (42.82)	
>3.0	2798 (40.93)	2598 (36.73)	
BMI, kg/m^2			<0.001[b]
<25	1585 (23.19)	1841 (26.02)	
25-30	2749 (40.21)	2117 (29.93)	
≥30	2502 (36.60)	3116 (44.05)	
Smoking			<0.001[b]
Never smoker	2823 (41.30)	4297 (60.74)	
Previous smoker	2570 (37.60)	1666 (23.55)	
Current smoker	1443 (21.11)	1111 (15.71)	
Drinking (alcohol consumption)			<0.001[b]
Never drinker	527 (7.71)	1572 (22.22)	
Previous drinker	637 (9.32)	1466 (20.72)	
Current drinker	5672 (82.97)	4036 (57.05)	
Level of physical activity			<0.001[b]
Inactive	1775 (25.97)	2533 (35.81)	
Insufficient	802 (11.73)	1086 (15.35)	
Moderate	680 (9.95)	842 (11.90)	
High	3579 (52.36)	2613 (36.94)	
Sleep duration			<0.001[b]
<7 h	2572 (37.62)	2445 (34.56)	
7-8h	1858 (27.18)	1812 (25.61)	
≥8 h	2406 (35.20)	2817 (39.82)	
Hypertension			0.839[b]

No	3007 (43.99)	3067 (43.36)	
Yes	3829 (56.01)	4007 (56.64)	
Diabetes mellitus			<0.001[b]
No	5086 (74.40)	5515 (77.96)	
Yes	1750 (25.60)	1559 (22.04)	
Serum 25(OH)D, nmol/L			
Median (IQR)	63.80 (48.38, 80.20)	66.60 (47.00, 87.30)	<0.001[c]
<30	420 (6.14)	545 (7.70)	<0.001[b]
30-50	1451 (21.23)	1457 (20.60)	
50-75	2766 (40.46)	2293 (32.41)	
⩾75	2199 (32.17)	2779 (39.28)	
PhenoAge (median [IQR])	53.77 (45.07, 64.60)	53.05 (44.23, 64.13)	0.032
PhenoAgeAccel (median [IQR])	-2.40 (-5.13, 0.94)	-4.14 (-7.22, -0.46)	<0.001[c]
Standardized PhenoAgeAccel (mean±SD)	0.11 (0.95)	-0.16 (1.05)	<0.001[a]

[a] Student's t-test was used to compare means of variables between two groups

[b] Chi-square test was used to compare categorical variables between two groups.

[c] Mann-Whitney test was used to compare non-normal variables between two groups.

Table 2. Association between 25(OH)D serum level and PhenoAgeAccel

Characteristics	All			Male			Female		
	Beta coefficient (95% CI)		*P*-value	Beta coefficient (95% CI)		*P*-value	Beta coefficient (95% CI)		*P*-value
	Crude model	Adjusted model [a]		Crude model	Adjusted model [a]		Crude model	Adjusted model [a]	
Log(25(OH)D)	-0.27 (-0.32, -0.22)	-0.04 (-0.08, 0.00)	0.039	-0.13 (-0.20, -0.06)	0.03 (-0.02, 0.09)	0.334	-0.32 (-0.38, -0.26)	-0.07 (-0.12, -0.01)	0.007
Quartiles									
Q1	Reference	Reference		Reference	Reference		Reference	Reference	
Q2	-0.23 (-0.29, -0.17)	-0.10 (-0.16, -0.05)	<0.001	-0.16 (-0.24, -0.08)	-0.02 (-0.09, 0.04)	0.448	-0.33 (-0.41, -0.25)	-0.16 (-0.23, -0.08)	<0.001
Q3	-0.29 (-0.36, -0.23)	-0.10 (-0.16, -0.04)	0.001	-0.20 (-0.29, -0.11)	-0.03 (-0.12, 0.04)	0.355	-0.40 (-0.49, -0.32)	-0.15 (-0.22, -0.07)	<0.001
Q4	-0.32 (-0.38, -0.26)	-0.05 (-0.11, -0.00)	0.022	-0.16 (-0.24, -0.07)	0.02 (-0.04, 0.10)	0.553	-0.42 (-0.51, -0.34)	-0.10 (-0.18, -0.02)	0.009
P for trend			0.173			0.429			0.076
Categories based on the 2011 Institute of Medicine									
<30	Reference	Reference		Reference	Reference		Reference	Reference	
30-50	-0.23 (-0.33, -0.13)	-0.14 (-0.23, -0.05)	0.002	-0.26 (-0.41, -0.11)	-0.15 (-0.29, -0.02)	0.024	-0.22 (-0.34, -0.09)	-0.10 (-0.21, 0.00)	0.057
50-75	-0.42 (-0.52, -0.33)	-0.21 (-0.30, -0.13)	<0.001	-0.39 (-0.53, -0.25)	-0.16 (-0.29, -0.04)	0.009	-0.51 (-0.63, -0.40)	-0.24 (-0.33, -0.15)	<0.001
⩾75	-0.46 (-0.56, -0.36)	-0.15 (-0.24, -0.06)	0.001	-0.36 (-0.50, -0.22)	-0.09 (-0.22, 0.03)	0.131	-0.54 (-0.66, -0.42)	-0.17 (-0.27, -0.07)	0.001
P for trend			0.130			0.366			0.014

[a] Adjusted for age, race, education level, poverty-income ratio, body mass index, smoking, drinking, physical activity, sleep duration,

hypertension, diabetes and season of measurement.

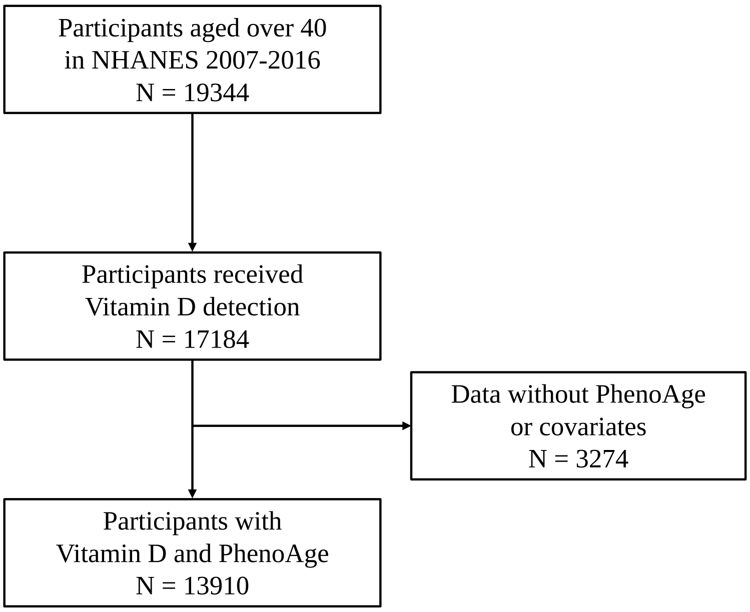

Figure 1 Flowchart of participants selection.

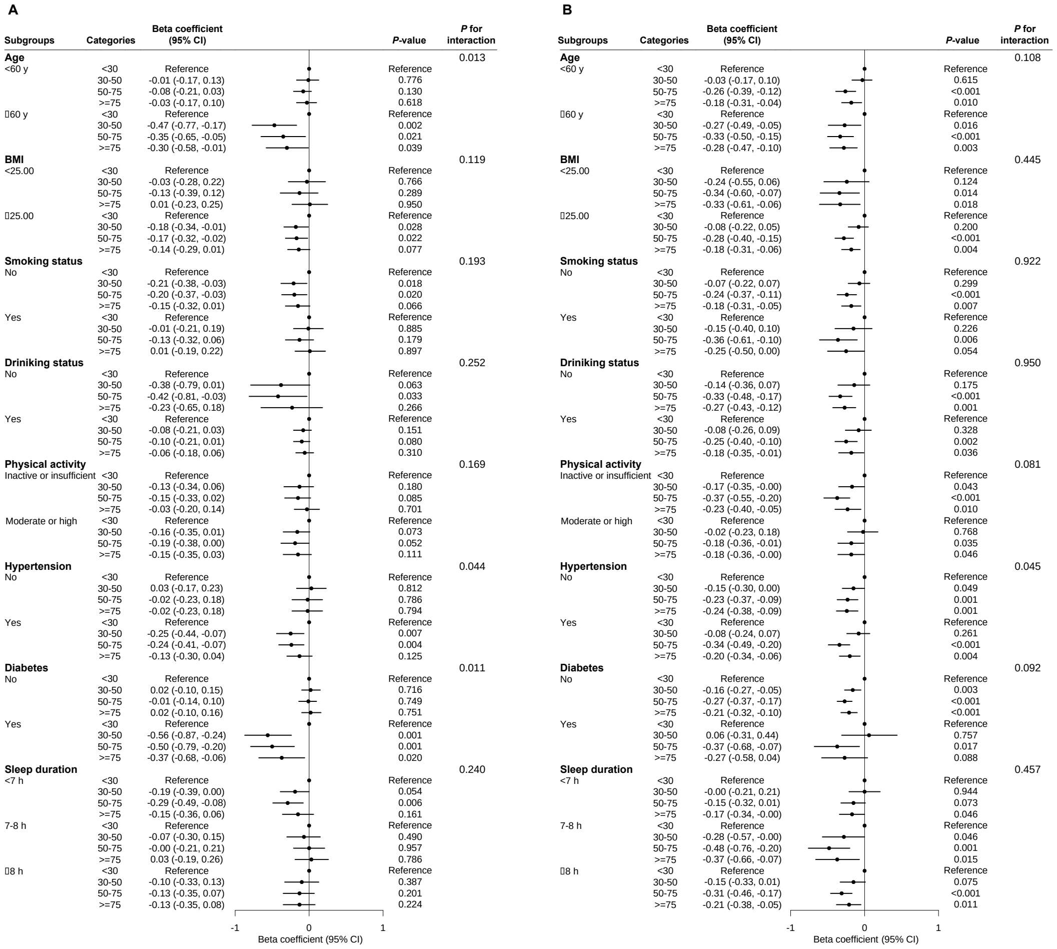

Figure 2 Subgroup analyses of the association between 25(OH)D categories (based on the 2011 Institute of Medicine) and PhenoAgeAccel among males (A) and females (B). The filled circles and horizontal lines represent regression coefficients and the 95% confidence intervals. Multiple linear regression models were adjusted for age, race, education level, poverty-income ratio, body mass index, smoking, drinking, physical activity, sleep duration, hypertension, diabetes and season. BMI, body mass index; CI, confidence intervals.

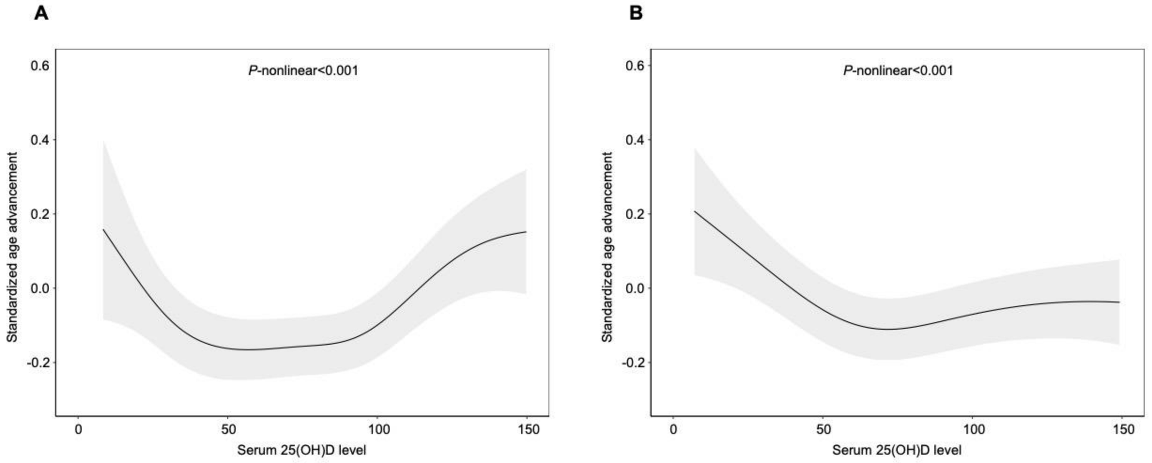

Figure 3 Nonlinear relationships between 25(OH)D serum level and PhenoAgeAccel among males (A) and females (B). Generalized additive models were adjusted for age, race, education level, poverty-income ratio, body mass index, smoking, drinking, physical activity, sleep duration, hypertension, diabetes and season. The black solid line represents the smooth curve fit between variables. The grey band represents the 95% confidence interval of the fit.

Table 3. Results of the analysis of the relationship between 25(OH)D serum levels and PhenoAge

25(OH)D serum levels (nmol/L)	Beta coefficient (95% CI)	*P*-value
Male		
<38.20	-0.013 (-0.025, -0.002)	0.017
38.20-91.55	0.001 (-0.001, 0.002)	0.487
⩾91.55	0.005 (0.002, 0.008)	<0.001
Female		
<62.50	-0.007 (-0.010, -0.004)	<0.001
62.50-125.00	0.002 (0.000, 0.004)	0.029
⩾125.00	-0.001 (-0.005, 0.002)	0.535

Generalized additive models were adjusted for age, race, education level, poverty-income ratio, body mass index, smoking, drinking, physical activity, sleep duration, hypertension, diabetes and season.

Click here to access/download
Supporting Information
Supplemental materials.docx

Click here to access/download
Other
PLOSOne_Human_Subjects_Research_Checklist.docx

Association of 25(OH)D serum level with biological aging: A Cross-Sectional Study of 2007-2016 NHANES surveys

Yi Feng[a]; Licheng Yu[b]; Jue Wang[a] *

[a] Nanhui new town Community Health Service Center, Pudong New Area, Shanghai, 201308, China.

[b] Prevention and Health Care Department, Heqing Community Health Service Center of Pudong New District, Shanghai, 201201, China.

Corresponding author. Jue Wang

E-mail address: wangjue180130@126.com

Abstract

Vitamin D plays a crucial role in various aspects of human body, including aging, but chronological age may not accurately reflect the true biological aging status. Recently, ~~PhenoAge~~PhenoAge has been developed to estimate an individual's biological age based on different biological and clinical measures. Therefore, we investigated the relationship between 25(OH)D serum levels and biological aging calculated by PhenoAge Acceleration (PhenoAgeAccel)~~PhenoAge~~ using data from the 2007-2016 National Health and Nutrition Examination Survey (NHANES)~~NHANES survey~~. 25(OH)D serum levels showed a negative association with PhenoAgeAccel ~~PhenoAge acceleration (PhenoAgeAccel)~~ (β=-0.04; 95% confidence interval [CI]~~CI~~: -0.08 to 0.00~~, p=0.039~~). This negative ~~correlation~~association was ~~only found to be~~ dose-dependent ~~for~~in females (β=-0.07; 95% CI: -0.12 to 0.01~~, p=0.007~~), but not ~~for~~ in males. Generalized additive models further found 25(OH)D serum levels were linked to negative PhenoAgeAccel at levels below 38.2 nmol/L (15.3 ng/mL) in males (β=-0.013; 95% CI: -0.025 to -0.002) and 62.5 nmol/L (25.0 ng/mL) in females (β=-0.007; 95% CI: -0.01 to -0.004,), ~~showed nonlinear associations were~~ revealing a U-shaped relationship observed in males but an L-shaped pattern in females. ~~Specifically, 25(OH)D serum levels were linked to reduced PhenoAgeAccel at levels below 38.20 nmol/L (15.28 ng/mL) in males (β=-0.013; 95% CI: -0.025 to -0.002, p=0.017) and 62.50 nmol/L (25ng/mL) in females (β=-0.007; 95% CI: -0.01 to -0.004, p<0.001)~~. However, 25(OH)D serum levels above 125 nmol/L were not associated with ~~PhenoAge~~PhenoAgeAccel in females (β=-0.001; 95% CI: -0.005-0.002), while in males, elevated levels (>91.55 nmol/L) were associated with ~~increased~~ positive ~~PhenoAge~~PhenoAgeAccel (β=0.005~~ SD~~; 95% CI: 0.002-0.008~~, p<0.001~~). Our findings indicate that vitamin D insufficiency~~deficiency~~ ~~shows~~ has an inverse link with~~a potential link to~~ accelerated biological aging, and high levels of vitamin D in males ~~link to~~ accelerated biological aging as well, offering valuable insights into the relationship between vitamin D and biological aging.

Key words: Vitamin D; Serum 25(OH)D; Biological aging; PhenoAgePhenoAge; Nonlinear association

1. Introduction

Vitamin D is a fat-soluble vitamin that plays a crucial role in various aspects of human health, with its effects extending to bone metabolism, immune function, etc. It is acquired through two primary sources: endogenous production (mainly vitamin D3) in the skin upon exposure to ultraviolet B (UVB) radiations, and exogenous intake (vitamin D2 and D3) through dietary sources, such as fatty fish, egg yolks or supplements. Once absorbed in the body, vitamin D is converted into 25-hydroxyvitamin D (25(OH)D) in the liver, then transformed into the biologically active form, 1α,25-dihydroxyvitamin D (1α,25$(OH)_2$D), in the kidneys [1]. This active form binds to the vitamin D receptor (VDR), regulating calcium and phosphate metabolism [2]. Serum 25(OH)D is the most commonly measured form of vitamin D in humans because it is the major circulating form and provides an accurate indicat~~or~~ion of the body's overall vitamin D status [3]~~,~~; and it can be influenced by various factors~~,~~ including the geographic location, race and food intake [4].

Vitamin D deficiency is a global public health concern, associated with increased risk of type 2 diabetes (T2D), hypertension, multiple sclerosis [5-7]. The prevalence of vitamin D deficiency is particularly high among the elderly [8], with some studies suggesting that it may accelerate chronological aging and contribute to age-related diseases and inflammatory diseases, such as cardiovascular disease, multiple sclerosis and rheumatoid arthritis [9]. However, several large randomized clinical trials of vitamin D supplementation failed to demonstrate significant benefits on cardiovascular disease, casting doubt on the evidence from observational studies linking vitamin D deficiency with cardiovascular disease [10, 11]. This ambiguity highlights challenges in assessing the association between vitamin D and aging. A major limitation of previous studies is that they relied on chronological age which is quite discrepant with biological age. Indeed, unlike ~~the~~ chronological age which is a precise reflection of an individual's lifetime, ~~the~~ biomarkers~~ that~~ mak~~e~~ing up the biological age are mainly determined by a combination of genetic, environmental and lifestyle factors [12]. Accordingly, some individuals may age biologically faster or slower than indicated by

their chronological age.

To better characterize physiological aging in epidemiological studies, several aging metrics based on the combination of clinical (e.g., ~~PhenoAge~~PhenoAge and GrimAge) and biological (~~DNAmPhenoAge~~PhenoAge acceleration) markers have been developed [13]. In a post hoc analysis of the European DO-HEALTH randomized clinical trial involving 777 participants with a mean 25(OH)D serum level of ~~22.69~~ 56.75 n~~g~~mol/~~m~~L, ~~DNAm PhenoAge~~PhenoAge ~~–~~was the only biological age metric shown to be slowed by daily vitamin D (2000 UI) supplementation among the four biological age metrics that were assessed:~~ DNAm~~ ~~PhenoAge~~PhenoAge, GrimAge, GrimAge2 and DunedinPACE [14]. PhenoAgeAccel ~~PhenoAge~~ was developed and validated using machine learning methods on a National Health and Nutrition Examination Survey (NHANES) dataset of 4000 adults aged 20 to 84 years and it showed a mean absolute error of approximately 3.6 years when predicting the chronological age [15]. ~~The calculation of PhenoAge is based on the addition of serum levels of the following nine biomarkers on top of the chronological age.~~ Although vitamin D supplementation was shown to slow the PhenoAgeAccel ~~PhenoAge~~ value in older adults with low 25(OH)D serum concentrations, observational studies linking 25(OH)D serum concentrations with PhenoAgeAccel ~~PhenoAge~~ are lacking. Therefore, in this study, we assessed whether there is a linear and/or non-linear relationship between 25(OH)D serum concentration and PhenoAgeAccel ~~PhenoAge~~ using datasets of 2007-2016 NHANES surveys. By exploring this relationship, the study aims to enhance our understanding of the link between serum vitamin D levels and biological ag~~e~~ing in human beings for the benefit of public health.

2. Materials and Methods~~Method~~

2.1 Study design and population

This study was conducted and reported in agreement ~~to~~ with STROBE guidelines for reporting observational studies. This study utilized data derived from the 2007-2016 NHANES~~, surveys~~ conducted in the United States of America by the National Center

Field Code Changed

Field Code Changed

Field Code Changed

for Health Statistics of the Centers for Disease Control and Prevention. All data included in the current study are publicly available on the NHANES website [16]. NHANES includes in-home interview, physical measurement and laboratory tests. The 2007-2016 NHANES datasets involved 50558 adult participants.~~This study is based on data from 50558 participants of the 2007-2016 NHANES datasets. We excluded~~ participants with incomplete information and participants aged less than 40 years old were excluded. After excluding, ~~A~~a total of 13,910 participants were finally included in this analysis (Figure 1).

2.2 Measurement of ~~serum~~ 25(OH)D serum ~~concentrations~~level

~~Serum v~~Vitamin D serum levels were reflected by 25(OH)D~~ levels~~. Because the forms of 25(OH)D measured in the 2007-2016 NHANES samples included both 25(OH)D2 and 25(OH)D3 (classic 25(OH)D3 and C3 epimer of 25(OH)D3 [C3-epi-25(OH)D3]), serum 25(OH)D included 25(OH)D2, 25(OH)D3 and C3-epi-25(OH)D3 in this study. During the study period (2007-2016), 25(OH)D levels were measured in the NHANES population using the liquid chromatography-tandem mass spectrometry (LC-MS/MS). According to the 2011 Institute of Medicine's Committee recommendations, we transformed serum 25(OH)D concentrations into a categorical variable with the following modalities: ~~categorical variables: risk of~~ deficiency (<30 nmol/L), insufficiency ~~risk of inadequacy~~ (30–50 nmol/L), ~~likelihood of~~ sufficiency (50–75 nmol/L) and abnormally high ~~no increased benefit~~ (≥75 nmol/L)~~[17, 18]~~[17].

2.3 Measurement of PhenoAge ~~biological aging~~

In this study, we utilized PhenoAge ~~PhenoAge~~ as the indicator of biological aging, considering its high robustness and feasibility in NHANES data as a reliable predictor of mortality and age-related disease risks. The PhenoAgeAccel ~~PhenoAge~~ algorithm ~~is~~ was constructed by applying elastic-net regression methods to NHANES III (1988-1994) datasets~~using NHANES III (1988–1994) by elastic-net regression~~, encompasses 10 markers: serum albumin, serum creatinine, plasma glucose, white blood cell count,

Field Code Changed
Formatted: Indent: First line: 2 ch
Formatted: Font: (Default) Times New Roman
Field Code Changed
Formatted: Font: (Default) Times New Roman
Formatted: Indent: First line: 2 ch
Formatted: Font: (Default) Times New Roman

blood lymphocyte percent ~~lymphocyte percent~~, red cell distribution width, mean red cell volume, serum C-reactive protein, serum alkaline phosphatase and chronological age [18]. ~~The R package BioAge was used to compute PhenoAge [15]. PhenoAge was both developed and validated within NHANES populations. Meanwhile, a~~All constituent biomarkers of PhenoAge were ~~are~~ routinely measured ~~in~~ during NHANES ~~with~~ using standardized protocols~~,~~ ensuring reproducibility across study cycles. One of the markers, CRP, was unavailable from 2011 to 2018 datasets. Previous studies have applied an algorithm that excluded CRP to calculate ~~PhenoAge~~PhenoAge, demonstrating good applicability [19, 20]. To assess the validity of using ~~PhenoAge~~PhenoAge without CRP data, we calculated 2 ~~PhenoAge~~PhenoAge estimates with and without C-reactive protein, using data from cycles 2007-2008 and 2009-2010, which contained all 10 markers. We found a strong ~~correlation~~ association between 2 ~~PhenoAge~~PhenoAge estimates with and without CRP (Pearson's correlation coefficient of 0.986). Based on ~~these~~ this finding~~s~~, we proceeded with the main ~~analysis~~analysis of data from the five NHANES cycles using the ~~PhenoAge~~PhenoAge algorithm excluding CRP~~, employing data from five NHANES cycles~~. We further calculated ~~PhenoAge acceleration (PhenoAge~~PhenoAgeAccel~~)~~ as the difference between ~~PhenoAge~~PhenoAge and chronological age. A positive value of ~~PhenoAge~~PhenoAgeAccel represents accelerated biological aging process, while a negative value represented slower biological aging process. Subsequently, ~~PhenoAge~~PhenoAgeAccel was standardized to have a mean of 0 and a standard deviation (SD) of 1.

2.4 Covariates

NHANES interviews were conducted by trained interviewers.~~,~~ ~~where q~~Questionnaires were administered to participants to collect ~~collect~~ information on demographics (chronological age, gender, race, education level, poverty income ratio (PIR) and lifestyle-related factors ~~lifestyle~~ (tobacco smoking, ~~and~~ alcohol consumption ~~variable~~, physical activity, and comorbidities). The~~B~~ body mass index (BMI) were

Field Code Changed

Field Code Changed

Formatted: Font: (Default) Times New Roman

Formatted: Font: (Default) Times New Roman

Field Code Changed

Formatted: Indent: First line: 1.5 ch

Formatted: Font: (Default) Times New Roman

measured at the Mobile Examination Center.

Covariates in regression analyses testing the association between 25(OH)D serum levels and biological aging include age, race, education level, PIR, BMI, tobacco smoking, alcohol consumption, physical activity. The variable active tobacco smoking was classified into three categories: never smokers (previous smoking of a total of less than 100 cigarettes), current smokers (lifetime smoking of over 100 cigarettes) and former smokers (had smoked ≥100 cigarettes in their lifetime but had quit smoking at the time of the survey). The, variable –alcohol consumption ~~variable~~ was classified as never drinkers (participants who had ~~had~~ drunk <12 drinks in their lifetime), current drinkers (participants who had ~~had~~ drunk ≥12 drinks in their lifetime and at least one drink in the past year), and former drinkers (participants who had~~had~~ drunk ≥12 drinks in their lifetime but no drink in the past year). A~~M~~ Metabolic ~~e~~Equivalent task (MET)-min per week were calculated to assess physical activity, levels (in min per week) that ~~and levels of physical activity~~ were categorized as: inactive (no regular physical activity), insufficient (<500 MET-min activity/week), moderate (500-1,000 MET-min activity/week) and high (≥1,000 MET-min activity/week) [21]. For sleep duration, participants responded to the question: "How much sleep do you usually get at night on weekdays or on workdays?" ~~and~~Sleep duration was ~~were~~ categorized into 3 groups: <7 h, 7-8 h and ≥8h). The definition of hypertension was based on self-reports, the use of antihypertensive drugs or on the record of a systolic blood pressure≥140 mmHg with or without a diastolic blood pressure≥90 mmHg on physical exam. The definition of diabetes mellitus was based on self-reports or on laboratory test results showing fasting blood glucose levels ≥126 mg/dL or HbA1c values ≥6.5%.

2.5 Statistical analysis

Weighted multivariable linear regression models were constructed to estimate the association between serum 25(OH)D status and ~~PhenoAge~~PhenoAgeAccel. 25(OH)D

Formatted: Font: (Default) Times New Roman

Field Code Changed

serum levels were log-transformed due to their skewed distribution. To evaluate whether the association between 25(OH)D serum levels and ~~PhenoAge~~PhenoAgeAccel was not just linear but also dose-dependent~~ potential dose-response patterns~~, 25(OH)D serum level was analyzed as both a continuous (log-transformed) and a categorical variable. In agreement with standards for linear regression analyses involving continuous dependent variables, the association between 25(OH)D serum levels as continuous variables and ~~PhenoAge~~PhenoAgeAccel was estimated based on beta coefficients with their 95% confidence intervals [22]. All models were adjusted for chronological age, gender, race, education level, PIR, BMI, tobacco smoking and alcohol consumption variable, physical activity, sleep duration, hypertension, diabetes and season of measurement.

To explore whether there is also a non-linear relationship between 25(OH)D serum levels and ~~PhenoAge~~PhenoAgeAccel, a Generalized Additive Model (GAM) was employed to fit the smooth curve~~-~~. The GAMs extend traditional linear regression by replacing linear terms with smooth functions, allowing flexible modeling of complex dose-response relationships without assuming a priori parametric forms [23]. The threshold effect was further estimated using segmented regression model. This method identifies inflection points where the slope of the association between 25(OH)D serum levels and ~~PhenoAge~~PhenoAgeAccel changes significantly. The optimal breakpoint was determined via likelihood ratio tests, comparing the goodness-of-fit between the non-segmented linear model and the segmented regression model [24]. Results of *P*-value of <0.05 were considered to be statistically significance ~~A *P*-value of <0.05 was considered statistical significance~~. All data analyses were performed using R software version 4.2.1 (R Core Team, Vienna, Austria). R package 'BioAge' was used to calculate ~~PhenoAge~~PhenoAge [15], while the package 'mgcv' was used to conduct GAM analysis [25]. Continuous data were reported as means ± standard deviations (SDs) for normal distributions or as ~~or~~ medians (interquartile ~~range)~~ranges) for skewed distributions. Categorical data were reported as numbers (percentages).

Field Code Changed

Field Code Changed

Formatted: Font: (Default) Times New Roman

Field Code Changed

Formatted: Font: (Default) Times New Roman

Field Code Changed

Field Code Changed

Formatted: Font: (Default) Times New Roman

2.6 Ethical considerations

This study utilized data from NHANES study, which was conducted in accordance with the principles in the Declaration of Helsinki. The NHANES protocol received prior ethical approval from the National Center for Health Statistics' Review Board. Since our study is a secondary analysis of publicly accessible, de-identified NHANES data sets, additional institutional review board approval was not required [26] ~~[27, 28]~~.

Formatted: Indent: First line: 2 ch

Field Code Changed

Field Code Changed

3. Results

3.1 Characteristics of the study population

The current study included a total of 6836 males and 7074 females, with the characteristics of the study population presented in Table 1. There are significant differences between males and females regarding ~~in~~ the distributions of age, race, BMI, and poverty-income ratio (p < 0.001). Additionally, gender-specific differences were noted in terms of ~~in~~ behaviors influencing biological aging, such as smoking, drinking, physical activity, and sleep duration~~, were also observed between males and females~~ (p < 0.001). Notably, males had a higher prevalence of diabetes mellitus ~~diabetes~~ than females. The median 25(OH)D serum level in females was 66.6~~0~~ nmol/L compared with 63.8~~0~~ nmol/L in males (p<0.001). The PhenoAgeAccel~~,~~ of males and females were -2.40 (-5.13, 0.94) and -4.14 (-7.22, -0.46) respectively, suggesting that PhenoAgeAccel ~~the PhenoAge~~ was lower in females than in males.

3.2 Association between 25(OH)D serum level ~~with~~ and biological aging

The associations between 25(OH)D serum levels and ~~PhenoAge~~PhenoAgeAccel were presented in Table 2. After adjusting for age, race, education level, PIR, BMI, smoking, alcohol consumption, level of physical, sleep duration and presence of hypertension and/or diabetes mellitus~~.~~, ~~Serum~~ 25(OH)D serum levels ~~was found to be~~ were negatively associated with ~~PhenoAge~~PhenoAgeAccel (β=-0.04 SD; 95% CI: -0.08 to 0.00). Segregating this data by sex, ~~this~~ the association was statistically significant only in females (p=0.007). ~~To further explore this relationship, the population was~~

~~categorized into four groups based on quartiles of 25(OH)D serum level.~~ When 25(OH)D serum levels were subdivided into quartiles, 25(OH)D ~~25(OH)D~~ serum levels were inversely associated with ~~PhenoAge~~PhenoAgeAccel, with no apparent dose-dependent trend (p for trend =0.173). When segregating the data by sex, 25(OH)D serum levels were inversely associated with PhenoAgeAccel only in women, with no apparent dose-response relationship (p for trend = 0.076). When categorizing 25(OH)D serum levels as recommended in the 2011 USA institute of Medicine guidelines, 25(OH)D serum levels were inversely associated with PhenoAgeAccel for 25(OH)D serum levels between 30-50 nmol/L and 50-75 nmol/L in males (with no apparent dose-response relationship), and between 50-75 nmol/L and ≥75 nmol/L in females, with an apparent dose-response relationship (p=0.014).~~we found the population was categorized into four groups according to the IMO guideline, which similarly revealed that higher 25(OH)D serum levels were associated with reduced PhenoAgeAccel, especially in females (p for trend =0.014). Additionally, 25(OH)D levels at 30-75 nmol/L were associated with reduced PhenoAgeAccel in males categorized based on IMO guidelines, though these statistical differences were not observed in males based on quartile categories.~~

Results of subgroup/effect modification analysis showed that ~~Stratified analysis furtherly revealed that no overall association was observed in males; however,~~ in males high 25(OH)D serum levels were associated with negative PhenoAgeAccel only in people aged over 60 years, those with hypertension and those with diabetes mellitus, suggesting that ~~subpopulations over 60 years of age with hypertension or diabetes, higher 25(OH)D serum levels were associated with reduced PhenoAgeAccel. Additionally,~~ there were statistically significant interactions between 25(OH)D serum levels and age, hypertension, ~~or~~ and diabetes (Fig.2A). In females, we did not find any effect modifier for the association between 25(OH)D serum levels and PhenoAge Accel~~mediators such as age, BMI, smoking, drinking, physical activity, and hypertension did not alter the inverse association between 25(OH)D serum levels and PhenoAgeAccel, indicating the robustness of this relationship~~ (Fig.2B).

3.3 Association of 25(OH)D serum levels with ~~PhenoAge~~PhenoAgeAccel on GAM

Given the lack of linear associations between 25(OH)D serum levels and PhenoAgeAccel when subdividing 25(OH)D serum levels into quartiles (Table 2), ~~the non-significant trend in Table 2 indicating a non-linear correlation,~~ GAM was utilized to investigate the potential linkage between serum 25(OH)D and ~~PhenoAge~~PhenoAgeAccel. There was a nonlinear relationship between 25(OH)D serum level and ~~PhenoAge~~PhenoAgeAccel in both males and females (p<0.001) (Fig 3 A-B). In men, the relationship was U-shaped with the two inflection points at 38.2 nmol/L~~15.8 ng/mL (38.2 nmol/L)~~ and ~~36.6 ng/mL (~~91.5 nmol/L~~)~~ and in women, the segmented regression modelling confirmed a L-shaped relationship. Low 25(OH)D serum levels (<38.2 nmol/L) were negatively ~~correlated~~ related ~~with~~ to ~~PhenoAge~~PhenoAgeAccel (β=-0.013 SD; 95% CI: -0.025 to -0.002), whereas high levels (>91.55 nmol/L) showed a positive association (β=0.005 SD; 95% CI: 0.002 to 0.008). For females, 25(OH)D serum levels below 62.5 nmol/L were negatively linked to ~~PhenoAge~~PhenoAgeAccel (β=-0.007 SD; 95% CI: -0.01 to -0.004), with no significant association observed beyond 125 nmol/L (β=-0.001 SD; 95% CI: -0.005 to -0.002). Overall, low (<38.2 nmol/L) or high (>91.55 nmol/L) 25(OH)D serum levels in males were associated with ~~PhenoAge~~PhenoAgeAccel, while in females, only low levels (<62.5 nmol/L) was associated with ~~pose a risk factor~~ for PhenoAgeAccel ~~biological aging~~ (Table 3).

4. Discussion

In the current study, the association between 25(OH)D serum level and biological aging was investigated in the 2007-2016 NHANES surveys. The analysis revealed a negative association between 25(OH)D serum levels and PhenoAge. The effect modifier analysis showed that age above 60 years, diabetes mellitus and hypertension modified the association between 25(OH)D serum levels and PhenoAge in men, but no modifier was found for that association in females. When subdividing 25(OH)D serum

levels into quartiles and stratifying data by sex, this association was still observed only in females, but in a non-linear fashion. When further subdividing 25(OH)D serum levels as recommended by the Institute of Medicine in USA in 2011, the association persisted for 25(OH)D serum levels between 30-50 nmol/L and between 50-75 nmol/L in males and between 50-75 nmol/L and $\geqslant$ 75 nmol/L, with a linear pattern of relationship observed only in females.biological age with 25(OH)D serum level, whether categorized by quartiles or according to IOM guideline. Additionally, the trends and association can be observed in females but not in males. Non-linear associations were also found between 25(OH)D serum levels and PhenoAgePhenoAgeAccel in both males (U-shaped) and females (L-shaped).

Previous Numerous studies have established that 25(OH)D serum level, representing vitamin D status in our body, are associated with various diseases, including all-cause mortality, cardiovascular diseases, and aging etc. Multiple large-scale observational studies have consistently shown a negative correlation between 25(OH)D serum levels and all-cause mortality risk, lower 25(OH)D serum levels are associated with an increased risk of all-cause mortality, and a dose-response relationship observed [28, 29]. Individuals with lower vitamin D levels (typically below 30 ng/mL) linked to a higher risk of all-cause mortality compared to those with adequate vitamin D status [30]. Regarding aging, sstudies have reported that a gradual decline in 25(OH)D serum levels with chronological age, and vitamin D supplementation can mitigate oxidative damage and improve immune function in elder people, suggesting its potential role in slowing age-related physiological decline [27]. This decline is attributed to several factors, including decreased sun exposure, reduced capacity of the skin to synthesize vitamin D, and decreased dietary intake of vitamin D, resulting in vitamin D deficiency in older adults [28]. The synthesis of vitamin D from sun exposure vary in different races, but our study lacks this data of sun exposure. Additionally, we also found that low 25(OH)D serum levels were associated with positive PhenoAgeAccel increased biological aging, suggesting vitamin D may also play a crucial role in the regulation of the aging process. Adequate 25(OH)D levels are

Field Code Changed

Field Code Changed

Field Code Changed

Field Code Changed

essential for maintaining calcium absorption and bone health preventing osteoporosis, a common age-related condition, and regulating calcium homeostasis and bone metabolism [29]. Moreover, vitamin D can interact with antioxidant and anti-inflammatory pathways, such as activating Nrf2 (nuclear factor erythroid 2-related factor 2) and SIRT1 (sirtuin 1) signaling cascades, and suppressing the production of pro-inflammatory cytokines, including interleukin-6 (IL-6) and tumor necrosis factor-alpha (TNF-α), through inhibiting nuclear factor-κB (NF-κB). These actions lead to reduction of inflammation and oxidative stress, both of which are known to contribute to the aging process [29, 30]. Therefore, vitamin D and aging have a mutual influence on each other.

Studies report that vVitamin D supplementation could be an effective way to reverse or mitigate diseases, including lower blood pressure and improve endothelial function, leading to reduced risk of cardiovascular events, such as heart attacks and strokes, and improved cardiovascular outcomes [31]. A meta-analysis of 75 randomized controlled trials in 2014 revealed that vitamin D supplementation was associated with a 6% reduction in all-cause mortality [32]. Some studies suggest that the impact of vitamin D supplementation on all-cause mortality may be more pronounced in certain subgroups, such as older adults or individuals with vitamin D deficiency. For example, vitamin D supplementation was associated with a reduced risk of all-cause mortality in trials with a mean baseline 25(OH)D level <3075 ngmol/mL (RR 0.89, 95% CI 0.80-0.99), but not in trials with a mean baseline 25(OH)D level ≥7530 ngmol/mL (RR 1.04, 95% CI 0.97-1.11) [33]. Thus, these evidence highlights the potential of vitamin D supplementation in reducing the risk of several chronic diseases, particularly in elderly people, where serum vitamin D levels tend to decline with age.

Despite its potential benefits, the use of vitamin D supplements has increased markedly, and concerns about excessive intake have emerged. the Vitamin D and Omega-3 Trial (VITAL) study, a randomized trial involving over 25,000 participants, discovered that vitamin D supplementation did not significantly reduce the risk of all-cause mortality or lower risk of fractures among generally healthy midlife and older

adults without vitamin D deficiency or osteoporosis [34]. Furthermore, increased intake of vitamin D supplements by the general population and a growing number of high-dose prescriptions without medical monitoring might result in a greater risk of excessive vitamin D toxicity [35]. Our current results indicated that the correlation association of 25(OH)D serum level and biological aging followed a U-shaped curve in males and an L-shaped curve in females, revealing that high serum level of 25(OH)D is not associated with decelerated aging in either males and females, and even associated with accelerated biological aging in males. These detrimental effects may stem from the toxicity of excessive vitamin D. When vitamin D intake is too high, serum levels of its metabolites [25(OH)D and 24,25(OH)$_2$D] rise beyond the binding capacity of vitamin D binding protein, leading to an increase in the free active metabolite, 1,25(OH)$_2$D. This active form reaches the VDR in the nucleus, constantly promoting gene expression that enhances calcium absorption and bone mobilization [36]. Consequently, this can result in hypercalcemia or hyperphosphatemia, which in turn suppresses initial parathyroid hormone (iPTH) level [36, 37]. Such disrupting balance of calcium and hormone may contribute to cardiovascular disease, kidney damage, and skin aging. Research reported that the working diagnosis of vitamin D toxicity is diagnosed based on total 25(OH)D concentrations of 3280~2080ngmol/mL (80~200 nmol/L) detected by immunoassay [38]. Our results found the 25(OH)D levels over 91.5 nmol/L in male participants were not linked to accelerated biological aging, approximately consistent with those reports.

Our study identified a transition point in relationship between 25(OH)D serum level and biological aging, with males exhibiting a threshold at 38.20 nmol/L and females at 62.50 nmol/L. Beyond this transition point, the increase in 25(OH)D level no longer associates with biological aging deceleration. Regarding the threshold of vitamin D deficiency, it remains an ongoing controversy. The Scientific Advisory Committee on Nutrition (2016) recommended threshold at 25 nmol/L among UK population [39], whereas the IOM (2011) suggested thresholds at 30 nmol/L based on evidence in U.S. and Canadian populations [17] [17]. Meanwhile, the European Food Safety Authority 2016 and the

Endocrine Society guideline 2011 proposed higher level of 50 nmol/L as deficiency cutoff [40, 41]. The discrepancy in cutoff values stems from multiple factors, for example, different regional ultraviolet B exposure patterns, dietary practice and population-specific biological variability. Furthermore, guideline variations also reflect different focus on health outcomes, with some prioritizing musculoskeletal health [42]. In 2024, the Endocrine Society guideline retracted their previous recommendation of threshold at 50 nmol/L due to limited clinical trial evidence [43]. It also highlighted the impracticality of universal thresholds due to heterogeneity in health outcomes and population characteristics. Hence, the interpretation of 25(OH)D level threshold determination in our study should be with caution, especially when it derived from observational study in an ethnically diverse population. Nevertheless, the trend of benefits attenuation we observed is worth noting, suggesting excessive level of serum 25(OH)D may not be beneficial regarding biological aging process.

Our results reported differences of dose-response curve between in male and in female, which may be attributed to the sex hormones, especially estrogen. As women age, particularly during the menopausal transition and after menopause, their ovaries produce less estrogen, leading to a decline of estrogen levels. This decline contributes to the thinning and wrinkling of the skin, as well as changes in hair texture and growth patterns [44]. Some studies suggest that vitamin D could enhance the activity of estrogen receptors (ER) via interacting with them, potentially amplifying the anti-aging effects of estrogen in the body [45]. Vitamin D and estrogen also have a synergistic effect on bone metabolism, helping to prevent osteoporosis and fractures, especially in postmenopausal women [46]. Additionally, vitamin D regulate estrogen metabolism, leading to higher circulating estrogen levels and subsequently delay aging [45]. Therefore, high level of serum 25(OH)D may not be associated with biological aging in females, but the mechanisms underlying the adverse effects of extremely high levels (>100 nmol/L) in males remain unclear. Further studies are needed to clarify the complex interplay between these two important hormonal systems.

The current study has several limitations. First, as a cross-sectional investigation,

Field Code Changed
Field Code Changed
Field Code Changed
Field Code Changed
Field Code Changed
Field Code Changed
Field Code Changed

it only provides information on the association between serum 25(OH)D levels and biological aging, making it challenging to establish causal relationships. Second, the classification of 25(OH)D serum levels used in our study is based on the 2011 IOM guidelines; however, more recent classifications, such as those from the Scientific Advisory Committee on Nutrition (2016), are considered more accurate. It would be preferable to analyze the association between vitamin D status and biological aging using these updated classifications. While sun exposure and dietary intake are primary sources of vitamin D, this information was not included in our analysis due to limitations in the NHANES dataset. Additionally, race significantly influences serum vitamin D levels, but the definition of race varies across NHANES data collected over different years, which posed a challenge in our study. Finally, emerging evidence questions the traditional view of serum 25(OH)D as a definitive biomarker of vitamin D status, although it remains the most widely accepted marker. For instance, in individuals with diabetes or obesity, the expression of CYP2R1—the enzyme responsible for vitamin D 25-hydroxylation—is suppressed, leading to decreased 25(OH)D levels despite stable overall vitamin D levels~~The current study has several limitations. First, this cross-sectional study only provides information on the association of 25(OH)D serum level and biological aging, making it difficult to establish causal relationships. Secondly, the PhenoAge algorithm we applied did not contain CRP data. This could be a limitation given that CRP is a key marker of inflammation and is potentially closely associated with vitamin D status. The absence of CRP in the PhenoAge calculation might lead to underestimation of the true relationship between vitamin D status and age acceleration. To gain a more accurate understanding of this association, future studies with more comprehensive datasets should be conducted. Moreover, recent evidence challenge the traditional view that serum 25(OH)D is treated as a reliable biomarker of vitamin D, though it remains the most accepted marker of vitamin D status. For example, in individuals diagnosed with diabetes or obesity, the expression of CYP2R1, the enzyme responsible for vitamin D 25-hydroxylation, are suppressed, resulting in the decrease of 25(OH)D while vitamin~~

Formatted: Font color: Accent 1

~~D level is stable~~ [47, 48]. ~~Finally, the gender specific differences in results require further detailed analysis to fully understand the underlying mechanisms.~~

Field Code Changed

5. Conclusion

In this study, we investigated the relationship between 25(OH)D serum levels and PhenoAge~~biological age~~ in NHANES, and found that there was a negative association between PhenoAgeAccel~~biological age~~ and 25(OH)D serum level, and these associations were nonlinear in both genders, revealing a U-shaped curve for males and an L-shaped curve for females. Low 25(OH)D serum levels shows a potential positively link to ~~-~~PhenoAgeAccel ~~accelerated biological aging~~ in both genders, but high 25(OH)D serum levels do not ~~correlate~~ associate with decelerated PhenoAge~~biological aging~~, and even associated with accelerated PhenoAge~~biological aging~~ in male. Our results suggest that the association between vitamin D levels with biological aging is different in men and women.

Authors contributions

Yi Feng: Conceptualization; Methodology; Data curation; Formal analysis; Writing original draft**; Licheng Yu**: Data curation; Formal analysis; **Jue Wang**: Project administration; Supervision; Writing - review &editing.

Conflicts of interest

The authors declare no competing financial or non-financial interests related to this work. NHANES is a publicly available dataset managed by the Centers for Disease Control and Prevention. The authors confirm that there is no affiliation with or involvement from the NHANES program administrators in this study. The funding source had no role in study design, data collection, analysis, or manuscript preparation.

References

1. Bikle DD. Vitamin D metabolism, mechanism of action, and clinical applications. Chem Biol. 2014;21(3):319-29. Epub 2014/02/18. doi: 10.1016/j.chembiol.2013.12.016. PubMed PMID: 24529992; PubMed Central PMCID: PMCPMC3968073.
2. Dominguez LJ, Farruggia M, Veronese N, Barbagallo M. Vitamin D Sources, Metabolism, and Deficiency: Available Compounds and Guidelines for Its Treatment. Metabolites. 2021;11(4). Epub 2021/05/01. doi: 10.3390/metabo11040255. PubMed PMID: 33924215; PubMed Central PMCID: PMCPMC8074587.
3. Couchman L, Moniz CF. Analytical considerations for the biochemical assessment of vitamin D status. Ther Adv Musculoskelet Dis. 2017;9(4):97-104. Epub 2017/04/07. doi: 10.1177/1759720X17692500. PubMed PMID: 28382113; PubMed Central PMCID: PMCPMC5367642.
4. Rahman A, Elmi A. Air pollutants are negatively associated with vitamin D-synthesizing UVB radiation intensity on the ground. Sci Rep. 2021;11(1):21480. Epub 2021/11/04. doi: 10.1038/s41598-021-00980-6. PubMed PMID: 34728744; PubMed Central PMCID: PMCPMC8563978.
5. Zhao S, Qian F, Wan Z, Chen X, Pan A, Liu G. Vitamin D and major chronic diseases. Trends Endocrinol Metab. 2024. Epub 2024/06/02. doi: 10.1016/j.tem.2024.04.018. PubMed PMID: 38824035.
6. Gaksch M, Jorde R, Grimnes G, Joakimsen R, Schirmer H, Wilsgaard T, et al. Vitamin D and mortality: Individual participant data meta-analysis of standardized 25-hydroxyvitamin D in 26916 individuals from a European consortium. Plos One. 2017;12(2):e0170791. Epub 2017/02/17. doi: 10.1371/journal.pone.0170791. PubMed PMID: 28207791; PubMed Central PMCID: PMCPMC5312926.
7. Fang A, Zhao Y, Yang P, Zhang X, Giovannucci EL. Vitamin D and human health: evidence from Mendelian randomization studies. Eur J Epidemiol. 2024;39(5):467-90. Epub 2024/01/12. doi: 10.1007/s10654-023-01075-4. PubMed PMID: 38214845.
8. Cui A, Xiao P, Ma Y, Fan Z, Zhou F, Zheng J, et al. Prevalence, trend, and predictor analyses of vitamin D deficiency in the US population, 2001-2018. Front Nutr. 2022;9:965376. Epub 2022/10/21. doi: 10.3389/fnut.2022.965376. PubMed PMID: 36263304; PubMed Central PMCID: PMCPMC9573946.
9. Berridge MJ. Vitamin D deficiency accelerates ageing and age-related diseases: a novel hypothesis. The Journal of physiology. 2017;595(22):6825-36. Epub 2017/09/28. doi: 10.1113/JP274887. PubMed PMID: 28949008; PubMed Central PMCID: PMCPMC5685827.
10. Zittermann A, Ernst JB, Prokop S, Fuchs U, Dreier J, Kuhn J, et al. Effect of vitamin D on all-cause mortality in heart failure (EVITA): a 3-year randomized clinical trial with 4000 IU vitamin D daily. Eur Heart J. 2017;38(29):2279-86. Epub 2017/05/13. doi: 10.1093/eurheartj/ehx235. PubMed PMID: 28498942.
11. Scragg R, Stewart AW, Waayer D, Lawes CMM, Toop L, Sluyter J, et al. Effect of Monthly High-Dose Vitamin D Supplementation on Cardiovascular Disease in the Vitamin D Assessment Study : A Randomized Clinical Trial. JAMA Cardiol. 2017;2(6):608-16. Epub 2017/04/07. doi: 10.1001/jamacardio.2017.0175. PubMed PMID: 28384800; PubMed Central PMCID: PMCPMC5815022 ICMJE Form of Disclosure of Potential Conflicts of Interest and none were reported.
12. Xia X, Wang Y, Yu Z, Chen J, Han JJ. Assessing the rate of aging to monitor aging itself. Ageing Res Rev. 2021;69:101350. Epub 2021/05/04. doi: 10.1016/j.arr.2021.101350. PubMed PMID: 33940202.
13. Jylhava J, Pedersen NL, Hagg S. Biological Age Predictors. EBioMedicine. 2017;21:29-36. Epub 2017/04/12. doi: 10.1016/j.ebiom.2017.03.046. PubMed PMID: 28396265; PubMed Central PMCID:

Field Code Changed

Formatted: Font: (Default) Times New Roman

PMCPMC5514388.
14. Bischoff-Ferrari HA, Gangler S, Wieczorek M, Belsky DW, Ryan J, Kressig RW, et al. Individual and additive effects of vitamin D, omega-3 and exercise on DNA methylation clocks of biological aging in older adults from the DO-HEALTH trial. Nat Aging. 2025;5(3):376-85. Epub 2025/02/04. doi: 10.1038/s43587-024-00793-y. PubMed PMID: 39900648.
15. Kwon D, Belsky DW. A toolkit for quantification of biological age from blood chemistry and organ function test data: BioAge. Geroscience. 2021;43(6):2795-808. Epub 2021/11/03. doi: 10.1007/s11357-021-00480-5. PubMed PMID: 34725754; PubMed Central PMCID: PMCPMC8602613.
16. Centers for Disease Control and Prevention National Health and Nutrition Examination Survey Questionnaires D, and Related Documentation 2020. [cited 2025 March 7]. Available from: https://wwwn.cdc.gov/nchs/nhanes/.
17. Ross AC, Taylor CL, Yaktine AL, Del Valle HB. Dietary Reference Intakes for Calcium and Vitamin D. The National Academies Collection: Reports funded by National Institutes of Health. Washington (DC)2011.
18. Liu Z, Kuo PL, Horvath S, Crimmins E, Ferrucci L, Levine M. A new aging measure captures morbidity and mortality risk across diverse subpopulations from NHANES IV: A cohort study. PLoS Med. 2018;15(12):e1002718. Epub 2019/01/01. doi: 10.1371/journal.pmed.1002718. PubMed PMID: 30596641; PubMed Central PMCID: PMCPMC6312200.
19. Liu W, Wang J, Wang M, Hou H, Ding X, Ma L, et al. Oxidative Stress Factors Mediate the Association Between Life's Essential 8 and Accelerated Phenotypic Aging: NHANES 2005-2018. J Gerontol A Biol Sci Med Sci. 2024;79(1). Epub 2023/10/10. doi: 10.1093/gerona/glad240. PubMed PMID: 37813096.
20. He H, Chen X, Ding Y, Chen X, He X. Composite dietary antioxidant index associated with delayed biological aging: a population-based study. Aging (Albany NY). 2024;16(1):15-27. Epub 2024/01/04. doi: 10.18632/aging.205232. PubMed PMID: 38170244; PubMed Central PMCID: PMCPMC10817368.
21. Haskell WL, Lee IM, Pate RR, Powell KE, Blair SN, Franklin BA, et al. Physical activity and public health: updated recommendation for adults from the American College of Sports Medicine and the American Heart Association. Circulation. 2007;116(9):1081-93. Epub 2007/08/03. doi: 10.1161/CIRCULATIONAHA.107.185649. PubMed PMID: 17671237.
22. Bender R. Introduction to the use of regression models in epidemiology. Cancer epidemiology: Springer; 2009. p. 179-95.
23. Hastie TJ. Generalized additive models. Statistical models in S. 2017:249-307.
24. Muggeo VM. Estimating regression models with unknown break-points. Stat Med. 2003;22(19):3055-71. Epub 2003/09/16. doi: 10.1002/sim.1545. PubMed PMID: 12973787.
25. Wood SN. Stable and efficient multiple smoothing parameter estimation for generalized additive models. Journal of the American Statistical Association. 2004;99(467):673-86.
26. World Medical A. World Medical Association Declaration of Helsinki: Ethical Principles for Medical Research Involving Human Participants. JAMA. 2025;333(1):71-4. Epub 2024/10/19 20:42. doi: 10.1001/jama.2024.21972. PubMed PMID: 39425955.
27. Schottker B, Hagen L, Zhang Y, Gao X, Holleczek B, Gao X, et al. Serum 25-Hydroxyvitamin D Levels as an Aging Marker: Strong Associations With Age and All-Cause Mortality Independent From Telomere Length, Epigenetic Age Acceleration, and 8-Isoprostane Levels. J Gerontol A Biol Sci Med Sci. 2019;74(1):121-8. Epub 2018/10/30. doi: 10.1093/gerona/gly253. PubMed PMID: 30371905.
28. Liu L, Chen C, Lo K, Huang J, Yu Y, Huang Y, et al. Serum 25-hydroxyvitamin D, frailty, and

mortality among the Chinese oldest old: Results from the CLHLS study. Nutr Metab Cardiovasc Dis. 2021;31(9):2707-15. Epub 2021/08/06. doi: 10.1016/j.numecd.2021.05.033. PubMed PMID: 34348876.

29. Veldurthy V, Wei R, Oz L, Dhawan P, Jeon YH, Christakos S. Vitamin D, calcium homeostasis and aging. Bone Res. 2016;4:16041. Epub 2016/10/30. doi: 10.1038/boneres.2016.41. PubMed PMID: 27790378; PubMed Central PMCID: PMCPMC5068478.

30. Ge D, Chen Q, Xie X, Li Q, Yang Y. Unveiling the potent effect of vitamin D: harnessing Nrf2/HO-1 signaling pathways as molecular targets to alleviate urban particulate matter-induced asthma inflammation. BMC Pulm Med. 2024;24(1):55. Epub 2024/01/26. doi: 10.1186/s12890-024-02869-2. PubMed PMID: 38273268; PubMed Central PMCID: PMCPMC10809564.

31. Pilz S, Gaksch M, Kienreich K, Grubler M, Verheyen N, Fahrleitner-Pammer A, et al. Effects of vitamin D on blood pressure and cardiovascular risk factors: a randomized controlled trial. Hypertension. 2015;65(6):1195-201. Epub 2015/03/25. doi: 10.1161/HYPERTENSIONAHA.115.05319. PubMed PMID: 25801871.

32. Chowdhury R, Kunutsor S, Vitezova A, Oliver-Williams C, Chowdhury S, Kiefte-de-Jong JC, et al. Vitamin D and risk of cause specific death: systematic review and meta-analysis of observational cohort and randomised intervention studies. BMJ. 2014;348:g1903. Epub 2014/04/03. doi: 10.1136/bmj.g1903. PubMed PMID: 24690623; PubMed Central PMCID: PMCPMC3972416 at www.icmje.org/coi_disclosure.pdf (available on request from the corresponding authors) and declare: no support from any organisation for the submitted work; no financial relationships with any organisations that might have an interest in the submitted work in the previous three years; no other relationships or activities that could appear to have influenced the submitted work..

33. Rejnmark L, Bislev LS, Cashman KD, Eiriksdottir G, Gaksch M, Grubler M, et al. Non-skeletal health effects of vitamin D supplementation: A systematic review on findings from meta-analyses summarizing trial data. Plos One. 2017;12(7):e0180512. Epub 2017/07/08. doi: 10.1371/journal.pone.0180512. PubMed PMID: 28686645; PubMed Central PMCID: PMCPMC5501555.

34. LeBoff MS, Chou SH, Ratliff KA, Cook NR, Khurana B, Kim E, et al. Supplemental Vitamin D and Incident Fractures in Midlife and Older Adults. N Engl J Med. 2022;387(4):299-309. Epub 2022/08/09. doi: 10.1056/NEJMoa2202106. PubMed PMID: 35939577; PubMed Central PMCID: PMCPMC9716639.

35. Pludowski P, Grant WB, Karras SN, Zittermann A, Pilz S. Vitamin D Supplementation: A Review of the Evidence Arguing for a Daily Dose of 2000 International Units (50 microg) of Vitamin D for Adults in the General Population. Nutrients. 2024;16(3). Epub 2024/02/10. doi: 10.3390/nu16030391. PubMed PMID: 38337676; PubMed Central PMCID: PMCPMC10857599.

36. Marcinowska-Suchowierska E, Kupisz-Urbanska M, Lukaszkiewicz J, Pludowski P, Jones G. Vitamin D Toxicity-A Clinical Perspective. Front Endocrinol (Lausanne). 2018;9:550. Epub 2018/10/09. doi: 10.3389/fendo.2018.00550. PubMed PMID: 30294301; PubMed Central PMCID: PMCPMC6158375.

37. Batman A, Saygili ES, Yildiz D, Sen EC, Erol RS, Canat MM, et al. Risk of hypercalcemia in patients with very high serum 25-OH vitamin D levels. Int J Clin Pract. 2021;75(7):e14181. Epub 2021/03/25. doi: 10.1111/ijcp.14181. PubMed PMID: 33759301; PubMed Central PMCID: PMCPMC8250214.

38. Sajid U, Orton D, Kaufmann M, Jones G, Kline GA. Understanding Elevated Vitamin D Measurements to Uncover Hypercalcemia Etiology. Clin Chem. 2024;70(6):798-802. Epub 2024/06/03.

Formatted: Font: (Default) Times New Roman

Formatted: Font: (Default) Times New Roman

doi: 10.1093/clinchem/hvae044. PubMed PMID: 38825340.
39. England PH. SACN vitamin D and health report 2016 [updated 2025-04-22]. Available from: https://www.gov.uk/government/publications/sacn-vitamin-d-and-health-report.
40. Turck D, Bresson J, Burlingame B. Scientific opinion on dietary reference values for vitamin D EFSA Panel on Dietetic Products, Nutrition, and Allergies (NDA). EFSA J. 2016;179.
41. Holick MF, Binkley NC, Bischoff-Ferrari HA, Gordon CM, Hanley DA, Heaney RP, et al. Evaluation, treatment, and prevention of vitamin D deficiency: an Endocrine Society clinical practice guideline. J Clin Endocrinol Metab. 2011;96(7):1911-30. Epub 2011/06/08. doi: 10.1210/jc.2011-0385. PubMed PMID: 21646368.
42. Roth DE, Abrams SA, Aloia J, Bergeron G, Bourassa MW, Brown KH, et al. Global prevalence and disease burden of vitamin D deficiency: a roadmap for action in low- and middle-income countries. Ann N Y Acad Sci. 2018;1430(1):44-79. Epub 2018/09/19. doi: 10.1111/nyas.13968. PubMed PMID: 30225965; PubMed Central PMCID: PMCPMC7309365.
43. Demay MB, Pittas AG, Bikle DD, Diab DL, Kiely ME, Lazaretti-Castro M, et al. Vitamin D for the Prevention of Disease: An Endocrine Society Clinical Practice Guideline. J Clin Endocrinol Metab. 2024. Epub 2024/06/03. doi: 10.1210/clinem/dgae290. PubMed PMID: 38828931.
44. Santoro N, Roeca C, Peters BA, Neal-Perry G. The Menopause Transition: Signs, Symptoms, and Management Options. J Clin Endocrinol Metab. 2021;106(1):1-15. Epub 2020/10/24. doi: 10.1210/clinem/dgaa764. PubMed PMID: 33095879.
45. Crescioli C. The Role of Estrogens and Vitamin D in Cardiomyocyte Protection: A Female Perspective. Biomolecules. 2021;11(12). Epub 2021/12/25. doi: 10.3390/biom11121815. PubMed PMID: 34944459; PubMed Central PMCID: PMCPMC8699224.
46. McCurdy R, Jiang X, Clarkson TB, Nudy M, Schnatz PF. Vitamin D and conjugated equine estrogen: the association with coronary artery atherosclerosis in cynomolgus monkeys. Menopause. 2016;23(5):481-7. Epub 2016/02/18. doi: 10.1097/GME.0000000000000582. PubMed PMID: 26886883.
47. Elkhwanky MS, Kummu O, Piltonen TT, Laru J, Morin-Papunen L, Mutikainen M, et al. Obesity Represses CYP2R1, the Vitamin D 25-Hydroxylase, in the Liver and Extrahepatic Tissues. JBMR Plus. 2020;4(11):e10397. Epub 2020/11/20. doi: 10.1002/jbm4.10397. PubMed PMID: 33210060; PubMed Central PMCID: PMCPMC7657391.
48. Aatsinki SM, Elkhwanky MS, Kummu O, Karpale M, Buler M, Viitala P, et al. Fasting-Induced Transcription Factors Repress Vitamin D Bioactivation, a Mechanism for Vitamin D Deficiency in Diabetes. Diabetes. 2019;68(5):918-31. Epub 2019/03/06. doi: 10.2337/db18-1050. PubMed PMID: 30833469; PubMed Central PMCID: PMCPMC6477896.

Table 1. Characteristics of the study population

Characteristics	Male (n=6836)	Female (n=7074)	*P*-value
Age (mean±SD)	57.07 (11.34)	58.12 (11.82)	<0.001
Race			0.001[b]
Non-Hispanic white	3249 (47.53)	3288 (46.48)	
Non-Hispanic black	1360 (19.89)	1417 (20.03)	
Others ()	2227 (32.58)	2369 (33.49)	
Education level			0.394[b]
Below high school	1847 (27.02)	1831 (25.88)	
High school or equivalent	1588 (23.23)	1597 (22.58)	
Above high school	3401 (49.75)	3646 (51.54)	
Poverty-income ratio			<0.001[b]
≤1.0	1199 (17.54)	1447 (20.46)	
1.0-3.0	2839 (41.53)	3029 (42.82)	
>3.0	2798 (40.93)	2598 (36.73)	
BMI, kg/m^2			<0.001[b]
<25	1585 (23.19)	1841 (26.02)	
25-30	2749 (40.21)	2117 (29.93)	
≥30	2502 (36.60)	3116 (44.05)	
Smoking			<0.001[b]
Never smoker	2823 (41.30)	4297 (60.74)	
Previous smoker	2570 (37.60)	1666 (23.55)	
Current smoker	1443 (21.11)	1111 (15.71)	
Drinking (alcohol consumption)			<0.001[b]
Never drinker	527 (7.71)	1572 (22.22)	
Previous drinker	637 (9.32)	1466 (20.72)	
Current drinker	5672 (82.97)	4036 (57.05)	
Level of physical activity			<0.001[b]
Inactive	1775 (25.97)	2533 (35.81)	
Insufficient	802 (11.73)	1086 (15.35)	
Moderate	680 (9.95)	842 (11.90)	
High	3579 (52.36)	2613 (36.94)	
Sleep duration			<0.001[b]
<7 h	2572 (37.62)	2445 (34.56)	
7-8h	1858 (27.18)	1812 (25.61)	
≥8 h	2406 (35.20)	2817 (39.82)	
Hypertension			0.839[b]

No	3007 (43.99)	3067 (43.36)	
Yes	3829 (56.01)	4007 (56.64)	
Diabetes mellitus~~Diabetes~~			<0.001[b]
No	5086 (74.40)	5515 (77.96)	
Yes	1750 (25.60)	1559 (22.04)	
Serum 25(OH)D, nmol/L			
Median (IQR)	63.80 (48.38, 80.20)	66.60 (47.00, 87.30)	<0.001[c]
<30	420 (6.14)	545 (7.70)	<0.001[b]
30-50	1451 (21.23)	1457 (20.60)	
50-75	2766 (40.46)	2293 (32.41)	
⩾75	2199 (32.17)	2779 (39.28)	
~~PhenoAge~~PhenoAge (median [IQR])	53.77 (45.07, 64.60)	53.05 (44.23, 64.13)	0.032
~~PhenoAge~~PhenoAgeAccel (median [IQR])	-2.40 (-5.13, 0.94)	-4.14 (-7.22, -0.46)	<0.001[c]
Standardized ~~PhenoAge~~PhenoAgeAccel (mean±SD)	0.11 (0.95)	-0.16 (1.05)	<0.001[a]

[a] Student's t-test was used to compare means of variables between two groups

[b] Chi-square test was used to compare categorical variables between two groups.

[c] Mann-Whitney test was used to compare non-normal variables between two groups.

Table 2. Association between 25(OH)D serum level and PhenoAgeAccel~~biological aging~~

Characteristics	All			Male			Female		
	Beta coefficient (95% CI)		*P*-value	Beta coefficient (95% CI)		*P*-value	Beta coefficient (95% CI)		*P*-value
	Crude model	Adjusted model [a]		Crude model	Adjusted model [a]		Crude model	Adjusted model [a]	
Log(25(OH)D)	-0.27 (-0.32, -0.22)	-0.04 (-0.08, 0.00)	0.039	-0.13 (-0.20, -0.06)	0.03 (-0.02, 0.09)	0.334	-0.32 (-0.38, -0.26)	-0.07 (-0.12, -0.01)	0.007
Quartiles									
Q1	Reference	Reference		Reference	Reference		Reference	Reference	
Q2	-0.23 (-0.29, -0.17)	-0.10 (-0.16, -0.05)	<0.001	-0.16 (-0.24, -0.08)	-0.02 (-0.09, 0.04)	0.448	-0.33 (-0.41, -0.25)	-0.16 (-0.23, -0.08)	<0.001
Q3	-0.29 (-0.36, -0.23)	-0.10 (-0.16, -0.04)	0.001	-0.20 (-0.29, -0.11)	-0.03 (-0.12, 0.04)	0.355	-0.40 (-0.49, -0.32)	-0.15 (-0.22, -0.07)	<0.001
Q4	-0.32 (-0.38, -0.26)	-0.05 (-0.11, -0.00)	0.022	-0.16 (-0.24, -0.07)	0.02 (-0.04, 0.10)	0.553	-0.42 (-0.51, -0.34)	-0.10 (-0.18, -0.02)	0.009
P for trend			0.173			0.429			0.076
Categories based on the 2011 Institute of Medicine~~Institute of Medicine guideline~~									
<30	Reference	Reference		Reference	Reference		Reference	Reference	
30-50	-0.23 (-0.33, -0.13)	-0.14 (-0.23, -0.05)	0.002	-0.26 (-0.41, -0.11)	-0.15 (-0.29, -0.02)	0.024	-0.22 (-0.34, -0.09)	-0.10 (-0.21, 0.00)	0.057
50-75	-0.42 (-0.52, -0.33)	-0.21 (-0.30, -0.13)	<0.001	-0.39 (-0.53, -0.25)	-0.16 (-0.29, -0.04)	0.009	-0.51 (-0.63, -0.40)	-0.24 (-0.33, -0.15)	<0.001
⩾75	-0.46 (-0.56, -0.36)	-0.15 (-0.24, -0.06)	0.001	-0.36 (-0.50, -0.22)	-0.09 (-0.22, 0.03)	0.131	-0.54 (-0.66, -0.42)	-0.17 (-0.27, -0.07)	0.001
P for trend			0.130			0.366			0.014

[a] Adjusted for age, race, education level, poverty-income ratio, body mass index, smoking, drinking, physical activity, sleep duration,

hypertension, diabetes and season of measurement.

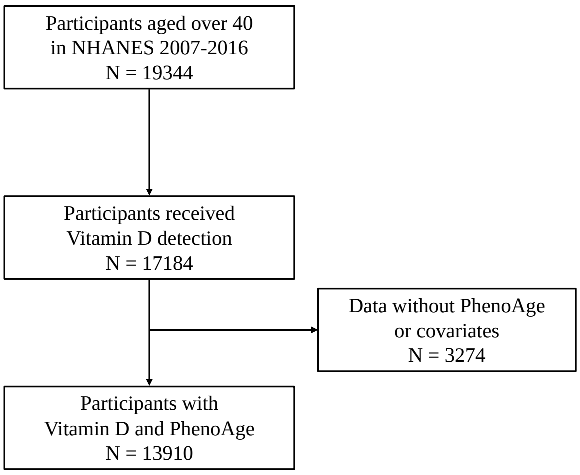

Figure 1 Flowchart of participants selection.

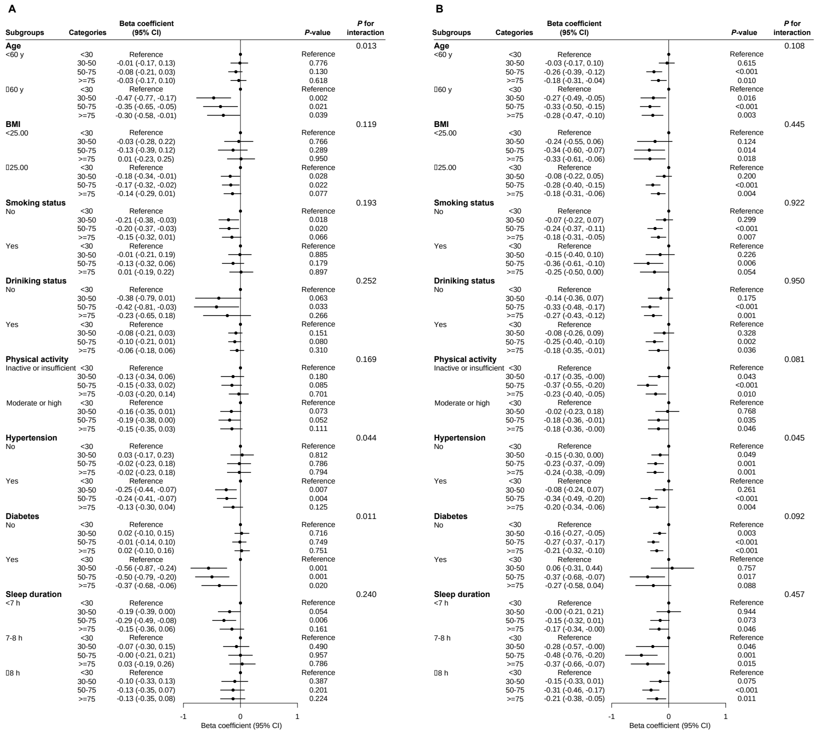

Figure 2 Subgroup analyses of the association between 25(OH)D categories (based on the 2011 Institute of Medicine) and PhenoAgeAccel~~biological aging~~ among males (A) and females (B). The filled circles and horizontal lines represent regression coefficients and the 95% confidence intervals. Multiple linear regression models were adjusted for age, race, education level, poverty-income ratio, body mass index, smoking, drinking, physical activity, sleep duration, hypertension, diabetes and season ~~of measurement~~. BMI, body mass index; CI, confidence intervals.

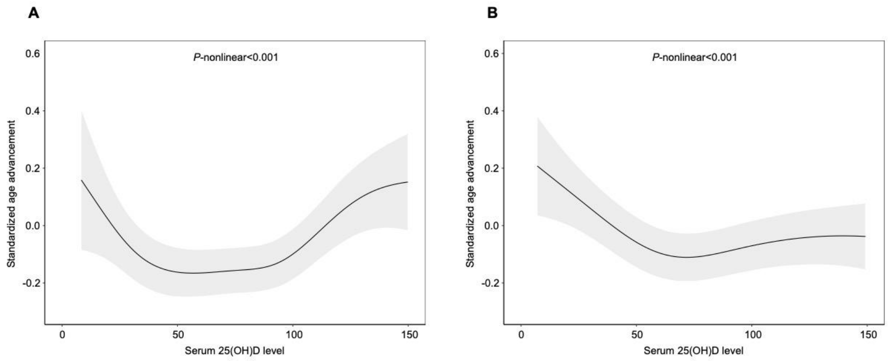

Figure 3 Nonlinear relationships between 25(OH)D serum level and PhenoAgeAccel biological aging among males (A) and females (B). Generalized additive models were adjusted for age, race, education level, poverty-income ratio, body mass index, smoking, drinking, physical activity, sleep duration, hypertension, diabetes and season of measurement. The black solid line represents the smooth curve fit between variables. The grey band represents the 95% confidence interval of the fit.

Table 3. Results of the analysis of the relationship between 25(OH)D serum levels and PhenoAgePhenoAge

25(OH)D serum levels (nmol/L)	Beta coefficient (95% CI)	*P*-value
Male		
<38.20	-0.013 (-0.025, -0.002)	0.017
38.20-91.55	0.001 (-0.001, 0.002)	0.487
⩾91.55	0.005 (0.002, 0.008)	<0.001
Female		
<62.50	-0.007 (-0.010, -0.004)	<0.001
62.50-125.00	0.002 (0.000, 0.004)	0.029
⩾125.00	-0.001 (-0.005, 0.002)	0.535

Generalized additive modelsCI: confidence intervals

Segmented regression models were adjusted for age, race, education level, poverty-income ratio, body mass index, smoking, drinking, physical activity, sleep duration, hypertension, diabetes and season of measurement.

Dear editor Mickael Essouma and reviewers

We sincerely thank the reviewers for their approval of our manuscript and the editor for the valuable and constructive suggestions, especially the meticulous word-for-word revisions. We have carefully studied all comments and incorporated them thoroughly into our revisions. We are very grateful for the editor's dedicated support and hope the revised manuscript is now satisfactory. Should you have any questions, please feel free to contact us. We appreciate your continued consideration.

1. Consider addressing the points 2, 3, 4 and 5 highlighted in the section "Additional Editor comments" of the Decision letter about manuscript R1 when discussing the study limitations in the discussion section.

Response：The limitations have been revised in the manuscript, and the concerns raised in the "Additional Editor comments," including the vitamin D classification and race definition, have been addressed. Thank you so much.

2. The first major reporting issue is the lack of clarity about the metrics of biological aging studied. Was it PhenoAge alone, PhenoAge and PhenoAgeAccel, or PhenoAgeAccel alone? This issue stems from the fact that the term PhenoAge is inconsistently used throughout the manuscript. In the same vein, it seems to me that developers of PhenoAge termed it "DNAm PhenoAge". So, even if many authors keep using the term "PhenoAge" rather than "DNAm PhenoAge", I would use the term "DNAm PhenoAge" and consequently also the term "DNAm PhenoAgeAccel" when talking about PHenoAge Acceleration throughout the manuscript. The choice of the term to use between "DNAm PhenoAge" and "PhenoAge" (and consequently also between "DNAm PhenoAgeAccel" and "PhenoAgeAccel") is up to you, the only requeirement in this regard is consistency with the term chosen.

Response： We have reviewed the entire manuscript and made corrections to use either "PhenoAge" or "PhenoAgeAccel" accordingly.

3. A second major issue is that the authors do not use the PhenoAge classification (positive versus negative) when reporting on the association between 25(OH)D serum levels and PhenoAgeAccel throughout the manuscript, although they clearly mentioned that classification in the Materials and Methods section. Rather, they kept using the expression "reduced PhenoAgeAccel" which would need to be replaced by "negative PhenoAgeAccel" or perhaps "Decreasing PhenoAGeAccel". Therefore, consider implementing the recognised PhenoAgeAccel classification in the results and discussion sections of the manuscript.

Response：A positive value of PhenoAgeAccel indicates an accelerated biological aging process, while a negative value indicates a slower biological aging process. We have revised "reduced/increased PhenoAgeAccel" to "negative/positive PhenoAgeAccel".

4. A third major issue (linked to the other major study variable: 25(OH)D serum level) is that the authors sticked to the 2011 IOM classification of 25(OH)D serum levels which includes four vitamin D serum level categories (sufficiency, insufficiency, deficiency and equal to or above 75 nmol/L) meanwhile they were advised to drop that classification given that the Endocrine Society no longer endorses that vitamin D classification status. I get it that the authors have the right to choose the classification system that best suits their needs. However, when doing so, they need to acknowledge in the limitations statement of the discussion section that the classification system used is now outdated and few evidence if any, currently to supports that classification system.

Response： We have added this information in the section of limitation. "the classification of 25(OH)D serum levels used in our study is based on the 2011 IOM guidelines; however, more recent classifications, such as those from the Scientific Advisory Committee on Nutrition (2016), are considered more accurate. It would be preferable to analyze the association between vitamin D status and biological aging using these updated classifications". Thank you very much.

5. In addition, 25(OH)D serum levels need to be expressed using either or both of the two units ng/mL and/or nmol/L, but the unit chosen should be consistently used throughout the manuscript. I advise against using the term "correlation" throughout the manuscript, given the lack of correlation analyses in the manuscript. More comments and suggested edits are available in the document PONE-D-24-25180_R2_Mickael Essouma comments and suggested edits.pdf attached to this decision letter.

Response：25(OH)D serum levels were expressed using nmol/L and we correct these through the whole manuscript.

In the revised manuscript, the term “correlation” has been replaced with “association”.

We have carefully reviewed all the comments in the document and accepted the suggested revisions. These suggestions have been very helpful in improving our manuscript. We are very grateful for the editor's support and revision. Thank you very much.
