# Supplementary material for: Association of 25(OH)D serum level with biological aging: A Cross-Sectional Study of 2007–2016 NHANES surveys
Source: PLoS One. 2025 Aug 6;20(8):e0328107. doi: 10.1371/journal.pone.0328107 (PMC12327616; doi:10.1371/journal.pone.0328107)
Supplement: Attachment — Submitted filename: PONE-D-24-25180_R4_reviewed by Mickael Essouma.pdf [file pone.0328107.s012.pdf]

PLOS ONE

Association of 25(OH)D serum level with biological aging: A Cross-Sectional Study of 2007-2016 NHANES surveys

--Manuscript Draft--

**Manuscript Number:**	PONE-D-24-25180R4
**Article Type:**	Research Article
**Full Title:**	Association of 25(OH)D serum level with biological aging: A Cross-Sectional Study of 2007-2016 NHANES surveys
**Short Title:**	aging and 25(OH)D serum level
**Corresponding Author:**	Jue Wang<br>nanhui new town community health service center<br>shanghai, CHINA
**Keywords:**	vitamin D; Serum 25(OH)D; Biological Aging; PhenoAge; nonlinear association
**Abstract:**	Vitamin D plays a crucial role in various aspects of human body, including aging, but chronological age may not accurately reflect the true biological aging status. Recently, PhenoAge has been developed to estimate an individual's biological age based on different biological and clinical measures. Therefore, we investigated the relationship between 25(OH)D serum levels and biological aging calculated by PhenoAge Acceleration (PhenoAgeAccel) using data from the 2007-2016 National Health and Nutrition Examination Survey (NHANES). 25(OH)D serum levels were negatively associated with PhenoAgeAccel (β=-0.04 standard deviation [SD]; 95% confidence interval [CI]: -0.08 to 0.00). This negative association was dose-dependent in females (β=-0.07 SD; 95% CI: -0.12 to 0.01), but not in males. Generalized additive models further revealed gender-specific non-linear patterns: a U-shape pattern in males but an L-shaped pattern in females. Using segmented regression to confirm inflection points, we observed that 25(OH)D serum levels were linked to reduced PhenoAgeAccel at levels below 38.2 nmol/L (15.3 ng/mL) in males (β=-0.013 SD; 95% CI: -0.025 to -0.002) and 62.5 nmol/L (25.0 ng/mL) in females (β=-0.007 SD; 95% CI: -0.01 to -0.004,). However, 25(OH)D serum levels above 125 nmol/L showed no association with PhenoAgeAccel in females (β=-0.001 SD; 95% CI: -0.005-0.002), while in males, elevated levels (>91.6 nmol/L) were associated with increased PhenoAgeAccel (β=0.005 SD; 95% CI: 0.002-0.008). Our findings indicate that vitamin D insufficiency has an inverse link with accelerated biological aging, and high levels of vitamin D in males accelerated biological aging as well, offering valuable insights into the relationship between vitamin D and biological aging.
**Order of Authors:**	Yi Feng
	Licheng Yu
	Jue Wang
**Opposed Reviewers:**	
**Response to Reviewers:**	Dear editor Mickael Essouma<br>We sincerely thank you for the valuable and constructive suggestions, especially the meticulous word-for-word revisions. We have carefully studied all comments and incorporated them thoroughly into our revisions. We are very grateful for the editor's dedicated support and hope the revised manuscript is now satisfactory. Should you have any questions, please feel free to contact us.<br><br>1. Please review your reference list to ensure that it is complete and correct. If you have cited papers that have been retracted, please include the rationale for doing so in the manuscript text, or remove these references and replace them with relevant current references. Any changes to the reference list should be mentioned in the rebuttal letter that accompanies your revised manuscript. If you need to cite a retracted article, indicate the article's retracted status in the References list and also include a citation and full reference for the retraction notice.<br>Response: We have checked the references one by one, and no retracted article was listed in the manuscript. [22-24] and [50] were added in the references.

Additional Editor Comments:

2. The authors have continued to improve upon their manuscript, but need to polish the presentation of the materials and methods section and further edit the manuscript to improve the language quality.
Response: Thank you for your valuable feedback. We appreciate your suggestion to enhance the presentation of the materials and methods section. We will carefully review and revise this section to improve clarity, organization, and detail, ensuring that the methodology is clearly described and easily reproducible. Additionally, we will conduct a thorough language review throughout the manuscript to improve the overall quality and readability. We are committed to refining our manuscript accordingly and believe these revisions will strengthen the clarity and impact of our study.

Materials and Methods section.
3. Lines 159-164: consider addressing my request to provide references supporting the classification made for sleep duration, hypertension and diabetes.
Response: We thank the editor for your constructive suggestions. We have supplemented the following references to support the classification regarding sleep duration, hypertension and diabetes.
22.Chaput JP, McNeil J, Despres JP, Bouchard C, Tremblay A. Seven to eight hours of sleep a night is associated with a lower prevalence of the metabolic syndrome and reduced overall cardiometabolic risk in adults. PLoS One. 2013;8(9):e72832. Epub 2013/09/17. doi: 10.1371/journal.pone.0072832. PubMed PMID: 24039808; PubMed Central PMCID: PMCPMC3764138.
23.Muntner P, Hardy ST, Fine LJ, Jaeger BC, Wozniak G, Levitan EB, et al. Trends in Blood Pressure Control Among US Adults With Hypertension, 1999-2000 to 2017-2018. JAMA. 2020;324(12):1190-200. Epub 2020/09/10. doi: 10.1001/jama.2020.14545. PubMed PMID: 32902588; PubMed Central PMCID: PMCPMC7489367
24.American Diabetes A. Diagnosis and classification of diabetes mellitus. Diabetes Care. 2014;37 Suppl 1:S81-90. Epub 2013/12/21. doi: 10.2337/dc14-S081. PubMed PMID: 24357215.

4. As commented in the last round of review, you need to clearly specify that you conducted effect modification analyses for the association between 25(OH)D serum levels and PhenoAgeAccel in the statistical analysis sub-section before making the comment currently available in line 179. These references may be helpful in this respect: Edwarsd JR. Methods for Integrating Moderation and Mediation: A General Analytical Framework Using Moderated Path Analysis. Psychological Methods 2007, Vol. 12, No. 1, 1–22 and Knol et al. Recommendations for presenting analyses of effect modification and interaction. International Journal of Epidemiology 2012;41:514–520 doi:10.1093/ije/dyr218IF: 6.4 Q1 .This statement appears in the abstract: "Generalized additive models further found 25(OH)D serum 25 levels were linked to negative PhenoAgeAccel at levels below 38.2 nmol/L (15.3 ng/mL) in males (β=-0.013; 95% CI: -0.025 to -0.002) and 62.5 nmol/L (25.0 ng/mL) in females (β=-0.007; 95% CI: -0.01 to -0.004,), revealing a U-shaped relationship observed in males but an L-shaped pattern in females. . However, 25(OH)D serum levels above 125 nmol/L were not associated with PhenoAgeAccel in females (β=-0.001; 95% CI: -0.005-0.002), while in males, elevated levels (>91.55 nmol/L) were associated with positive PhenoAgeAccel (β=0.005; 95% CI: 0.002-0.008).". As requested in the last round of review, consider specifying in the methods section of the main manuscript the reasons why you chose those cut-off values for serum 25-vitamin D while performing the corresponding statistical analyses.
Response: We thank the editor for your constructive suggestions. We have supplemented moderated regression analyses incorporating a multiplicative interaction term (25(OH)D × gender) to evaluate whether the relationship between vitamin D status and biological aging differs by gender. The results of these analyses revealed statistically significant gender-specific effects (p for interaction <0.001), indicating that the association between 25(OH)D and PhenoAgeAccel is moderated by gender. In the revised manuscript, we have supplemented the following sentences in the Methods section: “To evaluate potential effect modification by gender and other key covariates, subgroup analyses and interaction tests were conducted. Interaction term of serum

	25(OH)D and stratifying factor were included in the multivariable linear regression models to test for significance of interaction.”<br>In the Results section, we have supplemented the following sentences: “Interaction test confirmed that association between serum 25(OH)D and PhenoAgeAccel was modified by gender, regardless of whether 25(OH)D was modeled as a continuous variable or categorized using quartiles or IOM guidelines (all p for interaction <0.001).”<br>Regarding the cut-off values identified in the segmented regression analysis, these were determined based on data-driven statistical criteria. Specifically, we first used a generalized additive model (GAM) with a smooth term for serum 25(OH)D levels to flexibly characterize the non-linear relationship with PhenoAgeAccel, as GAMs effectively capture complex dose-response patterns without assuming parametric forms. The GAM revealed distinct non-linear associations by sex, prompting further investigation using segmented regression—a method designed to formally identify inflection points (thresholds) where the slope of the association changes directionally. For each gender, optimal breakpoints were selected via likelihood ratio tests comparing nested models, ensuring statistical rigor in threshold estimation. These thresholds were computationally derived rather than manually assigned. However, we recognized potential ambiguity in how these thresholds were presented. To clarify, we have revised the following sentences in the Abstract: “25(OH)D serum levels were negatively associated with PhenoAgeAccel (β=-0.04 standard deviation [SD]; 95% confidence interval [CI]: -0.08 to 0.00). This negative association was dose-dependent in females (β=-0.07 SD; 95% CI: -0.12 to 0.01), but not in males. Generalized additive models further revealed gender-specific non-linear patterns: a U-shape pattern in males but an L-shaped pattern in females. Using segmented regression to confirm inflection points, we observed that 25(OH)D serum levels were linked to reduced PhenoAgeAccel at levels below 38.2 nmol/L (15.3 ng/mL) in males (β=-0.013 SD; 95% CI: -0.025 to -0.002) and 62.5 nmol/L (25.0 ng/mL) in females (β=-0.007 SD; 95% CI: -0.01 to -0.004,). However, 25(OH)D serum levels above 125 nmol/L showed no association with PhenoAgeAccel in females (β=-0.001 SD; 95% CI: -0.005-0.002), while in males, elevated levels (>91.55 nmol/L) were associated with increased PhenoAgeAccel (β=0.005 SD; 95% CI: 0.002-0.008).” Additionally, we have polished the language in the Results section to improve clarity.<br>.<br>5. Results section. As commented in the last round of review, consider rounding all the numbers to one decimal place when providing results throughout the manuscript (Table 1 and so on).<br>Response: We have corrected all the numbers to one decimal place.<br><br>Comment appended to Table 1.<br>6. Discussion section: limitations statement. You also need to address the lack of data on vitamin D supplement among the confounders, the effect of unmeasured confounding and the reverse causation and residual confounding.<br>Response: This information has been added in section of limitation.<br><br>7. Comment about editing: Consider complying with PLOS ONE authors guidelines by not numbering the manuscript's sections and sub-sections.<br>Edits proposed in lines 123-125, 141-142, 178, 188, 251, 262,Table 1.<br>Response: We have made corrections following the editor’s suggestion for our manuscript. Thank you for your valuable feedback.
**Additional Information:**	
**Question**	**Response**
**Financial Disclosure**<br><br>Enter a financial disclosure statement that describes the sources of funding for the work included in this submission. Review the submission guidelines for detailed requirements. View published research articles from *PLOS ONE* for specific examples.	The author(s) received no specific funding for this work.

Powered by Editorial Manager® and ProduXion Manager® from Aries Systems Corporation

This statement is required for submission and **will appear in the published article** if the submission is accepted. Please make sure it is accurate.<br><br>**Funded studies**<br>Enter a statement with the following details:<br>• Initials of the authors who received each award<br>• Grant numbers awarded to each author<br>• The full name of each funder<br>• URL of each funder website<br>• Did the sponsors or funders play any role in the study design, data collection and analysis, decision to publish, or preparation of the manuscript?<br><br>Did you receive funding for this work?	
**Competing Interests**<br><br>Use the instructions below to enter a competing interest statement for this submission. On behalf of all authors, disclose any competing interests that could be perceived to bias this work—acknowledging all financial support and any other relevant financial or non-financial competing interests.<br><br>This statement is required for submission and **will appear in the published article** if the submission is accepted. Please make sure it is accurate and that any funding sources listed in your Funding Information later in the submission form are also declared in your Financial Disclosure statement.<br><br>View published research articles from *PLOS ONE* for specific examples.	The authors have declared that no competing interests exist.

**NO authors have competing interests**<br><br>Enter: *The authors have declared that no competing interests exist.*<br><br>**Authors with competing interests**<br><br>Enter competing interest details beginning with this statement:<br><br>*I have read the journal's policy and the authors of this manuscript have the following competing interests: [insert competing interests here]*<br><br>* typeset	
**Ethics Statement**<br><br>Enter an ethics statement for this submission. This statement is required if the study involved:<br><br>• Human participants<br>• Human specimens or tissue<br>• Vertebrate animals or cephalopods<br>• Vertebrate embryos or tissues<br>• Field research<br><br>Write "N/A" if the submission does not require an ethics statement.<br><br>General guidance is provided below. Consult the submission guidelines for detailed instructions. **Make sure that all information entered here is included in the Methods section of the manuscript.**	the study involves human participants. all data from NHANES, which is a publicly available dataset managed by the Centers for Disease Control and Prevention. The ethical approval of NHANES has been stated on the website. (https://wwwn.cdc.gov/nchs/nhanes/Default.aspx)

Powered by Editorial Manager® and ProduXion Manager® from Aries Systems Corporation

**Format for specific study types**<br><br>**Human Subject Research (involving human participants and/or tissue)**<br>• Give the name of the institutional review board or ethics committee that approved the study<br>• Include the approval number and/or a statement indicating approval of this research<br>• Indicate the form of consent obtained (written/oral) or the reason that consent was not obtained (e.g. the data were analyzed anonymously)<br><br>**Animal Research (involving vertebrate animals, embryos or tissues)**<br>• Provide the name of the Institutional Animal Care and Use Committee (IACUC) or other relevant ethics board that reviewed the study protocol, and indicate whether they approved this research or granted a formal waiver of ethical approval<br>• Include an approval number if one was obtained<br>• If the study involved *non-human primates*, add *additional details* about animal welfare and steps taken to ameliorate suffering<br>• If anesthesia, euthanasia, or any kind of animal sacrifice is part of the study, include briefly which substances and/or methods were applied<br><br>**Field Research**<br><br>Include the following details if this study involves the collection of plant, animal, or other materials from a natural setting:<br>• Field permit number<br>• Name of the institution or relevant body that granted permission	
**Data Availability**<br><br>Authors are required to make all data underlying the findings described fully available, without restriction, and from the time of publication. PLOS allows rare exceptions to address legal and ethical concerns. See the PLOS Data Policy and FAQ for detailed information.	Yes - all data are fully available without restriction

A Data Availability Statement describing where the data can be found is required at submission. Your answers to this question constitute the Data Availability Statement and **will be published in the article**, if accepted.<br><br>**Important:** Stating 'data available on request from the author' is not sufficient. If your data are only available upon request, select 'No' for the first question and explain your exceptional situation in the text box.<br><br>Do the authors confirm that all data underlying the findings described in their manuscript are fully available without restriction?	
**Describe where the data may be found in full sentences. If you are copying our sample text, replace any instances of XXX with the appropriate details.**<br><br>• If the data are **held or will be held in a public repository**, include URLs, accession numbers or DOIs. If this information will only be available after acceptance, indicate this by ticking the box below. For example: *All XXX files are available from the XXX database (accession number(s) XXX, XXX.*).<br>• If the data are all contained **within the manuscript and/or Supporting Information files**, enter the following: *All relevant data are within the manuscript and its Supporting Information files.*<br>• If neither of these applies but you are able to provide **details of access elsewhere**, with or without limitations, please do so. For example:<br><br>*Data cannot be shared publicly because of [XXX]. Data are available from the XXX Institutional Data Access / Ethics Committee (contact via XXX) for researchers who meet the criteria for access to confidential data.*<br><br>*The data underlying the results presented in the study are available from (include the name of the third party*	All files are available from the NHANES database

Powered by Editorial Manager® and ProduXion Manager® from Aries Systems Corporation

and contact information or URL).<br>• This text is appropriate if the data are owned by a third party and authors do not have permission to share the data.<br><br>* typeset	
Additional data availability information:	

Powered by Editorial Manager® and ProduXion Manager® from Aries Systems Corporation

Dear editor Mickael Essouma

We sincerely thank you for your valuable and constructive suggestions, especially the meticulous word-for-word revisions. We have carefully studied all comments and incorporated them thoroughly into our revisions. We are very grateful for the editor's dedicated support and hope the revised manuscript is now satisfactory. Should you have any questions, please feel free to contact us.

Yours sincerely

Corresponding author: Jue Wang

JUN, 2025

Manuscript

Click here to access/download;Manuscript;Manuscript-R4 20250623 clean.docx

Association of 25(OH)D serum level with biological aging: A Cross-Sectional Study of 2007-2016 NHANES surveys

Yi Feng[a]; Licheng Yu[b]; Jue Wang[a] *

[a] Nanhui new town Community Health Service Center, Pudong New Area, Shanghai, 201308, China.

[b] Prevention and Health Care Department, Heqing Community Health Service Center of Pudong New District, Shanghai, 201201, China.

Corresponding author. Jue Wang

E-mail address: wangjue180130@126.com

Abstract

Vitamin D plays a crucial role in various aspects of human body, including aging, but chronological age may not accurately reflect the true biological aging status. Recently, PhenoAge has been developed to estimate an individual's biological age based on different biological and clinical measures. Therefore, we investigated the relationship between 25(OH)D serum levels and biological aging calculated by PhenoAge Acceleration (PhenoAgeAccel) using data from the 2007-2016 National Health and Nutrition Examination Survey (NHANES). 25(OH)D serum levels were negatively associated with PhenoAgeAccel (β=-0.04 standard deviation [SD]; 95% confidence interval [CI]: -0.08 to 0.00). This negative association was dose-dependent in females (β=-0.07 SD; 95% CI: -0.12 to 0.01), but not in males. Generalized additive models further revealed gender-specific non-linear patterns: a U-shape pattern in males but an L-shaped pattern in females. Using segmented regression to confirm inflection points, we observed that 25(OH)D serum levels were linked to reduced PhenoAgeAccel at levels below 38.2 nmol/L (15.3 ng/mL) in males (β=-0.013 SD; 95% CI: -0.025 to -0.002) and 62.5 nmol/L (25.0 ng/mL) in females (β=-0.007 SD; 95% CI: -0.01 to -0.004,). However, 25(OH)D serum levels above 125 nmol/L showed no association with PhenoAgeAccel in females (β=-0.001 SD; 95% CI: -0.005-0.002), while in males, elevated levels (>91.6 nmol/L) were associated with increased PhenoAgeAccel (β=0.005 SD; 95% CI: 0.002-0.008). Our findings indicate that vitamin D insufficiency has an inverse link with accelerated biological aging, and high levels of vitamin D in males accelerated biological aging as well, offering valuable insights into the relationship between vitamin D and biological aging.

Key words: Vitamin D; Serum 25(OH)D; Biological aging; PhenoAge; Nonlinear association

Introduction

Vitamin D is a fat-soluble vitamin that plays a crucial role in human health, influencing various functions such as bone metabolism and immune regulation. It is primarily obtained through two sources: endogenous production (mainly vitamin D3) in the skin upon exposure to ultraviolet B (UVB) radiations, and exogenous intake (vitamin D2 and D3) through dietary sources, such as fatty fish, egg yolks or supplements. Once absorbed in the body, vitamin D is converted into 25-hydroxyvitamin D (25(OH)D) in the liver, then transformed into the biologically active form, 1α,25-dihydroxyvitamin D (1α,25$(OH)_2$D), in the kidneys [1]. This active form binds to the vitamin D receptor (VDR) to regulate calcium and phosphate metabolism [2]. Serum 25(OH)D is the most commonly measured form of vitamin D in humans because it is the major circulating form and provides an accurate indication of the body's overall vitamin D status [3]; and can be influenced by various factors including the geographic location, race and food intake [4].

Vitamin D deficiency is a global public health concern, associated with increased risk of type 2 diabetes (T2D), hypertension, multiple sclerosis [5-7]. It is especially prevalent among the elderly [8], with some studies suggesting that deficiency may accelerate chronological aging and contribute to age-related diseases and inflammatory diseases, such as cardiovascular disease, multiple sclerosis and rheumatoid arthritis [9]. However, several large randomized clinical trials of vitamin D supplementation failed to demonstrate significant benefits for cardiovascular disease, casting doubt on the evidence from observational studies linking vitamin D deficiency with cardiovascular disease [10, 11]. This ambiguity highlights challenges in assessing the association between vitamin D and aging. One major limitation of previous studies is that they relied on chronological age which does not always reflect an individual's biological age. Indeed, unlike chronological age which simply counts the years lived, biomarkers making up the biological age are mainly determined by a combination of genetic, environmental and lifestyle factors [12]. Consequently, some individuals may age biologically faster or slower than indicated by their chronological age.

To better characterize physiological aging in epidemiological studies, several aging metrics have been developed that combine clinical markers (e.g., PhenoAge and GrimAge) and biological (PhenoAge acceleration) markers [13]. In a post hoc analysis of the European DO-HEALTH randomized clinical trial involving 777 participants with a mean 25(OH)D serum level of 56.8 nmol/L, PhenoAge was the only biological age metric shown to be slowed by daily vitamin D (2000 UI) supplementation among the four biological age metrics that were assessed: PhenoAge, GrimAge, GrimAge2 and DunedinPACE [14]. PhenoAgeAccel was developed and validated using machine learning methods on a National Health and Nutrition Examination Survey (NHANES) dataset of 4000 adults aged 20 to 84 years, demonstrating a mean absolute error of approximately 3.6 years when predicting chronological age [15]. Although vitamin D supplementation has been shown to slow the increase of PhenoAgeAccel in older adults with low 25(OH)D serum concentrations, observational studies linking 25(OH)D serum concentrations with PhenoAgeAccel are limited. Therefore, this study aims to investigate whether there is a linear and/or non-linear relationship between 25(OH)D serum concentration and PhenoAgeAccel, using datasets of 2007-2016 NHANES surveys. By exploring this association, the study seeks to improve our understanding of the link between serum vitamin D levels and biological aging in human beings, with potential implications for public health.

Materials and Methods

Study design and population

This study was conducted and reported in agreement with STROBE guidelines for reporting observational studies. This study utilized data derived from the 2007-2016 NHANES conducted in the United States of America by the National Center for Health Statistics of the Centers for Disease Control and Prevention. All data included in the current study are publicly available on the NHANES website [16]. NHANES includes in-home interview, physical measurements and laboratory tests. The 2007-2016 NHANES datasets involved 50558 adult participants. Participants with incomplete

information and participants aged less than 40 years old were excluded. After applying the exclusion criteria, a total of 13,910 participants were finally included in this analysis (Figure 1).

Measurement of 25(OH)D serum level

Vitamin D serum levels were reflected by 25(OH)D. Because the forms of 25(OH)D measured in the 2007-2016 NHANES samples included both 25(OH)D2 and 25(OH)D3 (classic 25(OH)D3 and C3 epimer of 25(OH)D3 [C3-epi-25(OH)D3]), serum 25(OH)D included 25(OH)D2, 25(OH)D3 and C3-epi-25(OH)D3 in this study. During the study period (2007-2016), 25(OH)D levels were measured in the NHANES population using the liquid chromatography-tandem mass spectrometry (LC-MS/MS). According to the 2011 Institute of Medicine's Committee recommendations, we transformed serum 25(OH)D concentrations into a categorical variable with the following modalities: deficiency (<30 nmol/L), insufficiency (30–50 nmol/L), sufficiency (50–75 nmol/L) and abnormally high (≥75 nmol/L)[17].

Measurement of PhenoAge

In this study, we utilized PhenoAge as the indicator of biological aging, considering its high robustness and feasibility in NHANES data as a reliable predictor of mortality and age-related disease risks. The PhenoAgeAccel algorithm was constructed by applying elastic-net regression methods to NHANES III (1988-1994) datasets, encompasses 10 markers: serum albumin, serum creatinine, plasma glucose, white blood cell count, blood lymphocyte percent, red cell distribution width, mean red cell volume, serum C-reactive protein, serum alkaline phosphatase and chronological age [18]. All constituent biomarkers of PhenoAge were routinely measured during NHANES using standardized protocols ensuring reproducibility across study cycles. One of the markers, CRP, was unavailable from 2011 to 2018 datasets. However, previous studies have applied an algorithm that excluded CRP when calculating the PhenoAge, demonstrating good applicability [19, 20]. To assess the validity of

PhenoAge without CRP data, we calculated 2 PhenoAge estimates with and without C-reactive protein, using data from cycles 2007-2008 and 2009-2010, which contained all 10 markers. We found a strong association between 2 PhenoAge estimates with and without CRP (Pearson's correlation coefficient of 0.986). Based on this finding, we proceeded with the main analysis of data from the five NHANES cycles using the PhenoAge algorithm excluding CRP. We further calculated PhenoAgeAccel as the difference between PhenoAge and chronological age. A positive value of PhenoAgeAccel represents accelerated biological aging process, while a negative value represented slower biological aging process. Subsequently, PhenoAgeAccel was standardized to have a mean of 0 and a standard deviation (SD) of 1.

Covariates

NHANES interviews were conducted by trained interviewers. Questionnaires were administered to participants to collect information on demographics (chronological age, gender, race, education level, poverty income ratio (PIR) and lifestyle-related factors (tobacco smoking, alcohol consumption, physical activity, and comorbidities) and the body mass index (BMI). These variables were measured at the Mobile Examination Center.

Covariates in regression analyses testing the association between 25(OH)D serum levels and biological aging include age, race, education level, PIR, BMI, tobacco smoking, alcohol consumption, physical activity. The variable active tobacco smoking was classified into three categories: never smokers (previous smoking of a total of less than 100 cigarettes), current smokers (lifetime smoking of over 100 cigarettes) and former smokers (had smoked ⩾100 cigarettes in their lifetime but had quit smoking at the time of the survey). The variable alcohol consumption was classified as never drinkers (participants who had drunk <12 drinks in their lifetime), current drinkers (participants who had drunk ⩾12 drinks in their lifetime and at least one drink in the past year), and former drinkers (participants who had drunk ⩾12 drinks in their

lifetime but no drink in the past year). A Metabolic Equivalent task (MET)-min per week were calculated to assess physical activity, levels (in min per week) that were categorized as: inactive (no regular physical activity), insufficient (<500 MET-min activity/week), moderate (500-1,000 MET-min activity/week) and high (⩾1,000 MET-min activity/week) [21]. For sleep duration, participants responded to the question: “How much sleep do you usually get at night on weekdays or on workdays?” Sleep duration was categorized into 3 groups: <7 h, 7-8 h and ⩾8h [22]. The definition of hypertension was based on self-reports, the use of antihypertensive drugs or on the record of a systolic blood pressure⩾140 mmHg with or without a diastolic blood pressure⩾90 mmHg on physical exam [23]. The definition of diabetes mellitus was based on self-reports or on laboratory test results showing fasting blood glucose levels ⩾126 mg/dL or HbA1c values ⩾6.5% [24].

Statistical analysis

Weighted multivariable linear regression models were constructed to estimate the association between serum 25(OH)D status and PhenoAgeAccel. 25(OH)D serum levels were log-transformed due to their skewed distribution. To evaluate whether the association between 25(OH)D serum levels and PhenoAgeAccel was not just linear but also dose-dependent, 25(OH)D serum level was analyzed as both a continuous (log-transformed) and a categorical variable. In agreement with standards for linear regression analyses involving continuous dependent variables, the association between 25(OH)D serum levels as continuous variables and PhenoAgeAccel was estimated based on beta coefficients with their 95% confidence intervals [25]. All models were adjusted for chronological age, gender, race, education level, PIR, BMI, tobacco smoking and alcohol consumption variable, physical activity, sleep duration, hypertension, diabetes and ~~climate~~ of measurement. To evaluate potential effect modification by gender and other key covariates, subgroup analyses and interaction tests were conducted. Interaction term of serum 25(OH)D and stratifying factor were

included in the multivariable linear regression models to test for significance of interaction.

To explore whether there is also a non-linear relationship between 25(OH)D serum levels and PhenoAgeAccel, a Generalized Additive Model (GAM) was employed to fit the smooth curve. The GAMs extend traditional linear regression by replacing linear terms with smooth functions, allowing flexible modeling of complex dose-response relationships without assuming a priori parametric forms [26]. The threshold effect was further estimated using segmented regression model. This method identifies inflection points where the slope of the association between 25(OH)D serum levels and PhenoAgeAccel changes significantly. The optimal breakpoint was determined via likelihood ratio tests, comparing the goodness-of-fit between the non-segmented linear model and the segmented regression model [27]. Results with *P*-values <0.05 were considered to be statistically significance. All data analyses were performed using R software version 4.2.1 (R Core Team, Vienna, Austria). R package 'BioAge' was used to calculate PhenoAge [15], while the package 'mgcv' was used to conduct GAM analysis [28]. Continuous data were reported as means ± standard deviations (SDs) for normal distributions or as medians (interquartile ranges) for skewed distributions. Categorical data were reported as numbers (percentages).

Ethical considerations

This study utilized data from NHANES study, which was conducted in accordance with the principles in the Declaration of Helsinki. The NHANES protocol received prior ethical approval from the National Center for Health Statistics' Review Board. Since our study is a secondary analysis of publicly accessible, de-identified NHANES data sets, additional institutional review board approval was not required [29].

Results

Characteristics of the study population

The current study included a total of 6836 males and 7074 females, with the

characteristics of the study population presented in Table 1. Significant differences were observed between males and females in terms of age, race, BMI, and poverty-income ratio ($p < 0.001$). Additionally, gender-specific differences were noted in behaviors influencing biological aging, such as smoking, drinking, physical activity, and sleep duration ($p < 0.001$). Notably, males had a higher prevalence of diabetes mellitus than females. The median 25(OH)D serum level in females was 66.6 nmol/L compared with 63.8 nmol/L in males ($p<0.001$). The PhenoAgeAccel of males and females were -2.40 (-5.13, 0.94) and -4.14 (-7.22, -0.46) respectively, suggesting that PhenoAgeAccel was lower in females than in males.

Association between 25(OH)D serum level and biological aging

The associations between 25(OH)D serum levels and PhenoAgeAccel were presented in Table 2. After adjusting for age, race, education level, PIR, BMI, smoking, alcohol consumption, level of physical, sleep duration and presence of hypertension and/or diabetes mellitus, 25(OH)D serum levels were negatively associated with PhenoAgeAccel (β=-0.04 SD; 95% CI: -0.08 to 0.00). When stratified by sex, the association was statistically significant only in females ($p=0.007$). Further analysis dividing serum 25(OH)D into quartiles revealed an inverse relationship with PhenoAgeAccel, although no clear dose-response trend was observed (p for trend =0.173). When segregating the data by sex, 25(OH)D serum levels were inversely associated with PhenoAgeAccel only in women, with no apparent dose-response relationship (p for trend = 0.076). Categorizing 25(OH)D serum levels according to the 2011 USA institute of Medicine guidelines, inverse association with PhenoAgeAccel were observed for levels between 30-50 nmol/L and 50-75 nmol/L in males (without a clear dose-response trend), and between 50-75 nmol/L and ≥75 nmol/L in females, where a dose-response relationship was apparent ($p=0.014$). Interaction test confirmed that association between serum 25(OH)D and PhenoAgeAccel was modified by gender, regardless of whether 25(OH)D was modeled as a continuous variable or categorized using quartiles or IOM guidelines (all p for interaction <0.001).

Results of subgroup/effect modification analysis showed that in males higher 25(OH)D serum levels were associated with reduction in PhenoAgeAccel primarily among individuals aged over 60 years, those with hypertension and those with diabetes mellitus, suggesting that there were statistically significant interactions between 25(OH)D serum levels and age, hypertension, and diabetes (Fig.2A). In females, no effect modifiers were identified in the association between 25(OH)D serum levels and PhenoAge Accel (Fig.2B).

Association of 25(OH)D serum levels with PhenoAgeAccel on GAM

There was no linear association between serum 25(OH)D levels and PhenoAgeAccel when dividing the levels into quartiles (Table 2). Results from GAM confirmed a nonlinear association between 25(OH)D serum level and PhenoAgeAccel in both males and females ($p<0.001$) (Fig 3 A-B). In males, the relationship was U-shaped with the two inflection points at 38.2 nmol/L and 91.5 nmol/L. Specifically, low 25(OH)D serum levels (<38.2 nmol/L) were negatively related to PhenoAgeAccel (β=-0.013 SD; 95% CI: -0.025 to -0.002), whereas high levels (>91.55 nmol/L) showed a positive association (β=0.005 SD; 95% CI: 0.002 to 0.008). In females, segmented regression models indicated an L-shaped relationship. 25(OH)D serum levels below 62.5 nmol/L were negatively linked to PhenoAgeAccel (β=-0.007 SD; 95% CI: -0.01 to -0.004), but no significant association observed beyond 125 nmol/L (β=-0.001 SD; 95% CI: -0.005 to -0.002). Overall, low (<38.2 nmol/L) or high (>91.6 nmol/L) 25(OH)D serum levels in males were associated with PhenoAgeAccel, while in females, only low levels (<62.5 nmol/L) were associated with PhenoAgeAccel (Table 3).

Discussion

In the current study, the association between 25(OH)D serum level and biological aging was investigated in the 2007-2016 NHANES surveys. The analysis revealed a negative association between 25(OH)D serum levels and PhenoAgeAccel. Further effect modifier analysis showed that this association was influenced by age above 60

years, diabetes mellitus and hypertension, however, no such modifiers were identified for females. When subdividing 25(OH)D serum levels into quartiles and stratifying data by sex, this association was still observed only in females, and it exhibited a non-linear pattern. Additionally, when further subdividing 25(OH)D serum levels according to the Institute of Medicine in USA in 2011, the association persisted for 25(OH)D serum levels between 30-50 nmol/L and between 50-75 nmol/L in males and between 50-75 nmol/L and ≥75 nmol/L, with a linear pattern of relationship observed only in females. Non-linear associations were also found between 25(OH)D serum levels and PhenoAgeAccel in both males (U-shaped) and females (L-shaped).

Previous studies have reported that a gradual decline in 25(OH)D serum levels with chronological age, and vitamin D supplementation can mitigate oxidative damage and improve immune function in elder people, suggesting its potential role in slowing age-related physiological decline [30]. This decline in vitamin D levels is attributed to multiple factors, including decreased sun exposure, reduced capacity of the skin to synthesize vitamin D, and decreased dietary intake of vitamin D, all of which contribute to vitamin D deficiency in older adults [31]. The synthesis of vitamin D from sun exposure vary in different races, but our study lacks this data of sun exposure. Additionally, we also found that low 25(OH)D serum levels were associated with positive PhenoAgeAccel, suggesting vitamin D may play a crucial role in the regulation of the aging process. Adequate 25(OH)D levels are essential for maintaining calcium absorption and bone health preventing osteoporosis, a common age-related condition, and regulating calcium homeostasis and bone metabolism [32]. Beyond these functions, vitamin D can interact with antioxidant and anti-inflammatory pathways, such as activating Nrf2 (nuclear factor erythroid 2-related factor 2) and SIRT1 (sirtuin 1) signaling cascades, and suppressing the production of pro-inflammatory cytokines, including interleukin-6 (IL-6) and tumor necrosis factor-alpha (TNF-α), through inhibiting nuclear factor-κB (NF-κB). These actions help reduce inflammation and oxidative stress, both of which are known contributors to the aging process [32, 33]. Consequently, vitamin D and aging influence each other mutually.

Vitamin D supplementation has been shown to be an effective way to reverse or mitigate diseases, including lower blood pressure and improve endothelial function, leading to reduced risk of cardiovascular events, such as heart attacks and strokes, and improved cardiovascular outcomes [34]. A meta-analysis of 75 randomized controlled trials in 2014 reported that vitamin D supplementation was associated with a 6% reduction in all-cause mortality [35]. Notably, the impact of vitamin D supplementation on mortality appears more significant in certain subgroups, such as older adults or individuals with vitamin D deficiency. For instance, vitamin D supplementation was associated with a reduced risk of all-cause mortality in trials where a mean baseline 25(OH)D level were below 75 nmol/L (RR 0.89, 95% CI 0.80-0.99), but not in trials with a mean baseline 25(OH)D level $\geqslant$75 nmol/L (RR 1.04, 95% CI 0.97-1.11) [36]. Thus, these evidence highlights the potential of vitamin D supplementation in reducing the risk of several chronic diseases, particularly in the elderly, in whom serum vitamin D levels tend to decline with age.

Despite its potential benefits, the use of vitamin D supplements has increased markedly, raising concerns about the risk of excessive intake. The vitamin D and Omega-3 Trial (VITAL) study, a randomized trial involving over 25,000 participants, discovered that vitamin D supplementation did not significantly reduce the risk of all-cause mortality or lower risk of fractures among generally healthy midlife and older adults without vitamin D deficiency or osteoporosis [37]. Furthermore, increased intake of vitamin D supplements by the general population and a growing number of high-dose prescriptions without medical monitoring might result in a greater risk of excessive vitamin D toxicity [38]. Our current results indicated that the association of 25(OH)D serum level and biological aging followed a U-shaped curve in males and an L-shaped curve in females. This suggests that high serum level of 25(OH)D is not associated with slower aging and may, in fact, be linked to accelerated biological aging in males. These detrimental effects may stem from the toxicity of excessive vitamin D. Excessive intake can cause serum levels of its metabolites, such as 25(OH)D and $24,25(OH)_2D$, to surpass the binding capacity of vitamin D binding protein, leading to

an increase in free, active 1,25$(OH)_2$D. This active form reaches the VDR in the nucleus, constantly promoting gene expression that enhances calcium absorption and bone mobilization [39]. Consequently, this can result in hypercalcemia or hyperphosphatemia, which in turn suppresses initial parathyroid hormone (iPTH) level [39, 40]. Such disruptions in calcium balance and hormone regulation may contribute to cardiovascular disease, kidney damage, and skin aging. Research reported that the working diagnosis of vitamin D toxicity is diagnosed based on total 25(OH)D concentrations of 80~200nmol/L detected by immunoassay [41]. Our findings that the 25(OH)D levels over 91.5 nmol/L in male participants were not linked to accelerated biological aging are approximately consistent with those reports.

Our study identified a transition point in the relationship between 25(OH)D serum level and biological aging, with males exhibiting a threshold at 38.20 nmol/L and females at 62.50 nmol/L. Beyond this transition point, increase in 25(OH)D level no longer associates with biological aging deceleration. Regarding the threshold of vitamin D deficiency, it remains an ongoing controversy. The Scientific Advisory Committee on Nutrition (2016) recommended threshold at 25 nmol/L among UK population [42], whereas the IOM (2011) suggested a threshold of 30 nmol/L based on evidence in U.S. and Canadian populations [17]. Meanwhile, the European Food Safety Authority 2016 and the Endocrine Society guideline 2011 proposed higher level of 50 nmol/L as deficiency cutoff [43, 44]. The discrepancy in cutoff values stems from multiple factors, including different regional ultraviolet B exposure patterns, dietary practice and population-specific biological variability. Furthermore, guideline variations also reflect differing priorities concerning health outcomes, with some prioritizing musculoskeletal health [45]. Notably, in 2024, the Endocrine Society guideline retracted their previous recommendation of threshold at 50 nmol/L due to limited clinical trial evidence [46]. They also highlighted the impracticality of universal thresholds due to heterogeneity in health outcomes and population characteristics. Therefore, the interpretation of our findings regarding 25(OH)D level threshold should be with caution, especially since it derived from observational study in an ethnically diverse population. Nevertheless, the trend of benefits attenuation we observed

is worth noting, suggesting excessive level of serum 25(OH)D may not be beneficial regarding biological aging process.

Our results revealed sex-specific differences of dose-response relationship between serum 25(OH)D levels and biological aging, which may be attributed to the sex hormones, particularly estrogen. As women age, especially during the menopausal transition and after menopause, their ovaries produce less estrogen, leading to a decline of estrogen levels. This decline contributes to the thinning and wrinkling of the skin, as well as changes in hair texture and growth patterns [47]. Some studies suggest that vitamin D could enhance the activity of estrogen receptors (ER) via interacting with them, potentially amplifying the anti-aging effects of estrogen in the body [48]. Additionally, vitamin D and estrogen also have a synergistic effect on bone metabolism, helping to prevent osteoporosis and fractures, especially in postmenopausal women [49]. Vitamin D may also regulate estrogen metabolism, leading to higher circulating estrogen levels and subsequently delay aging [48]. Therefore, high level of serum 25(OH)D may not be associated with decelerated biological aging in females. However, the mechanisms underlying the adverse effects observed at extremely high levels (>100 nmol/L) in males remain unclear. Further studies are needed to clarify the complex interplay between these two important hormonal systems.

This study has several limitations. First, as a cross-sectional investigation, it only assesses association between serum 25(OH)D levels and biological aging, making it challenging to establish the causality. Second, the classification of 25(OH)D serum levels used in our study is based on the 2011 IOM guidelines; however, more recent classifications, such as those from the Scientific Advisory Committee on Nutrition (2016) [50], may provide more accurate benchmarks. Analyzing the relationship using these updated criteria would be preferable. While sun exposure and dietary intake are primary sources of vitamin D, data on these factors, as well as supplement use, were not included in our analysis due to the lack of corresponding data in the NHANES dataset. The absence of these confounding variables may introduce residual confounding, reverse causation, and bias. Additionally, race significantly influences

serum vitamin D levels, but the definition of race varies across NHANES data collected over different years, which posed a challenge in our study. Finally, emerging evidence questions the traditional view of serum 25(OH)D as a definitive biomarker of vitamin D status, although it remains the most widely accepted marker. For instance, in individuals with diabetes or obesity, the expression of CYP2R1—the enzyme responsible for vitamin D 25-hydroxylation—is suppressed, leading to decreased 25(OH)D levels despite stable overall vitamin D levels [51, 52].

Conclusion

In this study, we investigated the relationship between 25(OH)D serum levels and PhenoAge in NHANES, and found that there was a negative association between PhenoAgeAccel and 25(OH)D serum level, and these associations were nonlinear in both genders, revealing a U-shaped curve for males and an L-shaped curve for females. Low 25(OH)D serum levels shows a potential positively link to PhenoAgeAccel in both genders, but high 25(OH)D serum levels do not associate with decelerated PhenoAge, and even associated with accelerated PhenoAge in male. Our results suggest that the association between vitamin D levels with biological aging is different in men and women.

Authors contributions

Yi Feng: Conceptualization; Methodology; Data curation; Formal analysis; Writing original draft**; Licheng Yu**: Data curation; Formal analysis; **Jue Wang**: Project administration; Supervision; Writing - review &editing.

Conflicts of interest

The authors declare no competing financial or non-financial interests related to this work. NHANES is a publicly available dataset managed by the Centers for Disease Control and Prevention. The authors confirm that there is no affiliation with or involvement from the NHANES program administrators in this study. The funding

source had no role in study design, data collection, analysis, or manuscript preparation.

References

1. Bikle DD. Vitamin D metabolism, mechanism of action, and clinical applications. Chem Biol. 2014;21(3):319-29. Epub 2014/02/18. doi: 10.1016/j.chembiol.2013.12.016. PubMed PMID: 24529992; PubMed Central PMCID: PMCPMC3968073.
2. Dominguez LJ, Farruggia M, Veronese N, Barbagallo M. Vitamin D Sources, Metabolism, and Deficiency: Available Compounds and Guidelines for Its Treatment. Metabolites. 2021;11(4). Epub 2021/05/01. doi: 10.3390/metabo11040255. PubMed PMID: 33924215; PubMed Central PMCID: PMCPMC8074587.
3. Couchman L, Moniz CF. Analytical considerations for the biochemical assessment of vitamin D status. Ther Adv Musculoskelet Dis. 2017;9(4):97-104. Epub 2017/04/07. doi: 10.1177/1759720X17692500. PubMed PMID: 28382113; PubMed Central PMCID: PMCPMC5367642.
4. Rahman A, Elmi A. Air pollutants are negatively associated with vitamin D-synthesizing UVB radiation intensity on the ground. Sci Rep. 2021;11(1):21480. Epub 2021/11/04. doi: 10.1038/s41598-021-00980-6. PubMed PMID: 34728744; PubMed Central PMCID: PMCPMC8563978.
5. Zhao S, Qian F, Wan Z, Chen X, Pan A, Liu G. Vitamin D and major chronic diseases. Trends Endocrinol Metab. 2024. Epub 2024/06/02. doi: 10.1016/j.tem.2024.04.018. PubMed PMID: 38824035.
6. Gaksch M, Jorde R, Grimnes G, Joakimsen R, Schirmer H, Wilsgaard T, et al. Vitamin D and mortality: Individual participant data meta-analysis of standardized 25-hydroxyvitamin D in 26916 individuals from a European consortium. Plos One. 2017;12(2):e0170791. Epub 2017/02/17. doi: 10.1371/journal.pone.0170791. PubMed PMID: 28207791; PubMed Central PMCID: PMCPMC5312926.
7. Fang A, Zhao Y, Yang P, Zhang X, Giovannucci EL. Vitamin D and human health: evidence from Mendelian randomization studies. Eur J Epidemiol. 2024;39(5):467-90. Epub 2024/01/12. doi: 10.1007/s10654-023-01075-4. PubMed PMID: 38214845.
8. Cui A, Xiao P, Ma Y, Fan Z, Zhou F, Zheng J, et al. Prevalence, trend, and predictor analyses of vitamin D deficiency in the US population, 2001-2018. Front Nutr. 2022;9:965376. Epub 2022/10/21. doi: 10.3389/fnut.2022.965376. PubMed PMID: 36263304; PubMed Central PMCID: PMCPMC9573946.
9. Berridge MJ. Vitamin D deficiency accelerates ageing and age-related diseases: a novel hypothesis. The Journal of physiology. 2017;595(22):6825-36. Epub 2017/09/28. doi: 10.1113/JP274887. PubMed PMID: 28949008; PubMed Central PMCID: PMCPMC5685827.
10. Zittermann A, Ernst JB, Prokop S, Fuchs U, Dreier J, Kuhn J, et al. Effect of vitamin D on all-cause mortality in heart failure (EVITA): a 3-year randomized clinical trial with 4000 IU vitamin D daily. Eur Heart J. 2017;38(29):2279-86. Epub 2017/05/13. doi: 10.1093/eurheartj/ehx235. PubMed PMID: 28498942.
11. Scragg R, Stewart AW, Waayer D, Lawes CMM, Toop L, Sluyter J, et al. Effect of Monthly High-Dose Vitamin D Supplementation on Cardiovascular Disease in the Vitamin D Assessment Study : A Randomized Clinical Trial. JAMA Cardiol. 2017;2(6):608-16. Epub 2017/04/07. doi: 10.1001/jamacardio.2017.0175. PubMed PMID: 28384800; PubMed Central PMCID:

PMCPMC5815022 ICMJE Form of Disclosure of Potential Conflicts of Interest and none were reported.
12. Xia X, Wang Y, Yu Z, Chen J, Han JJ. Assessing the rate of aging to monitor aging itself. Ageing Res Rev. 2021;69:101350. Epub 2021/05/04. doi: 10.1016/j.arr.2021.101350. PubMed PMID: 33940202.
13. Jylhava J, Pedersen NL, Hagg S. Biological Age Predictors. EBioMedicine. 2017;21:29-36. Epub 2017/04/12. doi: 10.1016/j.ebiom.2017.03.046. PubMed PMID: 28396265; PubMed Central PMCID: PMCPMC5514388.
14. Bischoff-Ferrari HA, Gangler S, Wieczorek M, Belsky DW, Ryan J, Kressig RW, et al. Individual and additive effects of vitamin D, omega-3 and exercise on DNA methylation clocks of biological aging in older adults from the DO-HEALTH trial. Nat Aging. 2025;5(3):376-85. Epub 2025/02/04. doi: 10.1038/s43587-024-00793-y. PubMed PMID: 39900648.
15. Kwon D, Belsky DW. A toolkit for quantification of biological age from blood chemistry and organ function test data: BioAge. Geroscience. 2021;43(6):2795-808. Epub 2021/11/03. doi: 10.1007/s11357-021-00480-5. PubMed PMID: 34725754; PubMed Central PMCID: PMCPMC8602613.
16. Centers for Disease Control and Prevention National Health and Nutrition Examination Survey Questionnaires D, and Related Documentation 2020. Available from: https://wwwn.cdc.gov/nchs/nhanes/.
17. Ross AC, Taylor CL, Yaktine AL, Del Valle HB. Dietary Reference Intakes for Calcium and Vitamin D. The National Academies Collection: Reports funded by National Institutes of Health. Washington (DC)2011.
18. Liu Z, Kuo PL, Horvath S, Crimmins E, Ferrucci L, Levine M. A new aging measure captures morbidity and mortality risk across diverse subpopulations from NHANES IV: A cohort study. PLoS Med. 2018;15(12):e1002718. Epub 2019/01/01. doi: 10.1371/journal.pmed.1002718. PubMed PMID: 30596641; PubMed Central PMCID: PMCPMC6312200.
19. Liu W, Wang J, Wang M, Hou H, Ding X, Ma L, et al. Oxidative Stress Factors Mediate the Association Between Life's Essential 8 and Accelerated Phenotypic Aging: NHANES 2005-2018. J Gerontol A Biol Sci Med Sci. 2024;79(1). Epub 2023/10/10. doi: 10.1093/gerona/glad240. PubMed PMID: 37813096.
20. He H, Chen X, Ding Y, Chen X, He X. Composite dietary antioxidant index associated with delayed biological aging: a population-based study. Aging (Albany NY). 2024;16(1):15-27. Epub 2024/01/04. doi: 10.18632/aging.205232. PubMed PMID: 38170244; PubMed Central PMCID: PMCPMC10817368.
21. Haskell WL, Lee IM, Pate RR, Powell KE, Blair SN, Franklin BA, et al. Physical activity and public health: updated recommendation for adults from the American College of Sports Medicine and the American Heart Association. Circulation. 2007;116(9):1081-93. Epub 2007/08/03. doi: 10.1161/CIRCULATIONAHA.107.185649. PubMed PMID: 17671237.
22. Chaput JP, McNeil J, Despres JP, Bouchard C, Tremblay A. Seven to eight hours of sleep a night is associated with a lower prevalence of the metabolic syndrome and reduced overall cardiometabolic risk in adults. PLoS One. 2013;8(9):e72832. Epub 2013/09/17. doi: 10.1371/journal.pone.0072832. PubMed PMID: 24039808; PubMed Central PMCID: PMCPMC3764138.
23. Muntner P, Hardy ST, Fine LJ, Jaeger BC, Wozniak G, Levitan EB, et al. Trends in Blood Pressure Control Among US Adults With Hypertension, 1999-2000 to 2017-2018. JAMA. 2020;324(12):1190-200. Epub 2020/09/10. doi: 10.1001/jama.2020.14545. PubMed PMID: 32902588; PubMed Central PMCID: PMCPMC7489367 consulting fees from Amgen Inc. Dr Levitan reported receiving grant funding from and serving on advisory boards for Amgen Inc; and serving as a consultant to Novartis. Dr Colantonio reported receiving grant funding from Amgen Inc. No other disclosures were reported.

24. American Diabetes A. Diagnosis and classification of diabetes mellitus. Diabetes Care. 2014;37 Suppl 1:S81-90. Epub 2013/12/21. doi: 10.2337/dc14-S081. PubMed PMID: 24357215.
25. Bender R. Introduction to the use of regression models in epidemiology. Cancer epidemiology: Springer; 2009. p. 179-95.
26. Hastie TJ. Generalized additive models. Statistical models in S. 2017:249-307.
27. Muggeo VM. Estimating regression models with unknown break-points. Stat Med. 2003;22(19):3055-71. Epub 2003/09/16. doi: 10.1002/sim.1545. PubMed PMID: 12973787.
28. Wood SN. Stable and efficient multiple smoothing parameter estimation for generalized additive models. Journal of the American Statistical Association. 2004;99(467):673-86.
29. World Medical A. World Medical Association Declaration of Helsinki: Ethical Principles for Medical Research Involving Human Participants. JAMA. 2025;333(1):71-4. Epub 2024/10/19 20:42. doi: 10.1001/jama.2024.21972. PubMed PMID: 39425955.
30. Schottker B, Hagen L, Zhang Y, Gao X, Holleczek B, Gao X, et al. Serum 25-Hydroxyvitamin D Levels as an Aging Marker: Strong Associations With Age and All-Cause Mortality Independent From Telomere Length, Epigenetic Age Acceleration, and 8-Isoprostane Levels. J Gerontol A Biol Sci Med Sci. 2019;74(1):121-8. Epub 2018/10/30. doi: 10.1093/gerona/gly253. PubMed PMID: 30371905.
31. Liu L, Chen C, Lo K, Huang J, Yu Y, Huang Y, et al. Serum 25-hydroxyvitamin D, frailty, and mortality among the Chinese oldest old: Results from the CLHLS study. Nutr Metab Cardiovasc Dis. 2021;31(9):2707-15. Epub 2021/08/06. doi: 10.1016/j.numecd.2021.05.033. PubMed PMID: 34348876.
32. Veldurthy V, Wei R, Oz L, Dhawan P, Jeon YH, Christakos S. Vitamin D, calcium homeostasis and aging. Bone Res. 2016;4:16041. Epub 2016/10/30. doi: 10.1038/boneres.2016.41. PubMed PMID: 27790378; PubMed Central PMCID: PMCPMC5068478.
33. Ge D, Chen Q, Xie X, Li Q, Yang Y. Unveiling the potent effect of vitamin D: harnessing Nrf2/HO-1 signaling pathways as molecular targets to alleviate urban particulate matter-induced asthma inflammation. BMC Pulm Med. 2024;24(1):55. Epub 2024/01/26. doi: 10.1186/s12890-024-02869-2. PubMed PMID: 38273268; PubMed Central PMCID: PMCPMC10809564.
34. Pilz S, Gaksch M, Kienreich K, Grubler M, Verheyen N, Fahrleitner-Pammer A, et al. Effects of vitamin D on blood pressure and cardiovascular risk factors: a randomized controlled trial. Hypertension. 2015;65(6):1195-201. Epub 2015/03/25. doi: 10.1161/HYPERTENSIONAHA.115.05319. PubMed PMID: 25801871.
35. Chowdhury R, Kunutsor S, Vitezova A, Oliver-Williams C, Chowdhury S, Kiefte-de-Jong JC, et al. Vitamin D and risk of cause specific death: systematic review and meta-analysis of observational cohort and randomised intervention studies. BMJ. 2014;348:g1903. Epub 2014/04/03. doi: 10.1136/bmj.g1903. PubMed PMID: 24690623; PubMed Central PMCID: PMCPMC3972416.
36. Rejnmark L, Bislev LS, Cashman KD, Eiriksdottir G, Gaksch M, Grubler M, et al. Non-skeletal health effects of vitamin D supplementation: A systematic review on findings from meta-analyses summarizing trial data. Plos One. 2017;12(7):e0180512. Epub 2017/07/08. doi: 10.1371/journal.pone.0180512. PubMed PMID: 28686645; PubMed Central PMCID: PMCPMC5501555.
37. LeBoff MS, Chou SH, Ratliff KA, Cook NR, Khurana B, Kim E, et al. Supplemental Vitamin D and Incident Fractures in Midlife and Older Adults. N Engl J Med. 2022;387(4):299-309. Epub 2022/08/09. doi: 10.1056/NEJMoa2202106. PubMed PMID: 35939577; PubMed Central PMCID: PMCPMC9716639.
38. Pludowski P, Grant WB, Karras SN, Zittermann A, Pilz S. Vitamin D Supplementation: A Review

of the Evidence Arguing for a Daily Dose of 2000 International Units (50 microg) of Vitamin D for Adults in the General Population. Nutrients. 2024;16(3). Epub 2024/02/10. doi: 10.3390/nu16030391. PubMed PMID: 38337676; PubMed Central PMCID: PMCPMC10857599.

39. Marcinowska-Suchowierska E, Kupisz-Urbanska M, Lukaszkiewicz J, Pludowski P, Jones G. Vitamin D Toxicity-A Clinical Perspective. Front Endocrinol (Lausanne). 2018;9:550. Epub 2018/10/09. doi: 10.3389/fendo.2018.00550. PubMed PMID: 30294301; PubMed Central PMCID: PMCPMC6158375.

40. Batman A, Saygili ES, Yildiz D, Sen EC, Erol RS, Canat MM, et al. Risk of hypercalcemia in patients with very high serum 25-OH vitamin D levels. Int J Clin Pract. 2021;75(7):e14181. Epub 2021/03/25. doi: 10.1111/ijcp.14181. PubMed PMID: 33759301; PubMed Central PMCID: PMCPMC8250214.

41. Sajid U, Orton D, Kaufmann M, Jones G, Kline GA. Understanding Elevated Vitamin D Measurements to Uncover Hypercalcemia Etiology. Clin Chem. 2024;70(6):798-802. Epub 2024/06/03. doi: 10.1093/clinchem/hvae044. PubMed PMID: 38825340.

42. England PH. SACN vitamin D and health report 2016 [updated 2025-04-22]. Available from: https://www.gov.uk/government/publications/sacn-vitamin-d-and-health-report.

43. Turck D, Bresson J, Burlingame B. Scientific opinion on dietary reference values for vitamin D EFSA Panel on Dietetic Products, Nutrition, and Allergies (NDA). EFSA J. 2016;179.

44. Holick MF, Binkley NC, Bischoff-Ferrari HA, Gordon CM, Hanley DA, Heaney RP, et al. Evaluation, treatment, and prevention of vitamin D deficiency: an Endocrine Society clinical practice guideline. J Clin Endocrinol Metab. 2011;96(7):1911-30. Epub 2011/06/08. doi: 10.1210/jc.2011-0385. PubMed PMID: 21646368.

45. Roth DE, Abrams SA, Aloia J, Bergeron G, Bourassa MW, Brown KH, et al. Global prevalence and disease burden of vitamin D deficiency: a roadmap for action in low- and middle-income countries. Ann N Y Acad Sci. 2018;1430(1):44-79. Epub 2018/09/19. doi: 10.1111/nyas.13968. PubMed PMID: 30225965; PubMed Central PMCID: PMCPMC7309365.

46. Demay MB, Pittas AG, Bikle DD, Diab DL, Kiely ME, Lazaretti-Castro M, et al. Vitamin D for the Prevention of Disease: An Endocrine Society Clinical Practice Guideline. J Clin Endocrinol Metab. 2024. Epub 2024/06/03. doi: 10.1210/clinem/dgae290. PubMed PMID: 38828931.

47. Santoro N, Roeca C, Peters BA, Neal-Perry G. The Menopause Transition: Signs, Symptoms, and Management Options. J Clin Endocrinol Metab. 2021;106(1):1-15. Epub 2020/10/24. doi: 10.1210/clinem/dgaa764. PubMed PMID: 33095879.

48. Crescioli C. The Role of Estrogens and Vitamin D in Cardiomyocyte Protection: A Female Perspective. Biomolecules. 2021;11(12). Epub 2021/12/25. doi: 10.3390/biom11121815. PubMed PMID: 34944459; PubMed Central PMCID: PMCPMC8699224.

49. McCurdy R, Jiang X, Clarkson TB, Nudy M, Schnatz PF. Vitamin D and conjugated equine estrogen: the association with coronary artery atherosclerosis in cynomolgus monkeys. Menopause. 2016;23(5):481-7. Epub 2016/02/18. doi: 10.1097/GME.0000000000000582. PubMed PMID: 26886883.

50. Jenkins DJA, Spence JD, Giovannucci EL, Kim YI, Josse RG, Vieth R, et al. Supplemental Vitamins and Minerals for Cardiovascular Disease Prevention and Treatment: JACC Focus Seminar. J Am Coll Cardiol. 2021;77(4):423-36. Epub 2021/01/30. doi: 10.1016/j.jacc.2020.09.619. PubMed PMID: 33509399.

51. Elkhwanky MS, Kummu O, Piltonen TT, Laru J, Morin-Papunen L, Mutikainen M, et al. Obesity Represses CYP2R1, the Vitamin D 25-Hydroxylase, in the Liver and Extrahepatic Tissues. JBMR Plus.

2020;4(11):e10397. Epub 2020/11/20. doi: 10.1002/jbm4.10397. PubMed PMID: 33210060; PubMed Central PMCID: PMCPMC7657391.

52. Aatsinki SM, Elkhwanky MS, Kummu O, Karpale M, Buler M, Viitala P, et al. Fasting-Induced Transcription Factors Repress Vitamin D Bioactivation, a Mechanism for Vitamin D Deficiency in Diabetes. Diabetes. 2019;68(5):918-31. Epub 2019/03/06. doi: 10.2337/db18-1050. PubMed PMID: 30833469; PubMed Central PMCID: PMCPMC6477896.

Table 1. Characteristics of the study population

Characteristics	Male (n=6836)	Female (n=7074)	*P*-value
Age (mean±SD)	57.07 (11.3)	58.12 (11.8)	<0.001
Race			0.001[b]
Non-Hispanic white	3249 (47.5)	3288 (46.5)	
Non-Hispanic black	1360 (19.9)	1417 (20.0)	
Others (Asian, Hispanic white, etc.)	2227 (32.6)	2369 (33.5)	
Education level			0.394[b]
Below high school	1847 (27.0)	1831 (25.9)	
High school or equivalent	1588 (23.2)	1597 (22.6)	
Above high school	3401 (49.8)	3646 (51.5)	
Poverty-income ratio			<0.001[b]
≤1.0	1199 (17.5)	1447 (20.5)	
1.0-3.0	2839 (41.5)	3029 (42.8)	
>3.0	2798 (40.9)	2598 (36.7)	
BMI, kg/m^2			<0.001[b]
<25	1585 (23.2)	1841 (26.0)	
25-30	2749 (40.2)	2117 (29.9)	
≥30	2502 (36.6)	3116 (44.1)	
Smoking			<0.001[b]
Never smoker	2823 (41.3)	4297 (60.7)	
Previous smoker	2570 (37.6)	1666 (23.6)	
Current smoker	1443 (21.1)	1111 (15.7)	
Drinking (alcohol consumption)			<0.001[b]
Never drinker	527 (7.7)	1572 (22.2)	
Previous drinker	637 (9.3)	1466 (20.7)	
Current drinker	5672 (83.0)	4036 (57.1)	
Level of physical activity			<0.001[b]
Inactive	1775 (26.0)	2533 (35.8)	
Insufficient	802 (11.7)	1086 (15.4)	
Moderate	680 (10.0)	842 (11.9)	
High	3579 (52.4)	2613 (36.9)	
Sleep duration			<0.001[b]
<7 h	2572 (37.6)	2445 (34.6)	
7-8h	1858 (27.2)	1812 (25.6)	
≥8 h	2406 (35.2)	2817 (39.8)	

Hypertension			0.839[b]
No	3007 (44.0)	3067 (43.4)	
Yes	3829 (56.0)	4007 (56.6)	
Diabetes mellitus			<0.001[b]
No	5086 (74.4)	5515 (78.0)	
Yes	1750 (25.6)	1559 (22.0)	
25(OH)D Serum level, nmol/L			
Median (IQR)	63.8 (48.4, 80.2)	66.6 (47.0, 87.3)	<0.001[c]
<30	420 (6.1)	545 (7.7)	<0.001[b]
30-50	1451 (21.2)	1457 (20.6)	
50-75	2766 (40.5)	2293 (32.4)	
⩾75	2199 (32.2)	2779 (39.3)	
PhenoAge (median [IQR])	53.8 (45.1, 64.6)	53.1 (44.2, 64.1)	0.032
PhenoAgeAccel (median [IQR])	-2.4 (-5.1, 0.9)	-4.1 (-7.2, -0.5)	<0.001[c]
Standardized PhenoAgeAccel (mean±SD)	0.1 (0.9)	-0.2 (1.1)	<0.001[a]

[a] Student's t-test was used to compare means of variables between two groups

[b] Chi-square test was used to compare categorical variables between two groups.

[c] Mann-Whitney test was used to compare non-normal variables between two groups.

Table 2. Association between 25(OH)D serum level and PhenoAgeAccel

Characteristics	All			Male			Female		
	Beta coefficient (95% CI)		*P*-value	Beta coefficient (95% CI)		*P*-value	Beta coefficient (95% CI)		*P*-value
	Crude model	Adjusted model [a]		Crude model	Adjusted model [a]		Crude model	Adjusted model [a]	
Log(25(OH)D)	-0.27 (-0.32, -0.22)	-0.04 (-0.08, 0.00)	0.039	-0.13 (-0.20, -0.06)	0.03 (-0.02, 0.09)	0.334	-0.32 (-0.38, -0.26)	-0.07 (-0.12, -0.01)	0.007
Quartiles									
Q1	Reference	Reference		Reference	Reference		Reference	Reference	
Q2	-0.23 (-0.29, -0.17)	-0.10 (-0.16, -0.05)	<0.001	-0.16 (-0.24, -0.08)	-0.02 (-0.09, 0.04)	0.448	-0.33 (-0.41, -0.25)	-0.16 (-0.23, -0.08)	<0.001
Q3	-0.29 (-0.36, -0.23)	-0.10 (-0.16, -0.04)	0.001	-0.20 (-0.29, -0.11)	-0.03 (-0.12, 0.04)	0.355	-0.40 (-0.49, -0.32)	-0.15 (-0.22, -0.07)	<0.001
Q4	-0.32 (-0.38, -0.26)	-0.05 (-0.11, -0.00)	0.022	-0.16 (-0.24, -0.07)	0.02 (-0.04, 0.10)	0.553	-0.42 (-0.51, -0.34)	-0.10 (-0.18, -0.02)	0.009
P for trend			0.173			0.429			0.076
Categories based on the 2011 Institute of Medicine									
<30	Reference	Reference		Reference	Reference		Reference	Reference	
30-50	-0.23 (-0.33, -0.13)	-0.14 (-0.23, -0.05)	0.002	-0.26 (-0.41, -0.11)	-0.15 (-0.29, -0.02)	0.024	-0.22 (-0.34, -0.09)	-0.10 (-0.21, 0.00)	0.057
50-75	-0.42 (-0.52, -0.33)	-0.21 (-0.30, -0.13)	<0.001	-0.39 (-0.53, -0.25)	-0.16 (-0.29, -0.04)	0.009	-0.51 (-0.63, -0.40)	-0.24 (-0.33, -0.15)	<0.001
⩾75	-0.46 (-0.56, -0.36)	-0.15 (-0.24, -0.06)	0.001	-0.36 (-0.50, -0.22)	-0.09 (-0.22, 0.03)	0.131	-0.54 (-0.66, -0.42)	-0.17 (-0.27, -0.07)	0.001
P for trend			0.130			0.366			0.014

[a] Adjusted for age, race, education level, poverty-income ratio, body mass index, smoking, drinking, physical activity, sleep duration,

hypertension, diabetes and season of measurement.

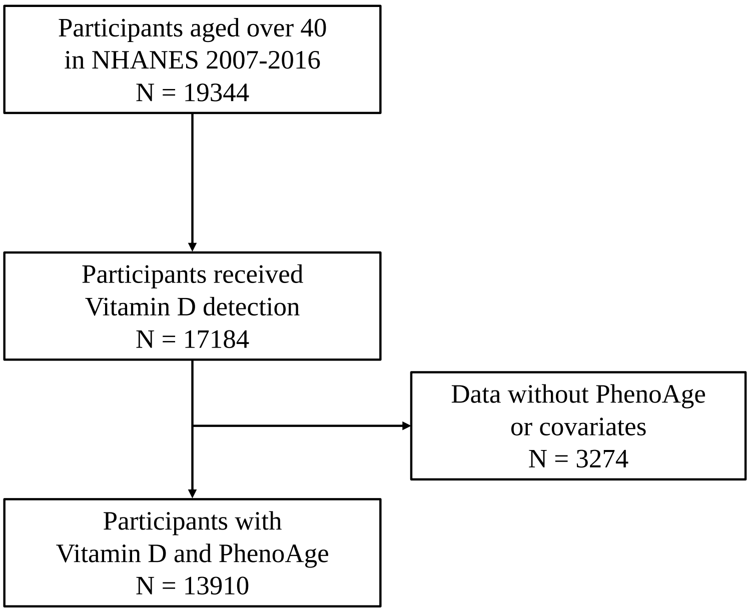

Figure 1 Flowchart of participants selection.

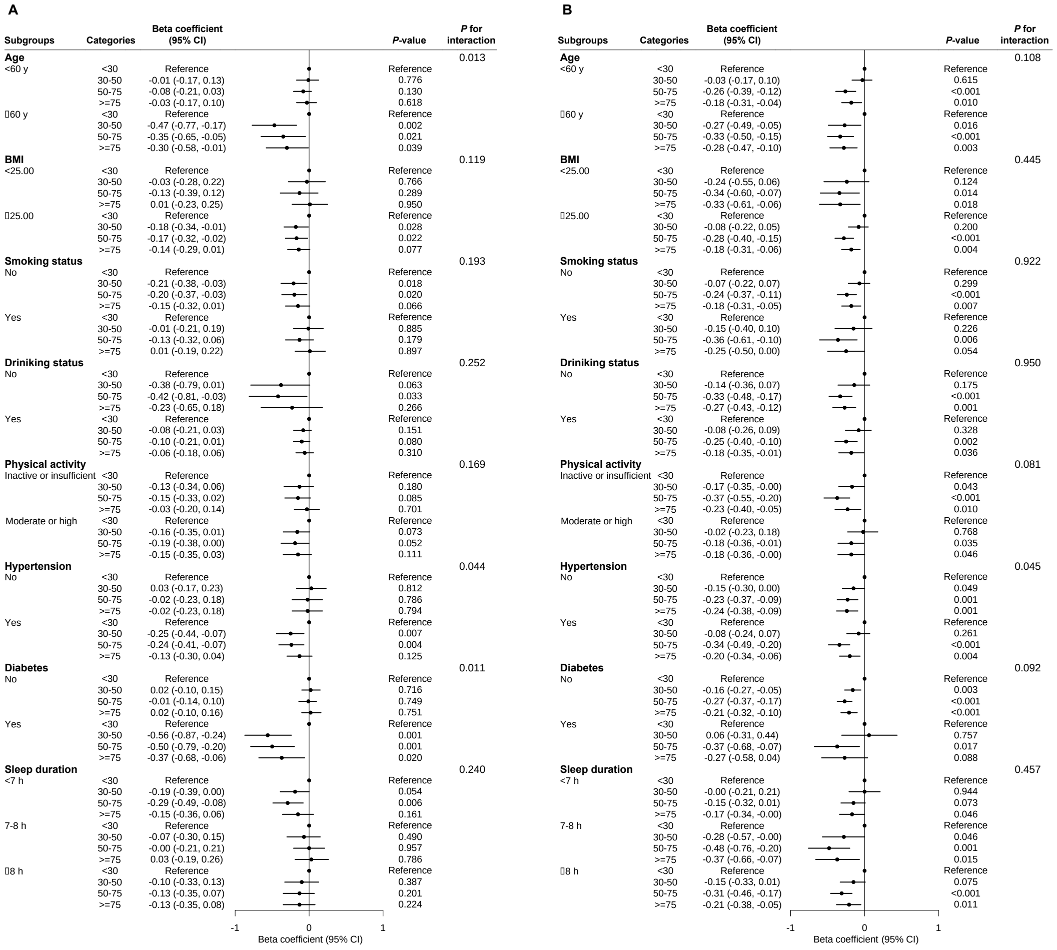

Figure 2 Subgroup analyses of the association between 25(OH)D categories (based on the 2011 Institute of Medicine) and PhenoAgeAccel among males (A) and females (B). The filled circles and horizontal lines represent regression coefficients and the 95% confidence intervals. Multiple linear regression models were adjusted for age, race, education level, poverty-income ratio, body mass index, smoking, drinking, physical activity, sleep duration, hypertension, diabetes and season. BMI, body mass index; CI, confidence intervals.

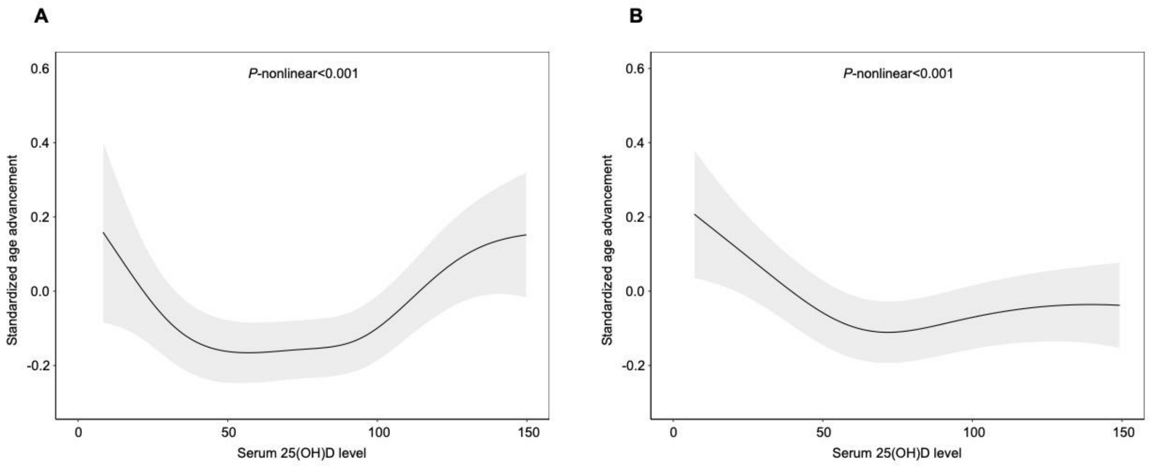

Figure 3 Nonlinear relationships between 25(OH)D serum level and PhenoAgeAccel among males (A) and females (B). Generalized additive models were adjusted for age, race, education level, poverty-income ratio, body mass index, smoking, drinking, physical activity, sleep duration, hypertension, diabetes and season. The black solid line represents the smooth curve fit between variables. The grey band represents the 95% confidence interval of the fit.

Table 3. Results of the analysis of the relationship between 25(OH)D serum levels and PhenoAge

25(OH)D serum levels (nmol/L)	Beta coefficient (95% CI)	*P*-value
Male		
<38.2	-0.013 (-0.025, -0.002)	0.017
38.2-91.6	0.001 (-0.001, 0.002)	0.487
⩾91.6	0.005 (0.002, 0.008)	<0.001
Female		
<62.5	-0.007 (-0.010, -0.004)	<0.001
62.5-125.0	0.002 (0.000, 0.004)	0.029
⩾125.0	-0.001 (-0.005, 0.002)	0.535

Generalized additive models were adjusted for age, race, education level, poverty-income ratio, body mass index, smoking, drinking, physical activity, sleep duration, hypertension, diabetes and season.

Click here to access/download
Supporting Information
Supplemental materials.docx

Click here to access/download
Other
PLOSOne_Human_Subjects_Research_Checklist.docx

Association of 25(OH)D serum level with biological aging: A Cross-Sectional Study of 2007-2016 NHANES surveys

Yi Feng[a]; Licheng Yu[b]; Jue Wang[a] *

[a] Nanhui new town Community Health Service Center, Pudong New Area, Shanghai, 201308, China.

[b] Prevention and Health Care Department, Heqing Community Health Service Center of Pudong New District, Shanghai, 201201, China.

Corresponding author. Jue Wang

E-mail address: wangjue180130@126.com

Abstract

Vitamin D plays a crucial role in various aspects of human body, including aging, but chronological age may not accurately reflect the true biological aging status. Recently, PhenoAge has been developed to estimate an individual's biological age based on different biological and clinical measures. Therefore, we investigated the relationship between 25(OH)D serum levels and biological aging calculated by PhenoAge Acceleration (PhenoAgeAccel) using data from the 2007-2016 National Health and Nutrition Examination Survey (NHANES). 25(OH)D serum levels were negatively associated with PhenoAgeAccel (β=-0.04 standard deviation [SD]; 95% confidence interval [CI]: -0.08 to 0.00). This negative association was dose-dependent in females (β=-0.07 SD; 95% CI: -0.12 to 0.01), but not in males. Generalized additive models further revealed gender-specific non-linear patterns: a a U-shape pattern in males but an L-shaped pattern in females. Using segmented regression to confirm inflection points, we observed that 25(OH)D serum levels were linked to reduced PhenoAgeAccel at levels below 38.2 nmol/L (15.3 ng/mL) in males (β=-0.013 SD; 95% CI: -0.025 to -0.002) and 62.5 nmol/L (25.0 ng/mL) in females (β=-0.007 SD; 95% CI: -0.01 to -0.004,). However, 25(OH)D serum levels above 125 nmol/L showed no association with PhenoAgeAccel in females (β=-0.001 SD; 95% CI: -0.005-0.002), while in males, elevated levels (>91.556 nmol/L) were associated with increased PhenoAgeAccel (β=0.005 SD; 95% CI: 0.002-0.008). 25(OH)D serum levels showed a negative association with PhenoAgeAccel (β=-0.04; 95% confidence interval [CI]: -0.08 to 0.00). This negative association was dose-dependent in females (β=-0.07; 95% CI: -0.12 to 0.01), but not in males. Generalized additive models further found 25(OH)D serum levels were linked to negative PhenoAgeAccel at levels below 38.2 nmol/L (15.3 ng/mL) in males (β=-0.013; 95% CI: -0.025 to -0.002) and 62.5 nmol/L (25.0 ng/mL) in females (β=-0.007; 95% CI: -0.01 to -0.004,), revealing a U-shaped relationship observed in males but an L-shaped pattern in females. . However, 25(OH)D serum levels above 125 nmol/L were not associated with PhenoAgeAccel in females (β=-0.001; 95% CI: -0.005-0.002), while in males, elevated levels (>91.55 nmol/L) were associated with positive PhenoAgeAccel (β=0.005; 95% CI: 0.002-0.008). Our findings indicate that

Formatted: Font color: Auto

vitamin D insufficiency has an inverse link with accelerated biological aging, and high levels of vitamin D in males accelerated biological aging as well, offering valuable insights into the relationship between vitamin D and biological aging.

Key words: Vitamin D; Serum 25(OH)D; Biological aging; PhenoAge; Nonlinear association

~~1.~~ Introduction

Vitamin D is a fat-soluble vitamin that plays a crucial role in ~~various aspects of~~ human health, influencing various functions such as bone metabolism and immune regulation~~with its effects extending to bone metabolism, immune function, etc~~. It is primarily obtained~~acquired~~ through two~~ primary~~ sources: endogenous production (mainly vitamin D3) in the skin upon exposure to ultraviolet B (UVB) radiations, and exogenous intake (vitamin D2 and D3) through dietary sources, such as fatty fish, egg yolks or supplements. Once absorbed in the body, vitamin D is converted into 25-hydroxyvitamin D (25(OH)D) in the liver, then transformed into the biologically active form, 1α,25-dihydroxyvitamin D (1α,25(OH)$_2$D), in the kidneys [1]. This active form binds to the vitamin D receptor (VDR) to~~,~~ regulat~~inge~~e calcium and phosphate metabolism [2]. Serum 25(OH)D is the most commonly measured form of vitamin D in humans because it is the major circulating form and provides an accurate indication of the body's overall vitamin D status [3]; and ~~it~~ can be influenced by various factors including the geographic location, race and food intake [4].

Vitamin D deficiency is a global public health concern, associated with increased risk of type 2 diabetes (T2D), hypertension, multiple sclerosis [5-7]. It is especially prevalent among the elderly ~~The prevalence of vitamin D deficiency is particularly high among the elderly~~ [8], with some studies suggesting that deficiency ~~it~~ may accelerate chronological aging and contribute to age-related diseases and inflammatory diseases, such as cardiovascular disease, multiple sclerosis and rheumatoid arthritis [9]. However, several large randomized clinical trials of vitamin D supplementation failed to demonstrate significant benefits ~~on~~for cardiovascular disease, casting doubt on the evidence from observational studies linking vitamin D deficiency with cardiovascular disease [10, 11]. This ambiguity highlights challenges in assessing the association between vitamin D and aging. One~~A~~ major limitation of previous studies is that they relied on chronological age which does not always reflect an individual's ~~is quite discrepant with~~ biological age. Indeed, unlike chronological age which simply counts the years lived~~is a precise reflection of an individual's lifetime~~, biomarkers making up

Field Code Changed

Field Code Changed

Field Code Changed

Field Code Changed

Field Code Changed

Field Code Changed

Field Code Changed

Field Code Changed

the biological age are mainly determined by a combination of genetic, environmental and lifestyle factors [12]. ConsequentlyAccordingly, some individuals may age biologically faster or slower than indicated by their chronological age.

To better characterize physiological aging in epidemiological studies, several aging metrics have been developed that combine clinical markers based on the combination of clinical (e.g., PhenoAge and GrimAge) and biological (PhenoAge acceleration) markers have been developed [13]. In a post hoc analysis of the European DO-HEALTH randomized clinical trial involving 777 participants with a mean 25(OH)D serum level of 56.785 nmol/L, PhenoAge was the only biological age metric shown to be slowed by daily vitamin D (2000 UI) supplementation among the four biological age metrics that were assessed: PhenoAge, GrimAge, GrimAge2 and DunedinPACE [14]. PhenoAgeAccel was developed and validated using machine learning methods on a National Health and Nutrition Examination Survey (NHANES) dataset of 4000 adults aged 20 to 84 years, demonstrating a mean absolute error of approximately 3.6 years when predicting chronological age and it showed a mean absolute error of approximately 3.6 years when predicting the chronological age [15]. Although vitamin D supplementation has beenwas shown to slow the increase of PhenoAgeAccel the PhenoAgeAccel value in older adults with low 25(OH)D serum concentrations, observational studies linking 25(OH)D serum concentrations with PhenoAgeAccel are lackinglimited. Therefore, this study aims to investigate whether in this study, we assessed whether there is a linear and/or non-linear relationship between 25(OH)D serum concentration and PhenoAgeAccel, using datasets of 2007-2016 NHANES surveys. By exploring this relationshipassociation, the study seeks to improve aims to enhance our understanding of the link between serum vitamin D levels and biological aging in human beings, with potential implications for public health. for the benefit of public health.

Field Code Changed

Field Code Changed

Field Code Changed

Field Code Changed

Formatted: Font: (Default) Times New Roman

Formatted: Normal, Line spacing: 1.5 lines, Don't adjust space between Latin and Asian text, Don't adjust space between Asian text and numbers

~~2.~~ Materials and Methods

~~2.1~~ Study design and population

This study was conducted and reported in agreement with STROBE guidelines for reporting observational studies. This study utilized data derived from the 2007-2016 NHANES conducted in the United States of America by the National Center for Health Statistics of the Centers for Disease Control and Prevention. All data included in the current study are publicly available on the NHANES website [16]. NHANES includes in-home interview, physical measurements and laboratory tests. The 2007-2016 NHANES datasets involved 50558 adult participants. P~~p~~articipants with incomplete information and participants aged less than 40 years old were excluded. After applying the exclusion criteria~~excluding~~, a total of 13,910 participants were finally included in this analysis (Figure 1).

~~2.2~~ Measurement of 25(OH)D serum level

Vitamin D serum levels were reflected by 25(OH)D. Because the forms of 25(OH)D measured in the 2007-2016 NHANES samples included both 25(OH)D2 and 25(OH)D3 (classic 25(OH)D3 and C3 epimer of 25(OH)D3 [C3-epi-25(OH)D3]), serum 25(OH)D included 25(OH)D2, 25(OH)D3 and C3-epi-25(OH)D3 in this study. During the study period (2007-2016), 25(OH)D levels were measured in the NHANES population using the liquid chromatography-tandem mass spectrometry (LC-MS/MS). According to the 2011 Institute of Medicine's Committee recommendations, we transformed serum 25(OH)D concentrations into a categorical variable with the following modalities: deficiency (<30 nmol/L), insufficiency (30–50 nmol/L), sufficiency (50–75 nmol/L) and abnormally high (≥75 nmol/L)[17].

~~2.3~~ Measurement of PhenoAge

In this study, we utilized PhenoAge as the indicator of biological aging, considering its high robustness and feasibility in NHANES data as a reliable predictor of mortality and age-related disease risks. The PhenoAgeAccel algorithm was

Field Code Changed

Field Code Changed

constructed by applying elastic-net regression methods to NHANES III (1988-1994) datasets, encompasses 10 markers: serum albumin, serum creatinine, plasma glucose, white blood cell count, blood lymphocyte percent, red cell distribution width, mean red cell volume, serum C-reactive protein, serum alkaline phosphatase and chronological age [18]. All constituent biomarkers of PhenoAge were routinely measured during NHANES using standardized protocols ensuring reproducibility across study cycles. One of the markers, CRP, was unavailable from 2011 to 2018 datasets. However, p~~P~~revious studies have applied an algorithm that excluded CRP ~~to~~ when calculat~~e~~ing the PhenoAge, demonstrating good applicability [19, 20]. To assess the validity of ~~using~~ PhenoAge without CRP data, we calculated 2 PhenoAge estimates with and without C-reactive protein, using data from cycles 2007-2008 and 2009-2010, which contained all 10 markers. We found a strong association between 2 PhenoAge estimates with and without CRP (Pearson's correlation coefficient of 0.986). Based on this finding, we proceeded with the main analysis of data from the five NHANES cycles using the PhenoAge algorithm excluding CRP. We further calculated PhenoAgeAccel as the difference between PhenoAge and chronological age. A positive value of PhenoAgeAccel represents accelerated biological aging process, while a negative value represented slower biological aging process. Subsequently, PhenoAgeAccel was standardized to have a mean of 0 and a standard deviation (SD) of 1.

Field Code Changed

Field Code Changed

~~2.4~~ Covariates

NHANES interviews were conducted by trained interviewers. Questionnaires were administered to participants to collect information on demographics (chronological age, gender, race, education level, poverty income ratio (PIR) and lifestyle-related factors (tobacco smoking, alcohol consumption, physical activity, and comorbidities) and~~.~~ t~~T~~he body mass index (BMI). These variables were measured at the Mobile Examination Center.

Covariates in regression analyses testing the association between 25(OH)D serum levels and biological aging include age, race, education level, PIR, BMI, tobacco

smoking, alcohol consumption, physical activity. The variable active tobacco smoking was classified into three categories: never smokers (previous smoking of a total of less than 100 cigarettes), current smokers (lifetime smoking of over 100 cigarettes) and former smokers (had smoked ⩾100 cigarettes in their lifetime but had quit smoking at the time of the survey). The variable alcohol consumption was classified as never drinkers (participants who had drunk <12 drinks in their lifetime), current drinkers (participants who had drunk ⩾12 drinks in their lifetime and at least one drink in the past year), and former drinkers (participants who had drunk ⩾12 drinks in their lifetime but no drink in the past year). A Metabolic Equivalent task (MET)-min per week were calculated to assess physical activity, levels (in min per week) that were categorized as: inactive (no regular physical activity), insufficient (<500 MET-min activity/week), moderate (500-1,000 MET-min activity/week) and high (⩾1,000 MET-min activity/week) [21]. For sleep duration, participants responded to the question: "How much sleep do you usually get at night on weekdays or on workdays?" Sleep duration was categorized into 3 groups: <7 h, 7-8 h and ⩾8h [22]). The definition of hypertension was based on self-reports, the use of antihypertensive drugs or on the record of a systolic blood pressure⩾140 mmHg with or without a diastolic blood pressure⩾90 mmHg on physical exam [23]. The definition of diabetes mellitus was based on self-reports or on laboratory test results showing fasting blood glucose levels ⩾126 mg/dL or HbA1c values ⩾6.5% [24].

Field Code Changed

Field Code Changed

Field Code Changed

Field Code Changed

~~2.5~~ Statistical analysis

Weighted multivariable linear regression models were constructed to estimate the association between serum 25(OH)D status and PhenoAgeAccel. 25(OH)D serum levels were log-transformed due to their skewed distribution. To evaluate whether the association between 25(OH)D serum levels and PhenoAgeAccel was not just linear but also dose-dependent, 25(OH)D serum level was analyzed as both a continuous (log-

transformed) and a categorical variable. In agreement with standards for linear regression analyses involving continuous dependent variables, the association between 25(OH)D serum levels as continuous variables and PhenoAgeAccel was estimated based on beta coefficients with their 95% confidence intervals [25]. All models were adjusted for chronological age, gender, race, education level, PIR, BMI, tobacco smoking and alcohol consumption variable, physical activity, sleep duration, hypertension, diabetes and ~~season~~ climate of measurement. To evaluate potential effect modification by gender and other key covariates, subgroup analyses and interaction tests were conducted. Interaction term of serum 25(OH)D and stratifying factor were included in the multivariable linear regression models to test for significance of interaction.

To explore whether there is also a non-linear relationship between 25(OH)D serum levels and PhenoAgeAccel, a Generalized Additive Model (GAM) was employed to fit the smooth curve. The GAMs extend traditional linear regression by replacing linear terms with smooth functions, allowing flexible modeling of complex dose-response relationships without assuming a priori parametric forms [26]. The threshold effect was further estimated using segmented regression model. This method identifies inflection points where the slope of the association between 25(OH)D serum levels and PhenoAgeAccel changes significantly. The optimal breakpoint was determined via likelihood ratio tests, comparing the goodness-of-fit between the non-segmented linear model and the segmented regression model [27]. Results ~~of~~ with *P*-values ~~of~~ <0.05 were considered to be statistically significance~~-~~. All data analyses were performed using R software version 4.2.1 (R Core Team, Vienna, Austria). R package ‘BioAge’ was used to calculate PhenoAge [15], while the package ‘mgcv’ was used to conduct GAM analysis [28]. Continuous data were reported as means ± standard deviations (SDs) for normal distributions or as medians (interquartile ranges) for skewed distributions. Categorical data were reported as numbers (percentages).

~~2.6~~ Ethical considerations

Field Code Changed

Field Code Changed

Field Code Changed

Field Code Changed

Field Code Changed

This study utilized data from NHANES study, which was conducted in accordance with the principles in the Declaration of Helsinki. The NHANES protocol received prior ethical approval from the National Center for Health Statistics' Review Board. Since our study is a secondary analysis of publicly accessible, de-identified NHANES data sets, additional institutional review board approval was not required [29].

Field Code Changed

~~3.~~ Results

~~3.1~~ Characteristics of the study population

The current study included a total of 6836 males and 7074 females, with the characteristics of the study population presented in Table 1. Significant differences were observed~~There are significant differences~~ between males and females in terms of ~~regarding the distributions of~~ age, race, BMI, and poverty-income ratio (p < 0.001). Additionally, gender-specific differences were noted in ~~terms of~~ behaviors influencing biological aging, such as smoking, drinking, physical activity, and sleep duration (p < 0.001). Notably, males had a higher prevalence of diabetes mellitus than females. The median 25(OH)D serum level in females was 66.6 nmol/L compared with 63.8 nmol/L in males (p<0.001). The PhenoAgeAccel of males and females were -2.40 (-5.13, 0.94) and -4.14 (-7.22, -0.46) respectively, suggesting that PhenoAgeAccel was lower in females than in males.

~~3.2~~ Association between 25(OH)D serum level and biological aging

The associations between 25(OH)D serum levels and PhenoAgeAccel were presented in Table 2. After adjusting for age, race, education level, PIR, BMI, smoking, alcohol consumption, level of physical, sleep duration and presence of hypertension and/or diabetes mellitus, –25(OH)D serum levels were negatively associated with PhenoAgeAccel (β=-0.04 SD; 95% CI: -0.08 to 0.00). When stratified by sex~~Segregating this data by sex~~, the association was statistically significant only in females (p=0.007). Further analysis dividing serum 25(OH)D into quartiles revealed an inverse relationship with PhenoAgeAccel, although no clear dose-response trend was

observed ~~When 25(OH)D serum levels were subdivided into quartiles, 25(OH)D serum levels were inversely associated with PhenoAgeAccel, with no apparent dose-dependent trend~~ (p for trend =0.173). When segregating the data by sex, 25(OH)D serum levels were inversely associated with PhenoAgeAccel only in women, with no apparent dose-response relationship (p for trend = 0.076). ~~When c~~Categorizing 25(OH)D serum levels according to ~~as recommended in~~ the 2011 USA institute of Medicine guidelines, ~~25(OH)D serum levels were~~ inverse~~ly~~ associat~~ed~~ion with PhenoAgeAccel were observed for levels~~for 25(OH)D serum levels~~ between 30-50 nmol/L and 50-75 nmol/L in males (without ~~no~~ a ~~apparent~~clear dose-response ~~relationship~~trend), and between 50-75 nmol/L and ≥75 nmol/L in females, where a dose-response relationship was apparent ~~with an apparent dose-response relationship~~ (p=0.014). Interaction test confirmed that association between serum 25(OH)D and PhenoAgeAccel was modified by gender, regardless of whether 25(OH)D was modeled as a continuous variable or categorized using quartiles or IOM guidelines (all p for interaction <0.001).

Results of subgroup/effect modification analysis showed that in males higher 25(OH)D serum levels were associated with ~~negative~~ reduc~~ed~~tion in PhenoAgeAccel primarily among~~only in~~ individuals~~people~~ aged over 60 years, those with hypertension and those with diabetes mellitus, suggesting that there were statistically significant interactions between 25(OH)D serum levels and age, hypertension, and diabetes (Fig.2A). In females, ~~we did not find any~~no effect modifiers were identified in~~for~~ the association between 25(OH)D serum levels and PhenoAge Accel (Fig.2B).

~~3.3~~ Association of 25(OH)D serum levels with PhenoAgeAccel on GAM

There was no linear association between serum 25(OH)D levels and PhenoAgeAccel when dividing the levels into quartiles ~~Given the lack of linear associations between 25(OH)D serum levels and PhenoAgeAccel when subdividing 25(OH)D serum levels into quartiles~~ (Table 2)~~,~~. Result~~e~~s from GAM ~~was utilized to~~

~~investigate the potential linkage between serum 25(OH)D and PhenoAgeAccel.~~confirmed ~~that t~~~~There was~~ a nonlinear ~~relationship~~association between 25(OH)D serum level and PhenoAgeAccel in both males and females (p<0.001) (Fig 3 A-B). In males~~en~~, the relationship was U-shaped with the two inflection points at 38.2 nmol/L and 91.5 nmol/L~~, while and in females women, the segmented regression modelsling confirmed an L-shaped relationship~~. Specifically, ~~L~~low 25(OH)D serum levels (<38.2 nmol/L) were negatively related to PhenoAgeAccel (β=-0.013 SD; 95% CI: -0.025 to -0.002), whereas high levels (>91.55 nmol/L) showed a positive association (β=0.005 SD; 95% CI: 0.002 to 0.008). In females, segmented regression models indicated an L-shaped relationship.~~ For females,~~ 25(OH)D serum levels below 62.5 nmol/L were negatively linked to PhenoAgeAccel (β=-0.007 SD; 95% CI: -0.01 to -0.004), ~~with~~ but no significant association observed beyond 125 nmol/L (β=-0.001 SD; 95% CI: -0.005 to -0.002). Overall, low (<38.2 nmol/L) or high (>91.~~55~~6 nmol/L) 25(OH)D serum levels in males were associated with PhenoAgeAccel, while in females, only low levels (<62.5 nmol/L) ~~was~~ were associated with ~~for~~ PhenoAgeAccel (Table 3).

~~4.~~ Discussion

In the current study, the association between 25(OH)D serum level and biological aging was investigated in the 2007-2016 NHANES surveys. The analysis revealed a negative association between 25(OH)D serum levels and PhenoAgeAccel. Further ~~The~~ effect modifier analysis showed that this association was influenced by age above 60 years, diabetes mellitus and hypertension~~ modified the association between 25(OH)D serum levels and PhenoAge in men~~, however, no such modifiers were identified for females~~but no modifier was found for that association in females~~. When subdividing 25(OH)D serum levels into quartiles and stratifying data by sex, this association was still observed only in females, and it exhibited ~~but in~~ a non-linear pattern~~fashion~~. Additionally, ~~W~~when further subdividing 25(OH)D serum levels according to ~~as recommended by~~ the Institute of Medicine in USA in 2011, the association persisted

for 25(OH)D serum levels between 30-50 nmol/L and between 50-75 nmol/L in males and between 50-75 nmol/L and ≥75 nmol/L, ~~, ~~with a linear pattern of relationship observed only in females. Non-linear associations were also found between 25(OH)D serum levels and PhenoAgeAccel in both males (U-shaped) and females (L-shaped).

Previous studies have reported that a gradual decline in 25(OH)D serum levels with chronological age, and vitamin D supplementation can mitigate oxidative damage and improve immune function in elder people, suggesting its potential role in slowing age-related physiological decline [30]. This decline in vitamin D levels is attributed to multiple ~~several ~~factors, including decreased sun exposure, reduced capacity of the skin to synthesize vitamin D, and decreased dietary intake of vitamin D, all of which contribute to ~~resulting in ~~vitamin D deficiency in older adults [31]. The synthesis of vitamin D from sun exposure vary in different races, but our study lacks this data of sun exposure. Additionally, we also found that low 25(OH)D serum levels were associated with positive PhenoAgeAccel, suggesting vitamin D may~~ also~~ play a crucial role in the regulation of the aging process. Adequate 25(OH)D levels are essential for maintaining calcium absorption and bone health preventing osteoporosis, a common age-related condition, and regulating calcium homeostasis and bone metabolism [32]. Beyond these functions~~Moreover~~, vitamin D can interact with antioxidant and anti-inflammatory pathways, such as activating Nrf2 (nuclear factor erythroid 2-related factor 2) and SIRT1 (sirtuin 1) signaling cascades, and suppressing the production of pro-inflammatory cytokines, including interleukin-6 (IL-6) and tumor necrosis factor-alpha (TNF-α), through inhibiting nuclear factor-κB (NF-κB). These actions ~~lead to reduction of ~~help reduce inflammation and oxidative stress, both of which are known~~ to~~ contribut~~e~~ors to the aging process [32, 33]. Consequently,~~Therefore,~~ vitamin D and aging ~~have a mutual ~~influence ~~on ~~each other mutually.

Vitamin D supplementation has been shown to~~could~~ be an effective way to reverse or mitigate diseases, including lower blood pressure and improve endothelial function, leading to reduced risk of cardiovascular events, such as heart attacks and strokes, and

Field Code Changed

Field Code Changed

Field Code Changed

Field Code Changed

improved cardiovascular outcomes [34]. A meta-analysis of 75 randomized controlled trials in 2014 reported ~~revealed~~ that vitamin D supplementation was associated with a 6% reduction in all-cause mortality [35]. Notably, the impact of vitamin D supplementation on mortality appears more significant in certain subgroups~~Some studies suggest that the impact of vitamin D supplementation on all-cause mortality may be more pronounced in certain subgroups~~, such as older adults or individuals with vitamin D deficiency. For instance~~example~~, vitamin D supplementation was associated with a reduced risk of all-cause mortality in trials where ~~with~~ a mean baseline 25(OH)D level ~~<~~were below 75 nmol/L (RR 0.89, 95% CI 0.80-0.99), but not in trials with a mean baseline 25(OH)D level ≥75 nmol/L (RR 1.04, 95% CI 0.97-1.11) [36]. Thus, these evidence highlights the potential of vitamin D supplementation in reducing the risk of several chronic diseases, particularly in the elderly~~ people~~, in whom ~~where~~ serum vitamin D levels tend to decline with age.

Despite its potential benefits, the use of vitamin D supplements has increased markedly, raising concerns ~~and concerns~~ about the risk of excessive intake~~ have emerged~~. ~~t~~The ~~V~~vitamin D and Omega-3 Trial (VITAL) study, a randomized trial involving over 25,000 participants, discovered that vitamin D supplementation did not significantly reduce the risk of all-cause mortality or lower risk of fractures among generally healthy midlife and older adults without vitamin D deficiency or osteoporosis [37]. Furthermore, increased intake of vitamin D supplements by the general population and a growing number of high-dose prescriptions without medical monitoring might result in a greater risk of excessive vitamin D toxicity [38]. Our current results indicated that the association of 25(OH)D serum level and biological aging followed a U-shaped curve in males and an L-shaped curve in females. This suggests ~~, revealing~~ that high serum level of 25(OH)D is not associated with slower aging and may, in fact, be linked to ~~decelerated aging in either males and females, and even associated with~~ accelerated biological aging in males. These detrimental effects may stem from the toxicity of excessive vitamin D. Excessive intake can cause serum levels of its metabolites, such

- Field Code Changed
- Field Code Changed
- Field Code Changed
- Field Code Changed
- Field Code Changed

as 25(OH)D and 24,25(OH)$_2$D, to surpass the binding capacity of vitamin D binding protein, leading to an increase in free, active 1,25(OH)$_2$DWhen vitamin D intake is too high, serum levels of its metabolites [25(OH)D and 24,25(OH)$_2$D] rise beyond the binding capacity of vitamin D binding protein, leading to an increase in the free active metabolite, 1,25(OH)$_2$D. This active form reaches the VDR in the nucleus, constantly promoting gene expression that enhances calcium absorption and bone mobilization [39]. Consequently, this can result in hypercalcemia or hyperphosphatemia, which in turn suppresses initial parathyroid hormone (iPTH) level [39, 40]. Such disruptingons in balance of calcium balance and hormone regulation may contribute to cardiovascular disease, kidney damage, and skin aging. Research reported that the working diagnosis of vitamin D toxicity is diagnosed based on total 25(OH)D concentrations of 80~200nmol/L detected by immunoassay [41]. Our findings that Our results found the 25(OH)D levels over 91.5 nmol/L in male participants were not linked to accelerated biological aging, are approximately consistent with those reports.

Our study identified a transition point in the relationship between 25(OH)D serum level and biological aging, with males exhibiting a threshold at 38.20 nmol/L and females at 62.50 nmol/L. Beyond this transition point, the increase in 25(OH)D level no longer associates with biological aging deceleration. Regarding the threshold of vitamin D deficiency, it remains an ongoing controversy. The Scientific Advisory Committee on Nutrition (2016) recommended threshold at 25 nmol/L among UK population [42], whereas the IOM (2011) suggested a thresholds of at 30 nmol/L based on evidence in U.S. and Canadian populations [17]. Meanwhile, the European Food Safety Authority 2016 and the Endocrine Society guideline 2011 proposed higher level of 50 nmol/L as deficiency cutoff [43, 44]. The discrepancy in cutoff values stems from multiple factors, including for example, different regional ultraviolet B exposure patterns, dietary practice and population-specific biological variability. Furthermore, guideline variations also reflect differing priorities concerning different focus on health outcomes, with some prioritizing musculoskeletal health [45]. Notably, Iin 2024, the Endocrine Society guideline retracted

Field Code Changed
Field Code Changed
Field Code Changed
Field Code Changed
Field Code Changed
Field Code Changed
Field Code Changed

their previous recommendation of threshold at 50 nmol/L due to limited clinical trial evidence [46]. They ~~It~~ also highlighted the impracticality of universal thresholds due to heterogeneity in health outcomes and population characteristics. Therefore~~Hence~~, the interpretation of our findings regarding 25(OH)D level threshold ~~determination in our study~~ ~~s~~should be with caution, especially ~~when~~ since it derived from observational study in an ethnically diverse population. Nevertheless, the trend of benefits attenuation we observed is worth noting, suggesting excessive level of serum 25(OH)D may not be beneficial regarding biological aging process.

Our results revealed sex-specific ~~reported~~ differences of dose-response relationship between serum 25(OH)D levels and biological aging, ~~curve between in male and in female,~~ which may be attributed to the sex hormones, particularly ~~especially~~ estrogen. As women age, especially ~~particularly~~ during the menopausal transition and after menopause, their ovaries produce less estrogen,~~,~~ leading to a decline of estrogen levels. This decline contributes to the thinning and wrinkling of the skin, as well as changes in hair texture and growth patterns [47]. Some studies suggest that vitamin D could enhance the activity of estrogen receptors (ER) via interacting with them, potentially amplifying the anti-aging effects of estrogen in the body [48]. Additionally, ~~V~~vitamin D and estrogen also have a synergistic effect on bone metabolism, helping to prevent osteoporosis and fractures, especially in postmenopausal women [49]. ~~Additionally, v~~Vitamin D may also regulate estrogen metabolism, leading to higher circulating estrogen levels and subsequently delay aging [48]. Therefore, high level of serum 25(OH)D may not be associated with decelerated biological aging in females. However,~~,~~ ~~but~~ the mechanisms underlying the adverse effects observed at ~~of~~ extremely high levels (>100 nmol/L) in males remain unclear. Further studies are needed to clarify the complex interplay between these two important hormonal systems.

~~The current~~This study has several limitations. First, as a cross-sectional investigation, it only assesses ~~provides information on the~~ association between serum 25(OH)D levels and biological aging, making it challenging to establish the

Field Code Changed

Field Code Changed

Field Code Changed

Field Code Changed

Field Code Changed

causality~~causal relationships~~. Second, the classification of 25(OH)D serum levels used in our study is based on the 2011 IOM guidelines; however, more recent classifications, such as those from the Scientific Advisory Committee on Nutrition (2016) [50], may provide more accurate benchmarks~~are considered more accurate~~. Analyzing the relationship using these updated criteria would be preferable. ~~It would be preferable to analyze the association between vitamin D status and biological aging using these updated classifications.~~ While sun exposure and dietary intake are primary sources of vitamin D, data on these factors, as well as supplement use,~~this information was~~ were not included in our analysis due to the lack of corresponding data ~~limitations~~ in the NHANES dataset. The absence of these confounding variables may introduce residual confounding, reverse causation, and bias.~~.~~ Additionally, race significantly influences serum vitamin D levels, but the definition of race varies across NHANES data collected over different years, which posed a challenge in our study. Finally, emerging evidence questions the traditional view of serum 25(OH)D as a definitive biomarker of vitamin D status, although it remains the most widely accepted marker. For instance, in individuals with diabetes or obesity, the expression of CYP2R1—the enzyme responsible for vitamin D 25-hydroxylation—is suppressed, leading to decreased 25(OH)D levels despite stable overall vitamin D levels [51, 52].

Field Code Changed

Field Code Changed

~~5.~~ Conclusion

In this study, we investigated the relationship between 25(OH)D serum levels and PhenoAge in NHANES, and found that there was a negative association between PhenoAgeAccel and 25(OH)D serum level, and these associations were nonlinear in both genders, revealing a U-shaped curve for males and an L-shaped curve for females. Low 25(OH)D serum levels shows a potential positively link to PhenoAgeAccel in both genders, but high 25(OH)D serum levels do not associate with decelerated PhenoAge, and even associated with accelerated PhenoAge in male. Our results suggest that the association between vitamin D levels with biological aging is different in men and women.

Authors contributions

Yi Feng: Conceptualization; Methodology; Data curation; Formal analysis; Writing original draft**; Licheng Yu**: Data curation; Formal analysis; **Jue Wang**: Project administration; Supervision; Writing - review &editing.

Conflicts of interest

The authors declare no competing financial or non-financial interests related to this work. NHANES is a publicly available dataset managed by the Centers for Disease Control and Prevention. The authors confirm that there is no affiliation with or involvement from the NHANES program administrators in this study. The funding source had no role in study design, data collection, analysis, or manuscript preparation.

References

1. Bikle DD. Vitamin D metabolism, mechanism of action, and clinical applications. Chem Biol. 2014;21(3):319-29. Epub 2014/02/18. doi: 10.1016/j.chembiol.2013.12.016. PubMed PMID: 24529992; PubMed Central PMCID: PMCPMC3968073.
2. Dominguez LJ, Farruggia M, Veronese N, Barbagallo M. Vitamin D Sources, Metabolism, and Deficiency: Available Compounds and Guidelines for Its Treatment. Metabolites. 2021;11(4). Epub 2021/05/01. doi: 10.3390/metabo11040255. PubMed PMID: 33924215; PubMed Central PMCID: PMCPMC8074587.
3. Couchman L, Moniz CF. Analytical considerations for the biochemical assessment of vitamin D status. Ther Adv Musculoskelet Dis. 2017;9(4):97-104. Epub 2017/04/07. doi: 10.1177/1759720X17692500. PubMed PMID: 28382113; PubMed Central PMCID: PMCPMC5367642.
4. Rahman A, Elmi A. Air pollutants are negatively associated with vitamin D-synthesizing UVB radiation intensity on the ground. Sci Rep. 2021;11(1):21480. Epub 2021/11/04. doi: 10.1038/s41598-021-00980-6. PubMed PMID: 34728744; PubMed Central PMCID: PMCPMC8563978.
5. Zhao S, Qian F, Wan Z, Chen X, Pan A, Liu G. Vitamin D and major chronic diseases. Trends Endocrinol Metab. 2024. Epub 2024/06/02. doi: 10.1016/j.tem.2024.04.018. PubMed PMID: 38824035.
6. Gaksch M, Jorde R, Grimnes G, Joakimsen R, Schirmer H, Wilsgaard T, et al. Vitamin D and mortality: Individual participant data meta-analysis of standardized 25-hydroxyvitamin D in 26916 individuals from a European consortium. Plos One. 2017;12(2):e0170791. Epub 2017/02/17. doi: 10.1371/journal.pone.0170791. PubMed PMID: 28207791; PubMed Central PMCID: PMCPMC5312926.

Field Code Changed

Formatted: Font: (Default) Times New Roman

7. Fang A, Zhao Y, Yang P, Zhang X, Giovannucci EL. Vitamin D and human health: evidence from Mendelian randomization studies. Eur J Epidemiol. 2024;39(5):467-90. Epub 2024/01/12. doi: 10.1007/s10654-023-01075-4. PubMed PMID: 38214845.

8. Cui A, Xiao P, Ma Y, Fan Z, Zhou F, Zheng J, et al. Prevalence, trend, and predictor analyses of vitamin D deficiency in the US population, 2001-2018. Front Nutr. 2022;9:965376. Epub 2022/10/21. doi: 10.3389/fnut.2022.965376. PubMed PMID: 36263304; PubMed Central PMCID: PMCPMC9573946.

9. Berridge MJ. Vitamin D deficiency accelerates ageing and age-related diseases: a novel hypothesis. The Journal of physiology. 2017;595(22):6825-36. Epub 2017/09/28. doi: 10.1113/JP274887. PubMed PMID: 28949008; PubMed Central PMCID: PMCPMC5685827.

10. Zittermann A, Ernst JB, Prokop S, Fuchs U, Dreier J, Kuhn J, et al. Effect of vitamin D on all-cause mortality in heart failure (EVITA): a 3-year randomized clinical trial with 4000 IU vitamin D daily. Eur Heart J. 2017;38(29):2279-86. Epub 2017/05/13. doi: 10.1093/eurheartj/ehx235. PubMed PMID: 28498942.

11. Scragg R, Stewart AW, Waayer D, Lawes CMM, Toop L, Sluyter J, et al. Effect of Monthly High-Dose Vitamin D Supplementation on Cardiovascular Disease in the Vitamin D Assessment Study : A Randomized Clinical Trial. JAMA Cardiol. 2017;2(6):608-16. Epub 2017/04/07. doi: 10.1001/jamacardio.2017.0175. PubMed PMID: 28384800; PubMed Central PMCID: PMCPMC5815022 ICMJE Form of Disclosure of Potential Conflicts of Interest and none were reported.

12. Xia X, Wang Y, Yu Z, Chen J, Han JJ. Assessing the rate of aging to monitor aging itself. Ageing Res Rev. 2021;69:101350. Epub 2021/05/04. doi: 10.1016/j.arr.2021.101350. PubMed PMID: 33940202.

13. Jylhava J, Pedersen NL, Hagg S. Biological Age Predictors. EBioMedicine. 2017;21:29-36. Epub 2017/04/12. doi: 10.1016/j.ebiom.2017.03.046. PubMed PMID: 28396265; PubMed Central PMCID: PMCPMC5514388.

14. Bischoff-Ferrari HA, Gangler S, Wieczorek M, Belsky DW, Ryan J, Kressig RW, et al. Individual and additive effects of vitamin D, omega-3 and exercise on DNA methylation clocks of biological aging in older adults from the DO-HEALTH trial. Nat Aging. 2025;5(3):376-85. Epub 2025/02/04. doi: 10.1038/s43587-024-00793-y. PubMed PMID: 39900648.

15. Kwon D, Belsky DW. A toolkit for quantification of biological age from blood chemistry and organ function test data: BioAge. Geroscience. 2021;43(6):2795-808. Epub 2021/11/03. doi: 10.1007/s11357-021-00480-5. PubMed PMID: 34725754; PubMed Central PMCID: PMCPMC8602613.

16. Centers for Disease Control and Prevention National Health and Nutrition Examination Survey Questionnaires D, and Related Documentation 2020. ~~[cited 2025 March 7].~~ Available from: https://wwwn.cdc.gov/nchs/nhanes/.

17. Ross AC, Taylor CL, Yaktine AL, Del Valle HB. Dietary Reference Intakes for Calcium and Vitamin D. The National Academies Collection: Reports funded by National Institutes of Health. Washington (DC)2011.

18. Liu Z, Kuo PL, Horvath S, Crimmins E, Ferrucci L, Levine M. A new aging measure captures morbidity and mortality risk across diverse subpopulations from NHANES IV: A cohort study. PLoS Med. 2018;15(12):e1002718. Epub 2019/01/01. doi: 10.1371/journal.pmed.1002718. PubMed PMID: 30596641; PubMed Central PMCID: PMCPMC6312200.

19. Liu W, Wang J, Wang M, Hou H, Ding X, Ma L, et al. Oxidative Stress Factors Mediate the Association Between Life's Essential 8 and Accelerated Phenotypic Aging: NHANES 2005-2018. J Gerontol A Biol Sci Med Sci. 2024;79(1). Epub 2023/10/10. doi: 10.1093/gerona/glad240. PubMed

Formatted: Font: (Default) Times New Roman

Formatted: Font: (Default) Times New Roman

PMID: 37813096.

20. He H, Chen X, Ding Y, Chen X, He X. Composite dietary antioxidant index associated with delayed biological aging: a population-based study. Aging (Albany NY). 2024;16(1):15-27. Epub 2024/01/04. doi: 10.18632/aging.205232. PubMed PMID: 38170244; PubMed Central PMCID: PMCPMC10817368.

21. Haskell WL, Lee IM, Pate RR, Powell KE, Blair SN, Franklin BA, et al. Physical activity and public health: updated recommendation for adults from the American College of Sports Medicine and the American Heart Association. Circulation. 2007;116(9):1081-93. Epub 2007/08/03. doi: 10.1161/CIRCULATIONAHA.107.185649. PubMed PMID: 17671237.

22. Chaput JP, McNeil J, Despres JP, Bouchard C, Tremblay A. Seven to eight hours of sleep a night is associated with a lower prevalence of the metabolic syndrome and reduced overall cardiometabolic risk in adults. PLoS One. 2013;8(9):e72832. Epub 2013/09/17. doi: 10.1371/journal.pone.0072832. PubMed PMID: 24039808; PubMed Central PMCID: PMCPMC3764138.

23. Muntner P, Hardy ST, Fine LJ, Jaeger BC, Wozniak G, Levitan EB, et al. Trends in Blood Pressure Control Among US Adults With Hypertension, 1999-2000 to 2017-2018. JAMA. 2020;324(12):1190-200. Epub 2020/09/10. doi: 10.1001/jama.2020.14545. PubMed PMID: 32902588; PubMed Central PMCID: PMCPMC7489367 consulting fees from Amgen Inc. Dr Levitan reported receiving grant funding from and serving on advisory boards for Amgen Inc; and serving as a consultant to Novartis. Dr Colantonio reported receiving grant funding from Amgen Inc. No other disclosures were reported.

24. American Diabetes A. Diagnosis and classification of diabetes mellitus. Diabetes Care. 2014;37 Suppl 1:S81-90. Epub 2013/12/21. doi: 10.2337/dc14-S081. PubMed PMID: 24357215.

25. Bender R. Introduction to the use of regression models in epidemiology. Cancer epidemiology: Springer; 2009. p. 179-95.

26. Hastie TJ. Generalized additive models. Statistical models in S. 2017:249-307.

27. Muggeo VM. Estimating regression models with unknown break-points. Stat Med. 2003;22(19):3055-71. Epub 2003/09/16. doi: 10.1002/sim.1545. PubMed PMID: 12973787.

28. Wood SN. Stable and efficient multiple smoothing parameter estimation for generalized additive models. Journal of the American Statistical Association. 2004;99(467):673-86.

29. World Medical A. World Medical Association Declaration of Helsinki: Ethical Principles for Medical Research Involving Human Participants. JAMA. 2025;333(1):71-4. Epub 2024/10/19 20:42. doi: 10.1001/jama.2024.21972. PubMed PMID: 39425955.

30. Schottker B, Hagen L, Zhang Y, Gao X, Holleczek B, Gao X, et al. Serum 25-Hydroxyvitamin D Levels as an Aging Marker: Strong Associations With Age and All-Cause Mortality Independent From Telomere Length, Epigenetic Age Acceleration, and 8-Isoprostane Levels. J Gerontol A Biol Sci Med Sci. 2019;74(1):121-8. Epub 2018/10/30. doi: 10.1093/gerona/gly253. PubMed PMID: 30371905.

31. Liu L, Chen C, Lo K, Huang J, Yu Y, Huang Y, et al. Serum 25-hydroxyvitamin D, frailty, and mortality among the Chinese oldest old: Results from the CLHLS study. Nutr Metab Cardiovasc Dis. 2021;31(9):2707-15. Epub 2021/08/06. doi: 10.1016/j.numecd.2021.05.033. PubMed PMID: 34348876.

32. Veldurthy V, Wei R, Oz L, Dhawan P, Jeon YH, Christakos S. Vitamin D, calcium homeostasis and aging. Bone Res. 2016;4:16041. Epub 2016/10/30. doi: 10.1038/boneres.2016.41. PubMed PMID: 27790378; PubMed Central PMCID: PMCPMC5068478.

33. Ge D, Chen Q, Xie X, Li Q, Yang Y. Unveiling the potent effect of vitamin D: harnessing Nrf2/HO-1 signaling pathways as molecular targets to alleviate urban particulate matter-induced asthma inflammation. BMC Pulm Med. 2024;24(1):55. Epub 2024/01/26. doi: 10.1186/s12890-024-02869-2. PubMed PMID: 38273268; PubMed Central PMCID: PMCPMC10809564.

34. Pilz S, Gaksch M, Kienreich K, Grubler M, Verheyen N, Fahrleitner-Pammer A, et al. Effects of vitamin D on blood pressure and cardiovascular risk factors: a randomized controlled trial. Hypertension. 2015;65(6):1195-201. Epub 2015/03/25. doi: 10.1161/HYPERTENSIONAHA.115.05319. PubMed PMID: 25801871.

35. Chowdhury R, Kunutsor S, Vitezova A, Oliver-Williams C, Chowdhury S, Kiefte-de-Jong JC, et al. Vitamin D and risk of cause specific death: systematic review and meta-analysis of observational cohort and randomised intervention studies. BMJ. 2014;348:g1903. Epub 2014/04/03. doi: 10.1136/bmj.g1903. PubMed PMID: 24690623; PubMed Central PMCID: PMCPMC3972416.

36. Rejnmark L, Bislev LS, Cashman KD, Eiriksdottir G, Gaksch M, Grubler M, et al. Non-skeletal health effects of vitamin D supplementation: A systematic review on findings from meta-analyses summarizing trial data. Plos One. 2017;12(7):e0180512. Epub 2017/07/08. doi: 10.1371/journal.pone.0180512. PubMed PMID: 28686645; PubMed Central PMCID: PMCPMC5501555.

37. LeBoff MS, Chou SH, Ratliff KA, Cook NR, Khurana B, Kim E, et al. Supplemental Vitamin D and Incident Fractures in Midlife and Older Adults. N Engl J Med. 2022;387(4):299-309. Epub 2022/08/09. doi: 10.1056/NEJMoa2202106. PubMed PMID: 35939577; PubMed Central PMCID: PMCPMC9716639.

38. Pludowski P, Grant WB, Karras SN, Zittermann A, Pilz S. Vitamin D Supplementation: A Review of the Evidence Arguing for a Daily Dose of 2000 International Units (50 microg) of Vitamin D for Adults in the General Population. Nutrients. 2024;16(3). Epub 2024/02/10. doi: 10.3390/nu16030391. PubMed PMID: 38337676; PubMed Central PMCID: PMCPMC10857599.

39. Marcinowska-Suchowierska E, Kupisz-Urbanska M, Lukaszkiewicz J, Pludowski P, Jones G. Vitamin D Toxicity-A Clinical Perspective. Front Endocrinol (Lausanne). 2018;9:550. Epub 2018/10/09. doi: 10.3389/fendo.2018.00550. PubMed PMID: 30294301; PubMed Central PMCID: PMCPMC6158375.

40. Batman A, Saygili ES, Yildiz D, Sen EC, Erol RS, Canat MM, et al. Risk of hypercalcemia in patients with very high serum 25-OH vitamin D levels. Int J Clin Pract. 2021;75(7):e14181. Epub 2021/03/25. doi: 10.1111/ijcp.14181. PubMed PMID: 33759301; PubMed Central PMCID: PMCPMC8250214.

41. Sajid U, Orton D, Kaufmann M, Jones G, Kline GA. Understanding Elevated Vitamin D Measurements to Uncover Hypercalcemia Etiology. Clin Chem. 2024;70(6):798-802. Epub 2024/06/03. doi: 10.1093/clinchem/hvae044. PubMed PMID: 38825340.

42. England PH. SACN vitamin D and health report 2016 [updated 2025-04-22]. Available from: https://www.gov.uk/government/publications/sacn-vitamin-d-and-health-report.

43. Turck D, Bresson J, Burlingame B. Scientific opinion on dietary reference values for vitamin D EFSA Panel on Dietetic Products, Nutrition, and Allergies (NDA). EFSA J. 2016;179.

44. Holick MF, Binkley NC, Bischoff-Ferrari HA, Gordon CM, Hanley DA, Heaney RP, et al. Evaluation, treatment, and prevention of vitamin D deficiency: an Endocrine Society clinical practice guideline. J Clin Endocrinol Metab. 2011;96(7):1911-30. Epub 2011/06/08. doi: 10.1210/jc.2011-0385. PubMed PMID: 21646368.

45. Roth DE, Abrams SA, Aloia J, Bergeron G, Bourassa MW, Brown KH, et al. Global prevalence and disease burden of vitamin D deficiency: a roadmap for action in low- and middle-income countries. Ann N Y Acad Sci. 2018;1430(1):44-79. Epub 2018/09/19. doi: 10.1111/nyas.13968. PubMed PMID: 30225965; PubMed Central PMCID: PMCPMC7309365.

Formatted: Font: (Default) Times New Roman

Formatted: Font: (Default) Times New Roman

46. Demay MB, Pittas AG, Bikle DD, Diab DL, Kiely ME, Lazaretti-Castro M, et al. Vitamin D for the Prevention of Disease: An Endocrine Society Clinical Practice Guideline. J Clin Endocrinol Metab. 2024. Epub 2024/06/03. doi: 10.1210/clinem/dgae290. PubMed PMID: 38828931.
47. Santoro N, Roeca C, Peters BA, Neal-Perry G. The Menopause Transition: Signs, Symptoms, and Management Options. J Clin Endocrinol Metab. 2021;106(1):1-15. Epub 2020/10/24. doi: 10.1210/clinem/dgaa764. PubMed PMID: 33095879.
48. Crescioli C. The Role of Estrogens and Vitamin D in Cardiomyocyte Protection: A Female Perspective. Biomolecules. 2021;11(12). Epub 2021/12/25. doi: 10.3390/biom11121815. PubMed PMID: 34944459; PubMed Central PMCID: PMCPMC8699224.
49. McCurdy R, Jiang X, Clarkson TB, Nudy M, Schnatz PF. Vitamin D and conjugated equine estrogen: the association with coronary artery atherosclerosis in cynomolgus monkeys. Menopause. 2016;23(5):481-7. Epub 2016/02/18. doi: 10.1097/GME.0000000000000582. PubMed PMID: 26886883.
50. Jenkins DJA, Spence JD, Giovannucci EL, Kim YI, Josse RG, Vieth R, et al. Supplemental Vitamins and Minerals for Cardiovascular Disease Prevention and Treatment: JACC Focus Seminar. J Am Coll Cardiol. 2021;77(4):423-36. Epub 2021/01/30. doi: 10.1016/j.jacc.2020.09.619. PubMed PMID: 33509399.
51. Elkhwanky MS, Kummu O, Piltonen TT, Laru J, Morin-Papunen L, Mutikainen M, et al. Obesity Represses CYP2R1, the Vitamin D 25-Hydroxylase, in the Liver and Extrahepatic Tissues. JBMR Plus. 2020;4(11):e10397. Epub 2020/11/20. doi: 10.1002/jbm4.10397. PubMed PMID: 33210060; PubMed Central PMCID: PMCPMC7657391.
52. Aatsinki SM, Elkhwanky MS, Kummu O, Karpale M, Buler M, Viitala P, et al. Fasting-Induced Transcription Factors Repress Vitamin D Bioactivation, a Mechanism for Vitamin D Deficiency in Diabetes. Diabetes. 2019;68(5):918-31. Epub 2019/03/06. doi: 10.2337/db18-1050. PubMed PMID: 30833469; PubMed Central PMCID: PMCPMC6477896.

Table 1. Characteristics of the study population

Characteristics	Male (n=6836)	Female (n=7074)	*P*-value
Age (mean±SD)	57.07 (11.3)~~57.07 (11.34)~~	58.12 (11.8)~~58.12 (11.82)~~	<0.001
Race			0.001[b]
Non-Hispanic white	3249 (47.5)~~3249 (47.53)~~	3288 (46.5)~~3288 (46.48)~~	
Non-Hispanic black	1360 (19.9)~~1360 (19.89)~~	1417 (20.0)~~1417 (20.03)~~	
Others (Asian, Hispanic white, etc.) ~~()~~	2227 (32.6)~~2227 (32.58)~~	2369 (33.5)~~2369 (33.49)~~	
Education level			0.394[b]
Below high school	1847 (27.0)~~1847 (27.02)~~	1831 (25.9)~~1831 (25.88)~~	
High school or equivalent	1588 (23.2)~~1588 (23.23)~~	1597 (22.6)~~1597 (22.58)~~	
Above high school	3401 (49.8)~~3401 (49.75)~~	3646 (51.5)~~3646 (51.54)~~	
Poverty-income ratio			<0.001[b]
≤1.0	1199 (17.5)~~1199 (17.54)~~	1447 (20.5)~~1447 (20.46)~~	
1.0-3.0	2839 (41.5)~~2839 (41.53)~~	3029 (42.8)~~3029 (42.82)~~	
>3.0	2798 (40.9)~~2798 (40.93)~~	2598 (36.7)~~2598 (36.73)~~	
BMI, kg/m^2			<0.001[b]
<25	1585 (23.2)~~1585 (23.19)~~	1841 (26.0)~~1841 (26.02)~~	
25-30	2749 (40.2)~~2749 (40.21)~~	2117 (29.9)~~2117 (29.93)~~	
≥30	2502 (36.6)~~2502 (36.60)~~	3116 (44.1)~~3116 (44.05)~~	
Smoking			<0.001[b]
Never smoker	2823 (41.3)~~2823 (41.30)~~	4297 (60.7)~~4297 (60.74)~~	
Previous smoker	2570 (37.6)~~2570 (37.60)~~	1666 (23.6)~~1666 (23.55)~~	
Current smoker	1443 (21.1)~~1443 (21.11)~~	1111 (15.7)~~1111 (15.71)~~	
Drinking (alcohol consumption)			<0.001[b]
Never drinker	527 (7.7)~~527 (7.71)~~	1572 (22.2)~~1572 (22.22)~~	

Formatted: Font: (Default) Times New Roman

Previous drinker	637 (9.3)~~637 (9.32)~~	1466 (20.7)~~1466 (20.72)~~	
Current drinker	5672 (83.0)~~5672 (82.97)~~	4036 (57.1)~~4036 (57.05)~~	
Level of physical activity			<0.001[b]
Inactive	1775 (26.0)~~1775 (25.97)~~	2533 (35.8)~~2533 (35.81)~~	
Insufficient	802 (11.7)~~802 (11.73)~~	1086 (15.4)~~1086 (15.35)~~	
Moderate	680 (10.0)~~680 (9.95)~~	842 (11.9)~~842 (11.90)~~	
High	3579 (52.4)~~3579 (52.36)~~	2613 (36.9)~~2613 (36.94)~~	
Sleep duration			<0.001[b]
<7 h	2572 (37.6)~~2572 (37.62)~~	2445 (34.6)~~2445 (34.56)~~	
7-8h	1858 (27.2)~~1858 (27.18)~~	1812 (25.6)~~1812 (25.61)~~	
⩾8 h	2406 (35.2)~~2406 (35.20)~~	2817 (39.8)~~2817 (39.82)~~	
Hypertension			0.839[b]
No	3007 (44.0)~~3007 (43.99)~~	3067 (43.4)~~3067 (43.36)~~	
Yes	3829 (56.0)~~3829 (56.01)~~	4007 (56.6)~~4007 (56.64)~~	
Diabetes mellitus			<0.001[b]
No	5086 (74.4)~~5086 (74.40)~~	5515 (78.0)~~5515 (77.96)~~	
Yes	1750 (25.6)~~1750 (25.60)~~	1559 (22.0)~~1559 (22.04)~~	
~~Serum~~ 25(OH)D Serum level, nmol/L			
Median (IQR)	63.8 (48.4, 80.2)~~63.80 (48.38, 80.20)~~	66.6 (47.0, 87.3)~~66.60 (47.00, 87.30)~~	<0.001[c]
<30	420 (6.1)~~420 (6.14)~~	545 (7.7)~~545 (7.70)~~	<0.001[b]
30-50	1451 (21.2)~~1451 (21.23)~~	1457 (20.6)~~1457 (20.60)~~	
50-75	2766 (40.5)~~2766 (40.46)~~	2293 (32.4)~~2293 (32.41)~~	
⩾75	2199 (32.2)~~2199 (32.17)~~	2779 (39.3)~~2779 (39.28)~~	

PhenoAge (median [IQR])	53.8 (45.1, 64.6)~~53.77 (45.07, 64.60)~~	53.1 (44.2, 64.1)~~53.05 (44.23, 64.13)~~	0.032
PhenoAgeAccel (median [IQR])	-2.4 (-5.1, 0.9)~~-2.40 (-5.13, 0.94)~~	-4.1 (-7.2, -0.5)~~-4.14 (-7.22, -0.46)~~	<0.001[c]
Standardized PhenoAgeAccel (mean±SD)	0.1 (0.9)~~0.11 (0.95)~~	-0.2 (1.1)~~-0.16 (1.05)~~	<0.001[a]

[a] Student's t-test was used to compare means of variables between two groups

[b] Chi-square test was used to compare categorical variables between two groups.

[c] Mann-Whitney test was used to compare non-normal variables between two groups.

Table 2. Association between 25(OH)D serum level and PhenoAgeAccel

Characteristics	All			Male			Female		
	Beta coefficient (95% CI)		*P*-value	Beta coefficient (95% CI)		*P*-value	Beta coefficient (95% CI)		*P*-value
	Crude model	Adjusted model [a]		Crude model	Adjusted model [a]		Crude model	Adjusted model [a]	
Log(25(OH)D)	-0.27 (-0.32, -0.22)	-0.04 (-0.08, 0.00)	0.039	-0.13 (-0.20, -0.06)	0.03 (-0.02, 0.09)	0.334	-0.32 (-0.38, -0.26)	-0.07 (-0.12, -0.01)	0.007
Quartiles									
Q1	Reference	Reference		Reference	Reference		Reference	Reference	
Q2	-0.23 (-0.29, -0.17)	-0.10 (-0.16, -0.05)	<0.001	-0.16 (-0.24, -0.08)	-0.02 (-0.09, 0.04)	0.448	-0.33 (-0.41, -0.25)	-0.16 (-0.23, -0.08)	<0.001
Q3	-0.29 (-0.36, -0.23)	-0.10 (-0.16, -0.04)	0.001	-0.20 (-0.29, -0.11)	-0.03 (-0.12, 0.04)	0.355	-0.40 (-0.49, -0.32)	-0.15 (-0.22, -0.07)	<0.001
Q4	-0.32 (-0.38, -0.26)	-0.05 (-0.11, -0.00)	0.022	-0.16 (-0.24, -0.07)	0.02 (-0.04, 0.10)	0.553	-0.42 (-0.51, -0.34)	-0.10 (-0.18, -0.02)	0.009
P for trend			0.173			0.429			0.076
Categories based on the 2011 Institute of Medicine									
<30	Reference	Reference		Reference	Reference		Reference	Reference	
30-50	-0.23 (-0.33, -0.13)	-0.14 (-0.23, -0.05)	0.002	-0.26 (-0.41, -0.11)	-0.15 (-0.29, -0.02)	0.024	-0.22 (-0.34, -0.09)	-0.10 (-0.21, 0.00)	0.057
50-75	-0.42 (-0.52, -0.33)	-0.21 (-0.30, -0.13)	<0.001	-0.39 (-0.53, -0.25)	-0.16 (-0.29, -0.04)	0.009	-0.51 (-0.63, -0.40)	-0.24 (-0.33, -0.15)	<0.001
⩾75	-0.46 (-0.56, -0.36)	-0.15 (-0.24, -0.06)	0.001	-0.36 (-0.50, -0.22)	-0.09 (-0.22, 0.03)	0.131	-0.54 (-0.66, -0.42)	-0.17 (-0.27, -0.07)	0.001
P for trend			0.130			0.366			0.014

[a] Adjusted for age, race, education level, poverty-income ratio, body mass index, smoking, drinking, physical activity, sleep duration,

hypertension, diabetes and season of measurement.

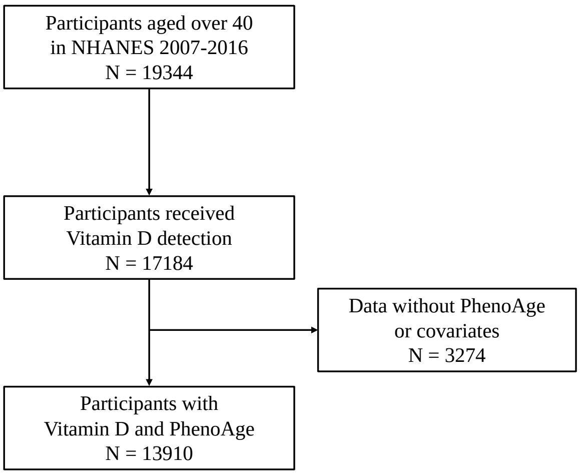

Figure 1 Flowchart of participants selection.

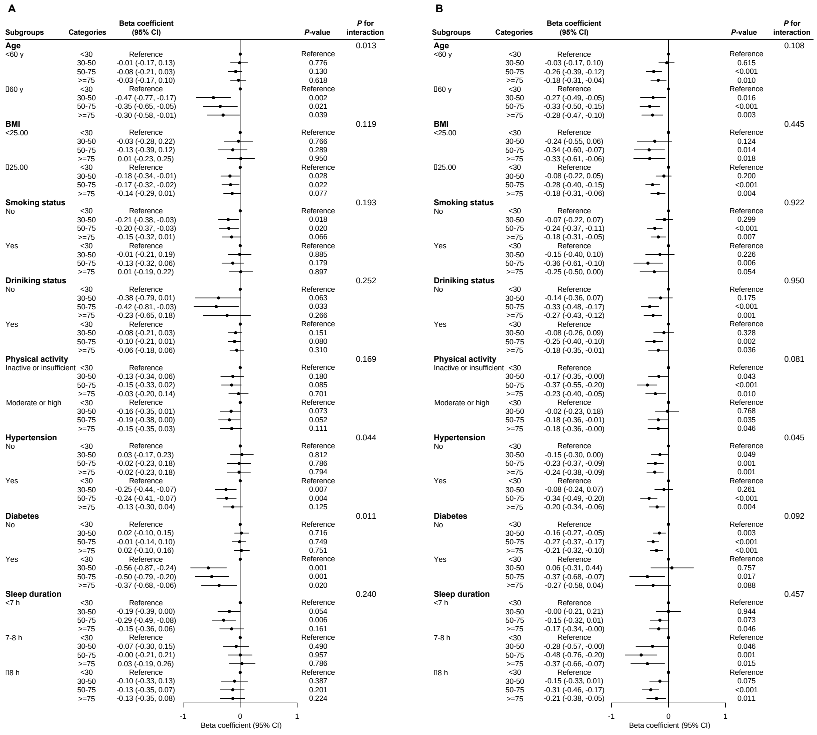

Figure 2 Subgroup analyses of the association between 25(OH)D categories (based on the 2011 Institute of Medicine) and PhenoAgeAccel among males (A) and females (B). The filled circles and horizontal lines represent regression coefficients and the 95% confidence intervals. Multiple linear regression models were adjusted for age, race, education level, poverty-income ratio, body mass index, smoking, drinking, physical activity, sleep duration, hypertension, diabetes and season. BMI, body mass index; CI, confidence intervals.

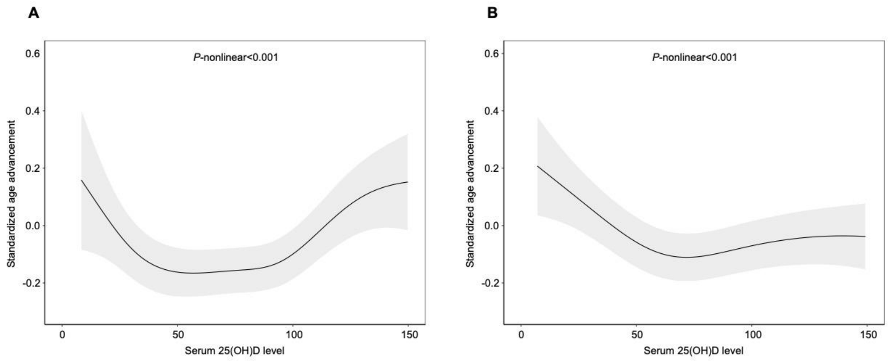

Figure 3 Nonlinear relationships between 25(OH)D serum level and PhenoAgeAccel among males (A) and females (B). Generalized additive models were adjusted for age, race, education level, poverty-income ratio, body mass index, smoking, drinking, physical activity, sleep duration, hypertension, diabetes and season. The black solid line represents the smooth curve fit between variables. The grey band represents the 95% confidence interval of the fit.

Table 3. Results of the analysis of the relationship between 25(OH)D serum levels and PhenoAge

25(OH)D serum levels (nmol/L)	Beta coefficient (95% CI)	*P*-value
Male		
<38.2~~0~~	-0.013 (-0.025, -0.002)	0.017
38.2~~0~~-91.~~55~~6	0.001 (-0.001, 0.002)	0.487
⩾91.~~55~~6	0.005 (0.002, 0.008)	<0.001
Female		
<62.5~~0~~	-0.007 (-0.010, -0.004)	<0.001
62.5~~0~~-125.0~~0~~	0.002 (0.000, 0.004)	0.029
⩾125.0~~0~~	-0.001 (-0.005, 0.002)	0.535

Generalized additive models were adjusted for age, race, education level, poverty-income ratio, body mass index, smoking, drinking, physical activity, sleep duration, hypertension, diabetes and season.

Dear editor Mickael Essouma

We sincerely thank you for the valuable and constructive suggestions, especially the meticulous word-for-word revisions. We have carefully studied all comments and incorporated them thoroughly into our revisions. We are very grateful for the editor's dedicated support and hope the revised manuscript is now satisfactory. Should you have any questions, please feel free to contact us.

1. Please review your reference list to ensure that it is complete and correct. If you have cited papers that have been retracted, please include the rationale for doing so in the manuscript text, or remove these references and replace them with relevant current references. Any changes to the reference list should be mentioned in the rebuttal letter that accompanies your revised manuscript. If you need to cite a retracted article, indicate the article's retracted status in the References list and also include a citation and full reference for the retraction notice.

Response: We have checked the references one by one, and no retracted article was listed in the manuscript. [22-24] and [50] were added in the references.

Additional Editor Comments:

2. The authors have continued to improve upon their manuscript, but need to polish the presentation of the materials and methods section and further edit the manuscript to improve the language quality.

Response: Thank you for your valuable feedback. We appreciate your suggestion to enhance the presentation of the materials and methods section. We will carefully review and revise this section to improve clarity, organization, and detail, ensuring that the methodology is clearly described and easily reproducible. Additionally, we will conduct a thorough language review throughout the manuscript to improve the overall quality and readability. We are committed to refining our manuscript accordingly and believe these revisions will strengthen the clarity and impact of our study.

Materials and Methods section.

3. Lines 159-164: consider addressing my request to provide references supporting the classification made for sleep duration, hypertension and diabetes.

Response: We thank the editor for your constructive suggestions. We have supplemented the following references to support the classification regarding sleep duration, hypertension and diabetes.

22. Chaput JP, McNeil J, Despres JP, Bouchard C, Tremblay A. Seven to eight hours of sleep a night is associated with a lower prevalence of the metabolic syndrome and reduced overall cardiometabolic risk in adults. PLoS One. 2013;8(9):e72832. Epub 2013/09/17. doi: 10.1371/journal.pone.0072832. PubMed PMID: 24039808; PubMed Central PMCID: PMCPMC3764138.

23. Muntner P, Hardy ST, Fine LJ, Jaeger BC, Wozniak G, Levitan EB, et al. Trends in Blood Pressure Control Among US Adults With Hypertension, 1999-2000 to 2017-2018. JAMA.

2020;324(12):1190-200. Epub 2020/09/10. doi: 10.1001/jama.2020.14545. PubMed PMID: 32902588; PubMed Central PMCID: PMCPMC7489367
24. American Diabetes A. Diagnosis and classification of diabetes mellitus. Diabetes Care. 2014;37 Suppl 1:S81-90. Epub 2013/12/21. doi: 10.2337/dc14-S081. PubMed PMID: 24357215.

4. As commented in the last round of review, you need to clearly specify that you conducted effect modification analyses for the association between 25(OH)D serum levels and PhenoAgeAccel in the statistical analysis sub-section before making the comment currently available in line 179. These references may be helpful in this respect: Edwarsd JR. Methods for Integrating Moderation and Mediation: A General Analytical Framework Using Moderated Path Analysis. Psychological Methods 2007, Vol. 12, No. 1, 1–22 and Knol et al. Recommendations for presenting analyses of effect modification and interaction. International Journal of Epidemiology 2012;41:514–520 doi:10.1093/ije/dyr218IF: 6.4 Q1 .This statement appears in the abstract: "Generalized additive models further found 25(OH)D serum 25 levels were linked to negative PhenoAgeAccel at levels below 38.2 nmol/L (15.3 ng/mL) in males (β=-0.013; 95% CI: -0.025 to -0.002) and 62.5 nmol/L (25.0 ng/mL) in females (β=-0.007; 95% CI: -0.01 to -0.004,), revealing a U-shaped relationship observed in males but an L-shaped pattern in females. . However, 25(OH)D serum levels above 125 nmol/L were not associated with PhenoAgeAccel in females (β=-0.001; 95% CI: -0.005-0.002), while in males, elevated levels (>91.55 nmol/L) were associated with positive PhenoAgeAccel (β=0.005; 95% CI: 0.002-0.008).". As requested in the last round of review, consider specifying in the methods section of the main manuscript the reasons why you chose those cut-off values for serum 25-vitamin D while performing the corresponding statistical analyses.

Response: We thank the editor for your constructive suggestions. We have supplemented moderated regression analyses incorporating a multiplicative interaction term (25(OH)D × gender) to evaluate whether the relationship between vitamin D status and biological aging differs by gender. The results of these analyses revealed statistically significant gender-specific effects (p for interaction <0.001), indicating that the association between 25(OH)D and PhenoAgeAccel is moderated by gender. In the revised manuscript, we have supplemented the following sentences in the **Methods** section: “To evaluate potential effect modification by gender and other key covariates, subgroup analyses and interaction tests were conducted. Interaction term of serum 25(OH)D and stratifying factor were included in the multivariable linear regression models to test for significance of interaction.”

In the **Results** section, we have supplemented the following sentences: “Interaction test confirmed that association between serum 25(OH)D and PhenoAgeAccel was modified by gender, regardless of whether 25(OH)D was modeled as a continuous variable or categorized using quartiles or IOM guidelines (all p for interaction <0.001).”

Regarding the cut-off values identified in the segmented regression analysis, these were determined based on data-driven statistical criteria. Specifically, we first used a generalized additive model (GAM) with a smooth term for serum 25(OH)D levels to flexibly characterize the non-linear relationship with PhenoAgeAccel, as GAMs effectively capture complex dose-response patterns without assuming parametric forms. The GAM revealed distinct non-linear associations by sex, prompting further investigation using segmented regression—a method designed to formally identify inflection points (thresholds) where the slope of the association changes directionally. For

each gender, optimal breakpoints were selected via likelihood ratio tests comparing nested models, ensuring statistical rigor in threshold estimation. These thresholds were computationally derived rather than manually assigned. However, we recognized potential ambiguity in how these thresholds were presented. To clarify, we have revised the following sentences in the **Abstract**: "25(OH)D serum levels were negatively associated with PhenoAgeAccel (β=-0.04 standard deviation [SD]; 95% confidence interval [CI]: -0.08 to 0.00). This negative association was dose-dependent in females (β=-0.07 SD; 95% CI: -0.12 to 0.01), but not in males. Generalized additive models further revealed gender-specific non-linear patterns: a U-shape pattern in males but an L-shaped pattern in females. Using segmented regression to confirm inflection points, we observed that 25(OH)D serum levels were linked to reduced PhenoAgeAccel at levels below 38.2 nmol/L (15.3 ng/mL) in males (β=-0.013 SD; 95% CI: -0.025 to -0.002) and 62.5 nmol/L (25.0 ng/mL) in females (β=-0.007 SD; 95% CI: -0.01 to -0.004,). However, 25(OH)D serum levels above 125 nmol/L showed no association with PhenoAgeAccel in females (β=-0.001 SD; 95% CI: -0.005-0.002), while in males, elevated levels (>91.55 nmol/L) were associated with increased PhenoAgeAccel (β=0.005 SD; 95% CI: 0.002-0.008)." Additionally, we have polished the language in the **Results** section to improve clarity.

.

5. Results section. As commented in the last round of review, consider rounding all the numbers to one decimal place when providing results throughout the manuscript (Table 1 and so on).

Response: We have corrected all the numbers to one decimal place.

Comment appended to Table 1.

6. Discussion section: limitations statement. You also need to address the lack of data on vitamin D supplement among the confounders, the effect of unmeasured confounding and the reverse causation and residual confounding.

Response: This information has been added in section of limitation.

7. Comment about editing: Consider complying with PLOS ONE authors guidelines by not numbering the manuscript's sections and sub-sections.

Edits proposed in lines 123-125, 141-142, 178, 188, 251, 262,Table 1.

Response: We have made corrections following the editor's suggestion for our manuscript. Thank you for your valuable feedback.
