# Supplementary material for: Association of 25(OH)D serum level with biological aging: A Cross-Sectional Study of 2007–2016 NHANES surveys
Source: PLoS One. 2025 Aug 6;20(8):e0328107. doi: 10.1371/journal.pone.0328107 (PMC12327616; doi:10.1371/journal.pone.0328107)
Supplement: Attachment — Submitted filename: Response to Reviewers.docx [file pone.0328107.s004.docx]

**Dear editor and reviewer:**

Thank you very much for your careful review and constructive suggestions with regard to our manuscript. Those comments are helpful for us to revise and improve our paper. We have studied comments carefully and tried our best to revise and improve the manuscript and made great changes in the manuscript according to the good comments. We earnestly appreciate for the warm work from you, and hope that the corrections will meet with approval. Please feel free to contact us with any questions and we are looking forward to your consideration. The main corrections in the paper and the responds to your comments are as following:

**Additional Editor Comments:**

**In addition to the reviewer's comments, I have the following comments:**

1. **In the title, you would better replace "age advancement" with "****biological aging" or "physiological aging" given that PhenoAge used as metric of aging in this study is a measure of biological/physiological aging.**

**Response:** Thank you for your insightful feedback. We have revised the title to use "biological aging" instead of "age advancement" to more accurately reflect our study's focus on the biological aspects measured by the PhenoAge metric.

2. **The authors should revise the "Materials and methods", results and discussion sections based on the STROBE guidelines statement (see von Elm et al. PLoS Med. 2007;4(10):e296) to allow for the reproducibility of their methods. Accordingly, they should start the "Materials and methods section" stating that their report conforms to STROBE guidelines for observational studies, and upload a STROBE checklist in the appendix. The "Statistical analysis" sub-section should follow the** **SAMPL guideline statement (see** **Lang TA et al. Medical Writing.2016;25(3):31-36) and the authors should upload a statistical analysis plan for this study as well as raw data (if legal frameworks of NHANES allow that) as supplemental materials to this manuscript. This recommendation aims to improve the readability of their manuscript, allow the credibility and reproducibility of their study methods, and increase the credibility of their findings and trustworthiness of the interpretation of their findings.**

**Response:** Thank you for your constructive suggestions and guidance. We have thoroughly revised the manuscript to ensure alignment with STROBE guidelines for observational studies, as well as the SAMPL recommendations for statistical reporting. Meanwhile, in the Materials and methods section, we supplemented the following statement: “This study was conducted and reported in adherence to STROBE guidelines for observational studies.” (marked in blue, Page 4, Lines 95). The completed STROBE checklist is provided in Supplementary materials. A statistical analysis plan has also been written and provided in Supplementary materials. Regarding data accessibility, all raw data was publicly available through the NHANES website: <https://wwwn.cdc.gov/nchs/nhanes/Default.aspx>. According to the NHANES data use agreement, redistribution of raw datasets is prohibited. Therefore, we are unable to provide NHANES raw data directly. This has been clarified in the Materials and methods section: “All data included in current study are publicly available through the NHANES website [18].” (marked in blue, Page 5, Lines 99).

The following reference was supplemented in the References List:

19.	Centers for Disease Control and Prevention National Health and Nutrition Examination Survey Questionnaires D, and Related Documentation 2020. [cited 2025 March 7]. Available from: <https://wwwn.cdc.gov/nchs/nhanes/>.

3. **The "Materials and methods" section should end with a sub-section titled "****Ethical considerations"in which the authors should state whether they abode by the ethical rules for conducting medical/health research (see the** **2024 declaration of** **Helsinki statement published in JAMA), how they accessed NHANES (USA) data whereas all authors are affiliated with China institutes and the study was funded by a Chinese organization). Did they get an IRB approval to use the NHANES dataset?**

**Response:** We thank the editor for your constructive suggestions and guidance. Our research adhered to the ethical principles in the 2024 Declaration of Helsinki regarding secondary analysis. It exempts studies from additional ethical review when utilizing pre-existing de-identified data [1]. Meanwhile the NHANES datasets we used were publicly available and did not contain restricted use datasets. According to the CDC/NCHS Research Ethics Review Board statement, researchers using only the publicly available data files do not need to seek IRB approval [2]. Hence, no additional IRB was required in our study.

In the Materials and methods section, we have supplemented the “Ethical considerations” sub-section in the revised manuscript: “This study utilized data from NHANES study, which was conducted in accordance with the principles in the Declaration of Helsinki. The NHANES protocol received prior ethical approval from the National Center for Health Statistics' Review Board. Since our study is a secondary analysis of publicly accessible, de-identified NHANES data sets, additional institutional review board approval was not required [26, 27].” (marked in blue, Page 7, Lines186).

The following reference was supplemented in the References List:

26.	University of Connecticut. Guidance on secondary analysis of existing data sets. [cited 2025 Feb 5]. Available from: https://ovpr.uconn.edu/services/rics/irb/researcher-guide/secondary-analysis-of-data-sets/.

27.	World Medical A. World Medical Association Declaration of Helsinki: Ethical Principles for Medical Research Involving Human Participants. JAMA. 2025;333(1):71-4. Epub 2024/10/19 20:42. doi: 10.1001/jama.2024.21972. PubMed PMID: 39425955.

4. **In the conflicts of interest statement on the manuscript's agenda, the authors should clarify any potential** **conflict of interest with the funder and/or NHANES database managers.**

**Response:** We thank the editor for your guidance. We have revised the “**Conflict of interest”** statement in the manuscript as follows: “The authors declare no competing financial or non-financial interests related to this work. NHANES is a publicly available dataset managed by the Centers for Disease Control and Prevention. The authors confirm that there is no affiliation with or involvement from the NHANES program administrators in this study. The funding source had no role in study design, data collection, analysis, or manuscript preparation.”**.**

5. **It is difficult to know in tables and figures what type of effect magnitude estimates (odds ratios? Others?) the authors base their conclusions on and whether those estimates are those that should be used in cross-sectional studies to assess associations: see Thiese MS.** **Observatiobal and interventional study design types; an overview. Biochemistry Med (Zagreb).2014;24(2):199-210. Did you perform logistic regression analyses and/ or correlation analyses? Why perform both or why perform one of them? These clarifications need to be made in the statistical analysis sub-section of the "Materials and methods" section. Along these lines, I would like to remember that the authors overstated their conclusions (see Schooling MC and Heidi JE. Clarifying questions about "risk factors": predictors versus explanation. Epidemiology in Themes. 2018;15:10), and this should be corrected throughout the manuscript.**

**Response:** We sincerely thank the editor for your guidance. Regarding the choice of effect estimates, our analyses utilized beta coefficients derived from weighted multivariable linear regression models. According to Thiese et al., odds ratios are standard for binary outcomes in logistic regression. However, when measuring continuous response variables, beta coefficients are recommended as they quantify linear association between continuous variables. To improve the clarity of our manuscript, we supplemented the following sentences in the Materials and methods section: “Beta coefficients with 95% confidence intervals (CIs) were reported, representing the change in dependent variable per unit increase in the independent variable. The use of beta coefficients provides interpretable estimates for linear associations when analyzing continuous dependent variables, which is widely recommended in epidemiological studies.” (marked in blue, Page 7). Meanwhile, we made adjustment to all Tables and Figures, as well as description of results to ensure the use of beta coefficient is straightforward.

We also supplemented the following sentences in the Materials and methods section to explain why correlation analysis is omitted in current study: “Correlation analysis was not employed because it does not account for confounding factors, whereas the primary aim of current study is to estimate the confounder-adjusted association between 25(OH)D status and PhenoAge aging.” (marked in blue, Page 7).

The following reference was supplemented in the References List:

24.	Bender R. Introduction to the use of regression models in epidemiology. Cancer epidemiology: Springer; 2009. P. 179-95

Regarding the potential overstatement of conclusion, we have revised the manuscript following the guidance of Schooling and Heidi (2018). Adjustments have been made throughout the Abstract, Results, and Discussion and Conclusion sections to avoid formative and causal language.

6. **The authors cite res 4 to 6 for the definition of vitamin D deficiency but use ref 18 for the definition of vitamin D deficiency in this study. Why can't they use only reference 6 in the introduction and for the definition in this study given that it is the most recently published article? Can you specify whether you assessed the human form of 25-OH-D (25-OH-D3) throughout the manuscript?**

**Response:** We thank the editor for your careful reading and constructive suggestions. We realize that the previous version of the manuscript cited multiple references regarding the deficiency of vitamin D level, which induced redundancy. We agree that ref 6 is the latest and authoritative source for defining vitamin D status. Therefore, we have unified citation of ref 6 in both introduction and methods sections, meanwhile remove ref 4-5 and 18 from our revised manuscript.

Regarding the detected form of 25-OH-D, we supplemented the following sentences in the Methods to improve the clarity of our manuscript: “From cycle 2007-2008 and beyond, liquid chromatography-tandem mass spectrometry (LC-MS/MS) was used to measure serum 25(OH)D concentrations, including 25(OH)D3, epi-25(OH)D3, and 25(OH)D2 in human serum. We calculated the 25(OH)D levels by adding 25(OH)D3 and 25(OH)D2 levels.” (marked in blue, Page 5).

7. **After reading your introduction, I still do not well understand the rationale for conducting this study given that as well mentioned in reference 6 of the current manuscript, it is largely known that older individuals generally have lower vitamin D levels than younger populations. Do you want to asses whether that knowledge based on social age could hold even when using biological aging metrics (taking into consideration the discrepancy between social and biological ages)? The aim and rationale for conducting the study should be unambiguous in the last paragraph of the introduction. Still regarding the introduction, it is important to begin to be more cautious and specific when making claims about** **the involvement of vitamin D in human disease based on epidemiological data (see Vitamin D and human healrh: evidence from Mendelian randomization studies. Eur J Epidemiol. 2024;39: 467-490 and Schooling MC and Jones HE. Clarifying questions about "risk factors": predictors versus explanation. Epidemiology in Themes. 2018; 15:10). From my perspective, in a study whereby you seemingly assess the epidemiological link between vitamin D and physiological aging, the introduction should (in a brief overview form: introduction of no more than 1.5 pages) highlight how the human form of vitamin D evolves throughout the age spectrum based on the most certain available peer-reviewed evidence, as well as what is known and what is unknown about the epidemiological association between the human form of vitamin D and physiological aging, before stating the rationale for conducting the study (gaps in knowledge you would like to fill) and the study's aim.**

**Response:** Thank you for your feedback regarding the introduction section of our manuscript. We have rewritten the introduction section to make it clear and concise in the revised manuscript. Briefly, the aim of current study is to investigate the relationship between vitamin D and physiological aging. We have read the articles mentioned above and rewrite the introduction of vitamin D in human disease based on epidemiological data. The length of introduction has been limited to less than 2 pages, a little longer than 1.5 pages. We have done our best to improve the induction section and hope this improvement could meet your requirement. Thank you very much.

**8. Finally, language editing is also required.**

**Response:** Thank you for your valuable feedback and suggestions for improvement. I acknowledge the need for language editing to enhance the clarity, precision, and professionalism of the manuscript. I will thoroughly review and ask help from professional language improvement to ensure that the text is clear and effectively conveys the intended meaning.

**Reviewer #1:**

**Feng et al conduct this research in a rare direction while most of researchers will focus on the association between 25(OH)D and disease. Here are some issues should be concerned.**

1. **In the abstract, there is no statistical results. The conclusion that VD deficiency promotes aging is not proper since cross sectional study only remind the possible association between exposure and outcome instead of causuality.**

**Response:** Thank you for your thoughtful feedback. We have revised the abstract to include key statistical results to better inform readers about the study's findings.

Additionally, we acknowledge your point regarding the limitations of cross-sectional studies in establishing causality. You are correct that such studies can only identify associations, and we will revise the language in the abstract and manuscript to reflect this appropriately. Specifically, we will modify the conclusion to state that vitamin D deficiency "may be associated with" or "shows a potential link to" biological aging, rather than implying causation.

2. **The introduction section is a little long, in which 3-4 paragraphes can be better.**

**Response:** Thank you for your suggestion. We acknowledge that the current introduction may be too lengthy and appreciate your suggestion to condense it into 3-4 focused paragraphs.

3. **In methods section, data including flow chart should be added, and please learn some from the below articles:**

**a.** **Possible non-linear relation between prostate specific antigen and vitamin D: a machine learning study based on cross-section data**

**b.** **Association between urinary metals and prostate-specific antigen in aging population with depression: a cross-sectional study**

**Response:** We thank the reviewer for your constructive suggestion. We have supplemented a flowchart that outline the participant selection process as Figure 1 in revised manuscript.

4. **In the discussion section, this study should declare the mechanism in the increase of 25(OH)D acclerate aging.**

**Response:** Thank you for your suggestion. We have rewritten the discussion section, providing the mechanism how the increase of 25(OH)D accelerate aging. “These detrimental effects may stem from the toxicity of excessive vitamin D. When vitamin D intake is too high, serum levels of its metabolites (25(OH)D and 24,25(OH)₂D) rise and surpass the binding capacity of vitamin D binding protein. This leads to an increase in the free active metabolite, 1,25(OH)₂D. This active form reaches the vitamin D receptor (VDR) in the nucleus, promoting gene expression that enhances calcium absorption and bone mobilization. Consequently, this can result in hypercalcemia or hyperphosphatemia, which in turn suppresses initial parathyroid hormone (iPTH) levels. These disrupting balance of calcium and hormone may lead to conditions such as cardiovascular disease, kidney damage, and skin aging. Research reported that the working diagnosis of vitamin D toxicity was determined based on total concentrations of 25(OH)D at 32~80ng/mL (80~200 nmol/L) detected by immunoassay. Our results found the 25(OH)D levels above125 nmol/L in female participants were linked to accelerated physiological aging, approximately consistent with those reports.” (marked in blue, Page 12)

5. **Except the increase of 25(OH)D acclerate aging, if there are some literatures reporting aging can increase 25(OH)D. Maybe it is hard to explain the causality of the interactive association between 25(OH)D and aging. Please make this part clear.**

**Response:** The relationship between 25(OH)D levels and aging is complex and bidirectional, making it challenging to establish a clear cause-and-effect relationship in our cross-sectional study. It is widely accepted that the level of vitamin D decrease as people get old, and people grow awareness of vitamin D deficiency and related health problems, vitamin D became a popular supplement, and its use has increased markedly. However, an increased intake of vitamin D supplements by the general population and a growing number of prescriptions of therapeutic doses without medical monitoring might result in a greater risk of excessive vitamin D toxicity. We have added some words to make this part clear in the discussion section. Thank you so much for your suggestion.

6. **Language should be revised. Like in conclusion, "These relationships exhibited nonlinearity in both males and females.". It is not clear between what.**

**Response:** Thank you for your valuable feedback and suggestions for improvement. I acknowledge the need for language editing to enhance the clarity, precision, and professionalism of the manuscript. I will thoroughly review and ask help from professional language improvement to ensure that the text is clear.

**Reference**

1.	World Medical A. World Medical Association Declaration of Helsinki: Ethical Principles for Medical Research Involving Human Participants. JAMA. 2025;333(1):71-4. Epub 2024/10/19 20:42. doi: 10.1001/jama.2024.21972. PubMed PMID: 39425955.

2.	NHANES Data Access Policy [cited 2025 feb 25]. Available from: <https://www.cdc.gov/nchs/policy/data-user-agreement.html>.
