# Supplementary material for: Association of 25(OH)D serum level with biological aging: A Cross-Sectional Study of 2007–2016 NHANES surveys
Source: PLoS One. 2025 Aug 6;20(8):e0328107. doi: 10.1371/journal.pone.0328107 (PMC12327616; doi:10.1371/journal.pone.0328107)
Supplement: Attachment — Submitted filename: response_rev2_20250514.docx [file pone.0328107.s007.docx]

**Dear editor and reviewer:**

Thank you very much for your careful review and constructive suggestions with regard to our manuscript. Those comments are helpful for us to revise and improve our paper. We have studied comments carefully and tried our best to revise and improve the manuscript and made great changes in the manuscript according to the good comments. We earnestly appreciate for the warm work from you, and hope that the corrections will meet with approval. Please feel free to contact us with any questions and we are looking forward to your consideration. The main corrections in the paper and the responds to your comments are as following:

**Additional Editor Comments:**

**The authors have improved the manuscript. However, as mentioned by the reviewers, there is a room for further improvement.**

**Suggestions about how to address the comments of Reviewer 5:**

**1. The terminologies "Physiological age" and "Biological age". Although the terminology "Physiological age" is known in the health literature to be based on some bodily functions as described in the article by Nakamura et al. Further evaluation of physical fitness age versus physiological age in women. Eur J Appl Physiol (1998) 78: 195-200 for example, the authors should indeed replace the terminology "Physiological age" with "biological age" where necessary because DNAm PhenoAge, the aging metric used in this study, is a composite of biomarkers, not bodily functions.**

**Response:** we have replaced the "Physiological age" with "biological age". Thank you for this suggestion.

**2. The race issue. The authors should stick to the racial stratification used in the 2007, 2008, 2009, 2010, 2011, 2012, 2013, 2014, 2015 and 2016 NHANES surveys. Indeed, this is not the first report on 25(OH)D assessment in a multiracial NHANES population as shown by the article Daraghmeh et al. Evidence for the vitamin D hypothesis: The NHANES III extended mortality follow-up. Atherosclerosis 255 (2016) 96e101. The lack of data segregation by race could be discussed as a common study limitation of NHANES surveys (https://pubmed.ncbi.nlm.nih.gov/?term=Vitamin+D+AND+NHANES) in the absence of race-specific cut-off values for vitamin D levels as for other biological biomarkers such as the creatinine-based estimated glomerular filtration rate, and when it is unclear whether 25-hydroxyvitamin serum levels ≥ 20 ng/mL are optimal across all racial groups in the USA: see The Journal of Clinical Endocrinology & Metabolism, Volume 109, Issue 8, August 2024, Pages 1948–1954, https://doi.org/10.1210/clinem/dgae322IF: 5.0 Q1 .**

**Response:** the discussion of race has been added in the revised manuscript. thanks for your suggestion**.** **(Page14, line 371-381)**

**3. The cut-off value of 25-hydroxyvitamin D for vitamin D deficiency. Given that the reviewer did not provide a reference to support the value of 25 nmol/L proposed, I recommend the authors stick to 20 ng/mL (50 nmol/L) as the cut-off value to define optimum and low levels of 25-hydroxyvitamin D in this study: see J Clin Endocrinol Metab. 2024 Jul 12;109(8):1907-1947. doi: 10.1210/clinem/dgae290IF: 5.0 Q1 . and The Journal of Clinical Endocrinology & Metabolism, Volume 109, Issue 8, August 2024, Pages 1948–1954, https://doi.org/10.1210/clinem/dgae322IF: 5.0 Q1 . However, the authors need to implement the reviewer's comment in their discussion of study limitations. In the same vein, the lack of genetic data on isoform of Vitamin D binding protein (VBD) should be discussed as a study limitation because VBD is allegedly the main determinant of the unbound 25-hydroxyvitamin D which is postulated to be the active compound of 25(OH)D (Bikle and Schwatrz. Vitamin D Binding Protein, Total and Free Vitamin D Levels in Different Physiological and Pathophysiological Conditions. Front Endocrinol (Lausanne). 2019 May 28:10:317. doi: 10.3389/fendo.2019.00317.).**

**Response:** we have added a paragraph discussing the cut-off value of 25-hydroxyvitamin D for vitamin D deficiency. (Page 13, line 332-352)

“Regarding the threshold of vitamin D deficiency, it remains an ongoing controversy. The Scientific Advisory Committee on Nutrition (2016) recommended threshold at 25 nmol/L among UK population [46], whereas the IOM (2011) suggested thresholds at 30 nmol/L based on evidence in U.S. and Canadian populations [20]. Meanwhile, the European Food Safety Authority 2016 and the Endocrine Society guideline 2011 proposed higher level of 50 nmol/L as deficiency cutoff [47, 48]. The discrepancy in cutoff values stems from multiple factors, for example, different regional ultraviolet B exposure patterns, dietary practice and population-specific biological variability. Furthermore, guideline variations also reflect different focus on health outcomes, with some prioritizing musculoskeletal health [49]. In 2024, the Endocrine Society guideline retracted their previous recommendation of threshold at 50 nmol/L due to limited clinical trial evidence [7]. It also highlighted the impracticality of universal thresholds due to heterogeneity in health outcomes and population characteristics. Hence, the interpretation of 25(OH)D level threshold determination in our study should be with caution, especially when it derived from observational study in an ethnically diverse population. Nevertheless, the trend of benefits attenuation we observed is worth noting, suggesting excessive level of serum 25(OH)D may not be beneficial regarding biological aging process.”

**4. Contemporary evidence about vitamin D association with disease. As mentioned in the first round of review and further highlighted by Reviewer 5, the authors need to exercise caution when stating that vitamin D serum concentrations are associated with diseases and that vitamin D supplementation is beneficial in disease prevention. For example, see the articles by Fang A, Zhao Y, Yang P, Zhang X, and Giovannucci EL. Vitamin D and human health: evidence from Mendelian randomization studies. Eur J Epidemiol. 2024;39(5):467-90. Epub 2024/01/12. doi: 10.1007/s10654-023-01075-4IF: 7.7 Q1 . PubMed PMID: 38214845IF: 7.7 Q1 , Sun et al. Association between vitamin D level and risk of type 2 diabetes: a systematic review of Mendelian Randomization studies. Crit Rev Food Sci Nutr. 2025 Mar 12:1-10. doi: 10.1080/10408398.2025.2466758IF: 7.3 Q1 , J Clin Endocrinol Metab. 2024 Jul 12;109(8):1907-1947. doi: 10.1210/clinem/dgae290IF: 5.0 Q1 . and The Journal of Clinical Endocrinology & Metabolism, Volume 109, Issue 8, August 2024, Pages 1948–1954, https://doi.org/10.1210/clinem/dgae322IF: 5.0 Q1 .**

**Response:** we have checked the expression of our result and conclusion to avoid expressing causal relationships. Our study is only a cross-sectional epidemiological research.

**5. The main source of vitamin D in human beings. I agree with Reviewer 5 that food intake may be the greatest source of vitamin D in White people living in the Transatlantic region where the sunny period is relatively limited. However, textbooks report vitamin D production by the skin as the main source of vitamin D in human beings, with food intake contributing to only up to 10% of the human body's store of vitamin D. The limited epidemiologic data on the source of vitamin D in the other ancestral groups in the transatlantic region and in tropical regions (for example, see the articles by Vearing, R.M., Hart, K.H., Darling, A.L. et al. Global Perspective of the Vitamin D Status of African-Caribbean Populations: A Systematic Review and Meta-analysis. Eur J Clin Nutr 76, 516–526 (2022). https://doi.org/10.1038/s41430-021-00980-9IF: 3.6 Q2 and Lancet Glob Health. 2020 Jan;8(1):e134-e142. doi: 10.1016/S2214-109X(19)30457-7IF: 19.9 Q1 . Epub 2019 Nov 27) does not allow the verification of this textbook knowledge in these other ancestral groups. However, vitamin D production by the skin could be the predominant source of vitamin D in the non-White population (at least those with a melanoderm skin), given their much higher exposure to sun-derived UVB radiations throughout the year with co-existent undernutrition when compared with White populations.I have also appended additional comments in the attached pdf.**

**Response:** sun exposure and food intake are main sources of vitamin D. We totally agree this comment from reviewer and editor. The discussion has been added in the section of limitation. We have carefully read the additional comments added from editor. thank you so much for helping us improving our manuscript.

**Reviewers' comments:**

**Reviewer #1: All revisions were made and there is no further comment. The revised manuscript meet the journal publish standard.**

**Response:** Thank you so much.

**Reviewer #2: There is no comment. In my opinion, the present version of the manuscript is ready for publication. However, please check the plagiarism of the whole text and be sure that the similarity index was less than 30 %.**

**Response:** Thank you so much. We have checked the plagiarism of manuscript and it meets the journal standards.

**Reviewer #3: Authors addressed most of the previous comments from reviewers. The manuscript reads better in terms of flow and clarity. However further language editing is still required in many sections throughout the manuscript.**

**1. In the abstract, authors to reflect the U shape effect observed among the males; improve clarity of sentences**

**Response:** we are sorry for this language problem. In the revised abstract, we clearly state the U-shaped effect, provides context for its significance, and ensures each sentence is concise and informative.

**2. In methodology, citing previous publications, authors to briefly elaborate on each biomarkers/model. At present, there's gap of information for these modelling.**

**Response:** We thank the reviewer for your constructive comments. To fully elucidate the rationale for selecting analysis models, we have supplemented the following sentences in the **Methods** section: “To explore the non-linear relationship between serum 25(OH)D and physiological aging, generalized additive model (GAM) was constructed. The GAMs extend traditional linear regression by replacing linear terms with smooth functions, allowing flexible modeling of complex dose-response relationships without assuming a priori parametric forms. The threshold effect was further estimated using segmented regression model. This method identifies inflection points where the slope of the association between 25(OH)D and PhenoAgeAccel changes significantly. The optimal breakpoint was determined via likelihood ratio tests, comparing the goodness-of-fit between the non-segmented linear model and the segmented regression model.” **(Page 7, line 180-189).**

The following references were supplemented in the References List:

28 Hastie TJ. Generalized additive models. Statistical models in S. 2017:249-307.

29 Muggeo VM. Estimating regression models with unknown break-points. Stat Med. 2003;22(19):3055-71. Epub 2003/09/16. doi: 10.1002/sim.1545. PubMed PMID: 12973787.

**3. Is there methodology established to determine physiological aging? if there is, how does the PhenoAge algorithm fare when compared? This would further establish validity of methodology.**

**Response:** We thank the reviewer for your constructive comments. In agree with the editor’s suggestion, the terminology has been revised from "physiological aging" to "biological aging" throughout the manuscript, as this better aligns with the definition of PhenoAge—a composite metric integrating 10 clinical biomarkers that collectively reflect systemic inflammatory, metabolic, and hematopoietic dysfunction. There are several established methods for quantification of biological aging, including molecular clocks (e.g., DNA methylation clocks), functional decline indices (e.g., frailty index) and composite biomarker metrics (e.g., PhenoAge). The PhenoAge algorithm was constructed based on NHANES III (1988–1994), and was validated using data form NHANES IV (1999–2010). Hence, in NHANES dataset, the PhenoAge method showed good data compatibility and epidemiological validity. In the **Methods** section, we have supplemented the following sentences to elucidate the rationale for choosing PhenoAge as biological aging indicator: “In this study, we utilized PhenoAge as the indicator of biological aging, considering its high robustness and feasibility in NHANES data as a reliable predictor of mortality and age-related disease risks. The PhenoAge algorithm is constructed using NHANES III (1988–1994) by elastic-net regression, encompasses 10 markers: albumin, creatinine, glucose, white blood cell count, lymphocyte percent, red cell distribution width, mean red cell volume, C-reactive protein, alkaline phosphatase and chronological age [21]. PhenoAge was both developed and validated within NHANES populations. Meanwhile, all constituent biomarkers are routinely measured in NHANES with standardized protocols, ensuring reproducibility across study cycles.” **(Page 5, line 118-140).**

**4. In discussion and results, there's inconsistency in reporting ie ie dose-dependent relationship in females**

**Response:** we have improved this description in the section of discussion and results. Thank you for your suggestion.

**5. In discussion, further strengthening of write up required. At present, it is unclear if low vit d level drives/associated with physiological ageing or is an impact of ageing process.**

**Response: N**umerous evidences have found that vitamin D may play a role in modulating key biological pathways associated with chronological age. Vitamin D is known to influence cellular senescence, oxidative stress, and inflammation, which are central to the aging process. Furthermore, experimental studies have demonstrated that vitamin D supplementation can mitigate oxidative damage and improve immune function in elder, suggesting its potential role in slowing age-related physiological decline. Thus, we did not provide enough information about this aspect. In the revised manuscript, we have added this in the discussion. Moreover, we have asked professional help to improve our language. Thank you so much for your review.

**Reviewer #4: Feng et al. investigated the association between vitamin D deficiency and physiological aging. While the authors have made considerable efforts to address previous reviewers’ comments, I believe the manuscript still requires further revision. Below are my comments:**

**1. The Introduction remains somewhat lengthy. Additionally, please clarify that endogenous vitamin D production in humans and animals yields vitamin D3, not D2 (see line 43).**

**Response:** thank you for your suggestion. The revised introduction is more streamlined, removing unnecessary details while maintaining essential information.

Vitamin D exists in two primary forms: vitamin D3 (cholecalciferol) and vitamin D2 (ergocalciferol). D3 is produced from 7-dehydrocholesterol (7-DHC) through a two-step process in which the B ring is broken by UV light (spectrum 280–320 UVB) radiation from the sun, forming pre-D3 that isomerizes to D3 in a thermo-sensitive but noncatalytic process. Both UVB intensity and skin pigmentation level contribute to the rate of D3 formation. Vitamin D3 is synthesized endogenously in the skin of humans and animals upon sunlight exposure, while vitamin D2 is primarily derived from dietary sources.

**2. Appropriate references should be added for the activation steps of vitamin D, including both the 25-hydroxylation and 1α-hydroxylation processes.**

**Response:** we have checked the reference and added appropriate one in the section of introduction.

**3. Line 47: Please correct the terminology to "****1****α,25-dihydroxyvitamin D."**

**Response:** Sorry for this mistake and we have corrected it. Thank you so much.

**4. Line 49: Choose either reference 1 or 2, citing both is unnecessary.**

**Response:** In the revised manuscript, reference 2 was removed.

**5. The criteria for defining vitamin D status are based solely on the Endocrine Society guidelines. The Institute of Medicine (IOM) guidelines, which differ substantially and are widely used in clinical settings, should also be mentioned. See ref below:**

**A. Catharine Ross, JoAnn E. Manson, Steven A. Abrams, John F. Aloia, Patsy M. Brannon, Steven K. Clinton, Ramon A. Durazo-Arvizu, J. Christopher Gallagher, Richard L. Gallo, Glenville Jones, Christopher S. Kovacs, Susan T. Mayne, Clifford J. Rosen, Sue A. Shapses, The 2011 Report on Dietary Reference Intakes for Calcium and Vitamin D from the Institute of Medicine: What Clinicians Need to Know, The Journal of Clinical Endocrinology & Metabolism, Volume 96, Issue 1, 1 January 2011, Pages 53–58, https://doi.org/10.1210/jc.2010-2704IF: 5.0 Q1**

**Response:** We thank the reviewer for your insightful comment. The Institute of Medicine (IOM) guidelines define serum 25(OH)D thresholds based on musculoskeletal health outcomes, including calcium absorption efficiency, fracture risk, and prevention of rickets/osteomalacia [1]. Their criteria, widely adopted in clinical practice for U.S. and Canadian populations, align with the NHANES data characteristics. The IOM classification are as follows: risk of deficiency (<30 nmol/L), risk of inadequacy (30–50 nmol/L), likelihood of sufficiency (50–75 nmol/L) and no increased benefit (≥75 nmol/L). These thresholds are evidence-based and regionally validated, making them appropriate for our exploratory analysis.

While the 2011 Endocrine Society guidelines proposed higher thresholds (sufficiency: ≥75 nmol/L and insufficiency: 50-75 nmol/L), their 2024 update withdrew these definitions due to insufficient clinical trial evidence linking specific 25(OH)D levels to net health benefits [2]. Hence, for operational clarity in cross-sectional analysis, we prioritized the IOM criteria. In the **Methods**, we revised the classification as follows: “According to the Institute of Medicine Committee recommendation, we transformed serum 25(OH)D concentration into categorical variables: risk of deficiency (<30 nmol/L), risk of inadequacy (30–50 nmol/L), likelihood of sufficiency (50–75 nmol/L) and no increased benefit (≥75 nmol/L) [20,21].” **(Page 5-6, line 112-115).**

The following reference was supplemented in the References List:

In: Ross AC, Taylor CL, Yaktine AL, Del Valle HB, editors. Dietary Reference Intakes for Calcium and Vitamin D. The National Academies Collection: Reports funded by National Institutes of Health. Washington (DC)2011.

Ross AC, Manson JE, Abrams SA, Aloia JF, Brannon PM, Clinton SK, et al. The 2011 report on dietary reference intakes for calcium and vitamin D from the Institute of Medicine: what clinicians need to know. J Clin Endocrinol Metab. 2011;96(1):53-8. Epub 2010/12/02. doi: 10.1210/jc.2010-2704. PubMed PMID: 21118827; PubMed Central PMCID: PMCPMC3046611.

**6. The rationale for the study remains unclear. The authors should clarify their aims more.**

**Response:** we have rewritten the introduction and improved clarification of our aims and background introduction (**Page 4, line 82-85**). We hope the improvement could make them clear and rational. Thank you very much.

**7. The sentence: “However, the comprehensive picture of the association between vitamin D levels and physiological aging remains to be fully elucidated, particularly in the general population without vitamin D deficiency or insufficiency” is vague. Please revise to clarify what is meant by “general population without deficiency or insufficiency.”**

**Response:** we want to investigate the relationship between vitamin D and biological aging in the general population, whether they have sufficient or insufficient vitamin D, as most of previous studies focus on the population with insufficient vitamin D, which may contribute to harmful effects on health. The former sentence has been rewritten as “However, the comprehensive picture of the association between vitamin D levels and biological aging remains to be fully elucidated, particularly in the general population with vitamin D levels within the normal range.”

**8. Although several covariates were adjusted for, the possibility of residual confounding, particularly from factors such as** **sun exposure and vitamin D supplementation, should be acknowledged in the discussion.**

**Response:** we totally agree with your opinion. sun exposure and vitamin D supplementation greatly affect vitamin D level in body. We have added the information in the section of discussion and limitation. (**Page 14, line371-381**)

**9. Recent research has provided new insights into vitamin D metabolism and its regulation by metabolic state:**

**Aatsinki and Elkhwanky et al. (2019) demonstrated that fasting and diabetes activate at least two transcriptional regulators i.e., PGC-1α/ERRα and the glucocorticoid receptor (GR), which repress CYP2R1 and thereby reduce bioactivation of vitamin D. These findings question the long-held assumption that vitamin D levels solely reflect intake and sun exposure.**

**Elkhwanky et al. (2020) showed that** **obesity suppresses CYP2R1, the enzyme responsible for vitamin D 25-hydroxylation, in both hepatic and extrahepatic tissues.**

**These two references below need to be cited and discussed in the discussion section:**

**Aatsinki SM*, Elkhwanky MS*, Kummu O, Karpale M, Buler M, Viitala P, Rinne V, Mutikainen M, Tavi P, Franko A, Wiesner RJ, Chambers KT, Finck BN & Hakkola J**

**(2019)** **Fasting-Induced Transcription Factors Repress Vitamin D Bioactivation, a**

**Mechanism for Vitamin D Deficiency in Diabetes. Diabetes 68(5): 918-931.**

**https://doi.org/10.2337/db18-1050IF: 6.2 Q1 .**

**Elkhwanky MS, Kummu O, Piltonen TT, Laru J, Morin-Papunen L, Mutikainen M,**

**Tavi P & Hakkola J (2020)** **Obesity Represses CYP2R1, the Vitamin D**

**25-Hydroxylase, in the Liver and Extrahepatic Tissues. JBMR PLUS https://doi.o**

**rg/10.1002/jbm4.10397IF: 3.4 Q2 .**

**These findings collectively** **challenge the traditional view that 25-hydroxylation is a stable, unregulated process, emphasizing instead the influence of metabolic state on vitamin D status. They also raise important questions about the suitability of 25(OH)D as a** **reliable biomarker in populations with metabolic disorders including diabetes.** **While 25(OH)D remains the most accepted marker of vitamin D status, these studies should be cited and discussed, particularly in relation to the study’s diabetic subgroup**

**Response:** Thank you very much for your insightful suggestion. This is an important issue and we totally agree that 25(OH)D may be not suitable for any conditions as a biomarker of vitamin D, though it is widely used. We have added this part in the limitation of this study (**Page 14, line371-381**). “Moreover, recent evidence challenge the traditional view that serum 25(OH)D is treated as a reliable biomarker of vitamin D, though it remains the most accepted marker of vitamin D status. For example, in individuals diagnosed with diabetes or obesity, the expression of CYP2R1, the enzyme responsible for vitamin D 25-hydroxylation, are suppressed, resulting in the decrease of 25(OH)D while vitamin D level is stable.”

**10. Line 261: Please remove either “diseases” or “disorders” to avoid redundancy.**

**Response:** we have removed the “disorders” to avoid redundancy.

**11. Lines 313–316: A reference on vitamin D toxicity should be included to support the statement.**

**Response:** a reference on vitamin D toxicity has been added at this paragraph.

43.	Marcinowska-Suchowierska E, Kupisz-Urbanska M, Lukaszkiewicz J, Pludowski P, Jones G. Vitamin D Toxicity-A Clinical Perspective. Front Endocrinol (Lausanne). 2018;9:550. Epub 2018/10/09. doi: 10.3389/fendo.2018.00550. PubMed PMID: 30294301; PubMed Central PMCID: PMCPMC6158375.

**12. Some phrases still imply causality (e.g., "vitamin D deficiency promotes aging"). Since this is a cross-sectional study, causal language must be avoided.**

**Response:** we are sorry for this mistake, and correct it.

**13. The suggestion that high vitamin D levels may accelerate aging in men appears speculative and requires either stronger supporting evidence or a more cautious tone.**

**Response:** we totally agree with your opinion. Our study could not conclude that high vitamin D levels may accelerate aging in men, and just found an interesting relationship between high vitamin D levels and biological aging. Further animal and clinical researches should be carried out to clarify this conclusion.

**14. English language revision is recommended.**

**Response:** we have done our best to solve this academic writing problem in the first revision. To further improve our writing, we have asked professional help. Thank you very much for your valuable review.

**In summary, although this is a timely and relevant study with notable improvements, several key areas including the clarity of the rationale, the discussion of mechanistic insights, and the interpretation of results, still require revision. Therefore, I recommend a major revision.**

**Reviewer #5: This is a study on chronological and biological aging and 25(OH-D-vitamin based on data from NHANES.**

**Major issues:**

**1. The first problem is how the authors treat age. Chronological age is simple, calculated biological age is also relevant. Phenoage is not used as defined by omitting some of the variables defining biological age.**

**Response:** We appreciate the reviewer’s insightful opinion. The algorithm we used to calculate PhenoAge omitted CRP values. For the current analysis, we used 5 cycles to achieve adequate sample size, 3 of which were absent of CRP data. We conducted additional correlation analyses between 2 PhenoAge estimates with and without CRP values. We found a high correlation of 0.986, suggesting that the exclusion of CRP did not significantly alter the overall estimates of PhenoAge. Additionally, this practice of omitting CRP was referenced form previous researches using the same NHANES datasets [3, 4].

We supplemented the following sentences in the **Methods**: “In this study, we utilized PhenoAge as the indicator of biological aging, considering its high robustness and feasibility in NHANES data as a reliable predictor of mortality and age-related disease risks. The PhenoAge algorithm is constructed using NHANES III (1988–1994) by elastic-net regression, encompasses 10 markers: albumin, creatinine, glucose, white blood cell count, lymphocyte percent, red cell distribution width, mean red cell volume, C-reactive protein, alkaline phosphatase and chronological age [22]. The R package BioAge was used to compute PhenoAge [16]. PhenoAge was both developed and validated within NHANES populations. Meanwhile, all constituent biomarkers are routinely measured in NHANES with standardized protocols, ensuring reproducibility across study cycles. One of the markers, CRP, was unavailable from 2011 to 2018. Previous studies have applied an algorithm that excluded CRP to calculate PhenoAge, demonstrating good applicability [23, 24]. To assess the validity of using PhenoAge without CRP data, we calculated 2 PhenoAge estimates with and without C-reactive protein, using data from cycles 2007-2008 and 2009-2010, which contained all 10 markers. We found a strong correlation between 2 PhenoAge estimates with and without CRP (correlation coefficient of 0.986). Based on these findings, we proceeded with the main analysis using the PhenoAge algorithm excluding CRP, employing data from five NHANES cycles.”. **(Page 5-6, line 118-140).**

Nevertheless, we acknowledge that this is a limitation of the current study and have explicitly acknowledged it in the **Discussion**: “Secondly, the PhenoAge algorithm we applied did not contain CRP data. This could be a limitation given that CRP is a key marker of inflammation and is potentially closely associated with vitamin D status. The absence of CRP in the PhenoAge calculation might lead to underestimation of the true relationship between vitamin D status and age acceleration. To gain a more accurate understanding of this association, future studies with more comprehensive datasets should be conducted.”. **(Page14, line371-381).**

The following reference was supplemented in the References List:

23.	Liu W, Wang J, Wang M, Hou H, Ding X, Ma L, et al. Oxidative Stress Factors Mediate the Association Between Life's Essential 8 and Accelerated Phenotypic Aging: NHANES 2005-2018. J Gerontol A Biol Sci Med Sci. 2024;79(1). Epub 2023/10/10. doi: 10.1093/gerona/glad240. PubMed PMID: 37813096.

24.	He H, Chen X, Ding Y, Chen X, He X. Composite dietary antioxidant index associated with delayed biological aging: a population-based study. Aging (Albany NY). 2024;16(1):15-27. Epub 2024/01/04. doi: 10.18632/aging.205232. PubMed PMID: 38170244; PubMed Central PMCID: PMCPMC10817368.

**2. The authors also define a concept called physiological age, which is also referenced in the title. This is a major problem.**

**Page 16 line “We further calculated physiological aging as the difference between PhenoAge and chronological age.“**

**This is a confusing terminology, not only for the readers, but the authors also have difficulties with this:**

**In the abstract line 21 “Biological age showed a negative correlation with serum 25(OH)D levels (β=-0.04; 95% CI: -0.08-0.00, p=0.039)”**

**In the results page 18 line 211: “physiological aging was found to be negatively associated with serum 25(OH)D levels (β=-0.04; 95% CI: -0.08-0.00).”**

**Omit the term' physiological age' and name it for what it really is.**

**Write instead: The difference between Phenoage and age was negatively correlated serum 25(OH)D levels …**

**Response:** We thank the reviewer for your insightful suggestions.

In agree with the editor’s suggestion, the terminology has been revised from "physiological aging" to "biological aging" throughout the manuscript, as this better aligns with the definition of PhenoAge—a composite metric integrating 10 clinical biomarkers that collectively reflect systemic inflammatory, metabolic, and hematopoietic dysfunction. In regression models, the standardized difference between PhenoAge and chronological age was included as the dependent variable. To enhance conciseness, we used the academic term “PhenoAgeAccel” as previously used by other literature [5, 6]. Throughout the Results, we consistently use "PhenoAgeAccel". When discussing accelerated biological aging processes, we used the term "biological aging". These revisions have been made throughout the whole manuscript.

In the **Methods** section, we have rewritten the following sentences to improve clarity: “We further calculated PhenoAge acceleration (PhenoAgeAccel) as the difference between PhenoAge and chronological age. A positive value of PhenoAgeAccel represents accelerated biological aging process, while a negative value represented slower biological aging process. Subsequently, PhenoAgeAccel was standardized to have a mean of 0 and a standard deviation (SD) of 1.” **(Page 6, line 125-140).**

**Major cohort problem**

**3. In NHANES, as in the rest of the US, race has been defined in different ways during the years. For example if one of the parents are of different races and one is white, the child is classified according to the race of the other parent. When neither parent is white the child is classified according to the fathers race. Other ways of defining race are in use, such as self-stated, decision by the doctor, or even name analysis. This is a major problem when discussing vitamin D in relation in a mixed cohort like the one used by the author. How big percentage would define race?**

**As has been thoroughly discussed about the inherent errors caused by race-adjusted equations for estimating glomerular filtration rate based on creatinine, this also applies to a study on vitamin D.**

**Response:** Thank you for your insightful comment regarding the classification of race in NHANES. Race definition indeed varies based on several criteria, including parental race and self-identification. These different definitions can lead to inconsistencies when analyzing health outcomes, such as vitamin D levels, across diverse populations. Understanding these complexities is essential for accurate interpretation of results and their implications in public health contexts. The lack of data segregation by race is a common study limitation of NHANES surveys in the absence of race-specific cut-off values for vitamin D levels as for other biological biomarkers such as the creatinine-based estimated glomerular filtration rate. Racial stratification has been used in studies of NHANES surveys, this is not the first report on 25(OH)D assessment in a multiracial NHANES population [7]. However, it should be more careful to use this race data, and we have added this in the limitation in the revised manuscript.

**4. The main determinant for 25(OH)-D-vitamin in serum/plasma is a genetic variant of vitamin D-binding protein = GC-globulin. There are three isoforms, Gc1, Gc1-2, and Gc2-2 isoforms. The genetic distribution of these isoforms is highly dependent on ethic origin. The three isoforms come with different 25(OH)-D vitamin levels, although this does not affect bioavailable vitamin D not measured in this and many other studies. Thus, there would be a need for** **different cut-offs for deficiency, depending on the isoforms. For example, vitamin D is associated with colorectal cancer only in one of these isoforms, and the isoforms modify the risk for bladder cancer.**

**Thus, it can be concluded that the NHANES study is not well suited for this kind of study applying knowledge from the last ten years. Perhaps if using whites only, but it is not biologically accurate to use a composite population like the authors do.**

**Response:** Thank you for your insightful comments regarding the influence of genetic variants of GC-globulin on serum levels of 25(OH)-D and the implications for different ethnic groups. This is an amazing idea applying different cut-offs for vitamin D deficiency depending on the isoforms. We acknowledge that the genetic distribution of these isoforms can significantly impact vitamin D levels and its associated health outcomes. However, we believe that our study's focus on the broader population sampling, including diverse ethnic backgrounds. Current analyses rely on total 25(OH)-D levels for practicality and this is the standard measurement. Several studies have investigated the role of vitamin D in various diseases based on the NHANES data [7-9]**.** Apparently, it would be better to analyze the isoform levels (Gc1, Gc1-2, Gc2-2) for a more detailed investigation, but NHANES currently lacks this data. With technological advancements and increasing demand, we hope that NHANES could expand and refine its relevant indicators to better accommodate various research needs.

**5. The authors state incorrectly that vitamin D is associated with cardiovascular disease and osteoporosis. This has been proven incorrect over the last decade. There remains an association with some inflammatory diseases such as multiple sclerosis and rheumatoid arthritis.**

**There is no “growing body of evidence” to support a statement of the potential of vitamin D supplementation to promote overall health.**

**Response:** studies found that vitamin D deficiency was associated with cardiovascular disease and osteoporosis. However, several large contemporary trials of vitamin D supplementation have failed to demonstrate significant benefits on cardiovascular disease, casting doubt on the observational evidence, as we described in the section of introduction. We agree with your comment, and rewrite this sentence to make it appropriate. Thank you very much.

As more evidence have been provided, the protective role of vitamin D or vitamin D supplementation has been challenged in some diseases. We need to exercise caution when stating that vitamin D serum concentrations are associated with diseases and that vitamin D supplementation is beneficial in disease prevention [10].

“These evidence highlights the potential of vitamin D supplementation in reducing the risk of several chronic diseases, particularly in elderly people, where serum vitamin D levels tend to decline with age.”

**Minor issues:**

**6. Vitamin D is more common in southern compared to northern Europe (PMID: 34933700IF: 3.0 Q2 ), as vitamin D is probably more dependent on food intake compared to sun exposure.**

**Response:** Thank you for your thoughtful comments. We agree with your opinion about the source of Vitamin D. studies report that the main source of vitamin D in human beings is vitamin D production by the skin, while food intake contributing to only up to 10% of the human body's store of vitamin D. however, in White people living in high latitude regions, food intake is the main source of vitamin D, as they are exposed to less sun-derived UVB radiations compared with other non-White population. In the current study, we analyzed the association of serum vitamin D level and biological aging, but did not investigate whether vitamin D is from sun exposure or food intake.

**7. The cut-off for deficiency at 50 nmol per liter is not solid, it is a consensus not anchored in facts. A more likely cut-off would be 25 nmol/L.**

**Response:** We appreciate the reviewer’s insightful comment. We fully recognize that vitamin D deficiency definitions remain contentious worldwide. Multiple scientific organizing committees have proposed distinct cutoff values for vitamin D deficiency status. Their suggestions stemmed from evidence in different target population, which means various factors would affect the population vitamin D status, such as UVB exposure, dietary habits, and genetic factors influencing vitamin D metabolism. In the current analysis, we categorize vitamin D level based on the Institute of Medicine (IOM) guidelines [1]. The IOM guidelines specifically addressed U.S. and Canadian populations with considerations for bone health outcomes, directly corresponding to the NHANES cohort's demographic characteristics. Hence, for operational clarity in cross-sectional analysis, we chose to reference the IOM criteria. In the **Methods**, we have rewritten the following sentences to improve clarity: “According to the Institute of Medicine Committee recommendation, we transformed serum 25(OH)D concentration into categorical variables: risk of deficiency (<30 nmol/L), risk of inadequacy (30–50 nmol/L), likelihood of sufficiency (50–75 nmol/L) and no increased benefit (≥75 nmol/L) [20,21].” **(Page 6, line 112-115).**

The following reference was supplemented in the References List:

20.	In: Ross AC, Taylor CL, Yaktine AL, Del Valle HB, editors. Dietary Reference Intakes for Calcium and Vitamin D. The National Academies Collection: Reports funded by National Institutes of Health. Washington (DC)2011.

21.	Ross AC, Manson JE, Abrams SA, Aloia JF, Brannon PM, Clinton SK, et al. The 2011 report on dietary reference intakes for calcium and vitamin D from the Institute of Medicine: what clinicians need to know. J Clin Endocrinol Metab. 2011;96(1):53-8. Epub 2010/12/02. doi: 10.1210/jc.2010-2704. PubMed PMID: 21118827; PubMed Central PMCID: PMCPMC3046611.

Nevertheless, we fully acknowledge that vitamin D deficiency threshold remain an ongoing controversy. We have expanded the Discussion to address this: “Our study identified a transition point in relationship between serum 25(OH)D level and biological aging, with males exhibiting a threshold at 38.20 nmol/L and females at 62.50 nmol/L. Beyond this transition point, the increase in 25(OH)D level no longer associates with biological aging deceleration. Regarding the threshold of vitamin D deficiency, it remains an ongoing controversy. The Scientific Advisory Committee on Nutrition (2016) recommended threshold at 25 nmol/L among UK population [46], whereas the IOM (2011) suggested thresholds at 30 nmol/L based on evidence in U.S. and Canadian populations [20]. Meanwhile, the European Food Safety Authority 2016 and the Endocrine Society guideline 2011 proposed higher level of 50 nmol/L as deficiency cutoff [47,48]. The discrepancy in cutoff values stems from multiple factors, for example, different regional ultraviolet B exposure patterns, dietary practice and population-specific biological variability. Furthermore, guideline variations also reflect different focus on health outcomes, with some prioritizing musculoskeletal health [49]. In 2024, the Endocrine Society guideline retracted their previous recommendation of threshold at 50 nmol/L due to limited clinical trial evidence [7]. It also highlighted the impracticality of universal thresholds due to heterogeneity in health outcomes and population characteristics. Hence, the interpretation of 25(OH)D level threshold determination in our study should be with caution, especially when it derived from observational study in an ethnically diverse population. Nevertheless, the trend of benefits attenuation we observed is worth noting, suggesting excessive level of serum 25(OH)D may not be beneficial regarding biological aging process.” **(Page 12, line332-352).**

The following reference was supplemented in the References List:

46.	England PH. SACN vitamin D and health report 2016 [updated 2025-04-22]. Available from: https://www.gov.uk/government/publications/sacn-vitamin-d-and-health-report.

47.	Turck D, Bresson J, Burlingame B. Scientific opinion on dietary reference values for vitamin D EFSA Panel on Dietetic Products, Nutrition, and Allergies (NDA). EFSA J. 2016;179.

48.	Holick MF, Binkley NC, Bischoff-Ferrari HA, Gordon CM, Hanley DA, Heaney RP, et al. Evaluation, treatment, and prevention of vitamin D deficiency: an Endocrine Society clinical practice guideline. J Clin Endocrinol Metab. 2011;96(7):1911-30. Epub 2011/06/08. doi: 10.1210/jc.2011-0385. PubMed PMID: 21646368.

49.	Roth DE, Abrams SA, Aloia J, Bergeron G, Bourassa MW, Brown KH, et al. Global prevalence and disease burden of vitamin D deficiency: a roadmap for action in low- and middle-income countries. Ann N Y Acad Sci. 2018;1430(1):44-79. Epub 2018/09/19. doi: 10.1111/nyas.13968. PubMed PMID: 30225965; PubMed Central PMCID: PMCPMC7309365.

**8. It is, however, interesting that higher levels are less beneficial in terms of biological age. This is not in line with the recommendations from organisations associated with the industry for supplementing vitamin D, aiming to increase vitamin D levels in the population.**

**Response:** Thank you for your comments. Our findings indicate that high levels of vitamin D are not associated with reduced aging; in fact, they may be linked to accelerated aging in males. While this is an intriguing observation, we cannot conclude that high vitamin D levels promote aging based on this cross-sectional study. Currently, we can only suggest a possible association, and further research is needed to clarify these results.

**Reviewer #6: In this manuscript by Feng et al, the authors aimed to explore the link between blood vitamin D levels and physiological aging, using data from over 13,000 U.S. adults in NHANES (2007–2016). The physiological aging was estimated using the PhenoAge model. The results revealed a nonlinear relationship: lower vitamin D levels were generally associated with accelerated aging, especially in females, while excessively high levels were linked to faster aging in males. These patterns suggest that both deficiency and excess of vitamin D may impact aging differently by gender. This revised manuscript has addressed most of the concerns raised by the Editor and Reviewer. However, there are a number of points that still need to be clarified. Specific comments are as follows:**

**1. The authors mentioned in the methods that CRP numbers were missing in some NHANES data and that PhenoAge (excluding CRP) was used. It would be helpful to briefly comment on how omitting CRP might affect comparability with studies using the standard PhenoAge metric.**

**Response:** We thank the reviewer for your constructive suggestion. For the current analysis, we used 5 cycles to achieve adequate sample size, 3 of which were absent of CRP data. We conducted additional correlation analyses between 2 PhenoAge estimates with and without CRP values. We found a high correlation of 0.986, suggesting that the exclusion of CRP did not significantly alter the overall estimates of PhenoAge. Additionally, this practice of omitting CRP was referenced form previous researches using the same NHANES datasets [3, 4].

We supplemented the following sentences in the **Methods**: “In this study, we utilized PhenoAge as the indicator of biological aging, considering its high robustness and feasibility in NHANES data as a reliable predictor of mortality and age-related disease risks. The PhenoAge algorithm is constructed using NHANES III (1988–1994) by elastic-net regression, encompasses 10 markers: albumin, creatinine, glucose, white blood cell count, lymphocyte percent, red cell distribution width, mean red cell volume, C-reactive protein, alkaline phosphatase and chronological age [22]. The R package BioAge was used to compute PhenoAge [16]. PhenoAge was both developed and validated within NHANES populations. Meanwhile, all constituent biomarkers are routinely measured in NHANES with standardized protocols, ensuring reproducibility across study cycles. One of the markers, CRP, was unavailable from 2011 to 2018. Previous studies have applied an algorithm that excluded CRP to calculate PhenoAge, demonstrating good applicability [23, 24]. To assess the validity of using PhenoAge without CRP data, we calculated 2 PhenoAge estimates with and without C-reactive protein, using data from cycles 2007-2008 and 2009-2010, which contained all 10 markers. We found a strong correlation between 2 PhenoAge estimates with and without CRP (correlation coefficient of 0.986). Based on these findings, we proceeded with the main analysis using the PhenoAge algorithm excluding CRP, employing data from five NHANES cycles.”. **(Page5-6, line 118-140).**

Additionally, we acknowledge that this is a limitation of the current study and have explicitly acknowledged it in the **Discussion**: “Secondly, the PhenoAge algorithm we applied did not contain CRP data. This could be a limitation given that CRP is a key marker of inflammation and is potentially closely associated with vitamin D status. The absence of CRP in the PhenoAge calculation might lead to underestimation of the true relationship between vitamin D status and age acceleration. To gain a more accurate understanding of this association, future studies with more comprehensive datasets should be conducted.”. **(Page 14, line371-381).**

The following reference was supplemented in the References List:

23.	Liu W, Wang J, Wang M, Hou H, Ding X, Ma L, et al. Oxidative Stress Factors Mediate the Association Between Life's Essential 8 and Accelerated Phenotypic Aging: NHANES 2005-2018. J Gerontol A Biol Sci Med Sci. 2024;79(1). Epub 2023/10/10. doi: 10.1093/gerona/glad240. PubMed PMID: 37813096.

24.	He H, Chen X, Ding Y, Chen X, He X. Composite dietary antioxidant index associated with delayed biological aging: a population-based study. Aging (Albany NY). 2024;16(1):15-27. Epub 2024/01/04. doi: 10.18632/aging.205232. PubMed PMID: 38170244; PubMed Central PMCID: PMCPMC10817368.

**2. In Line 220, the authors cite a p-value (p = 0.076) to describe the absence of dose-dependent trends in both the whole population and females. It is suggested to clarify this sentence to correctly distinguish between the groups with the right numbers.**

**Response:** thank you for your careful reading. We have corrected this mistake to make it clear.

**3. In the Discussion section (line 323), the authors state that serum 25(OH)D levels above 125 nmol/L in female participants were linked to accelerated physiological aging. However, this interpretation does not appear to be supported by the data in Table 3. Revising this statement to more accurately reflect the results is recommended.**

**Response:** thank you very much for your comments. We have revised as “Our results found the 25(OH)D levels over 91.5 nmol/L in male participants were not linked to accelerated biological aging, approximately consistent with those reports.”

**Reference**

1.	. Ross AC, Taylor CL, Yaktine AL, Del Valle HB, editors. Washington (DC)2011.

2.	McCartney CR, McDonnell ME, Corrigan MD, Lash RW. Vitamin D Insufficiency and Epistemic Humility: An Endocrine Society Guideline Communication. J Clin Endocrinol Metab. 2024;109(8):1948-54. Epub 2024/06/03. doi: 10.1210/clinem/dgae322. PubMed PMID: 38828961.

3.	He H, Chen X, Ding Y, Chen X, He X. Composite dietary antioxidant index associated with delayed biological aging: a population-based study. Aging (Albany NY). 2024;16(1):15-27. Epub 2024/01/04. doi: 10.18632/aging.205232. PubMed PMID: 38170244; PubMed Central PMCID: PMCPMC10817368.

4.	Liu W, Wang J, Wang M, Hou H, Ding X, Ma L, et al. Oxidative Stress Factors Mediate the Association Between Life's Essential 8 and Accelerated Phenotypic Aging: NHANES 2005-2018. J Gerontol A Biol Sci Med Sci. 2024;79(1). Epub 2023/10/10. doi: 10.1093/gerona/glad240. PubMed PMID: 37813096.

5.	Dan YL, Yang YQ, Zhu DC, Bo L, Lei SF. Accelerated biological aging as a potential risk factor for rheumatoid arthritis. Int J Rheum Dis. 2024;27(4):e15156. Epub 2024/04/26. doi: 10.1111/1756-185X.15156. PubMed PMID: 38665050.

6.	Jain P, Binder A, Chen B, Parada H, Jr., Gallo LC, Alcaraz J, et al. The Association of Epigenetic Age Acceleration and Multimorbidity at Age 90 in the Women's Health Initiative. J Gerontol A Biol Sci Med Sci. 2023;78(12):2274-81. Epub 2022/09/16. doi: 10.1093/gerona/glac190. PubMed PMID: 36107798; PubMed Central PMCID: PMCPMC10692424.

7.	Daraghmeh AH, Bertoia ML, Al-Qadi MO, Abdulbaki AM, Roberts MB, Eaton CB. Evidence for the vitamin D hypothesis: The NHANES III extended mortality follow-up. Atherosclerosis. 2016;255:96-101. Epub 2016/11/18. doi: 10.1016/j.atherosclerosis.2016.04.007. PubMed PMID: 27855294.

8.	Ma J, Li K. Negative Association between Serum Vitamin D Levels and Depression in a Young Adult US Population: A Cross-Sectional Study of NHANES 2007-2018. Nutrients. 2023;15(13). Epub 2023/07/14. doi: 10.3390/nu15132947. PubMed PMID: 37447273; PubMed Central PMCID: PMCPMC10346331.

9.	Liu C, Hua L, Xin Z. Synergistic impact of 25-hydroxyvitamin D concentrations and physical activity on delaying aging. Redox biology. 2024;73:103188. Epub 2024/05/14. doi: 10.1016/j.redox.2024.103188. PubMed PMID: 38740004; PubMed Central PMCID: PMCPMC11103937.

10.	Fang A, Zhao Y, Yang P, Zhang X, Giovannucci EL. Vitamin D and human health: evidence from Mendelian randomization studies. Eur J Epidemiol. 2024;39(5):467-90. Epub 2024/01/12. doi: 10.1007/s10654-023-01075-4. PubMed PMID: 38214845.
