# Supplementary material for: Association of 25(OH)D serum level with biological aging: A Cross-Sectional Study of 2007–2016 NHANES surveys
Source: PLoS One. 2025 Aug 6;20(8):e0328107. doi: 10.1371/journal.pone.0328107 (PMC12327616; doi:10.1371/journal.pone.0328107)
Supplement: Attachment — Submitted filename: response to comments-2.docx [file pone.0328107.s009.docx]

Dear editor Mickael Essouma and reviewers

We sincerely thank the reviewers for their approval of our manuscript and the editor for the valuable and constructive suggestions, especially the meticulous word-for-word revisions. We have carefully studied all comments and incorporated them thoroughly into our revisions. We are very grateful for the editor's dedicated support and hope the revised manuscript is now satisfactory. Should you have any questions, please feel free to contact us. We appreciate your continued consideration.

**1. Consider addressing the points 2, 3, 4 and 5 highlighted in the section "Additional Editor comments" of the Decision letter about manuscript R1 when discussing the study limitations in the discussion section.**

Response：The limitations have been revised in the manuscript, and the concerns raised in the "Additional Editor comments," including the vitamin D classification and race definition, have been addressed. Thank you so much.

**2. The first major reporting issue is the lack of clarity about the metrics of biological aging studied.  Was it PhenoAge alone, PhenoAge and PhenoAgeAccel, or PhenoAgeAccel alone? This issue stems from the fact that the term PhenoAge is inconsistently used throughout the manuscript. In the same vein, it seems to me that developers of PhenoAge termed it "DNAm PhenoAge". So, even if many authors keep using the term "PhenoAge" rather than "DNAm PhenoAge", I would use the term "DNAm PhenoAge" and consequently also the term "DNAm PhenoAgeAccel" when talking about PHenoAge Acceleration throughout the manuscript. The choice of the term to use between "DNAm PhenoAge" and "PhenoAge" (and consequently also between "DNAm PhenoAgeAccel" and "PhenoAgeAccel") is up to you, the only requeirement in this regard is consistency with the term chosen.**

Response： We have reviewed the entire manuscript and made corrections to use either "PhenoAge" or "PhenoAgeAccel" accordingly.

**3. A second major issue is that the authors do not use the PhenoAge classification (positive versus negative) when reporting on the association between 25(OH)D serum levels and PhenoAgeAccel throughout the manuscript, although they clearly mentioned that classification in the Materials and Methods section. Rather, they kept using the expression "reduced PhenoAgeAccel" which would need to be replaced by "negative PhenoAgeAccel" or perhaps "Decreasing PhenoAGeAccel". Therefore, consider implementing the recognised PhenoAgeAccel classification in the results and discussion sections of the manuscript.**

Response：A positive value of PhenoAgeAccel indicates an accelerated biological aging process, while a negative value indicates a slower biological aging process. We have revised "reduced/increased PhenoAgeAccel" to "negative/positive PhenoAgeAccel".

**4. A third major issue (linked to the other major study variable: 25(OH)D serum level) is that the authors sticked to the** **2011 IOM classification of 25(OH)D serum levels which includes four vitamin D serum level categories (sufficiency, insufficiency, deficiency and equal to or above 75 nmol/L) meanwhile they were advised to drop that classification given that the Endocrine Society no longer endorses that vitamin D classification status. I get it that the authors have the right to choose the classification system that best suits their needs. However, when doing so, they need to acknowledge in the limitations statement of the discussion section that the classification system used is now outdated and few evidence if any, currently to supports that classification system.**

Response： We have added this information in the section of limitation. “the classification of 25(OH)D serum levels used in our study is based on the 2011 IOM guidelines; however, more recent classifications, such as those from the Scientific Advisory Committee on Nutrition (2016), are considered more accurate. It would be preferable to analyze the association between vitamin D status and biological aging using these updated classifications”. Thank you very much.

**5. In addition, 25(OH)D serum levels need to be expressed using either or both of the two units ng/mL and/or nmol/L, but the unit chosen should be consistently used throughout the manuscript. I advise against using the term "correlation" throughout the manuscript, given the lack of correlation analyses in the manuscript. More comments and suggested edits are available in the document PONE-D-24-25180_R2_Mickael Essouma comments and suggested edits.pdf attached to this decision letter.**
Response：25(OH)D serum levels were expressed using nmol/L and we correct these through the whole manuscript.

In the revised manuscript, the term “correlation” has been replaced with “association”.

We have carefully reviewed all the comments in the document and accepted the suggested revisions. These suggestions have been very helpful in improving our manuscript. We are very grateful for the editor's support and revision. Thank you very much.
