# Supplementary material for: Association of 25(OH)D serum level with biological aging: A Cross-Sectional Study of 2007–2016 NHANES surveys
Source: PLoS One. 2025 Aug 6;20(8):e0328107. doi: 10.1371/journal.pone.0328107 (PMC12327616; doi:10.1371/journal.pone.0328107)
Supplement: Attachment — Submitted filename: RESPONSE-R4 20250623.docx [file pone.0328107.s011.docx]

Dear editor Mickael Essouma

We sincerely thank you for the valuable and constructive suggestions, especially the meticulous word-for-word revisions. We have carefully studied all comments and incorporated them thoroughly into our revisions. We are very grateful for the editor's dedicated support and hope the revised manuscript is now satisfactory. Should you have any questions, please feel free to contact us.

**1. Please review your reference list to ensure that it is complete and correct. If you have cited papers that have been retracted, please include the rationale for doing so in the manuscript text, or remove these references and replace them with relevant current references. Any changes to the reference list should be mentioned in the rebuttal letter that accompanies your revised manuscript. If you need to cite a retracted article, indicate the article’s retracted status in the References list and also include a citation and full reference for the retraction notice.**

**Response:** We have checked the references one by one, and no retracted article was listed in the manuscript. [22-24] and [50] were added in the references.

Additional Editor Comments:

**2. The authors have continued to improve upon their manuscript, but need to polish the presentation of the materials and methods section and further edit the manuscript to improve the language quality.**

**Response:** Thank you for your valuable feedback. We appreciate your suggestion to enhance the presentation of the materials and methods section. We will carefully review and revise this section to improve clarity, organization, and detail, ensuring that the methodology is clearly described and easily reproducible. Additionally, we will conduct a thorough language review throughout the manuscript to improve the overall quality and readability. We are committed to refining our manuscript accordingly and believe these revisions will strengthen the clarity and impact of our study.

Materials and Methods section.

**3. Lines 159-164: consider addressing my request to provide references supporting the classification made for sleep duration, hypertension and diabetes.**

**Response:** We thank the editor for your constructive suggestions. We have supplemented the following references to support the classification regarding sleep duration, hypertension and diabetes.

22.	Chaput JP, McNeil J, Despres JP, Bouchard C, Tremblay A. Seven to eight hours of sleep a night is associated with a lower prevalence of the metabolic syndrome and reduced overall cardiometabolic risk in adults. PLoS One. 2013;8(9):e72832. Epub 2013/09/17. doi: 10.1371/journal.pone.0072832. PubMed PMID: 24039808; PubMed Central PMCID: PMCPMC3764138.

23.	Muntner P, Hardy ST, Fine LJ, Jaeger BC, Wozniak G, Levitan EB, et al. Trends in Blood Pressure Control Among US Adults With Hypertension, 1999-2000 to 2017-2018. JAMA. 2020;324(12):1190-200. Epub 2020/09/10. doi: 10.1001/jama.2020.14545. PubMed PMID: 32902588; PubMed Central PMCID: PMCPMC7489367

24.	American Diabetes A. Diagnosis and classification of diabetes mellitus. Diabetes Care. 2014;37 Suppl 1:S81-90. Epub 2013/12/21. doi: 10.2337/dc14-S081. PubMed PMID: 24357215.

**4. As commented in the last round of review, you need to clearly specify that you conducted effect modification analyses for the association between 25(OH)D serum levels and PhenoAgeAccel in the statistical analysis sub-section before making the comment currently available in line 179. These references may be helpful in this respect: Edwarsd JR. Methods for Integrating Moderation and Mediation: A General Analytical Framework Using Moderated Path Analysis. Psychological Methods 2007, Vol. 12, No. 1, 1–22 and Knol et al. Recommendations for presenting analyses of effect modification and interaction. International Journal of Epidemiology 2012;41:514–520 doi:10.1093/ije/dyr218IF: 6.4 Q1 .This statement appears in the abstract: "Generalized additive models further found 25(OH)D serum 25 levels were linked to negative PhenoAgeAccel at levels below 38.2 nmol/L (15.3 ng/mL) in males (β=-0.013; 95% CI: -0.025 to -0.002) and 62.5 nmol/L (25.0 ng/mL) in females (β=-0.007; 95% CI: -0.01 to -0.004,), revealing a U-shaped relationship observed in males but an L-shaped pattern in females. . However, 25(OH)D serum levels above 125 nmol/L were not associated with PhenoAgeAccel in females (β=-0.001; 95% CI: -0.005-0.002), while in males, elevated levels (>91.55 nmol/L) were associated with positive PhenoAgeAccel (β=0.005; 95% CI: 0.002-0.008).". As requested in the last round of review, consider specifying in the methods section of the main manuscript the reasons why you chose those cut-off values for serum 25-vitamin D while performing the corresponding statistical analyses.**

**Response:** We thank the editor for your constructive suggestions. We have supplemented moderated regression analyses incorporating a multiplicative interaction term (25(OH)D × gender) to evaluate whether the relationship between vitamin D status and biological aging differs by gender. The results of these analyses revealed statistically significant gender-specific effects (p for interaction <0.001), indicating that the association between 25(OH)D and PhenoAgeAccel is moderated by gender. In the revised manuscript, we have supplemented the following sentences in the **Methods** section: “To evaluate potential effect modification by gender and other key covariates, subgroup analyses and interaction tests were conducted. Interaction term of serum 25(OH)D and stratifying factor were included in the multivariable linear regression models to test for significance of interaction.”

In the **Results** section, we have supplemented the following sentences: “Interaction test confirmed that association between serum 25(OH)D and PhenoAgeAccel was modified by gender, regardless of whether 25(OH)D was modeled as a continuous variable or categorized using quartiles or IOM guidelines (all p for interaction <0.001).”

Regarding the cut-off values identified in the segmented regression analysis, these were determined based on data-driven statistical criteria. Specifically, we first used a generalized additive model (GAM) with a smooth term for serum 25(OH)D levels to flexibly characterize the non-linear relationship with PhenoAgeAccel, as GAMs effectively capture complex dose-response patterns without assuming parametric forms. The GAM revealed distinct non-linear associations by sex, prompting further investigation using segmented regression—a method designed to formally identify inflection points (thresholds) where the slope of the association changes directionally. For each gender, optimal breakpoints were selected via likelihood ratio tests comparing nested models, ensuring statistical rigor in threshold estimation. These thresholds were computationally derived rather than manually assigned. However, we recognized potential ambiguity in how these thresholds were presented. To clarify, we have revised the following sentences in the **Abstract**: “25(OH)D serum levels were negatively associated with PhenoAgeAccel (β=-0.04 standard deviation [SD]; 95% confidence interval [CI]: -0.08 to 0.00). This negative association was dose-dependent in females (β=-0.07 SD; 95% CI: -0.12 to 0.01), but not in males. Generalized additive models further revealed gender-specific non-linear patterns: a U-shape pattern in males but an L-shaped pattern in females. Using segmented regression to confirm inflection points, we observed that 25(OH)D serum levels were linked to reduced PhenoAgeAccel at levels below 38.2 nmol/L (15.3 ng/mL) in males (β=-0.013 SD; 95% CI: -0.025 to -0.002) and 62.5 nmol/L (25.0 ng/mL) in females (β=-0.007 SD; 95% CI: -0.01 to -0.004,). However, 25(OH)D serum levels above 125 nmol/L showed no association with PhenoAgeAccel in females (β=-0.001 SD; 95% CI: -0.005-0.002), while in males, elevated levels (>91.55 nmol/L) were associated with increased PhenoAgeAccel (β=0.005 SD; 95% CI: 0.002-0.008).” Additionally, we have polished the language in the **Results** section to improve clarity.

.

**5. Results section. As commented in the last round of review, consider rounding all the numbers to one decimal place when providing results throughout the manuscript (Table 1 and so on).**

**Response:** We have corrected all the numbers to one decimal place.

Comment appended to Table 1.

**6. Discussion section: limitations statement. You also need to address the lack of data on vitamin D supplement among the confounders, the effect of unmeasured confounding and the reverse causation and residual confounding.**

**Response:** This information has been added in section of limitation.

**7. Comment about editing: Consider complying with PLOS ONE authors guidelines by not numbering the manuscript's sections and sub-sections.**

**Edits proposed in lines 123-125, 141-142, 178, 188, 251, 262,Table 1.**

**Response:** We have made corrections following the editor’s suggestion for our manuscript. Thank you for your valuable feedback.
